# Synthesis and Characterization of New Functionalized Pyrimidine (Hetero)Cyclic Molecular Hybrids as Chiral Heterocyclic Amino Acid Derivatives

**DOI:** 10.3390/molecules31152689

**Published:** 2026-08-02

**Authors:** Paulina Voznikaitė, Greta Račkauskienė, Miglė Dagilienė, Vilija Kederienė, Frank A. Sløk, Algirdas Šačkus

**Affiliations:** 1 Institute of Synthetic Chemistry, Kaunas University of Technology, K. Baršausko g. 59, LT-51423 Kaunas, Lithuania; paulina.voznikaite@ktu.lt (P.V.); migle.burinskaite@ktu.lt (M.D.); 2 Department of Organic Chemistry, Kaunas University of Technology, Radvilėnų pl. 19, LT-50254 Kaunas, Lithuania; vilija.kederiene@ktu.lt; 3 Vipergen ApS, Gammel Kongevej 23A, DK-1610 Copenhagen V, Denmark; fas@vipergen.com

**Keywords:** piperidine, pyrimidine, heterocyclic amino esters, *β*-keto esters, chirality, building blocks

## Abstract

Heterocyclic unnatural amino acids and their biheterocyclic derivatives represent invaluable structural scaffolds in modern medicinal chemistry and peptidomimetics due to their ability to induce conformational constraints and modulate pharmacokinetic profiles. While saturated nitrogen heterocycles and monocycle heteroaromatic systems are well-established pharmacophores, research on linear biheterocyclic amino acid frameworks remains significantly underrepresented in the literature. Addressing this structural gap, this study aims to synthesize and characterize a novel series of functionalized pyrimidine (hetero)cyclic molecular hybrids acting as chiral heterocyclic amino acid derivatives. The target pyrimidine-5-carboxylates and pyrimidine-4-carboxylic acid derivatives were prepared from *β*-dicarbonyl compounds and their corresponding enamine analogues via cyclocondensation approaches. Particular attention was paid to reaction optimization, substrate scope exploration, and stereochemical integrity preservation, utilizing chiral HPLC analysis to evaluate enantiomeric retention. Pyrimidine-5-carboxylates prepared from *β*-enamino keto esters retained high enantiomeric excess (90.2–100% ee), whereas cyclization of *β*-diketones under strongly basic conditions at elevated temperature resulted in complete racemization (ee < 1%). Modification of the synthetic route and application of milder cyclization conditions partially suppressed racemization, affording pyrimidine derivatives with 61.8–65.5% ee. These findings suggest that substrate structure influences stereochemical integrity during pyrimidine synthesis.

## 1. Introduction

**Figure 1:**
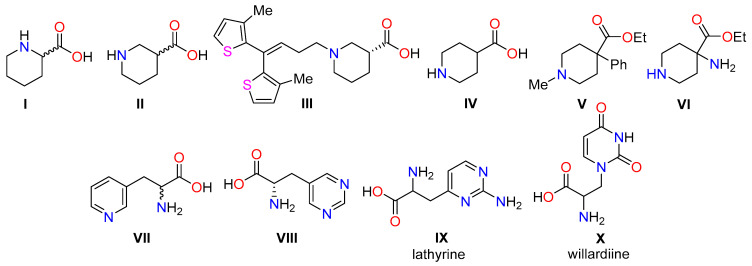
Examples of saturated heterocyclic and heteroaromatic amino acids.

Amino acids are organic compounds that serve as the fundamental building blocks of proteins and are also precursors to numerous organic molecules. These derivatives are widely used to improve performance in fields such as pharmaceuticals, materials science, food processing, and biotechnology [1,2,3,4]. In recent years, increasing attention has been paid to heterocyclic unnatural amino acids containing one or more heteroatoms (e.g., nitrogen, oxygen, or sulfur) in their cyclic structures [5,6,7]. Their significance is related to the different combinations of amino acid-type functional groups with heterocyclic frameworks, which may influence molecular shape, electronic properties, hydrogen-bonding capacity, basicity, lipophilicity, and metabolic stability [8,9]. Heterocycles are frequently found in biologically active compounds and approved molecule drugs, where they often participate in key interactions with biological targets [10,11,12,13]. Furthermore, natural and unnatural amino acids are widely used to modify peptides, and peptidomimetics conformational constraints are required [14]. In this respect, nitrogen-containing saturated heterocyclic and heteroaromatic amino acids represent valuable building blocks for preparing various constrained biological heterocyclic compounds, molecular hybrids [15,16], and peptides [6,14,17]. For example, piperidine derivatives represent an important class of saturated nitrogen heterocycles in medicinal and synthetic chemistry [18]. Among them, (*R*,*S*)-2-piperidine carboxylic acid **I**, also known as *D*,*L*-pipecolic acid, is a chiral cyclic non-protein amino acid and a proline homologue (Figure 1). Amino acid **I** is found naturally in microorganisms and plants and is a cornerstone of medicinal chemistry as a precursor for alkaloid synthesis [19]. (*R*,*S*)-piperidine-3-carboxylic acid (*D*,*L*-nipecotic acid) **II** is one of the most potent inhibitors of neuronal and glial *γ*-amino butyric acid (GABA) uptake in vitro [20]. Amino acid **II** is a building block for (*R*)-1-[4,4-bis-(3-methyl-2-thienyl)-3-butenyl]-3-piperidine carboxylic acid, named (*R*)-tiagabine **III**, which amplifies GABA neurotransmission, the predominant inhibitory neurotransmitter in the brain [21,22]. New derivatives of nipecotic acid **II** are very potent and are selective analogues of GABA uptake inhibitors [23,24,25]. Piperidine-4-carboxylic acid **IV**, also known as isonipecotic acid, is a conformationally constrained derivative of *γ*-aminobutyric acid (GABA) and a moderately potent GABA_A_ receptor partial agonist [26,27]; it is also a precursor to the drug pethidine **V**, also known as meperidine, which is an opioid as potent as morphine and can be administered intramuscularly [28,29].

From a synthetic perspective, piperidine carboxylic acid derivatives are attractive starting materials because their carboxyl group can be transformed into *β*-keto ester, enaminone, or related activated intermediates suitable for subsequent cyclocondensation reactions [30,31]. It is important to note that piperidine-4-carboxylic acid **IV** is often used as a substrate for modification to obtain various peptidomimetics [32]. In previous research, 4-aminopiperidine-4-carboxylic acid **VI** was used to create water-soluble highly helical peptides [33,34].

Meanwhile, aromatic heterocycles like benzene are ring-shaped organic molecules with a planar structure, an extended ring overlapping *p*-orbitals, and *π*-electrons that have at least one atom other than a hydrocarbon, such as nitrogen, oxygen, or sulfur, in the ring structure. Both synthetic and natural heteroaromatic amino acids, especially five- and six-membered rings containing nitrogen atoms which have unique stability and reactivity, are pharmacologically active compounds and have always attracted attention due to their pharmacological properties [35]. For example, 2-, 3-, and 4-pyridyl amino acids (commonly called regioisomeric pyridylalanines, where a pyridine ring replaces the phenyl ring of phenylalanine) are valuable non-canonical amino acids widely used in medicinal chemistry, pharmacology, and peptide engineering [36]. Heterocyclic alanines such as (*L*)-3-(2-pyridyl)alanine are useful precursors for preparing potential angiotensin **II** (AII) receptor antagonists [37,38]; 2-, 3-, and 4-pyridylalanines have attracted attention, as heteroaryl is substituted for phenylalanine, thus impacting VLA-4 inhibition. As such, these compounds may be considered for treating asthma and rheumatoid arthritis [39]. Moreover, these heterocyclic amino acids are used to form various biological complexes for transporting 1(LAT1) across the blood–brain barrier into cancer cells [40].

The pyrimidine ring is a structural unit of DNA and RNA and, therefore, chemical structures based on pyrimidine exhibit a variety of pharmacological activities, including antimalarial, antiviral, anticancer, anti-inflammatory, analgesic, anticonvulsant, and antioxidant effects [41,42]. Related synthetic pyrimidine amino acid derivatives, including *β*-pyrimidine alanine analogues, have also attracted interest as biologically important scaffolds and building blocks for modified peptides and biological heterocyclic compounds [43]. For example, Harrison et al. reported synthesizing functionalized urea compounds from heterocyclic alanines, including (2*R*)-2-amino-3-(pyrimidin-5-yl)propanoic acid **VIII,** as inflammasome inhibitors of NLRP3 [44]. Among this structurally diverse group are several natural pyrimidine-containing amino acids, including lathyrine **IX** and willardiine **X** [45]. Jane et al. demonstrated that willardiine analogues act as potent and selective agonists for either AMPA or kainate receptors, with receptor affinity and subtype selectivity strongly influenced by substitutions at the 5-position of the uracil ring. For example, 5-fluorowillardiine exhibited preferential affinity toward AMPA receptor subtypes, whereas 5-iodowillardiine showed high selectivity for the kainate receptor subtype hGluR5 [46].

Numerous methods for forming pyrimidine ring systems have been developed [47,48]. Classical condensation reactions to produce pyrimidines include condensation of the corresponding amidine derivative, which acts as a N-C-N nitrogen nucleophile, with three carbon units containing compounds such as *β*-dicarbonyl [49,50] or *β*-enaminone [51]. Furthermore, multicomponent reactions (MCRs) [52,53], including green chemistry approaches [54], have significantly expanded the set of synthetic tools available for synthesizing pyrimidines. The stereoselective synthesis of chiral pyrimidine derivatives continues to represent a notable synthetic challenge [55].

**Figure 2:**
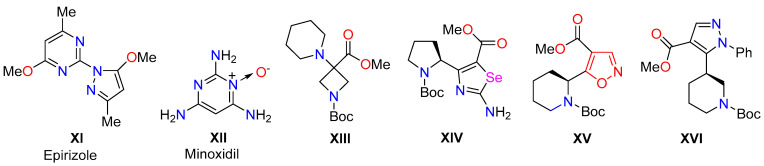
Examples of biheterocyclic drugs and amino acids.

Biheterocycles are an essential class of heterocyclic compounds in medicinal chemistry. Their design involves combining two pharmacophoric scaffolds into a single molecule. This combination enhances pharmacological potency, often exceeding the effects of each component alone [56,57]. Biheterocycles exhibit a variety of complex frameworks, including linear, angular, spiro, fused, and bridged heterocyclic rings and frequently serve as scaffolds for developing new biologically active molecules [58,59]. Linear biheterocycles can connect two heterocyclic rings with a single bond, and such molecules are found in many commercially available drugs. For example, the 2-(1*H*-pyrazol-1-yl)pyrimidine derivative Epirizole **XI** (Figure 2) is a nonsteroidal anti-inflammatory drug [60], the 4-(piperidin-1-yl)pyrimidine derivative Minoxidil **XII** is an anti-inflammatory agent [61], and the 3-(pyrimidin-4-yl)-1*H*-indole derivative Osimertinib (Tagrisso) is used to treat non-small-cell lung cancer [62].

The most common bicyclic amino acids contain two fused, spirocyclic, or bridged skeletons [63,64]; however, linear biheterocycle amino acids are much less well documented in the literature. Chalyk and co-workers obtained isoxazole-(hetero)cyclic molecular hybrids as linear biheterocyclic amino acid derivatives [65]. Our research group has synthesized various linear biheterocycle amino acid derivatives, for example, compound hybrids **XIII** [66], **XIV** [67], **XV** [31], and **XVI** [30], including chiral building blocks for synthesizing DNA-encoded compound libraries [68,69].

In this study, we synthesized and characterized new functionalized pyrimidine (hetero)cyclic molecular hybrids as chiral heterocyclic amino acid derivatives. The target pyrimidine-5-carboxylates and pyrimidine-4-carboxylic acid derivatives were prepared from *β*-dicarbonyl compounds and their enamine analogues via cyclocondensation approaches. An important objective of this study was to establish complementary synthetic routes toward new pyrimidine–heterocyclic amino acid derivatives while preserving the configuration of the inherited stereogenic center during pyrimidine formation. Particular attention was given to reaction optimization, substrate scope, and stereochemical integrity, with chiral HPLC analysis used to evaluate enantiomeric retention. The majority of the substrates showed excellent stereochemical retention, but select structurally distinct examples underwent enantiomeric purity erosion.

## 2. Results and Discussion

**Scheme 1:**
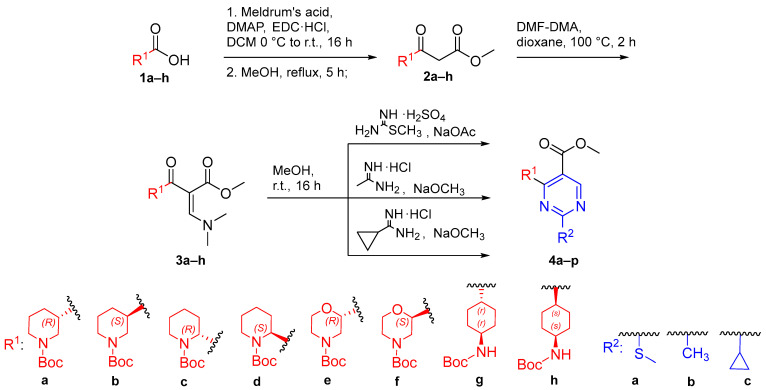
Synthesis of compounds **4a**–**p**.

**Figure 3:**
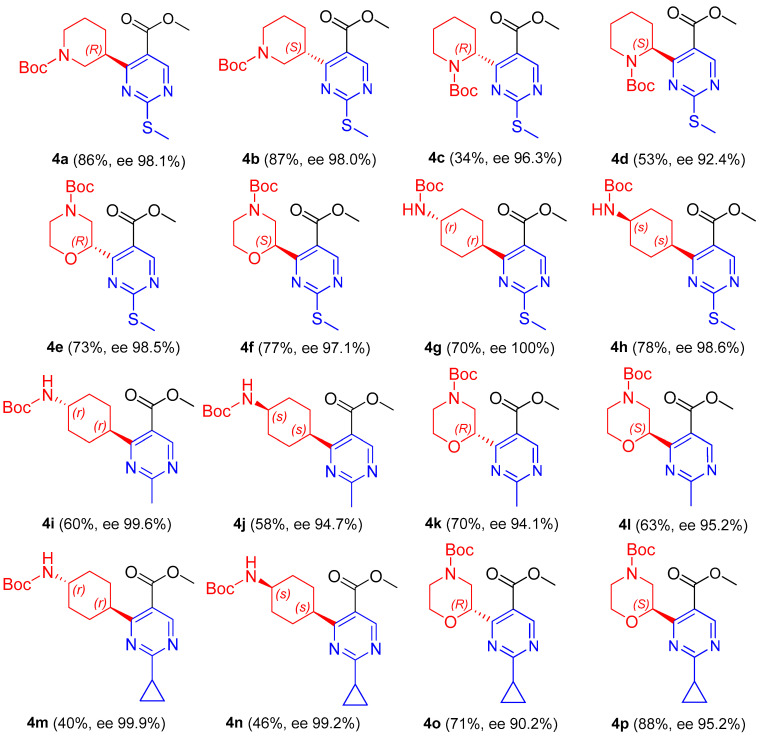
Synthesized compounds **4a**–**p**.

In our investigation, we started with synthesizing the novel (hetero)cyclic pyrimidine-5-carboxylates (Scheme 1 and Figure 3). The starting *β*-keto esters **2a–h** were synthesized from corresponding *N*-Boc protected amino acids **1a–h**, which were first treated with 2,2-dimethyl-1,3-dioxane-4,6-dione (Meldrum’s acid) under the action of 4-dimethylaminopyridine (DMAP) and *N*-ethyl-*N*′-(3-dimethylaminopropyl)carbodiimide hydrochloride (EDC⋅HCl), followed by further methanolysis of the Meldrum’s acid adduct [30,70,71].

The obtained compounds **2a–h** were converted into *β*-enamino keto esters **3a–h** by treatment with *N*,*N*-dimethylformamide dimethyl acetal (DMF−DMA) [30,71,72]. After successfully preparing **3a–h**, we investigated the formation of 2,6-substituted pyrimidine-5-carboxylates **4a–p** (Scheme 1 and Figure 3).

Initially, the synthetic approach involved synthesizing thiopyrimidine-5-carboxylates **4a–h**. Various reaction conditions have been reported in the literature for synthesizing substituted thiopyrimidine derivatives [73,74,75,76]. For instance, Goldber et al. described the cyclocondensation of enamine with 2-methyl-2-thiopseudourea sulfate mediated by sodium acetate [77]. Following the aforementioned procedure, *β*-enamino keto esters **3a–h** were treated with 2-methyl-2-thiopseudourea hemisulfate in dry methanol in the presence of anhydrous sodium acetate at room temperature for 16 h, affording the target 2-(methylsulfanyl)pyrimidine-5-carboxylates **4a–h** with moderate-to-good yields (34–87%).

Then, the cyclocondensation strategy was employed to synthesize pyrimidine-5-carboxylates **4i–p** using amidine reagents. Initially, the synthesis of compound **4i** was optimized to assess the base and reaction time effects on the formation of the target product. The reaction was carried out in methanol at room temperature using a range of organic and inorganic bases, with reaction times of 4, 16, and 48 h (Table 1). After 4 h, moderate yields were afforded with most of the bases investigated, with NaOCH_3_ providing the highest yield (45%). The lowest yields of product **4i** were observed in the presence of weaker bases such as TEA, K_2_CO_3_, and DIPEA. Extending the reaction time to 16 h improved the **4i** yield with several bases (NaOCH_3_, K_2_CO_3_). No further improvement in yield was observed when the reaction time was extended beyond 16 h; in some cases, yield decreased. The best yield for **4i** (60%) was obtained when the reaction was carried out in methanol at room temperature over 16 h, employing NaOCH_3_ (Table 1, Entry 4). The lower isolated yields obtained after prolonged reaction times are most likely attributable to competing side reactions during extended exposure to the basic reaction conditions. The optimized conditions were subsequently applied to the synthesis of a series of pyrimidine-5-carboxylates **4i–p** (Figure 3).

**Figure 4:**
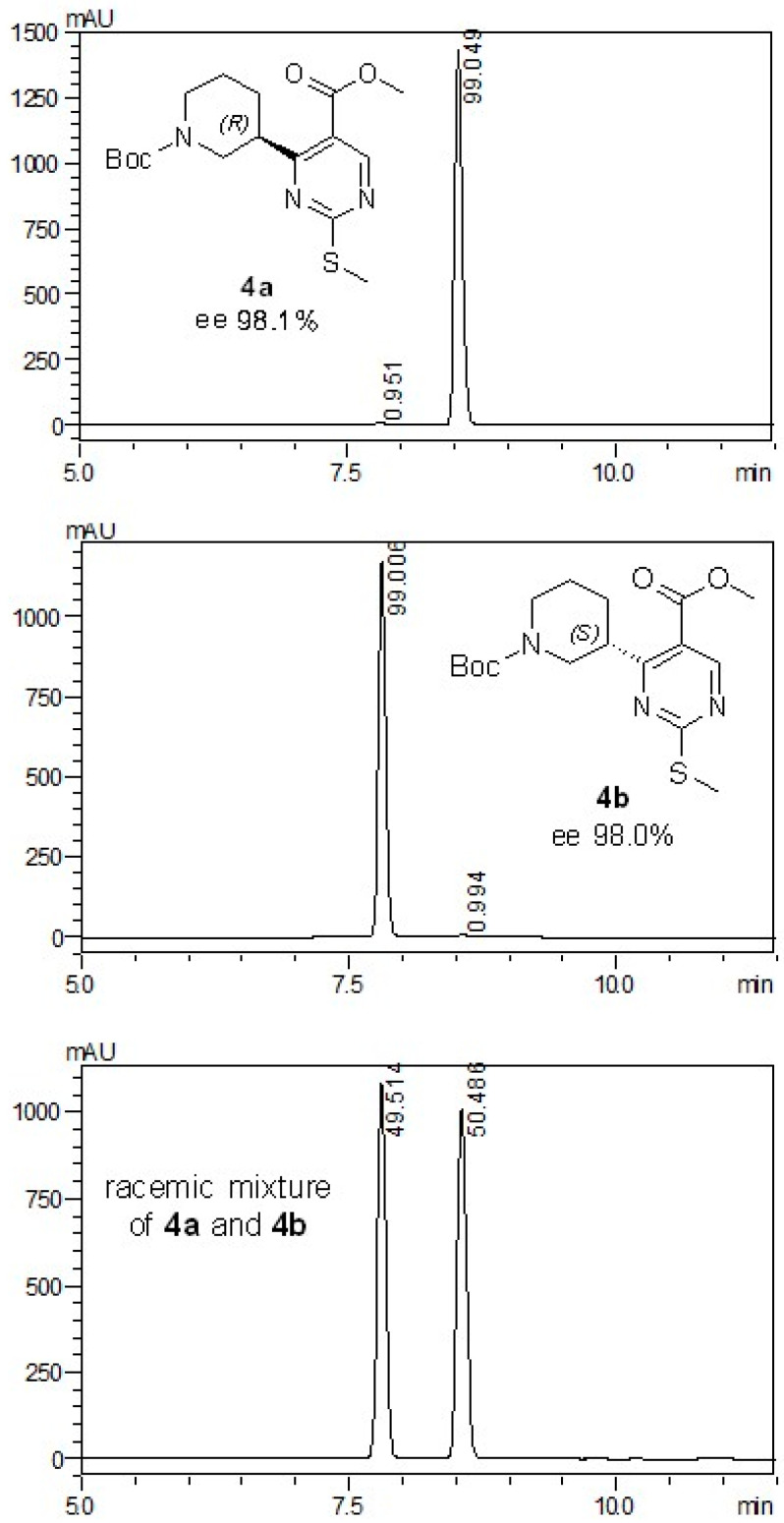
Chiral HPLC analysis of pyrimidine-5-carboxylates **4a**, **4b** and the corresponding racemic mixture. Conditions: CHIRAL ART Cellulose-SJ (100 × 4.6 mm I.D.); mobile phase: ACN/(H_2_O + 0.1% HCOOH) (gradient from 30:70 to 70:30 in 10 min); T = 36 °C; flow rate 1.0 mL/min, UV 254 nm.

The enantiomeric purity of pyrimidine-5-carboxylates **4a–p** was determined by chiral HPLC analysis. Representative chromatograms of compounds **4a** and **4b** are presented in Figure 4, while chromatograms of the remaining compounds **4c–p** are provided in the Supplementary Materials. The chiral pyrimidine-5-carboxylate (*R*)-**4a** was obtained from the corresponding *β*-enamino keto ester (*R*)-**3a** with an enantiomeric excess of 98.1%, whereas the corresponding (*S*)-**4b** enantiomer was obtained from the *β*-enamino keto ester (*S*)-**3b** with an enantiomeric excess of 98.0%.

**Figure 5:**
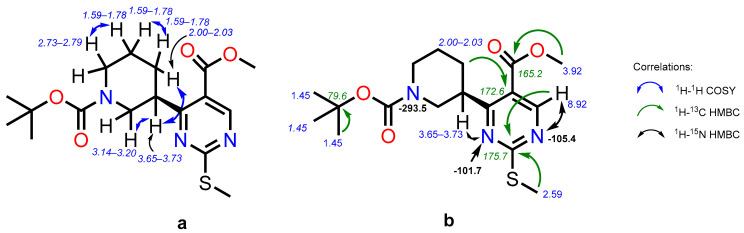
(**a**) Relevant ^1^H-^1^H COSY correlations along with ^1^H NMR (blue) chemical shifts for compound **4a.** (**b**) Relevant ^1^H-^13^C HMBC and ^1^H-^15^N correlations along with ^1^H NMR (blue), ^13^C NMR (green), and ^15^N NMR (black, bold) chemical shifts for compound **4a**.

The structures of substituted pyrimidine-5-carboxylates **4a–p** were elucidated through a combination of standard and advanced NMR spectroscopy techniques, including ^1^H, ^13^C, ^13^C DEPT-135, ^1^H-^1^H COSY, ^1^H-^13^C HSQC, ^1^H-^13^C HMBC, and ^1^H-^15^N HMBC. Compound **4a** was subjected to detailed NMR analysis (Figure 5).

The ^1^H NMR spectrum showed a tert-butyl singlet at *δ*_H_ 1.45 ppm (9H), indicative of a Boc-protecting group. A methoxy signal at *δ*_H_ 3.92 ppm correlated with *δ*_C_ 52.5 ppm in the HSQC spectrum and displayed a long-range HMBC correlation to the carbonyl carbon at *δ*_C_ 165.2 ppm, confirming the methyl ester moiety.

The aliphatic fragment was unambiguously defined by ^1^H-^1^H COSY correlations, which established a continuous spin system from 2-H through 6-H. HSQC data enabled assigning all protonated carbons within this fragment, including signals at *δ*_C_ 47.7 ppm (C-2), 41.0 ppm (C-3), 30.8 ppm (C-4), 25.2 ppm (C-5), and 44.2 ppm (C-6). Key HMBC correlations from 4-H to the quaternary carbon (C-5′) and 3-H ^3^*J* correlation to C-4′ provided direct evidence for attaching the aliphatic chain to the heteroaromatic ring.

The heteroaromatic system was further elucidated using long-range ^1^H-^13^C HMBC correlations. The downfield proton 6′-H (*δ*_H_ 8.92 ppm) exhibited correlations with C-4′ (*δ*_C_ 172.6 ppm) and C-2′ (*δ*_C_ 175.7 ppm), consistent with its position within an electron-deficient heterocycle. Additional HMBC correlations of protons in the linker region with aromatic carbons reinforced the connectivity between the two fragments. The observed chemical shifts and correlation pattern are in agreement with a substituted *N*-containing heteroaromatic ring bearing electron-withdrawing substituents.

The position of heteroatoms within the ring system was confirmed by ^1^H-^15^N HMBC data. Correlations from H-6′ and neighbouring protons to distinct nitrogen resonances established the arrangement of nitrogen atoms within the heterocycle. Furthermore, the S-methyl group (*δ*_H_ 2.59 ppm; *δ*_C_ 14.5 ppm) showed clear HMBC correlations to C-2′, confirming its attachment to sulfur and its position relative to the ring system.

**Scheme 2:**
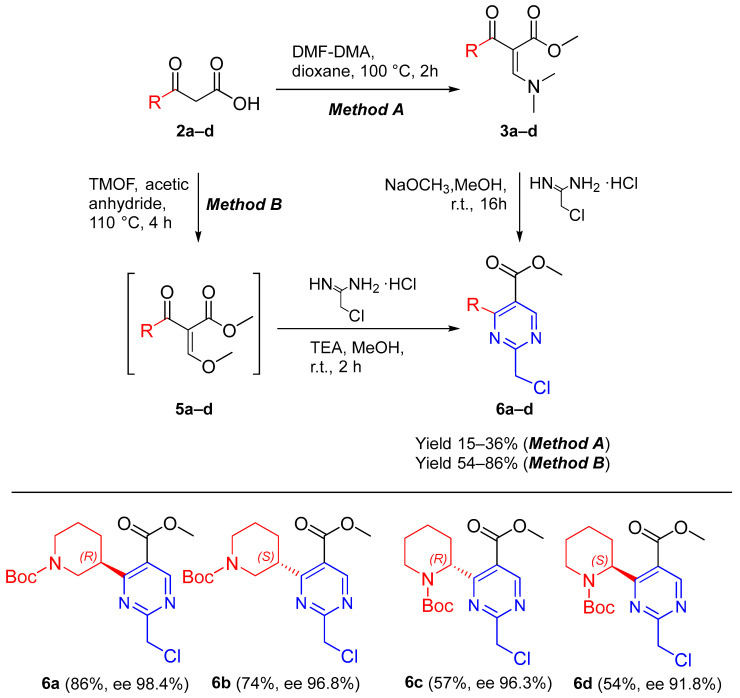
Synthesis pathways of compounds **6a–d**.

Next, we investigated the cyclization of *β*-enamino keto esters **3a–d** with 2-chloroacetimidamide hydrochloride (Scheme 2); however, the previously optimized reaction conditions proved unsuitable. After 16 h, treating *β*-enamino keto esters **3a–d** with 2-chloroacetimidamide hydrochloride, using NaOCH_3_ as the base, in methanol at room temperature afforded the desired 2-(chloromethyl)pyrimidine-5-carboxylates **6a–d** only in low yields (15–36%). To address this limitation, a known synthetic procedure was adapted to prepare 2-(chloromethyl)pyrimidine-5-carboxylates **6a–d** [78,79]. The appropriate *β*-keto esters **2a–d** initially reacted with trimethyl orthoformate (TMOF) in the presence of acetic anhydride to afford intermediates **5a–d** (Scheme 2). Without isolating intermediates, triethylamine and 2-chloroacetimidamide hydrochloride were added in the next step, forming targeted products **6a–d** in good total yields of 54–86%.

**Scheme 3:**
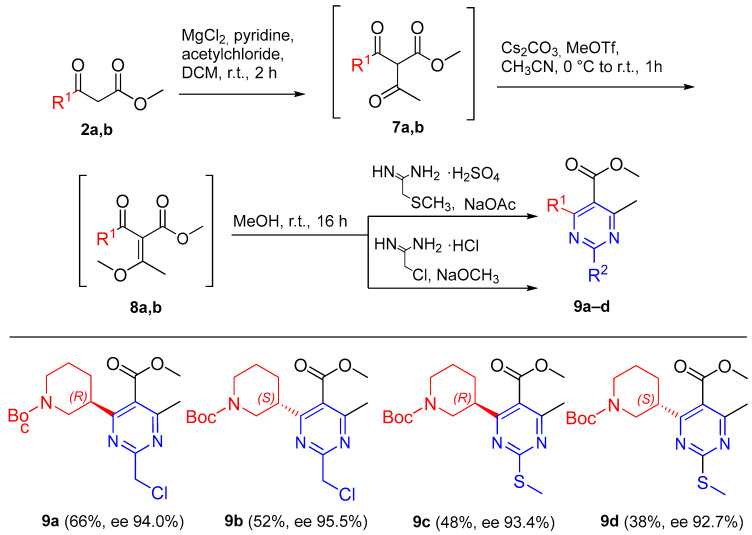
Conversion of diketones to pyrimidine-5-carboxylate derivatives **9a–d**.

Following the successful cyclocondensation reactions of *β*-keto esters **2**, pyrimidines **9a–d** were synthesized using different synthetic approaches (Scheme 3). In this case, substituted pyrimidine-5-carboxylates **9a–d** were obtained in three steps from the *β*-keto esters **2a,b**, following modified procedures previously applied to other compounds in the literature [80,81]. Initially, *β*-keto esters **2a,b** were converted into the corresponding acetoacetates **7a,b** using acetyl chloride in the presence of MgCl_2_ (Scheme 3). Acetoacetates **7a,b** were then treated with methyl trifluoromethanesulfonate under basic conditions at room temperature to give the intermediates **8a,b**. Finally, cyclization of compounds **8a,b** with methyl-2-thiopseudourea hemisulfate or 2-chloroacetimidamide hydrochloride under the conditions described above resulted in compounds **9a–d** in good yields (38–66%).

**Scheme 4:**
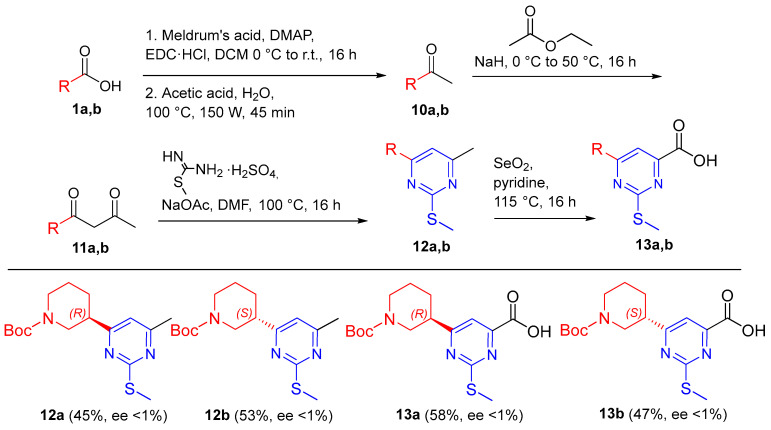
Synthesis of compounds **12a,b** and **13a,b**.

Pyrimidine derivatives play an important role in organic chemistry. In particular, 2-(methylsulfonyl)pyrimidines are broadly used in agrochemical and pharmaceutical applications [82,83]. Moreover, Mao et al. reported pyrimidine carboxylic acid derivatives exhibiting potent xanthine oxidase inhibitory activity [84,85]. Following the synthesis of pyrimidine-5-carboxylates, we further prepared several pyrimidine-4-carboxylic acids (Scheme 4). First, *β*-diketones **11a,b** were synthesized from the corresponding ketones **10a,b** via Claisen-type condensation [86]. Subsequent cyclization of *β*-diketones **11a,b** with 2-methyl-2-thiopseudourea hemisulfate using weaker basic conditions afforded 2-(methylsulfonyl)pyrimidines **12a,b** (45–53%).

Selenium dioxide-mediated oxidation of methylpyrimidines is a well-established method for synthesizing pyrimidine carboxylic acids [87]. Accordingly, oxidizing 4-methyl-2-(methylsulfonyl)pyrimidines **12a,b** with excess selenium dioxide in boiling pyridine resulted in pyrimidine-4-carboxylic acids **13a,b** in moderate yields (46–57%).

**Figure 6:**
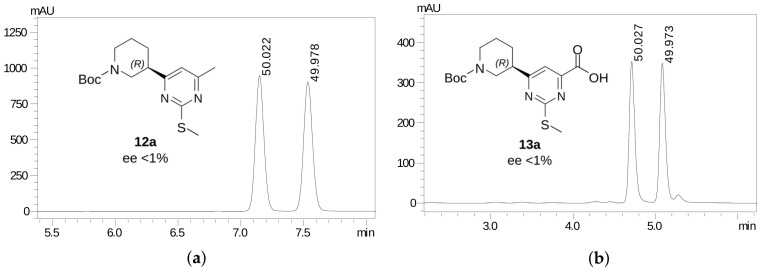
Chiral HPLC analysis of pyrimidine derivatives (**a**) **12a** and (**b**) **13a**. Conditions: CHIRAL ART Cellulose-SJ (100 × 4.6 mm I.D.); mobile phase: ACN/(H_2_O + 0.1% HCOOH) (gradient from 30:70 to 70:30 in 10 min); T = 36 °C; flow rate 1.0 mL/min, UV 254 nm.

Nevertheless, this synthetic approach resulted in a complete loss of stereochemical integrity, affording racemic products. Treating compounds **11a,b** with 2-methyl-2-thiopseudourea hemisulfate in DMF using anhydrous sodium acetate at an elevated temperature resulted in complete racemization, affording pyrimidines **12a,b** as racemic mixtures (**12a**, ee < 1%; **12b**, ee < 1%), which could not be separated into individual enantiomers (Figure 6). The subsequent oxidation of compounds **12a,b** afforded pyrimidine-4-carboxylic acids **13a,b** as inseparable racemic mixtures (**13a**, ee < 1%; **13b**, ee < 1%).

**Scheme 5:**
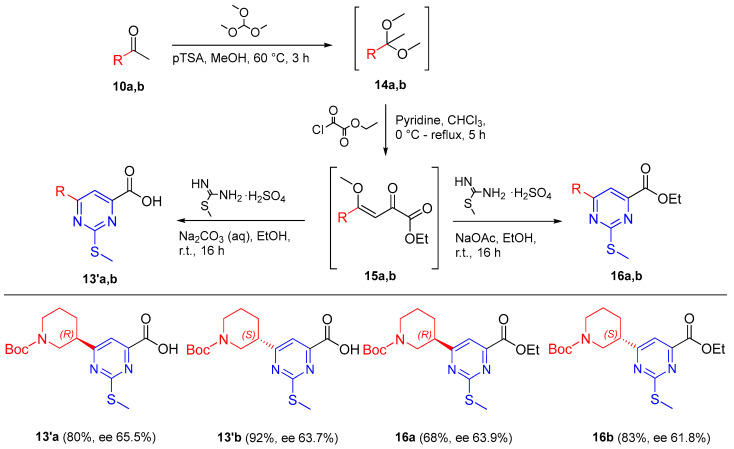
Synthesis of pyrimidine analogues **13′a,b** and **16a,b**.

Due to the complete loss of stereochemical integrity under the applied reaction conditions, a different synthetic strategy was developed to access pyrimidine derivatives with improved enantiomeric purity, as outlined in Scheme 5.

Adjusting the approach, ketones **10a,b** initially reacted with trimethyl orthoformate in methanol in the presence of pTSA to yield intermediate compounds **14a,b**. The solvent was removed under reduced pressure, after which ethyl oxalyl chloride and pyridine were added at 0 °C using chloroform as a solvent. The reaction mixture was stirred for 1 h at 0 °C and subsequently refluxed for 5 h, resulting in intermediates **15a,b** [88]. In this case, the cyclization reactions were carried out under mild conditions. In the first route, **15a,b** reacted with 2-methyl-2-thiopseudourea hemisulfate in ethanol in the presence of sodium carbonate at room temperature, and pyrimidine carboxylic acids **13′a,b** were obtained in good-to-excellent yields of 80–92%. In the second synthesis route, **15a,b** reacted with 2-methyl-2-thiopseudourea hemisulfate in the presence of anhydrous sodium acetate at room temperature in absolute ethanol to afford the corresponding pyrimidine esters **16a,b** in good yields (68–83%).

**Figure 7:**
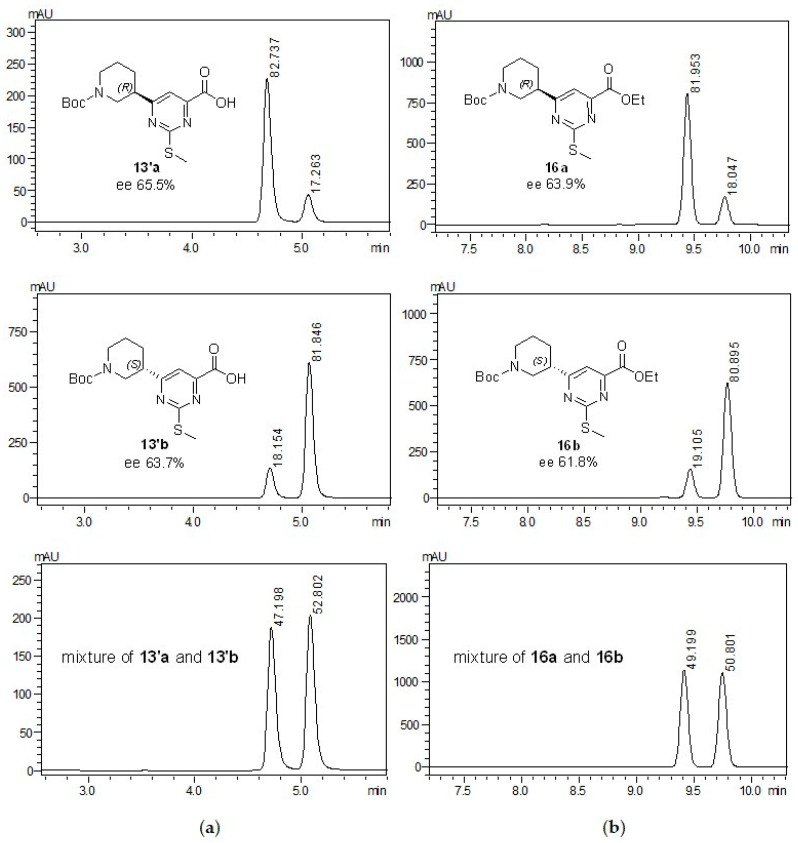
Chiral HPLC analysis of pyrimidine derivatives (**a**) **13′a**, **13′b**, and the corresponding racemic mixture; (**b**) **16a** and **16b**, and the corresponding racemic mixture. Conditions: CHIRAL ART Cellulose-SJ (100 × 4.6 mm I.D.); mobile phase: ACN/(H_2_O + 0.1% HCOOH) (gradient from 30:70 to 70:30 in 10 min); T = 36 °C; flow rate 1.0 mL/min, UV 254 nm.

The enantiomeric purity of the obtained pyrimidines **13′a,b** and **16a,b** was evaluated using chiral HPLC analysis. Representative chiral HPLC chromatograms of pyrimidine carboxylic acids **13′a,b** and pyrimidine esters **16a,b** are shown in Figure 7. Under milder cyclization conditions, complete racemization was avoided, and the products were obtained with moderate enantiomeric purities (**13a**, 65.5% ee; **13b**, 63.7% ee; **16a**, 63.9% ee; **16b**, 61.8% ee).

To identify the stage at which stereochemical erosion occurred, additional chiral HPLC analyses of key intermediates from the synthetic routes shown in Scheme 4 and Scheme 5 were performed (The corresponding chiral HPLC chromatograms of intermediates **10a,b** and **11a,b** are provided in the Supplementary Information as Figures S136 and S137). The ketone intermediates **10a,b** were found to be enantiomerically pure. In contrast, the corresponding intermediates **11a,b** exhibited identical chromatographic profiles irrespective of the configuration of the starting ketone, demonstrating that complete racemization had already occurred during the Claisen reaction.

**Scheme 6:**
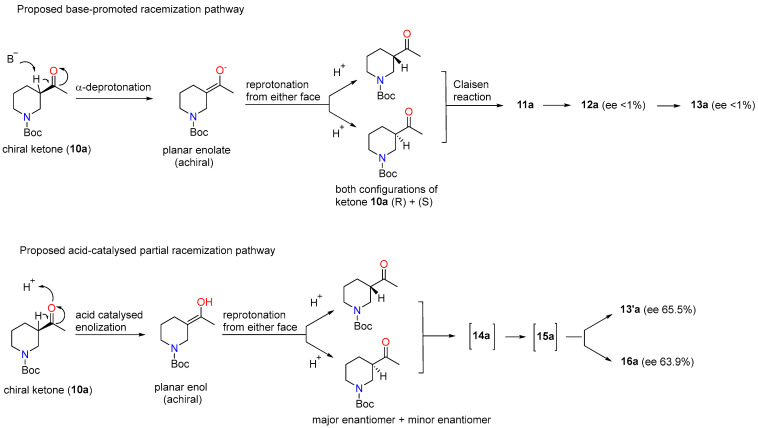
Plausible explanation for the observed complete and partial racemization under basic and acid-catalysed reaction conditions.

For the alternative synthetic route (Scheme 5), the starting ketones **10a,b** were likewise enantiomerically pure, whereas the final products **13′a,b** and **16a,b** retained moderate enantiomeric excesses (61.8–65.5% ee). Since the corresponding intermediates **14a,b** and **15a,b** were not isolated, the exact stage of stereochemical erosion could not be established. A plausible explanation for the different extents of racemization observed under the two reaction pathways is presented in Scheme 6.

The proposed racemization pathways shown in Scheme 6 provide a plausible explanation for the different stereochemical outcomes observed under the two synthetic routes. Under the strongly basic Claisen reaction conditions, where a stoichiometric amount of base is employed, deprotonation at the stereogenic α-carbon generates a planar enolate. Subsequent non-stereoselective reprotonation from either face results in complete loss of stereochemical integrity. In contrast, the alternative synthetic route employs catalytic amounts of *p*-toluenesulfonic acid. Under these milder acid-catalysed conditions, partial racemization is most plausibly associated with reversible keto–enol tautomerization, which proceeds to a significantly lesser extent than under the strongly basic conditions. Consequently, only partial erosion of enantiomeric excess is observed, consistent with the experimentally determined chiral HPLC data.

The different stereochemical outcomes observed for the two synthetic approaches can also be rationalized by the nature of the reaction intermediates. In the synthetic routes shown in Scheme 1, Scheme 2 and Scheme 3, pyrimidine derivatives are formed from conjugated β-enamino carbonyl or related intermediates, in which the carbonyl functionality is incorporated into a conjugated system. Consequently, the transformation proceeds under mild conditions without requiring deprotonation at the stereogenic centre, thereby preserving the inherited stereochemical information, as reflected by the high enantiomeric excesses (90.2–100% ee) observed for the corresponding products.

## 3. Materials and Methods

### 3.1. General Information

All starting materials were purchased from commercial suppliers and were used as received. Flash column chromatography was performed on Silica Gel 60 Å (Merck KGaA, Darmstadt, Germany). Vacuum distillation was performed in a Büchi Model B580 GKR oven (Büchi Labortechnik AG, Flawil, Switzerland). Thin-layer chromatography was carried out on Silica Gel plates (Merck Kieselgel 60 F254) and visualised by UV light (254 nm) (Merck KGaA, Darmstadt, Germany). The IR spectra were recorded on a Bruker Vertex 70v FT-IR spectrometer (Bruker Optik GmbH, Ettlingen, Germany) using neat samples and are reported in the frequency of absorption (cm^–1^). Mass spectra were obtained using a Shimadzu LCMS-2020 (ESI^+^) spectrometer (Shimadzu Corporation, Kyoto, Japan). High-resolution mass spectra were measured using a Bruker MicrOTOF-Q III (ESI^+^) apparatus (Bruker Daltonik GmbH, Bremen, Germany). Accurate measurements were achieved using the internal mass calibration of each sample using sodium formate calibration solution as a standard procedure, with a standard deviation always less than 1 ppm. In addition, all data files were recalibrated with an internal standard of sodium formate injected prior to initial sample elution for each sample. Optical rotation data were recorded on a UniPol L SCHMIDT+HAENSCH polarimeter (concentration of compound (g/100 mL) and were included in calculations automatically (Windaus-Labortechnik GmbH & Co. KG, Clausthal-Zellerfeld, Germany). ^1^H-NMR and ^13^C-NMR spectra were recorded from CDCl_3_ solutions at 25 °C on a Bruker Avance III 400 instrument (400 MHz for ^1^H, 100 MHz for ^13^C) using a directly detecting BBO probe (Bruker Bio Spin International AG, Faellanden, Switzerland). ^15^N-NMR spectra were recorded from CDCl_3_ solutions at 25 °C on a Bruker Avance III 400 instrument (40 MHz for ^15^N) using a directly detecting BBO probe. The chemical shifts (*δ*), expressed in ppm, were relative to tetramethylsilane (TMS). ^15^N-NMR spectra were referenced against neat external nitromethane (coaxial capillary). The following abbreviations were used in reporting the NMR data: Pyr, pyrimidine, Pip, piperidine; Cpr, cyclopropane; Morph, morpholine; Cy, cyclohexane.

### 3.2. Synthetic Procedures

#### 3.2.1. General Procedure for Compounds **2a–h**

Corresponding amino acid (1 equiv.), Meldrum‘s acid (1.1 equiv.) and DMAP (2 equiv.) were dissolved in methylene chloride and cooled to 0 °C. Maintaining the temperature at 0 °C, 1-ethyl-3-(3-dimethylaminopropyl)carbodiimide hydrochloride (1.1 equiv.) was added in portions and the solution was stirred at r.t. for 16 h. The reaction mixture was diluted with DCM (10 mL) and washed with 1M KHSO_4_ (2 × 15 mL) and brine (15 mL). The organic layer was dried with anhydrous sodium sulfate, filtered, and then concentrated under reduced pressure. The crude product was dissolved in methanol and stirred at 60 °C for 5 h. After removal of the solvent in vacuo, the residue was directly used in the next step without further purification.

#### 3.2.2. General Procedure for Compounds **3a–h**

An appropriate amount of compound **2a–h** (1 equiv.) was dissolved in 1,4-dioxane, and DMF-DMA (2 equiv.) was added dropwise. The reaction was stirred at 100 °C for 2 h. After removal of the solvent in vacuo, the obtained residue was directly used in the next step without further purification.

#### 3.2.3. General Procedure for Compounds **4a–h**

To a solution of corresponding *β*-enamino keto ester **3a–h** (1 equiv.) in dry methanol, sodium acetate anhydrous (4.2 equiv.) and 2-methyl-2-thiopseudourea hemisulfate (1.4 equiv.) were added and stirred at r.t. for 16 h. The resulting mixture was concentrated under reduced pressure and purified by flash chromatography to provide products **4a–h**.

##### Methyl 4-[(3*R*)-1-(tert-butoxycarbonyl)piperidin-3-yl]-2-(methylsulfanyl)pyrimidine-5-carboxylate (**4a**)

The sample was prepared from **3a** (0.340 g, 1 mmol), sodium acetate anhydrous (0.345 g, 4.2 mmol) and 2-methyl-2-thiopseudourea hemisulfate (0.390 g, 1.4 mmol) dissolved in dry methanol. The reaction was stirred at r.t. for 16 h. The obtained residue was purified by column chromatography (SiO_2_, eluent:ethylacetate/*n*-hexane, 1:6, *v*/*v*) to provide compound **4a** as a colorless liquid. Yield 0.316 g (86%), [*α*]_D_^20^ = 84.1 (*c* 0.927, MeOH). ^1^H NMR (400 MHz, CDCl_3_): *δ*_H_ ppm 1.45 (s, 9H, C(CH_3_)_3_), 1.59–1.77 (m, 3H, Pip 4-H, Pip 5-CH_2_), 2.00–2.03 (m, 1H, Pip 4-H), 2.58 (s, 3H, S-CH_3_), 2.76 (t, *J* = 12.5 Hz, 1H, Pip 6-H), 3.13–3.19 (m, 1H, Pip 2-H), 3.67–3.73 (m, 1H, Pip 3-H), 3.91 (s, 3H, COOCH_3_), 4.13–4.19 (m, 2H, Pip 2,6-H), 8.91 (s, 1H, Pyr). ^13^C NMR (101 MHz, CDCl_3_): *δ*_C_ ppm 14.5 (S-CH_3_), 25.2 (Pip C-5), 28.6 (COOC(CH_3_)_3_), 30.8 (Pip C-4), 41.0 (Pip C-3), 44.2 (Pip C-6), 47.6 (Pip C-2), 52.6 (COOCH_3_), 79.6 (COOC(CH_3_)_3_), 117.9 (Pyr C-5), 154.9 (COOC(CH_3_)_3_), 159.3 (Pyr C-6), 165.2 (COOCH_3_), 172.6 (Pyr C-4), 175.7 (Pyr C-2). ^15^N-NMR (71 MHz, CDCl_3_): *δ*_N_ ppm −293.5 (*N*-Boc), −105.4 (Pyr 1-N), −101.7 (Pyr 3-N). IR (FT-IR, *ν*_max_, cm^−1^): 2930 (CH_aliph_), 1725 (C=O), 1688 (C=O), 1560, 1402, 1163 (C=C, C–N, C–O–C). HRMS (ESI^+^) C_17_H_26_N_3_O_4_S ([M+H]^+^) calcd. 368.1639, found 368.1640.

##### Methyl 4-[(3*S*)-1-(*tert*-butoxycarbonyl)piperidin-3-yl]-2-(methylsulfanyl)pyrimidine-5-carboxylate (**4b**)

The sample was prepared from **3b** (0.340 g, 1 mmol), sodium acetate anhydrous (0.345 g, 4.2 mmol) and 2-methyl-2-thiopseudourea hemisulfate (0.390 g, 1.4 mmol) dissolved in dry methanol. The reaction was stirred at r.t. for 16 h. The obtained residue was purified by column chromatography (SiO_2_, eluent:ethylacetate/*n*-hexane, 1:6, *v*/*v*) to provide compound **4b** as a colorless liquid. Yield 0.320 g (87%), [*α*]_D_^20^ = −84.2 (*c* 0.907, MeOH). ^1^H NMR (400 MHz, CDCl_3_): *δ*_H_ ppm 1.45 (s, 9H, C(CH_3_)_3_), 1.59–1.78 (m, 3H, Pip 4-H, Pip 5-CH_2_), 2.00–2.03 (m, 1H, 4-H), 2.59 (s, 3H, S-CH_3_), 2.73–2.79 (m, 1H, Pip 6-H), 3.14–3.20 (m, 1H, Pip 2-H), 3.65–3.73 (m, 1H, Pip 3-H), 3.92 (s, 3H, COOCH_3_), 4.08–4.24 (m, 2H, Pip 2,6-H), 8.92 (s, 1H, Pyr). ^13^C NMR (101 MHz, CDCl_3_): *δ*_C_ ppm 14.5 (S-CH_3_), 25.2 (Pip C-5), 28.6 (COOC(CH_3_)_3_), 30.8 (Pip C-4), 41.0 (Pip C-3), 44.3 (Pip C-6), 47.7 (Pip C-2), 52.5 (COOCH_3_), 79.6 (COOC(CH_3_)_3_), 117.9 (Pyr C-5), 154.9 (COOC(CH_3_)_3_), 159.4 (Pyr C-6), 165.2 (COOCH_3_), 172.6 (Pyr C-4), 175.7 (Pyr C-2). ^15^N-NMR (71 MHz, CDCl_3_): *δ*_N_ ppm −104.9 (Pyr 1-N), −101.8 (Pyr 3-N), *N*-Boc was not found. IR (FT-IR, *ν*_max_, cm^−1^): 2930 (CH_aliph_), 1724 (C=O), 1688 (C=O), 1560, 1402, 1162 (C=C, C–N, C–O–C). HRMS (ESI^+^) C_17_H_26_N_3_O_4_S ([M+H]^+^) calcd. 368.1639, found 368.1640.

##### Methyl 4-[(2*R*)-1-(*tert*-butoxycarbonyl)piperidin-2-yl]-2-(methylsulfanyl)pyrimidine-5-carboxylate (**4c**)

The sample was prepared from **3c** (0.340 g, 1 mmol), sodium acetate anhydrous (0.345 g, 4.2 mmol) and 2-methyl-2-thiopseudourea hemisulfate (0.390 g, 1.4 mmol) dissolved in dry methanol. The reaction was stirred at r.t. for 16 h. The obtained residue was purified by column chromatography (SiO_2_, eluent:ethylacetate/*n*-hexane, 1:6, *v*/*v*) to provide compound **4c** as a colorless liquid. Yield 0.125 g (34%), [*α*]_D_^20^ = 85.8 (*c* 0.391, MeOH). ^1^H NMR (400 MHz, CDCl_3_): *δ*_H_ ppm 1.15–1.40 (m, 10H, C(CH_3_)_3_, Pip 4-H), 1.48–1.58 (m, 2H, Pip 4,5-H), 1.72–1.80 (m, 1H, Pip 5-H), 1.87–1.90 (m, 1H, Pip 3-H), 1.98–2.06 (m, 1H, SH), 2.57 (s, 3H, S-CH_3_), 3.53–3.61 (m, 1H, Pip 6-H), 3.90 (s, 3H, COOCH_3_), 3.98–4.09 (m, 1H, Pip 6-H), 5.94 (s, 1H, Pip 2-H), 8.93 (s, 1H, Pyr). ^13^C NMR (101 MHz, CDCl_3_): *δ*_C_ ppm 14.8 (S-CH_3_), 19.0 (Pip C-4), 24.7 (Pip C-5), 28.3 (COOC(CH_3_)_3_), 29.1 (Pip C-3), 42.3 Pip C-6), 52.6 (COOCH_3_), 53.3 (Pip C-2), 79.6 (COOC(CH_3_)_3_), 116.8 (Pyr 5-C), 156.2 (COOC(CH_3_)_3_), 159.3 (Pyr 6-C), 165.1 (COOCH_3_), 174.0 (Pyr 2-C), 175.8 (Pyr 4-C). ^15^N-NMR (71 MHz, CDCl_3_): *δ*_N_ ppm −136.8 (Pyr 3-N), −105.1 (Pyr 1-N), *N*-Boc was not found. IR (FT-IR, *ν*_max_, cm^−1^): 2932 (CH_aliph_), 1724 (C=O), 1693 (C=O), 1562, 1402, 1159 (C=C, C–N, C–O–C). HRMS (ESI^+^) C_17_H_25_N_3_NaO_4_S ([M+Na]^+^) calcd. 390.1458, found 390.1459.

##### Methyl 4-[(2*S*)-1-(*tert*-butoxycarbonyl)piperidin-2-yl]-2-(methylsulfanyl)pyrimidine-5-carboxylate (**4d**)

The sample was prepared from **3d** (0.340 g, 1 mmol), sodium acetate anhydrous (0.345 g, 4.2 mmol) and 2-methyl-2-thiopseudourea hemisulfate (0.390 g, 1.4 mmol) dissolved in dry methanol. The reaction was stirred at r.t. for 16 h. The obtained residue was purified by column chromatography (SiO_2_, eluent:ethylacetate/*n*-hexane, 1:6, *v*/*v*) to provide compound **4d** as a colorless liquid. Yield 0.195 g (53%), [*α*]_D_^20^ = −85.7 (*c* 0.350, MeOH). ^1^H NMR (400 MHz, CDCl_3_): *δ*_H_ ppm 1.17–1.26 (m, 10H, C(CH_3_)_3_, Pip 4-H), 1.43–1.57 (m, 2H, Pip 4,5-H), 1.77 (s, 1H, Pip 5-H), 1.88–1.90 (m, 1H, Pip 3-H), 1.99–2.07 (m, 1H, SH), 2.57 (s, 3H, S-CH_3_), 3.54–3.61 (m, 1H, Pip 6-H), 3.91 (s, 3H, COOCH_3_), 3.98–4.09 (m, 1H, Pip 6-H), 5.94 (s, 1H, Pip 2-H), 8.93 (s, 1H, Pyr). ^13^C NMR (101 MHz, CDCl_3_): *δ*_C_ ppm 14.8 (S-CH_3_), 19.0 (Pip C-4), 24.7 (Pip C-5), 28.3 (COOC(CH_3_)_3_), 29.1 (Pip C-3), 42.9 (Pip C-6), 52.6 (COOCH_3_), 53.3 (Pip C-2), 79.7 (COOC(CH_3_)_3_), 116.9 (Pyr C-5), 156.2 (COOC(CH_3_)_3_), 159.3 (Pyr C-6), 165.0 (COOCH_3_), 174.1 (Pyr C-2), 175.6 (Pyr C-4). ^15^N-NMR (71 MHz, CDCl_3_): *δ*_N_ ppm −105.5 (Pyr 1-N), Pyr 3-N and *N*-Boc were not found. IR (FT-IR, *ν*_max_, cm^−1^): 2942 (CH_aliph_), 1719 (C=O), 1698 (C=O), 1559, 1402, 1155 (C=C, C–N, C–O–C). HRMS (ESI^+^) C_17_H_26_N_3_O_4_S ([M+H]^+^) calcd. 368.1639, found 368.1639.

##### *Tert*-butyl (2*R*)-2-[5-(methoxycarbonyl)-2-(methylsulfanyl)pyrimidin-4-yl]morpholine-4-carboxylate (**4e**)

The sample was prepared from **3e** (0.342 g, 1 mmol), sodium acetate anhydrous (0.345 g, 4.2 mmol) and 2-methyl-2-thiopseudourea hemisulfate (0.390 g, 1.4 mmol) dissolved in dry methanol. The reaction was stirred at r.t. for 16 h. The obtained residue was purified by column chromatography (SiO_2_, eluent:ethylacetate/*n*-hexane, 1:6, *v*/*v*) to provide compound **4e** as a colorless liquid. Yield 0.270 g (73%), [*α*]_D_^20^ = −80.8 (*c* 1.050, MeOH). ^1^H NMR (400 MHz, CDCl_3_): *δ*_H_ ppm 1.47 (s, 9H, C(CH_3_)_3_), 2.59 (s, 3H, S-CH_3_), 3.01–3.16 (m, 1H, Pip 5-H), 3.25–3.30 (m, 1H, Pip 5-H), 3.65–3.71 (m, 1H, Pip 6-H), 3.87–4.05 (m, 5H, COOCH_3_, Pip 5,6-H), 4.14–4.34 (m, 1H, Pip 3-H), 5.14–5.17 (m, 1H, Pip 2-H), 8.91 (s, 1H, Pyr). ^13^C NMR (101 MHz, CDCl_3_): *δ*_C_ ppm 14.5 (S-CH_3_), 28.5 (COOC(CH_3_)_3_), 43.5 (Pip C-5), 46.3 (Pip C-3), 52.7 (COOCH_3_), 67.2 (Pip C-6), 74.7 (Pip C-2), 80.3 (COOC(CH_3_)_3_), 118.6 (Pyr C-5), 154.7 (COOC(CH_3_)_3_), 159.1 (Pyr C-6), 165.1 (COOCH_3_), 165.7 (Pyr C-4), 176.1 (Pyr C-2). ^15^N-NMR (71 MHz, CDCl_3_): *δ*_N_ ppm −104.1 (Pyr 3-N), −101.3 (Pyr 1-N), *N*-Boc was not found. IR (FT-IR, *ν*_max_, cm^−1^): 2929 (CH_aliph_), 1728 (C=O), 1694 (C=O), 1564, 1411, 1168 (C=C, C–N, C–O–C). HRMS (ESI^+^) C_16_H_24_N_3_O_5_S ([M+H]^+^) calcd. 370.1431, found 370.1431.

##### *Tert*-butyl (2*S*)-2-[5-(methoxycarbonyl)-2-(methylsulfanyl)pyrimidin-4-yl]morpholine-4-carboxylate (**4f**)

The sample was prepared from **3f** (0.342 g, 1 mmol), sodium acetate anhydrous (0.345 g, 4.2 mmol) and 2-methyl-2-thiopseudourea hemisulfate (0.390 g, 1.4 mmol) dissolved in dry methanol. The reaction was stirred at r.t. for 16 h. The obtained residue was purified by column chromatography (SiO_2_, eluent:ethylacetate/*n*-hexane, 1:6, *v*/*v*) to provide compound **4f** as a colorless liquid. Yield 0.284 g (77%), [*α*]_D_^20^ = 80.8 (*c* 1.150, MeOH). ^1^H NMR (400 MHz, CDCl_3_): *δ*_H_ ppm 1.48 (s, 9H, C(CH_3_)_3_), 2.60 (s, 3H, S-CH_3_), 3.02–3.13 (m, 1H, Pip 5-H), 3.25–3.31 (m, 1H, Pip 5-H), 3.66–3.72 (m, 1H, Pip 6-H), 3.92–3.99 (m, 5H, COOCH_3_, Pip 5,6-H), 4.15–4.36 (m, 1H, Pip 3-H), 5.15–5.18 (m, 1H, Pip 2-H), 8.91 (s, 1H, Pyr). ^13^C NMR (101 MHz, CDCl_3_): *δ*_C_ ppm 14.5 (S-CH_3_), 28.5 (COOC(CH_3_)_3_), 43.5 (Pip C-5), 46.3 (Pip C-3), 52.7 (COOCH_3_), 67.2 (Pip C-6), 74.7 (Pip C-2), 80.3 (COOC(CH_3_)_3_), 118.6 (Pyr C-5), 154.8 (COOC(CH_3_)_3_), 159.1 (Pyr C-6), 165.2 (COOCH_3_), 165.7 (Pyr C-4), 176.1 (Pyr C-2). ^15^N-NMR (71 MHz, CDCl_3_): *δ*_N_ ppm −300.4 (*N*-Boc), −103.2 (Pyr 3-N), −100.7 (Pyr 1-N). IR (FT-IR, *ν*_max_, cm^−1^): 2928 (CH_aliph_), 1727 (C=O), 1692 (C=O), 1564, 1409, 1165 (C=C, C–N, C–O–C). HRMS (ESI^+^) C_16_H_23_N_3_NaO_5_S ([M+Na]^+^) calcd. 392.1251, found 392.1250.

##### Methyl 4-{*trans*-4-[(*tert*-butoxycarbonyl)amino]cyclohexyl}-2-(methylsulfanyl)pyrimidine-5-carboxylate (**4g**)

The sample was prepared from **3g** (0.354 g, 1 mmol), sodium acetate anhydrous (0.345 g, 4.2 mmol) and 2-methyl-2-thiopseudourea hemisulfate (0.390 g, 1.4 mmol) dissolved in dry methanol. The reaction was stirred at r.t. for 16 h. The obtained residue was purified by column chromatography (SiO_2_, eluent:ethylacetate/*n*-hexane, 1:6, *v*/*v*) to provide compound **4g** as a white amorphous compound. Yield 0.267 g (70%), [*α*]_D_^20^ = −3.0 (*c* 0.455, MeOH). ^1^H NMR (400 MHz, CDCl_3_): *δ*_H_ ppm 1.22–1.31 (m, 2H, Cy), 1.45 (s, 9H, C(CH_3_)_3_), 1.77–1.85 (m, 4H, Cy), 2.10–2.13 (m, 2H, Cy), 2.57 (s, 3H, S-CH_3_), 3.46–3.61 (m, 2H, Cy 1,4-H), 3.90 (s, 3H, COOCH_3_), 4.42 (s, 1H, NH), 8.88 (s, 1H, Pyr). ^13^C NMR (101 MHz, CDCl_3_): *δ*_C_ ppm 14.4 (S-CH_3_), 28.6 (COOC(CH_3_)_3_), 30.5 (Cy 2 × CH_2_), 33.4 (Cy 2 × CH_2_), 41.5 (Cy C-1), 49.5 (Cy C-4), 52.5 (COOCH_3_), 79.3 (COOC(CH_3_)_3_), 117.5 (Pyr C-5), 155.4 (COOC(CH_3_)_3_), 159.1 (Pyr C-6), 165.5 (COOCH_3_), 175.1 (Pyr C-4), 175.7 (Pyr C-2). ^15^N-NMR (71 MHz, CDCl_3_): *δ*_N_ ppm −282.9 (*N*-Boc), −106.1 (Pyr 1-N), −101.7 (Pyr 3-N). IR (FT-IR, *ν*_max_, cm^−1^): 3350 (N–H), 2969, 2935 (CH_aliph_), 1720 (C=O), 1674 (C=O), 1560, 1515, 1161 (C=C, C–N, C–O–C). HRMS (ESI^+^) C_18_H_27_N_3_NaO_4_S ([M+Na]^+^) calcd. 404.1614, found 404.1614.

##### Methyl 4-{*cis*-4-[(*tert*-butoxycarbonyl)amino]cyclohexyl}-2-(methylsulfanyl)pyrimidine-5-carboxylate (**4h**)

The sample was prepared from **3h** (0.354 g, 1 mmol), sodium acetate anhydrous (0.345 g, 4.2 mmol) and 2-methyl-2-thiopseudourea hemisulfate (0.390 g, 1.4 mmol) dissolved in dry methanol. The reaction was stirred at r.t. for 16 h. The obtained residue was purified by column chromatography (SiO_2_, eluent:ethylacetate/*n*-hexane, 1:6, *v*/*v*) to provide compound **4h** as a white amorphous compound. Yield 0.298 g (78%), [*α*]_D_^20^ = 3.1 (*c* 0.405, MeOH). ^1^H NMR (400 MHz, CDCl_3_): *δ*_H_ ppm 1.46 (s, 9H, C(CH_3_)_3_), 1.70–1.82 (m, 6H, Cy), 1.88–1.91 (m, 2H, Cy), 2.62 (s, 3H, S-CH_3_), 3.63–3.68 (m, 1H, Cy 1-H), 3.87–3.94 (m, 4H, COOCH_3_, Cy 4-H), 4.85 (s, 1H, NH), 8.91 (s, 1H, Pyr). ^13^C NMR (101 MHz, CDCl_3_): *δ*_C_ ppm 14.5 (S-CH_3_), 26.2 (Cy 2 × CH_2_), 28.6 (COOC(CH_3_)_3_), 30.2 (Cy 2 × CH_2_), 41.4 (Cy C-1), 45.1 (Cy C-4), 52.5 (COOCH_3_), 79.3 (COOC(CH_3_)_3_), 117.6 (Pyr C-5), 155.4 (COOC(CH_3_)_3_), 159.1 (Pyr C-6), 165.4 (COOCH_3_), 175.1 (Pyr C-4), 175.5 (Pyr C-2). ^15^N-NMR (71 MHz, CDCl_3_): *δ*_N_ ppm −290.6 (*N*-Boc), −106.8 (Pyr 1-N), −101.1 (Pyr 3-N). IR (FT-IR, *ν*_max_, cm^−1^): 3333 (N–H), 2932 (CH_aliph_), 1720 (C=O), 1675 (C=O), 1559, 1505, 1162 (C=C, C–N, C–O–C). HRMS (ESI^+^) C_18_H_27_N_3_NaO_4_S ([M+Na]^+^) calcd. 404.1614, found 404.1614.

#### 3.2.4. General Procedure for Compounds **4i–p**

The corresponding *β*-enamino keto ester **3a–h** (1 equiv.), sodium methoxide (2 equiv.) and amidine (1.5 equiv.) were dissolved in dry methanol, and the reaction mixture was stirred at room temperature for 16 h. The resulting solution was concentrated under reduced pressure and purified by flash chromatography to provide products **4i–p**.

##### Methyl 4-{*trans*-4-[(*tert*-butoxycarbonyl)amino]cyclohexyl}-2-methylpyrimidine-5-carboxylate (**4i**)

The sample was prepared from **3g** (0.354 g, 1 mmol), sodium methoxide (0.108 g, 2 mmol) and acetamidine hydrochloride (0.142 g, 1.5 mmol) dissolved in dry methanol. The reaction was stirred at r.t. for 16 h. The obtained residue was purified by column chromatography (SiO_2_, eluent:ethylacetate/*n*-hexane, 1:8, *v*/*v*) to provide compound **4i** as a white amorphous compound. Yield 0.209 g (60%), [*α*]_D_^20^ = −1.1 (*c* 0.736, MeOH). ^1^H NMR (400 MHz, CDCl_3_): *δ*_H_ ppm 1.20–1.31 (m, 2H, Cy), 1.45 (s, 9H, C(CH_3_)_3_), 1.82–1.84 (m, 4H, Cy), 2.11–2.14 (m, 2H, Cy), 2.73 (s, 3H, CH_3_), 3.48–3.56 (m, 2H, Cy 1,4-H), 3.92 (s, 3H, COOCH_3_), 4.41 (s, 1H, NH), 8.98 (s, 1H, Pyr). ^13^C NMR (101 MHz, CDCl_3_): *δ*_C_ ppm 26.4 (CH_3_), 28.6 (COOC(CH_3_)_3_), 30.5 (Cy 2 × CH_2_), 33.4 (Cy 2 × CH_2_), 41.5 (Cy C-1), 49.4 (Cy C-4), 52.6 (COOCH_3_), 79.2 (COOC(CH_3_)_3_), 119.9 (Pyr C-5), 155.3 (COOC(CH_3_)_3_), 158.7 (Pyr C-6), 165.7 (COOCH_3_), 170.4 (Pyr C-4), 175.0 (Pyr C-2). ^15^N-NMR (71 MHz, CDCl_3_): *δ*_N_ ppm −100.2 (Pyr 1-N), −90.8 (Pyr 3-N), *N*-Boc was not found. IR (FT-IR, *ν*_max_, cm^−1^): 3348 (N–H), 2930 (CH_aliph_), 1726 (C=O), 1674 (C=O), 1518, 1433, 1272, 1163 (C=C, C–N, C–O–C). HRMS (ESI^+^) C_18_H_27_N_3_NaO_4_ ([M+Na]^+^) calcd. 372.1894, found 372.1892.

##### Methyl 4-{*cis*-4-[(*tert*-butoxycarbonyl)amino]cyclohexyl}-2-methylpyrimidine-5-carboxylate (**4j**)

The sample was prepared from **3h** (0.354 g, 1 mmol), sodium methoxide (0.108 g, 2 mmol) and acetamidine hydrochloride (0.142 g, 1.5 mmol) dissolved in dry methanol. The reaction was stirred at r.t. for 16 h. The obtained residue was purified by column chromatography (SiO_2_, eluent:ethylacetate/*n*-hexane, 1:8, *v*/*v*) to provide compound **4j** as a white amorphous compound. Yield 0.202 g (58%), [*α*]_D_^20^ = 1.2 (*c* 0.750, MeOH). ^1^H NMR (400 MHz, CDCl_3_): *δ*_H_ ppm 1.45 (s, 9H, C(CH_3_)_3_), 1.66–1.91 (m, 8H, Cy 4 × CH_2_), 2.75 (s, 3H, CH_3_), 3.57–3.62 (m, 1H, Cy 1-H), 3.91–3.94 (m, 4H, COOCH_3_, Cy 4-H), 4.96 (s, 1H, NH), 8.98 (s, 1H, Pyr). ^13^C NMR (101 MHz, CDCl_3_): *δ*_C_ ppm 26.2 (Cy CH_2_), 26.4 (CH_3_), 28.6 (COOC(CH_3_)_3_), 30.3 (Cy 3 × CH_2_), 41.4 (Cy C-1), 45.1 (Cy C-4), 52.6 (COOCH_3_), 79.2 (COOC(CH_3_)_3_), 119.9 (Pyr C-5), 155.4 (COOC(CH_3_)_3_), 158.7 (Pyr C-6), 165.6 (COOCH_3_), 170.2 (Pyr C-2), 175.0 (Pyr C-4). ^15^N-NMR (71 MHz, CDCl_3_): *δ*_N_ ppm −99.2 (Pyr 1-N), −91.4 (Pyr 3-N), *N*-Boc was not found. IR (FT-IR, *ν*_max_, cm^−1^): 3338 (N–H), 2933 (CH_aliph_), 1727 (C=O), 1673 (C=O), 1520, 1260, 1165, 1093 (C=C, C–N, C–O–C). HRMS (ESI^+^) C_18_H_27_N_3_NaO_4_ ([M+Na]^+^) calcd. 372.1894, found 372.1894.

##### *Tert*-butyl (2*R*)-2-[5-(methoxycarbonyl)-2-methylpyrimidin-4-yl]morpholine-4-carboxylate (**4k**)

The sample was prepared from **3e** (0.342 g, 1 mmol), sodium methoxide (0.108 g, 2 mmol) and acetamidine hydrochloride (0.142 g, 1.5 mmol) dissolved in dry methanol. The reaction was stirred at r.t. for 16 h. The obtained residue was purified by column chromatography (SiO_2_, eluent:ethylacetate/*n*-hexane, 1:8, *v*/*v*) to provide compound **4k** as a colorless liquid. Yield 0.236 g (70%), [*α*]_D_^20^ = −53.1 (*c* 0.250, MeOH). ^1^H NMR (400 MHz, CDCl_3_): *δ*_H_ ppm 1.47 (s, 9H, C(CH_3_)_3_), 2.80 (s, 3H, CH_3_), 3.02–3.23 (m, 2H, Morph 3,5-H), 3.69 (t, *J* = 11.7 Hz, 1H, Morph 6-H), 3.88–4.05 (m, 5H, COOCH_3_, Morph 5,6-H), 4.16–4.34 (m, 1H, Morph 3-H), 5.17–5.19 (m, 1H, Morph 2-H), 9.03 (s, 1H, Pyr). ^13^C NMR (101 MHz, CDCl_3_): *δ*_C_ ppm 26.6 (CH_3_), 28.5 (COOC(CH_3_)_3_), 43.3 (Morph C-5), 47.0 (Morph C-3), 52.8 (COOCH_3_), 67.3 (Morph C-6), 75.2 (Morph C-2), 80.3 (COOC(CH_3_)_3_), 120.6 (Pyr C-5), 154.7 (COOC(CH_3_)_3_), 158.9 (Pyr C-6), 165.1 (COOCH_3_), 165.8 (Pyr C-4), 170.9 (Pyr C-2). ^15^N-NMR (71 MHz, CDCl_3_): *δ*_N_ ppm −298.6 (*N*-Boc), −93.5 (Pyr 1-N), Pyr 3-N was not found. IR (FT-IR, *ν*_max_, cm^−1^): 2972, 2929, 2861 (CH_aliph_), 1731 (C=O), 1694 (C=O), 1574, 1415, 1250, 1165, 1100 (C=C, C–N, C–O–C). HRMS (ESI^+^) C_16_H_24_N_3_O_5_ ([M+H]^+^) calcd. 338.1710, found 338.1710.

##### *Tert*-butyl (2*S*)-2-[5-(methoxycarbonyl)-2-methylpyrimidin-4-yl]morpholine-4-carboxylate (**4l**)

The sample was prepared from **3f** (0.342 g, 1 mmol), sodium methoxide (0.108 g, 2 mmol) and acetamidine hydrochloride (0.142 g, 1.5 mmol) dissolved in dry methanol. The reaction was stirred at r.t. for 16 h. The obtained residue was purified by column chromatography (SiO_2_, eluent:ethylacetate/*n*-hexane, 1:8, *v*/*v*) to provide compound **4l** as a white amorphous compound. Yield 0.212 g (63%), [*α*]_D_^20^ = 53,2 (*c* 0.245, MeOH). ^1^H NMR (400 MHz, CDCl_3_): *δ*_H_ ppm 1.48 (s, 9H, C(CH_3_)_3_), 2.81 (s, 3H, CH_3_), 3.05–3.22 (m, 2H, Morph 3,5-H), 3.70 (t, *J* = 11.7 Hz, 1H, Morph 6-H), 3.84–4.06 (m, 5H, COOCH_3_, Morph 5,6-H), 4.18–4.36 (m, 1H, Morp 3-H), 5.18–5.20 (m, 1H, Morph 2-H), 9.04 (s, 1H, Pyr). ^13^C NMR (101 MHz, CDCl_3_): *δ*_C_ ppm 26.5 (CH_3_), 28.5 (COOC(CH_3_)_3_), 43.3 (Morph C-5), 47.1 (Morph C-3), 52.9 (COOCH_3_), 67.3 (Morph C-6), 75.2 (Morph C-2), 80.3 (COOC(CH_3_)_3_), 120.7 (Pyr C-5), 154.7 (COOC(CH_3_)_3_), 158.7 (Pyr C-6), 165.1 (COOCH_3_), 166.0 (Pyr C-4), 170.8 (Pyr C-2). ^15^N-NMR (71 MHz, CDCl_3_): *δ*_N_ ppm −299.5 (*N*-Boc), −93.5 (Pyr 1-N), Pyr 3-N was not found. IR (FT-IR, *ν*_max_, cm^−1^): 2966, 2920, 2865 (CH_aliph_), 1724 (C=O), 1684 (C=O), 1572, 1423, 1274, 1166, 1104 (C=C, C–N, C–O–C). HRMS (ESI^+^) C_16_H_24_N_3_O_5_ ([M+H]^+^) calcd. 338.1710, found 338.1710.

##### Methyl 4-{*trans*-4-[(*tert*-butoxycarbonyl)amino]cyclohexyl}-2-cyclopropylpyrimidine-5-carboxylate (**4m**)

The sample was prepared from **3g** (0.354 g, 1 mmol), sodium methoxide (0.108 g, 2 mmol) and cyclopropanecarboximidamide hydrochloride (0.181 g, 1.5 mmol) dissolved in dry methanol. The reaction was stirred at r.t. for 16 h. The obtained residue was purified by column chromatography (SiO_2_, eluent:ethylacetate/*n*-hexane, 1:8, *v*/*v*) to provide compound **4m** as a white amorphous compound. Yield 0.150 g (40%), [*α*]_D_^20^ = −15.5 (*c* 0.536, MeOH). ^1^H NMR (400 MHz, CDCl_3_): *δ*_H_ ppm 1.13–1.30 (m, 6H, Cpr 2 × CH_2_, Cy H), 1.45 (s, 9H, C(CH_3_)_3_), 1.75–1.84 (m, 4H, Cy H), 2.08–2.12 (m, 2H, Cy H), 2.25–2.31 (m, 1H, Cpr CH), 3.49–3.57 (m, 2H, Cy 1,4-H), 3.90 (s, 3H, COOCH_3_), 4.43 (s, 1H, NH), 8.92 (s, 1H, Pyr). ^13^C NMR (101 MHz, CDCl_3_): *δ*_C_ ppm 12.3 (Cpr 2 × CH_2_), 18.6 (Cpr CH), 28.6 (COOC(CH_3_)_3_), 30.4 (Cy 2 × CH_2_), 33.4 (Cy 2 × CH_2_), 41.4 (Cy C-1), 49.5 (Cy C-4), 52.5 (COOCH_3_), 79.3 (COOC(CH_3_)_3_), 119.3 (Pyr C-5), 155.4 (COOC(CH_3_)_3_), 158.3 (Pyr C-6), 165.6 (COOCH_3_), 174.2 (Pyr C-4), 175.1 (Pyr C-2). ^15^N-NMR (71 MHz, CDCl_3_): *δ*_N_ ppm −282.7 (*N*-Boc), −111.1 (Pyr 1-N), −102.0 (Pyr 3-N). IR (FT-IR, *ν*_max_, cm^−1^): 3356 (N–H), 2934 (CH_aliph_), 1724 (C=O), 1674 (C=O), 1512, 1453, 1265, 1163 (C=C, C–N, C–O–C). HRMS (ESI^+^) C_20_H_29_N_3_NaO_4_ ([M+Na]^+^) calcd. 398.2050, found 398.2051.

##### Methyl 4-{*cis*-4-[(*tert*-butoxycarbonyl)amino]cyclohexyl}-2-cyclopropylpyrimidine-5-carboxylate (**4n**)

The sample was prepared from **3h** (0.354 g, 1 mmol), sodium methoxide (0.108 g, 2 mmol) and cyclopropanecarboximidamide hydrochloride (0.181 g, 1.5 mmol) dissolved in dry methanol. The reaction was stirred at r.t. for 16 h. The obtained residue was purified by column chromatography (SiO_2_, eluent:ethylacetate/*n*-hexane, 1:8, *v*/*v*) to provide compound **4n** as a white amorphous compound. Yield 0.173 g (46%), [*α*]_D_^20^ = 15.4 (*c* 0.525, MeOH). ^1^H NMR (400 MHz, CDCl_3_): *δ*_H_ ppm 1.14–1.25 (m, 5H, Cpr 2 × CH_2_, Cy CH), 1.47 (s, 9H, C(CH_3_)_3_), 1.67–1.90 (m, 8H, Cy 4 × CH_2_), 2.29–2.34 (m, 1H, Cpr CH), 3.59–3.64 (m, 1H, Cy 1-H), 3.91 (s, 3H, COOCH_3_), 4.89 (s, 1H, NH), 8.93 (s, 1H, Pyr). ^13^C NMR (101 MHz, CDCl_3_): *δ*_C_ ppm 12.2 (Cpr 2 × CH_2_), 18.8 (Cpr CH), 26.3 (Cy 2 × CH_2_), 28.6 (COOC(CH_3_)_3_), 30.3 (Cy 2 × CH_2_), 41.1 (Cy C-1), 45.3 (Cy C-4), 52.5 (COOCH_3_), 79.3 (COOC(CH_3_)_3_), 119.3 (Pyr C-5), 155.4 (COOC(CH_3_)_3_), 158.7 (Pyr C-6), 165.7 (COOCH_3_), 174.2 (Pyr C-4), 174.7 (Pyr C-2). ^15^N-NMR (71 MHz, CDCl_3_): *δ*_N_ ppm −109.9 (Pyr 1-N), Pyr 3-N and *N*-Boc were not found. IR (FT-IR, *ν*_max_, cm^−1^): 3266 (N–H), 2938 (CH_aliph_), 1722 (C=O), 1699 (C=O), 1532, 1282, 1165 (C=C, C–N, C–O–C). HRMS (ESI^+^) C_20_H_30_N_3_O_4_ ([M+H]^+^) calcd. 376.2231, found 376.2231.

##### *Tert*-butyl (2*R*)-2-[2-cyclopropyl-5-(methoxycarbonyl)pyrimidin-4-yl]morpholine-4-carboxylate (**4o**)

The sample was prepared from **3e** (0.342 g, 1 mmol), sodium methoxide (0.108 g, 2 mmol) and cyclopropanecarboximidamide hydrochloride (0.181 g, 1.5 mmol) dissolved in dry methanol. The reaction was stirred at r.t. for 16 h. The obtained residue was purified by column chromatography (SiO_2_, eluent:ethylacetate/*n*-hexane, 1:8, *v*/*v*) to provide compound **4o** as a white amorphous compound. Yield 0.258 g (71%), [*α*]_D_^20^ = −45.3 (*c* 0.450, MeOH). ^1^H NMR (400 MHz, CDCl_3_): *δ*_H_ ppm 1.13–1.24 (m, 4H, Cpr 2 × CH_2_), 1.47 (s, 9H, C(CH_3_)_3_), 2.32–2.38 (m, 1H, Cpr CH), 3.02–3.10 (m, 1H, Morph 5-H), 3.20–3.26 (m, 1H, Morph 3-H), 3.65–3.70 (m, 1H, Morph 6-H), 3.86–4.04 (m, 5H, COOCH_3_, Morph 5,6-H), 4.14–4.34 (m, 1H, Morp 3-H), 5.12–5.15 (m, 1H, Morph 2-H), 8.93 (s, 1H, Pyr). ^13^C NMR (101 MHz, CDCl_3_): *δ*_C_ ppm 12.5 (Cpr 2 × CH_2_), 18.9 (Cpr CH), 28.5 (COOC(CH_3_)_3_), 43.4 (Morph C-5), 46.5 (Morph C-3), 52.7 (COOCH_3_), 67.2 (Morph C-6), 74.8 (Morph C-2), 80.3 (COOC(CH_3_)_3_), 120.3 (Pyr C-5), 154.7 (COOC(CH_3_)_3_), 158.7 (Pyr C-6), 165.3 (COOCH_3_), 165.5 (Pyr C-4), 174.7 (Pyr C-2). ^15^N-NMR (71 MHz, CDCl_3_): *δ*_N_ ppm −299.5 (*N*-Boc), −104.1 (Pyr 1-N), −102.4 (Pyr 3-N). IR (FT-IR, *ν*_max_, cm^−1^): 2978, 2859 (CH_aliph_), 1727 (C=O), 1685 (C=O), 1576, 1431, 1266, 1166, 1101 (C=C, C–N, C–O–C). HRMS (ESI^+^) C_18_H_26_N_3_O_5_ ([M+H]^+^) calcd. 364.1867, found 364.1866.

##### *Tert*-butyl (2*S*)-2-[2-cyclopropyl-5-(methoxycarbonyl)pyrimidin-4-yl]morpholine-4-carboxylate (**4p**)

The sample was prepared from **3f** (0.342 g, 1 mmol), sodium methoxide (0.108 g, 2 mmol) and cyclopropanecarboximidamide hydrochloride (0.181 g, 1.5 mmol) dissolved in dry methanol. The reaction was stirred at r.t. for 16 h. The obtained residue was purified by column chromatography (SiO_2_, eluent:ethylacetate/*n*-hexane, 1:8, *v*/*v*) to provide compound **4p** as a white amorphous compound. Yield 0.320 g (88%), [*α*]_D_^20^ = 45.4 (*c* 0.445, MeOH). ^1^H NMR (400 MHz, CDCl_3_): *δ*_H_ ppm 1.13–1.24 (m, 4H, Cpr 2 × CH_2_), 1.48 (s, 9H, C(CH_3_)_3_), 2.31–2.36 (m, 1H, Cpr CH), 3.05–3.11 (m, 1H, Morph 5-H), 3.20–3.28 (m, 1H, Morph 3-H), 3.69 (t, *J* = 11.7 Hz, 1H, Morph 6-H), 3.87–4.05 (m, 5H, COOCH_3_, Morph 5,6-H), 4.13–4.33 (m, 1H, Morph 3-H), 5.13–5.15 (m, 1H, Morph 2-H), 8.94 (s, 1H, Pyr). ^13^C NMR (101 MHz, CDCl_3_): *δ*_C_ ppm 12.4 (Cpr CH_2_), 12.5 (Cpr CH_2_), 19.0 (Cpr CH), 28.5 (COOC(CH_3_)_3_), 43.5 (Morph C-5), 46.5 (Morph C-3), 52.7 (COOCH_3_), 67.2 (Morph C-6), 74.9 (Morph C-2), 80.3 (COOC(CH_3_)_3_), 120.3 (Pyr C-5), 154.8 (COOC(CH_3_)_3_), 158.9 (Pyr C-6), 165.3 (COOCH_3_), 165.4 (Pyr C-4), 174.8 (Pyr C-2). ^15^N-NMR (71 MHz, CDCl_3_): *δ*_N_ ppm −299.5 (*N*-Boc), −103.0 (Pyr 1-N), −102.4 (Pyr 3-N). IR (FT-IR, *ν*_max_, cm^−1^): 2978, 2919, 2859 (CH_aliph_), 1728 (C=O), 1686 (C=O), 1575, 1430, 1265, 1165, 1100 (C=C, C–N, C–O–C). HRMS (ESI^+^) C_18_H_25_N_3_NaO_5_ ([M+Na]^+^) calcd. 386.1686, found 386.1686.

#### 3.2.5. General Procedure for the Preparation of Compounds **6a–d**

**Method A.** The corresponding *β*-enamino keto ester **3a–d** (1 equiv.), sodium methoxide (2 equiv.) and 2-chloroacetimidamide hydrochloride (1.5 equiv.) were dissolved in dry methanol, and the reaction mixture was stirred at room temperature for 16 h. The resulting solution was concentrated under reduced pressure and purified by flash chromatography to provide products **6a–d**.

**Method B.** The corresponding *β*-keto ester **2a–d** (1 equiv.) was dissolved in ethanoic anhydride (7.2 equiv.) and trimethyl orthoformate (7.2 equiv.) were added. The reaction mixture was stirred at 110 °C for 4 h. After removal of the solvent in vacuo, the obtained residue **5a–d** (1 equiv.) and 2-chloroacetimidamide hydrochloride (1.5 equiv.) were dissolved in dry methanol, then triethylamine (2 equiv.) was added dropwise. The reaction mixture was stirred at r.t. for 2 h. The resulting solution was concentrated under reduced pressure and purified by flash chromatography to provide products **6a–d**.

##### Methyl 4-[(3*R*)-1-(*tert*-butoxycarbonyl)piperidin-3-yl]-2-(chloromethyl)pyrimidine-5-carboxylate (**6a**)

The sample was prepared from **5a** (0.327 g, 1 mmol) and 2-chloroacetimidamide hydrochloride (0.194 g, 1.5 mmol) dissolved in dry methanol. Then triethylamine (0.28 mL, 2 mmol) was added dropwise and the reaction mixture was stirred at r.t. for 2 h. The obtained residue was purified by column chromatography (SiO_2_, eluent:ethylacetate/*n*-hexane, 1:6, *v*/*v*) to provide compound **6a** as a colorless liquid. Yield 0.317 g (86%), [*α*]_D_^20^ = −49.4 (*c* 0.664, MeOH). ^1^H NMR (400 MHz, CDCl_3_): *δ*_H_ ppm 1.44 (s, 9H, C(CH_3_)_3_), 1.59–1.65 (m, 1H, Pip 5-H), 1.71–1.77 (m, 2H, Pip 4,5-H), 1.96–2.02 (m, 1H, Pip 4-H), 2.78 (t, *J* = 11.5 Hz, 1H, Pip 6-H), 3.15–3.21 (m, 1H, Pip 2-H), 3.64–3.70 (m, 1H, Pip 3-H), 3.95 (s, 3H, COOCH_3_), 4.07–4.22 (m, 2H, Pip 2,6-H), 4.71 (s, 2H, CH_2_-Cl), 9.11 (s, 1H, Pyr). ^13^C NMR (101 MHz, CDCl_3_): *δ*_C_ ppm 25.1 (Pip C-5), 28.6 (COOC(CH_3_)_3_), 30.7 (Pip C-4), 41.0 (Pip C-3), 44.3 (Pip C-6), 46.7 (CH_2_-Cl), 47.6 (Pip C-2), 52.9 (COOCH_3_), 79.6 (COOC(CH_3_)_3_), 121.9 (Pyr C-5), 154.8 (COOC(CH_3_)_3_), 159.7 (Pyr C-6), 164.9 (COOCH_3_), 167.4 (Pyr C-2), 173.1 (Pyr C-4). ^15^N NMR (71 MHz, CDCl_3_): *δ*_N_ ppm −96.6 (Pyr 1-N), −90.7 (Pyr 3-N), *N*-Boc was not found. IR (FT-IR, *ν*_max_, cm^−1^): 2936 (CH_aliph_), 1730 (C=O), 1688 (C=O), 1570, 1419, 1167 (C=C, C–N, C–O–C). HRMS (ESI^+^) C_17_H_25_ClN_3_O_4_ ([M+H]^+^) calcd. 370.1528, found 370.1528.

##### Methyl 4-[(3*S*)-1-(*tert*-butoxycarbonyl)piperidin-3-yl]-2-(chloromethyl)pyrimidine-5-carboxylate (**6b**)

The sample was prepared from **5b** (0.327 g, 1 mmol) and 2-chloroacetimidamide hydrochloride (0.194 g, 1.5 mmol) dissolved in dry methanol. Then triethylamine (0.28 mL, 2 mmol) was added dropwise and the reaction mixture was stirred at r.t. for 2 h. The obtained residue was purified by column chromatography (SiO_2_, eluent:ethylacetate/*n*-hexane, 1:6, *v*/*v*) to provide compound **6b** as a colorless liquid. Yield 0.273 g (74%), [*α*]_D_^20^ = 49.5 (*c* 0.650, MeOH). ^1^H NMR (400 MHz, CDCl_3_): *δ*_H_ ppm 1.45 (s, 9H, C(CH_3_)_3_), 1.60–1.66 (m, 1H, Pip 5-H), 1.72–1.80 (m, 2H, Pip 4,5-H), 2.01–2.03 (m, 1H, Pip 4-H), 2.79 (t, *J* = 12.7 Hz, 1H, Pip 6-H), 3.16–3.22 (m, 1H, Pip 2-H), 3.66–3.70 (m, 1H, Pip 3-H), 3.96 (s, 3H, COOCH_3_), 4.15–4.18 (m, 2H, Pip 2,6-H), 4.73 (s, 2H, CH_2_-Cl), 9.12 (s, 1H, Pyr). ^13^C NMR (101 MHz, CDCl_3_): *δ*_C_ ppm 25.1 (Pip C-5), 28.6 (COOC(CH_3_)_3_), 30.7 (Pip C-4), 41.0 (Pip C-3), 44.2 (Pip C-6), 46.7 (CH_2_-Cl), 47.7 (Pip C-2), 52.9 (COOCH_3_), 79.7 (COOC(CH_3_)_3_), 122.0 (Pyr C-5), 154.9 (COOC(CH_3_)_3_), 159.7 (Pyr C-6), 164.9 (COOCH_3_), 167.4 (Pyr C-2), 173.2 (Pyr C-4). ^15^N-NMR (71 MHz, CDCl_3_): *δ*_N_ ppm −97.2 (Pyr 1-N), −90.8 (Pyr 3-N), *N*-Boc was not found. IR (FT-IR, *ν*_max_, cm^−1^): 2935 (CH_aliph_), 1730 (C=O), 1688 (C=O), 1570, 1419, 1167 (C=C, C–N, C–O–C). HRMS (ESI^+^) C_17_H_24_ClN_3_NaO_4_ ([M+Na]^+^) calcd. 392.1348, found 392.1349.

##### Methyl 4-[(2*R*)-1-(*tert*-butoxycarbonyl)piperidin-2-yl]-2-(chloromethyl)pyrimidine-5-carboxylate (**6c**)

The sample was prepared from **5c** (0.327 g, 1 mmol) and 2-chloroacetimidamide hydrochloride (0.194 g, 1.5 mmol) dissolved in dry methanol. Then triethylamine (0.28 mL, 2 mmol) was added dropwise and the reaction mixture was stirred at r.t. for 2 h. The obtained residue was purified by column chromatography (SiO_2_, eluent:ethylacetate/*n*-hexane, 1:6, *v*/*v*) to provide compound **6c** as a yellowish oil. Yield 0.210 g (57%), [*α*]_D_^20^ = 26.6 (*c* 0.491, MeOH). ^1^H NMR (400 MHz, CDCl_3_): *δ*_H_ ppm 1.22–1.41 (m, 10H, C(CH_3_)_3_, Pip 4-H), 1.52–1.54 (m, 2H, Pip 4,5-H), 1.77 (s, 1H, Pip 5-H), 1.89–1.91 (m, 1H, Pip 3-H), 1.98–2.05 (m, 1H, Pip 3-H), 3.64–3.71 (m, 1H, Pip 6-H), 3.94 (s, 3H, COOCH_3_), 3.99–4.02 (m, 1H, Pip 6-H), 4.70 (s, 2H, CH_2_-Cl), 5.94 (s, 1H, Pip 2-H), 9.12 (s, 1H, Pyr). ^13^C NMR (101 MHz, CDCl_3_): *δ*_C_ ppm 18.7 (Pip C-4), 24.7 (Pip C-5), 28.3 (COOC(CH_3_)_3_), 29.0 (Pip C-3), 42.7 (Pip C-6), 46.8 (CH_2_-Cl), 52.9 (COOCH_3_), 53.1 (Pip C-2), 79.7 (COOC(CH_3_)_3_), 120.9 (Pyr C-5), 156.1 (COOC(CH_3_)_3_), 159.8 (Pyr C-6), 164.7 (COOCH_3_), 166.9 (Pyr C-2), 174.5 (Pyr C-4). ^15^N-NMR (71 MHz, CDCl_3_): *δ*_N_ ppm −96.9 (Pyr 1-N), −92.0 (Pyr 3-N), *N*-Boc was not found. IR (FT-IR, *ν*_max_, cm^−1^): 2940 (CH_aliph_), 1729 (C=O), 1689 (C=O), 1572, 1363, 1273, 1159 (C=C, C–N, C–O–C). HRMS (ESI^+^) C_17_H_25_ClN_3_O_4_ ([M+H]^+^) calcd. 370.1528, found 370.1528.

##### Methyl 4-[(2*S*)-1-(*tert*-butoxycarbonyl)piperidin-2-yl]-2-(chloromethyl)pyrimidine-5-carboxylate (**6d**)

The sample was prepared from **5d** (0.327 g, 1 mmol) and 2-chloroacetimidamide hydrochloride (0.194 g, 1.5 mmol) dissolved in dry methanol. Then triethylamine (0.28 mL, 2 mmol) was added dropwise and the reaction mixture was stirred at r.t. for 2 h. The obtained residue was purified by column chromatography (SiO_2_, eluent:ethylacetate/*n*-hexane, 1:6, *v*/*v*) to provide compound **6d** as a yellowish oil. Yield 0.199 g (54%), [*α*]_D_^20^ = −26.6 (*c* 0.515, MeOH). ^1^H NMR (400 MHz, CDCl_3_): *δ*_H_ ppm 1.24–1.27 (m, 10H, C(CH_3_)_3_, Pip 4-H), 1.52–1.54 (m, 2H, Pip 4,5-H), 1.77 (s, 1H, Pip 5-H), 1.89–1.91 (m, 1H, Pip 3-H), 1.97–2.05 (m, 1H, Pip 3-H), 3.64–3.71 (m, 1H, Pip 6-H), 3.94 (s, 3H, COOCH_3_), 3.98–4.01 (m, 1H, Pip 6-H), 4.70 (s, 2H, CH_2_-Cl), 5.94 (s, 1H, Pip 2-H), 9.12 (s, 1H, Pyr). ^13^C NMR (101 MHz, CDCl_3_): *δ*_C_ ppm 18.8 (Pip C-4), 24.7 (Pip C-5), 28.3 (COOC(CH_3_)_3_), 29.0 (Pip C-3), 42.7 (Pip C-6), 46.8 (CH_2_-Cl), 52.9 (COOCH_3_), 53.1 (Pip C-2), 79.7 (COOC(CH_3_)_3_), 120.9 (Pyr C-5), 156.0 (COOC(CH_3_)_3_), 159.8 (Pyr C-6), 164.7 (COOCH_3_), 166.9 (Pyr C-2), 174.5 (Pyr C-4). ^15^N-NMR (71 MHz, CDCl_3_): *δ*_N_ ppm −96.8 (Pyr 1-N), −91.9 (Pyr 3-N), *N*-Boc was not found. IR (FT-IR, *ν*_max_, cm^−1^): 2938 (CH_aliph_), 1729 (C=O), 1690 (C=O), 1571, 1363, 1273, 1159 (C=C, C–N, C–O–C). HRMS (ESI^+^) C_17_H_24_ClN_3_NaO_4_ ([M+Na]^+^) calcd. 392.1348, found 392.1355.

#### 3.2.6. General Procedure for Compounds **7a,b**

An appropriate amount of *β*-keto ester **2a,b** (1 equiv.) was dissolved in methylene chloride and cooled to 0 °C, then pyridine (2 equiv.) was added. Maintaining the temperature at 0 °C, MgCl_2_ (1 equiv.) was added in portions and stirred for 20 min. After 20 min. acetyl chloride (1.2 equiv.) was dissolved in methylene chloride, added in portion and the reaction was stirred at r.t. for 1 h. The resulting solution was quenched with saturated aq. NH_4_Cl solution (15 mL) and extracted with diethyl ether (2 × 10 mL). The organic layer was dried with anhydrous sodium sulfate, filtered, and then concentrated under reduced pressure. The obtained residue was directly used in the next step without further purification.

#### 3.2.7. General Procedure for Compounds **8a,b**

The obtained acetoacetate **7a,b** (1 equiv.) and cesium carbonate (1.1 equiv.) were dissolved in acetonitrile and cooled to 0 °C. Methyl trifluoromethanesulfonate was added dropwise, and the reaction was stirred at r.t. for 1 h. The resulting solution was quenched with water (10 mL) and extracted with ethylacetate (2 × 15 mL) and brine. The organic layer was dried with anhydrous sodium sulfate, filtered, and then concentrated under reduced pressure. The obtained residue was directly used in the next step without further purification.

#### 3.2.8. General Procedure for Compounds **9a,b**

Diketoester **8a,b** (1 equiv.), sodium methoxide (2 equiv.) and 2-chloroacetimidamide hydrochloride (1.5 equiv.) were dissolved in dry methanol, and the reaction was stirred at r.t. for 16 h. The resulting solution was concentrated under reduced pressure and purified by flash chromatography to provide products **9a,b**.

##### Methyl 4-[(3*R*)-1-(*tert*-butoxycarbonyl)piperidin-3-yl]-2-(chloromethyl)-6-methylpyrimidine-5-carboxylate (**9a**)

The obtained diketoester **8a** (0.341 g, 1 mmol), sodium methoxide (0.108 g, 2 equiv.) and 2-chloroacetimidamide hydrochloride (0.194 g, 1.5 mmol) were dissolved in dry methanol, and the reaction was stirred at r.t. for 16 h. The obtained residue was purified by column chromatography (SiO_2_, eluent:ethylacetate/*n*-hexane, 1:6, *v*/*v*) to provide compound **9a** as a colorless liquid. Yield 0.253 g (66%), [*α*]_D_^20^ = −21.5 (*c* 0.718, MeOH). ^1^H NMR (400 MHz, CDCl_3_): *δ*_H_ ppm 1.45–1.61 (m, 10H, C(CH_3_)_3_, Pip 5-H), 1.73–1.92 (m, 3H, Pip 4,5-H), 2.53 (s, 3H, Pyr 6-CH_3_), 2.69–2.82 (m, 2H, Pip 3,6-H), 3.04–3.10 (m, 1H, Pip 2-H), 3.98 (s, 3H, COOCH_3_), 4.16 (s, 2H, Pip 2,6-H), 4.65 (s, 2H, CH_2_-Cl). ^13^C NMR (101 MHz, CDCl_3_): *δ*_C_ ppm 22.9 (Pyr C6-CH_3_), 25.0 (Pip C-5), 28.6 (COOC(CH_3_)_3_), 30.1 (Pip C-4), 42.1 (Pip C-3), 44.0 (Pip C-6), 46.9 (CH_2_-Cl), 48.1 (Pip C-2), 53.0 (COOCH_3_), 79.7 (COOC(CH_3_)_3_), 125.2 (Pyr C-6), 154.8 (COOC(CH_3_)_3_), 165.2 (Pyr C-5), 165.4 (Pyr C-2), 167.7 (COOCH_3_), 168.6 (Pyr C-4). ^15^N-NMR (71 MHz, CDCl_3_): *δ*_N_ ppm −98.6 (Pyr 3-N), −94.8 (Pyr 1-N), *N*-Boc was not found. IR (FT-IR, *ν*_max_, cm^−1^): 2940 (CH_aliph_), 1731 (C=O), 1690 (C=O), 1551, 1419, 1243, 1146, 1088 (C=C, C–N, C–O–C). HRMS (ESI^+^) C_18_H_26_ClN_3_NaO_4_ ([M+Na]^+^) calcd. 406.1504, found 406.1506.

##### Methyl 4-[(3*S*)-1-(*tert*-butoxycarbonyl)piperidin-3-yl]-2-(chloromethyl)-6-methylpyrimidine-5-carboxylate (**9b**)

The obtained diketoester **8b** (0.341 g, 1 mmol), sodium methoxide (0.108 g, 2 equiv.) and 2-chloroacetimidamide hydrochloride (0.194 g, 1.5 mmol) were dissolved in dry methanol, and the reaction was stirred at r.t. for 16 h. The obtained residue was purified by column chromatography (SiO_2_, eluent:ethylacetate/*n*-hexane, 1:6, *v*/*v*) to provide compound **9b** as a colorless liquid. Yield 0.199 g (52%), [*α*]_D_^20^ = 21.4 (*c* 0.650, MeOH). ^1^H NMR (400 MHz, CDCl_3_): *δ*_H_ ppm 1.44–1.54 (m, 10H, C(CH_3_)_3_, Pip 5-H), 1.73–1.91 (m, 3H, Pip 4,5-H), 2.53 (s, 3H, Pyr 6-CH_3_), 2.69–2.82 (m, 2H, Pip 3,6-H), 3.04–3.10 (m, 1H, Pip 2-H), 3.97 (s, 3H, COOCH_3_), 4.15 (s, 2H, Pip 2,6-H), 4.65 (s, 2H, CH_2_-Cl). ^13^C NMR (101 MHz, CDCl_3_): *δ*_C_ ppm 22.9 (Pyr C6-CH_3_), 25.0 (Pip C-5), 28.6 (COOC(CH_3_)_3_), 30.1 (Pip C-4), 42.1 (Pip C-3), 44.1 (Pip C-6), 46.9 (CH_2_-Cl), 48.1 (Pip C-2), 53.0 (COOCH_3_), 79.7 (COOC(CH_3_)_3_), 125.2 (Pyr C-6), 154.8 (COOC(CH_3_)_3_), 165.2 (Pyr C-5), 165.3 (Pyr C-2), 167.7 (COOCH_3_), 168.6 (Pyr C-4). ^15^N-NMR (71 MHz, CDCl_3_): *δ*_N_ ppm −98.8 (Pyr 3-N), −94.8 (Pyr 1-N), *N*-Boc was not found. IR (FT-IR, *ν*_max_, cm^−1^): 2932 (CH_aliph_), 1731 (C=O), 1690 (C=O), 1552, 1420, 1243, 1146, 1088 (C=C, C–N, C–O–C). HRMS (ESI^+^) C_18_H_27_ClN_3_O_4_ ([M+H]^+^) calcd. 384.1685, found 384.1685.

#### 3.2.9. General Procedure for Compounds **9c,d**

An appropriate compound **8a,b** (1 equiv.), sodium acetate anhydrous (4.2 equiv.) and 2-methyl-2-thiopseudourea hemisulfate (1.4 equiv.) were dissolved in dry methanol and stirred at r.t. for 16 h. The resulting solution was concentrated under reduced pressure and purified by flash chromatography to provide products **9c,d**.

##### Methyl 4-[(3*R*)-1-(*tert*-butoxycarbonyl)piperidin-3-yl]-6-methyl-2-(methylsulfanyl)pyrimidine-5-carboxylate (**9c**)

The sample was prepared from **8a** (0.341 g, 1 mmol), sodium acetate anhydrous (0.345 g, 4.2 mmol) and 2-methyl-2-thiopseudourea hemisulfate (0.390 g, 1.4 mmol) dissolved in dry methanol and the reaction was stirred at r.t. for 16 h. The obtained residue was purified by column chromatography (SiO_2_, eluent:ethylacetate/*n*-hexane, 1:6, *v*/*v*) to provide compound **9c** as a colorless liquid. Yield 0.183 g (48%), [*α*]_D_^20^ = −41.9 (*c* 0.610, MeOH). ^1^H NMR (400 MHz, CDCl_3_): *δ*_H_ ppm 1.45 (s, 9H, C(CH_3_)_3_), 1.50–1.61 (m, 1H, Pip 5-H), 1.73–1.82 (m, 2H, Pip 4,5-H), 1.94–1.97 (m, 1H, Pip 4-H), 2.49 (s, 3H, Pyr 6-CH_3_), 2.57 (s, 3H, S-CH_3_), 2.70–2.77 (m, 1H, Pip 6-H), 2.80–2.85 (m, 1H, Pip 3-H), 3.03–3.09 (m, 1H, Pip 2-H), 3.94 (s, 3H, COOCH_3_), 4.12–4.19 (m, 2H, Pip 2,6-H). ^13^C NMR (101 MHz, CDCl_3_): *δ*_C_ ppm 14.3 (S-CH_3_), 22.8 (Pyr C6-CH_3_), 25.0 (Pip C-5), 28.6 (COOC(CH_3_)_3_), 30.3 (Pip C-4), 42.2 (Pip C-3), 44.1 (Pip C-6), 48.1 (Pip C-2), 52.8 (COOCH_3_), 79.7 (COOC(CH_3_)_3_), 121.5 (Pyr C-5), 154.8 (COOC(CH_3_)_3_), 164.9 (Pyr C-6), 167.7 (COOCH_3_), 168.8 (Pyr C-4), 172.4 (Pyr C-2). ^15^N-NMR (71 MHz, CDCl_3_): *δ*_N_ ppm −108.9 (Pyr 1-N), Pyr 3-N and *N*-Boc were not found. IR (FT-IR, *ν*_max_, cm^−1^): 2930 (CH_aliph_), 1727 (C=O), 1689 (C=O), 1533, 1418, 1220 (C=C, C–N, C–O–C). HRMS (ESI^+^) C_18_H_28_N_3_O_4_S ([M+H]^+^) calcd. 382.1795, found 382.1795.

##### Methyl 4-[(3*S*)-1-(*tert*-butoxycarbonyl)piperidin-3-yl]-6-methyl-2-(methylsulfanyl)pyrimidine-5-carboxylate (**9d**)

The sample was prepared from **8b** (0.341 g, 1 mmol), sodium acetate anhydrous (0.345 g, 4.2 mmol) and 2-methyl-2-thiopseudourea hemisulfate (0.390 g, 1.4 mmol) dissolved in dry methanol and the reaction was stirred at r.t. for 16 h. The obtained residue was purified by column chromatography (SiO_2_, eluent:ethylacetate/*n*-hexane, 1:6, *v*/*v*) to provide compound **9d** as a colorless liquid. Yield 0.145 g (38%), [*α*]_D_^20^ = 41.8 (*c* 0.650, MeOH). ^1^H NMR (400 MHz, CDCl_3_): *δ*_H_ ppm 1.45 (s, 9H, C(CH_3_)_3_), 1.50–1.61 (m, 1H, Pip 5-H), 1.73–1.86 (m, 2H, Pip 4,5-H), 1.94–1.97 (m, 1H, Pip 4-H), 2.49 (s, 3H, Pyr 6-CH_3_), 2.57 (s, 3H, S-CH_3_), 2.70–2.77 (m, 1H, Pip 6-H), 2.79–2.85 (m, 1H, Pip 3-H), 3.03–3.09 (m, 1H, Pip 2-H), 3.94 (s, 3H, COOCH_3_), 4.12–4.19 (m, 2H, Pip 2,6-H). ^13^C NMR (101 MHz, CDCl_3_): *δ*_C_ ppm 14.3 (S-CH_3_), 22.7 (Pyr C6-CH_3_), 25.0 (Pip C-5), 28.6 (COOC(CH_3_)_3_), 30.3 (Pip C-4), 42.2 (Pip C-3), 44.1 (Pip C-6), 48.1 (Pip C-2), 52.8 (COOCH_3_), 79.7 (COOC(CH_3_)_3_), 121.5 (Pyr C-5), 154.8 (COOC(CH_3_)_3_), 164.9 (Pyr C-6), 167.7 (COOCH_3_), 168.8 (Pyr C-4), 172.3 (Pyr C-2). ^15^N-NMR (71 MHz, CDCl_3_): *δ*_N_ ppm −109.2 (Pyr 1-N), Pyr 3-N and *N*-Boc were not found. IR (FT-IR, *ν*_max_, cm^−1^): 2929 (CH_aliph_), 1727 (C=O), 1689 (C=O), 1533, 1418, 1221 (C=C, C–N, C–O–C). HRMS (ESI^+^) C_18_H_28_N_3_O_4_S ([M+H]^+^) calcd. 382.1795, found 382.1796.

#### 3.2.10. General Procedure for Compounds **10a,b**

Corresponding amino acid (1 equiv.), Meldrum‘s acid (1.1 equiv.) and DMAP (2 equiv.) were dissolved in methylene chloride and cooled to 0 °C. Maintaining the temperature at 0 °C, 1-ethyl-3-(3-dimethylaminopropyl)carbodiimide hydrochloride (1.1 equiv.) was added in portions and the solution was stirred at r.t. for 16 h. The reaction mixture was diluted with DCM (10 mL) and washed with 1M KHSO_4_ (2 × 15 mL) and brine (15 mL). The organic layer was dried with anhydrous sodium sulfate, filtered, and then concentrated under reduced pressure. The crude product was dissolved in 1,4-dioxane, then acetic acid and distilled water (2:1) were added. The reaction was stirred for 45 min at 100 °C (150 W) under microwave conditions. The resulting mixture was evaporated, neutralised with sat. NaHCO_3_ solution (10 mL) and extracted with ethylacetate (2 × 15 mL). The organic layer was dried with anhydrous sodium sulfate, filtered, concentrated under reduced pressure and used for further reactions.

#### 3.2.11. General Procedure for Compounds **11a,b**

NaH (60% dispersion in mineral oil, 5 equiv.) was added to a cooled (0 °C) suspension of ketone **10a,b** (1 equiv.) in dry ethyl acetate under an argon atmosphere. The reaction was stirred at 50 °C for 16 h. The resulting solution was quenched with 10% NH_4_Cl aq. solution (10 mL) and extracted with ethylacetate (2 × 15 mL). The organic layer was dried with anhydrous sodium sulfate, filtered, concentrated under reduced pressure and purified by flash chromatography to provide products **11a,b**.

##### *Tert*-butyl (3*R*)-3-[(1*Z*)-1-hydroxy-3-oxobut-1-en-1-yl]piperidine-1-carboxylate (**11a**)

NaH (60% dispersion in mineral oil, 0.2 g, 5 mmol) was added to a cooled (0 °C) suspension of ketone **10a** (0.227 g, 1 mmol) in dry ethyl acetate (3 mL) under an argon atmosphere. The reaction was stirred at 50 °C for 16 h. The resulting solution was quenched with 10% NH_4_Cl aq. solution (10 mL) and extracted with ethylacetate (2 × 15 mL). The organic layer was dried with anhydrous sodium sulfate, filtered, concentrated under reduced pressure and purified by column chromatography (SiO_2_, eluent:ethylacetate/*n*-hexane, 1:4, *v*/*v*) to provide compound **11a** as a yellowish oil. Yield 0.199 g (74%), [*α*]_D_^20^ = −0.8 (*c* 0.750, MeOH). ^1^H NMR (400 MHz, CDCl_3_): *δ*_H_ ppm 1.45–1.49 (m, 11H, C(CH_3_)_3_, Pip 5-H), 1.60–1.63 (m, 1H, Pip 4-H), 1.67–1.72 (m, 1H, Pip 5-H), 1.91–1.95 (m, 1H, Pip 4-H), 2.06 (s, 3H, COOCH_3_), 2.28–2.36 (m, 1H, Pip 3-H), 2.73–2.80 (m, 1H, Pip 6-H), 2.86–2.92 (m, 1H, Pip 2-H), 3.95–3.98 (m, 1H, Pip 6-H), 4.08–4.11 (m, 1H, Pip 2-H), 5.53 (s, 1H, Pip CH), 15.48 (OH). ^13^C NMR (101 MHz, CDCl_3_): *δ*_C_ ppm 24.5 (Pip C-5), 25.2 (COCH_3_), 27.8 (Pip C-4), 28.6 (COOC(CH_3_)_3_), 44.2 (Pip C-6), 44.5 (Pip C-3), 46.2 (Pip C-2), 79.8 (COOC(CH_3_)_3_), 99.1 (Pip C-3), 154.8 (COOC(CH_3_)_3_), 192.3 (COCH_3_), 194.1 (C-OH). ^15^N-NMR (71 MHz, CDCl_3_): *δ*_N_ ppm −295.0 (*N*-Boc). IR (FT-IR, *ν*_max_, cm^−1^): 2936 (CH_aliph_), 1688 (C=O), 1417, 1147 (C=C, C–N, C–O–C). HRMS (ESI^+^) C_14_H_23_NNaO_4_ ([M+Na]^+^) calcd. 292.1519, found 292.1519.

##### *Tert*-butyl (3*S*)-3-[(1*Z*)-1-hydroxy-3-oxobut-1-en-1-yl]piperidine-1-carboxylate (**11b**)

NaH (60% dispersion in mineral oil, 0.2 g, 5 mmol) was added to a cooled (0 °C) suspension of ketone **10b** (0.227 g, 1 mmol) in dry ethyl acetate (3 mL) under an argon atmosphere. The reaction was stirred at 50 °C for 16 h. The resulting solution was quenched with 10% NH_4_Cl aq. solution (10 mL) and extracted with ethylacetate (2 × 15 mL). The organic layer was dried with anhydrous sodium sulfate, filtered, concentrated under reduced pressure and purified by column chromatography (SiO_2_, eluent:ethylacetate/*n*-hexane, 1:4, *v*/*v*) to provide compound **11b** as a yellowish oil. Yield 0.188 g (70%), [*α*]_D_^20^ = 0.9 (*c* 0.735, MeOH). ^1^H NMR (400 MHz, CDCl_3_): *δ*_H_ ppm 1.45–1.49 (m, 11H, C(CH_3_)_3_, Pip 5-H), 1.57–1.64 (m, 1H, Pip 4-H), 1.67–1.72 (m, 1H, Pip 5-H), 1.91–1.96 (m, 1H, Pip 4-H), 2.06 (s, 3H, COOCH_3_), 2.29–2.36 (m, 1H, Pip 3-H), 2.73–2.80 (m, 1H, Pip 6-H), 2.86–2.92 (m, 1H, Pip 2-H), 3.95–3.98 (m, 1H, Pip 6-H), 4.08–4.12 (m, 1H, Pip 2-H), 5.54 (s, 1H, Pip CH), 15.49 (OH). ^13^C NMR (101 MHz, CDCl_3_): *δ*_C_ ppm 24.5 (Pip C-5), 25.2 (COCH_3_), 27.8 (Pip C-4), 28.6 (COOC(CH_3_)_3_), 43.9 (Pip C-6), 44.5 (Pip C-3), 46.1 (Pip C-2), 79.8 (COOC(CH_3_)_3_), 99.1 (Pip C-3), 154.8 (COOC(CH_3_)_3_), 192.3 (COCH_3_), 194.1 (C-OH). ^15^N-NMR (71 MHz, CDCl_3_): *δ*_N_ ppm −295.7 (*N*-Boc). IR (FT-IR, *ν*_max_, cm^−1^): 2935 (CH_aliph_), 1688 (C=O), 1417, 1146 (C=C, C–N, C–O–C). HRMS (ESI^+^) C_14_H_23_NNaO_4_ ([M+Na]^+^) calcd. 292.1519, found 292.1519.

#### 3.2.12. General Procedure for Compounds **12a,b**

Corresponding *β*-diketone **11a,b** (1 equiv.), sodium acetate anhydrous (4.2 equiv.) and 2-methyl-2-thiopseudourea hemisulfate (1.4 equiv.) were dissolved in DMF-DMA and stirred at 100 °C for 16 h. After completion of the reaction, solution was concentrated under reduced pressure and purified by flash chromatography to provide products **12a,b**.

##### *Tert*-butyl (3*R*)-3-[6-methyl-2-(methylsulfanyl)pyrimidin-4-yl]piperidine-1-carboxylate (**12a**)

The sample was prepared from **11a** (0.269 g, 1 mmol), sodium acetate anhydrous (0.345 g, 4.2 mmol) and 2-methyl-2-thiopseudourea hemisulfate (0.390 g, 1.4 mmol) dissolved in *N*,*N*-dimethylmethanamide and stirred at 100 °C for 16 h. The obtained residue was purified by column chromatography (SiO_2_, eluent:ethylacetate/*n*-hexane, 1:6, *v*/*v*) to provide compound **12a** as a yellowish oil. Yield 0.145 g (45%), [*α*]_D_^20^ = −2.3 (*c* 0.435, MeOH). ^1^H NMR (400 MHz, CDCl_3_): *δ*_H_ ppm 1.46 (s, 9H, C(CH_3_)_3_), 1.53–1.61 (m, 1H, Pip 5-H), 1.71–1.81 (m, 2H, Pip 4,5-H), 1.99–2.02 (m, 1H, Pip 4-H), 2.43 (s, 3H, Pyr 6-CH_3_), 2.56 (s, 3H, S-CH_3_), 2.68–2.73 (m, 1H, Pip 3-H), 2.78–2.84 (m, 1H, Pip 6-H), 2.97–3.07 (m, 1H, Pip 2-H), 3.97–4.10 (m, 1H, Pip 6-H), 4.14–4.24 (m, 1H, Pip 2-H), 6.71 (s, 1H, Pyr). ^13^C NMR (101 MHz, CDCl_3_): *δ*_C_ ppm 14.2 (S-CH_3_), 23.9 (Pyr C6-CH_3_), 24.9 (Pip C-5), 28.6 (COOC(CH_3_)_3_), 30.0 (Pip C-4), 43.6 (Pip C-3), 44.5 (Pip C-6), 47.9 (Pip C-2), 79.8 (COOC(CH_3_)_3_), 114.2 (Pyr C-5), 154.9 (COOC(CH_3_)_3_), 167.3 (COOCH_3_), 171.1 (Pyr C-4), 171.6 (Pyr C-2). ^15^N-NMR (71 MHz, CDCl_3_): *δ*_N_ ppm −294.6 (*N*-Boc), −106.6 (Pyr 1-N), −99.7 (Pyr 3-N). IR (FT-IR, *ν*_max_, cm^−1^): 2928 (CH_aliph_), 1688 (C=O), 1574, 1418, 1260, 1145 (C=C, C–N, C–O–C). HRMS (ESI^+^) C_16_H_26_N_3_O_2_S ([M+H]^+^) calcd. 324.1740, found 324.1740.

##### *Tert*-butyl (3*S*)-3-[6-methyl-2-(methylsulfanyl)pyrimidin-4-yl]piperidine-1-carboxylate (**12b**)

The sample was prepared from **11b** (0.269 g, 1 mmol), sodium acetate anhydrous (0.345 g, 4.2 mmol) and 2-methyl-2-thiopseudourea hemisulfate (0.390 g, 1.4 mmol) dissolved in *N*,*N*-dimethylmethanamide and stirred at 100 °C for 16 h. The obtained residue was purified by column chromatography (SiO_2_, eluent:ethylacetate/*n*-hexane, 1:6, *v*/*v*) to provide compound **12b** as a yellowish oil. Yield 0.171 g (53%), [*α*]_D_^20^ = 2.2 (*c* 0.450, MeOH). ^1^H NMR (400 MHz, CDCl_3_): *δ*_H_ ppm 1.46 (s, 9H, C(CH_3_)_3_), 1.53–1.62 (m, 1H, Pip 5-H), 1.72–1.81 (m, 2H, Pip 4,5-H), 1.99–2.03 (m, 1H, Pip 4-H), 2.44 (s, 3H, Pyr 6-CH_3_), 2.56 (s, 3H, S-CH_3_), 2.69–2.73 (m, 1H, Pip 3-H), 2.79–2.85 (m, 1H, Pip 6-H), 3.01–3.06 (m, 1H, Pip 2-H), 3.99–4.10 (m, 1H, Pip 6-H), 4.14–4.23 (m, 1H, Pip 2-H), 6.72 (s, 1H, Pyr). ^13^C NMR (101 MHz, CDCl_3_): *δ*_C_ ppm 14.2 (S-CH_3_), 23.9 (Pyr C6-CH_3_), 24.9 (Pip C-5), 28.6 (COOC(CH_3_)_3_), 30.0 (Pip C-4), 43.6 (Pip C-3), 44.3 (Pip C-6), 48.0 (Pip C-2), 79.8 (COOC(CH_3_)_3_), 114.2 (Pyr C-5), 154.9 (COOC(CH_3_)_3_), 167.2 (COOCH_3_), 171.2 (Pyr C-4), 171.6 (Pyr C-2). ^15^N-NMR (71 MHz, CDCl_3_): *δ*_N_ ppm −293.5 (*N*-Boc), −107.4 (Pyr 1-N), −99.2 (Pyr 3-N). IR (FT-IR, *ν*_max_, cm^−1^): 2928 (CH_aliph_), 1682 (C=O), 1573, 1406, 1262, 1136 (C=C, C–N, C–O–C). HRMS (ESI^+^) C_16_H_25_N_3_NaO_2_S ([M+Na]^+^) calcd. 346.1560, found 346.1560.

#### 3.2.13. General Procedure for Compounds **13a,b**

Intermediate pyrimidine **12a,b** (1 equiv.) was dissolved in pyridine (5 mL), and selenium dioxide (5eq.) was added. The reaction was heated at 115 °C for 16 h until completion of the reaction. Pyridine was evaporated and the product purified by flash chromatography to provide products **13a,b**.

##### 6-[(3*R*)-1-(*Tert*-butoxycarbonyl)piperidin-3-yl]-2-(methylsulfanyl)pyrimidine-4-carboxylic acid (**13a**)

The sample was prepared from **12a** (0.323 g, 1 mmol) dissolved in pyridine (5 mL), and selenium dioxide (0.55 g, 5 mmol) was added. The reaction was heated at 115 °C for 16 h until completion of the reaction. The obtained residue was purified by column chromatography (SiO_2_, eluent:methanol/methylene chloride, 5:100, *v*/*v*) to provide compound **13a** as a white amorphous compound. Yield 0.205 g (58%), [*α*]_D_^20^ = −39.7 (*c* 0.850, MeOH). ^1^H NMR (400 MHz, CDCl_3_): *δ*_H_ ppm 1.46 (s, 9H, C(CH_3_)_3_), 1.55–1.63 (m, 1H, Pip 5-H), 1.76–1.79 (m, 2H, Pip 4,5-H), 2.06–2.09 (m, 1H, Pip 4-H), 2.61 (s, 3H, S-CH_3_), 2.81–2.93 (m, 2H, Pip 3,6-H), 3.07 (s, 1H, Pip 2-H), 4.05–4.09 (m, 1H, Pip 6-H), 4.26 (s, 1H, Pip 2-H), 7.64 (s, 1H, Pyr). ^13^C NMR (101 MHz, CDCl_3_): *δ*_C_ ppm 14.4 (S-CH_3_), 24.8 (Pip C-5), 28.5 (COOC(CH_3_)_3_), 30.0 (Pip C-4), 44.0 (Pip C-3), 44.4 (Pip C-6), 47.8 (Pip C-2), 80.2 (COOC(CH_3_)_3_), 113.0 (Pyr C-5), 153.5 (Pyr C-4), 154.9 (COOC(CH_3_)_3_), 163.4 (COOH), 172.9 (Pyr C-2), 174.9 (Pyr C-6). ^15^N-NMR (71 MHz, CDCl_3_): *δ*_N_ ppm −293.9 (*N*-Boc), −117.4 (Pyr 3-N), −89.7 (Pyr 1-N). IR (FT-IR, *ν*_max_, cm^−1^): 3078, 2975 (CH_aliph_), 1718 (C=O), 1649 (C=O), 1536, 1429, 1267, 1150 (C=C, C–N, C–O–C). HRMS (ESI^+^) C_16_H_24_N_3_O_4_S ([M+H]^+^) calcd. 354.1482, found 354.1482.

##### 6-[(3*S*)-1-(*Tert*-butoxycarbonyl)piperidin-3-yl]-2-(methylsulfanyl)pyrimidine-4-carboxylic acid (**13b**)

The sample was prepared from **12b** (0.323 g, 1 mmol) dissolved in pyridine (5 mL), and selenium dioxide (0.55 g, 5 mmol) was added. The reaction was heated at 115 °C for 16 h until completion of the reaction. The obtained residue was purified by column chromatography (SiO_2_, eluent:methanol/methylene chloride, 5:100, *v*/*v*) to provide compound **13b** as a white amorphous compound. Yield 0.166 g (47%), [*α*]_D_^20^ = 39.8 (*c* 0.845, MeOH). ^1^H NMR (400 MHz, CDCl_3_): *δ*_H_ ppm 1.47 (s, 9H, C(CH_3_)_3_), 1.57–1.64 (m, 1H, Pip 5-H), 1.76–1.79 (m, 2H, Pip 4,5-H), 2.06–2.09 (m, 1H, Pip 4-H), 2.61 (s, 3H, S-CH_3_), 2.81–2.93 (m, 2H, Pip 3,6-H), 3.01–3.15 (m, 1H, Pip 2-H), 4.05–4.09 (m, 1H, Pip 6-H), 4.18–4.34 (s, 1H, Pip 2-H), 7.65 (s, 1H, Pyr). ^13^C NMR (101 MHz, CDCl_3_): *δ*_C_ ppm 14.4 (S-CH_3_), 24.8 (Pip C-5), 28.6 (COOC(CH_3_)_3_), 30.1 (Pip C-4), 44.0 (Pip C-3), 44.7 (Pip C-6), 47.7 (Pip C-2), 80.2 (COOC(CH_3_)_3_), 112.8 (Pyr C-5), 153.3 (Pyr C-4), 154.9 (COOC(CH_3_)_3_), 163.1 (COOH), 172.9 (Pyr C-2), 175.1 (Pyr C-6). ^15^N-NMR (71 MHz, CDCl_3_): *δ*_N_ ppm −294.1 (*N*-Boc), −118.5 (Pyr 3-N), −89.6 (Pyr 1-N). IR (FT-IR, *ν*_max_, cm^−1^): 3078, 2975 (CH_aliph_), 1718 (C=O), 1649 (C=O), 1537, 1430, 1267, 1151 (C=C, C–N, C–O–C). HRMS (ESI^+^) C_16_H_24_N_3_O_4_S ([M+H]^+^) calcd. 354.1482, found 354.1482.

#### 3.2.14. General Procedure for Compounds **14a,b**

To a solution of corresponding ketone **10a,b** (1 equiv.) in methanol, pTSA (0.1 equiv.) and trimethyl orthoformate were added. The solution was stirred at 60 °C for 3 h. The resulting solution was cooled on ice water, then triethylamine (5 equiv.) was added. The volatiles were removed under reduced pressure and the mixture was quenched with water (10 mL) and extracted with ethylacetate (2 × 15 mL). The organic layer was dried with anhydrous sodium sulfate, filtered, concentrated under reduced pressure and directly used in the next step without further purification.

#### 3.2.15. General Procedure for Compounds **15a,b**

To a stirred solution of ethyloxalyl chloride (2 equiv.) in dry chloroform at 0 °C, corresponding carboxylate **14a,b** (1 equiv.) and pyridine (2 equiv.) dissolved in dry chloroform were added dropwise, maintaining the temperature at 0 °C for 1 h. Then, the reaction was refluxed for 5 h. The resulting solution was quenched with a solution H_2_O:HCl (10:1) and extracted with chloroform (2 × 15 mL) and water (10 mL). The organic layer was dried with anhydrous sodium sulfate, filtered, and then concentrated under reduced pressure. After removal of the solvent in vacuo, the residue was directly used in the next step without further purification.

#### 3.2.16. General Procedure for Compounds **16a,b**

To a solution of the corresponding diketoester **15a,b** (1 equiv.) in absolute ethanol, sodium acetate anhydrous (4.2 equiv.) and 2-methyl-2-thiopseudourea hemisulfate (1.4 equiv.) were added and stirred at r.t. for 16 h. The resulting mixture was concentrated under reduced pressure and purified by flash chromatography to provide products **16a,b**.

##### Ethyl 6-[(3*R*)-1-(*tert*-butoxycarbonyl)piperidin-3-yl]-2-(methylsulfanyl)pyrimidine-4-carboxylate (**16a**)

The sample was prepared from **15a** (0.327 g, 1 mmol), sodium acetate anhydrous (0.345 g, 4.2 mmol) and 2-methyl-2-thiopseudourea hemisulfate (0.39 g, 1.4 mmol) dissolved in absolute ethanol. The reaction was stirred at r.t. for 16 h. The obtained residue was purified by column chromatography (SiO_2_, eluent:ethylacetate/*n*-hexane, 1:6, *v*/*v*) to provide compound **16a** as a white amorphous compound. Yield 0.26 g (68%), [*α*]_D_^20^ = −45.2 (*c* 1.490, MeOH). ^1^H NMR (400 MHz, CDCl_3_): *δ*_H_ ppm 1.39–1.46 (m, 12H, C(CH_3_)_3_, CH_2_-CH_3_), 1.55–1.61 (m, 1H, Pip 5-H), 1.73–1.82 (m, 2H, Pip 4,5-H), 2.04–2.07 (m, 1H, Pip 4-H), 2.60 (s, 3H, S-CH_3_), 2.78–2.84 (m, 2H, Pip 3,6-H), 2.94–3.17 (m, 1H, Pip 2-H), 4.05–4.08 (m, 1H, Pip 6-H), 4.18–4.33 (m, 1H, Pip 2-H), 4.42–4.47 (m, 2H, CH_2_-CH_3_), 7.48 (Pyr CH). ^13^C NMR (101 MHz, CDCl_3_): *δ*_C_ ppm 14.3 (CH_2_-CH_3_), 14.4 (S-CH_3_), 24.8 (Pip C-5), 28.6 (COOC(CH_3_)_3_), 30.0 (Pip C-4), 43.9 (Pip C-3), 44.6 (Pip C-6), 47.9 (Pip C-2), 62.6 (CH_2_-CH_3_), 79.9 (COOC(CH_3_)_3_), 114.1 (Pyr C-5), 154.9 (Pyr C-4), 155.6 (COOC(CH_3_)_3_), 164.3 (COOCH_2_CH_3_), 173.5 (Pyr C-2), 173.6 (Pyr C-6). ^15^N-NMR (71 MHz, CDCl_3_): *δ*_N_ ppm −107.2 (Pyr 1-N), −91.3 (Pyr 3-N), *N*-Boc was not found. IR (FT-IR, *ν*_max_, cm^−1^): 2925 (CH_aliph_), 1722 (C=O), 1689 (C=O), 1537, 1420, 1251, 1151 (C=C, C–N, C–O–C). HRMS (ESI^+^) C_18_H_28_N_3_O_4_S ([M+H]^+^) calcd. 382.1795, found 382.1795.

##### Ethyl 6-[(3*S*)-1-(*tert*-butoxycarbonyl)piperidin-3-yl]-2-(methylsulfanyl)pyrimidine-4-carboxylate (**16b**)

The sample was prepared from **15b** (0.327 g, 1 mmol), sodium acetate anhydrous (0.345 g, 4.2 mmol) and 2-methyl-2-thiopseudourea hemisulfate (0.39 g, 1.4 mmol) dissolved in absolute ethanol. The reaction was stirred at r.t. for 16 h. The obtained residue was purified by column chromatography (SiO_2_, eluent:ethylacetate/*n*-hexane, 1:6, *v*/*v*) to provide compound **16b** as a white amorphous compound. Yield 0.316 g (83%), [*α*]_D_^20^ = 45.1 (*c* 1.5, MeOH). ^1^H NMR (400 MHz, CDCl_3_): *δ*_H_ ppm 1.39–1.46 (m, 12H, C(CH_3_)_3_, CH_2_-CH_3_), 1.55–1.61 (m, 1H, Pip 5-H), 1.73–1.82 (m, 2H, Pip 4,5-H), 2.04–2.07 (m, 1H, Pip 4-H), 2.60 (s, 3H, S-CH_3_), 2.78–2.86 (m, 2H, Pip 3,6-H), 2.95–3.14 (m, 1H, Pip 2-H), 4.05–4.08 (m, 1H, Pip 6-H), 4.15–4.34 (m, 1H, Pip 2-H), 4.41–4.47 (m, 2H, CH_2_-CH_3_), 7.48 (Pyr CH). ^13^C NMR (101 MHz, CDCl_3_): *δ*_C_ ppm 14.3 (CH_2_-CH_3_), 14.4 (S-CH_3_), 24.8 (Pip C-5), 28.6 (COOC(CH_3_)_3_), 30.0 (Pip C-4), 43.9 (Pip C-3), 44.4 (Pip C-6), 47.9 (Pip C-2), 62.6 (CH_2_-CH_3_), 79.9 (COOC(CH_3_)_3_), 114.1 (Pyr C-5), 154.9 (Pyr C-4), 155.6 (COOC(CH_3_)_3_), 164.3 (COOCH_2_CH_3_), 173.5 (Pyr C-2), 173.6 (Pyr C-6). ^15^N-NMR (71 MHz, CDCl_3_): *δ*_N_ ppm −107.1 (Pyr 1-N), −91.7 (Pyr 3-N), *N*-Boc was not found. IR (FT-IR, *ν*_max_, cm^−1^): 2925 (CH_aliph_), 1722 (C=O), 1688 (C=O), 1537, 1420, 1250, 1150 (C=C, C–N, C–O–C). HRMS (ESI^+^) C_18_H_27_N_3_NaO_4_S ([M+Na]^+^) calcd. 404.1614, found 404.1614.

#### 3.2.17. General Procedure for Compounds **13’a,b**

To a solution of the corresponding compound**15a,b** (1 mmol.) in ethanol, 1M Na_2_CO_3_ (aq) (1 mL) and 2-methyl-2-thiopseudourea hemisulfate (1.4 equiv.) were added and stirred at r.t. for 16 h. The resulting mixture was concentrated under reduced pressure and purified by flash chromatography to provide products in good to excellent yields **13’a** (80%) and **13’b** (92%).

## 4. Conclusions

In conclusion, new synthetic approaches to obtain substituted pyrimidine-5-carboxylates and pyrimidine-4-carboxylic acids were developed. Cyclocondensation reactions provided the target pyrimidines in moderate-to-good yields, while sodium methoxide proved to be the most effective base under optimized conditions. The developed methodologies provide access to structurally diverse chiral pyrimidine derivatives, which are useful as heterocyclic building blocks. The structures of all synthesized compounds were confirmed by detailed NMR, IR, and HRMS data, and their enantiomeric purity was determined via chiral HPLC analysis.

Chiral HPLC analysis demonstrated that the stereochemical outcome depended strongly on the applied synthetic route. Pyrimidine-5-carboxylates prepared from *β*-enamino keto esters retained high enantiomeric excess (90.2–100% ee), whereas cyclization of *β*-diketones under strongly basic conditions at elevated temperature resulted in complete racemization (ee < 1%). Modification of the synthetic route and application of milder conditions partially suppressed racemization, affording pyrimidine derivatives with 61.8–65.5% ee. These results suggest that substrate structure influences stereochemical integrity during pyrimidine synthesis.

## Figures and Tables

**Table 1:** Optimizing reaction conditions of compound **4i**: base and reaction time effects.

Entry	Base *	Yield (%), 4 h **	Yield (%), 16 h **	Yield (%), 48 h **
1	NaH	37	15	5
2	*t*BuOK	41	32	21
3	Cs_2_CO_3_	39	40	35
**4**	**NaOCH_3_**	**45**	**60**	**49**
5	TEA	11	20	19
6	DIPEA	17	24	23
7	K_3_PO_4_	32	43	38
8	Na_2_CO_3_	32	37	39
9	K_2_CO_3_	16	48	29

* All reaction mixtures were stirred at room temperature, solvent MeOH. ** After purification by column chromatography. Bold indicates the optimized reaction conditions giving the highest isolated yield.

## Data Availability

The data presented in this study are available on request from the corresponding authors.

## Supplementary Materials

The following supporting information can be downloaded at: https://www.mdpi.com/article/10.3390/molecules31152689/s1, Figure S1: ^1^H NMR (400 MHz, CDCl_3_) spectrum of compound **4a**, Figure S2: ^13^C NMR (101 MHz, CDCl_3_) spectrum of compound **4a**, Figure S3: ^1^H–^1^H COSY NMR (400 MHz, CDCl_3_) spectrum of compound **4a**, Figure S4: ^1^H–^13^C HSQC NMR (400/101 MHz, CDCl_3_) spectrum of compound **4a**, Figure S5: ^1^H–^13^C HMBC NMR (400/101 MHz, CDCl_3_) spectrum of compound **4a**, Figure S6: ^1^H–^15^N HMBC NMR (71 MHz, CDCl_3_) spectrum of compound **4a**, Figure S7: HRMS (ESI-TOF) spectrum of compound **4a**, Figure S8: Chiral HPLC analysis of compound **4a**, Figure S9: ^1^H NMR (400 MHz, CDCl_3_) spectrum of compound **4b**, Figure S10: ^13^C NMR (101 MHz, CDCl_3_) spectrum of compound **4b**, Figure S11: HRMS (ESI-TOF) spectrum of compound **4b**, Figure S12: Chiral HPLC analysis of compound **4b**, Figure S13: ^1^H NMR (400 MHz, CDCl_3_) spectrum of compound **4c**, Figure S14: ^13^C NMR (101 MHz, CDCl_3_) spectrum of compound **4c**, Figure S15: HRMS (ESI-TOF) spectrum of compound **4c**, Figure S16: Chiral HPLC analysis of compound **4c**, Figure S17: ^1^H NMR (400 MHz, CDCl_3_) spectrum of compound **4d**, Figure S18: ^13^C NMR (101 MHz, CDCl_3_) spectrum of compound **4d**, Figure S19: HRMS (ESI-TOF) spectrum of compound **4d**, Figure S20: Chiral HPLC analysis of compound **4d**, Figure S21: ^1^H NMR (400 MHz, CDCl_3_) spectrum of compound **4e**, Figure S22: ^13^C NMR (101 MHz, CDCl_3_) spectrum of compound **4e**, Figure S23: HRMS (ESI-TOF) spectrum of compound **4e**, Figure S24: Chiral HPLC analysis of compound **4e**, Figure S25: ^1^H NMR (400 MHz, CDCl_3_) spectrum of compound **4f**, Figure S26: ^13^C NMR (101 MHz, CDCl_3_) spectrum of compound **4f**, Figure S27: HRMS (ESI-TOF) spectrum of compound **4f**, Figure S28: Chiral HPLC analysis of compound **4f**, Figure S29: ^1^H NMR (400 MHz, CDCl_3_) spectrum of compound **4g**, Figure S30: ^13^C NMR (101 MHz, CDCl_3_) spectrum of compound **4g**, Figure S31: HRMS (ESI-TOF) spectrum of compound **4g**, Figure S32: Chiral HPLC analysis of compound **4g**, Figure S33: ^1^H NMR (400 MHz, CDCl_3_) spectrum of compound **4h**, Figure S34: ^13^C NMR (101 MHz, CDCl_3_) spectrum of compound **4h**, Figure S35: HRMS (ESI-TOF) spectrum of compound **4h**, Figure S36: Chiral HPLC analysis of compound **4h**, Figure S37: ^1^H NMR (400 MHz, CDCl_3_) spectrum of compound **4i**, Figure S38: ^13^C NMR (101 MHz, CDCl_3_) spectrum of compound **4i**, Figure S39: HRMS (ESI-TOF) spectrum of compound **4i**, Figure S40: Chiral HPLC analysis of compound **4i**, Figure S41: ^1^H NMR (400 MHz, CDCl_3_) spectrum of compound **4j**, Figure S42: ^13^C NMR (101 MHz, CDCl_3_) spectrum of compound **4j**, Figure S43: HRMS (ESI-TOF) spectrum of compound **4j**, Figure S44: Chiral HPLC analysis of compound **4j**, Figure S45: ^1^H NMR (400 MHz, CDCl_3_) spectrum of compound **4k**, Figure S46: ^13^C NMR (101 MHz, CDCl_3_) spectrum of compound **4k**, Figure S47: HRMS (ESI-TOF) spectrum of compound **4k**, Figure S48: Chiral HPLC analysis of compound **4k**, Figure S49: ^1^H NMR (400 MHz, CDCl_3_) spectrum of compound **4l**, Figure S50: ^13^C NMR (101 MHz, CDCl_3_) spectrum of compound **4l**, Figure S51: HRMS (ESI-TOF) spectrum of compound **4l**, Figure S52: Chiral HPLC analysis of compound **4l**, Figure S53: ^1^H NMR (400 MHz, CDCl_3_) spectrum of compound **4m**, Figure S54: ^13^C NMR (101 MHz, CDCl_3_) spectrum of compound **4m**, Figure S55: HRMS (ESI-TOF) spectrum of compound **4m**, Figure S56: Chiral HPLC analysis of compound **4m**, Figure S57: ^1^H NMR (400 MHz, CDCl_3_) spectrum of compound **4n**, Figure S58: ^13^C NMR (101 MHz, CDCl_3_) spectrum of compound **4n**, Figure S59: HRMS (ESI-TOF) spectrum of compound **4n**, Figure S60: Chiral HPLC analysis of compound **4n**, Figure S61: ^1^H NMR (400 MHz, CDCl_3_) spectrum of compound **4o**, Figure S62: ^13^C NMR (101 MHz, CDCl_3_) spectrum of compound **4o**, Figure S63: HRMS (ESI-TOF) spectrum of compound **4o**, Figure S64: Chiral HPLC analysis of compound **4o**, Figure S65: ^1^H NMR (400 MHz, CDCl_3_) spectrum of compound **4p**, Figure S66: ^13^C NMR (101 MHz, CDCl_3_) spectrum of compound **4p**, Figure S67: HRMS (ESI-TOF) spectrum of compound **4p**, Figure S68: Chiral HPLC analysis of compound **4p**, Figure S69: ^1^H NMR (400 MHz, CDCl_3_) spectrum of compound **6a**, Figure S70: ^13^C NMR (101 MHz, CDCl_3_) spectrum of compound **6a**, Figure S71: HRMS (ESI-TOF) spectrum of compound **6a**, Figure S72: Chiral HPLC analysis of compound **6a**, Figure S73: ^1^H NMR (400 MHz, CDCl_3_) spectrum of compound **6b**, Figure S74: ^13^C NMR (101 MHz, CDCl_3_) spectrum of compound **6b**, Figure S75: HRMS (ESI-TOF) spectrum of compound **6b**, Figure S76: Chiral HPLC analysis of compound **6b**, Figure S77: ^1^H NMR (400 MHz, CDCl_3_) spectrum of compound **6c**, Figure S78: ^13^C NMR (101 MHz, CDCl_3_) spectrum of compound **6c**, Figure S79: HRMS (ESI-TOF) spectrum of compound **6c**, Figure S80: Chiral HPLC analysis of compound **6c**, Figure S81: ^1^H NMR (400 MHz, CDCl_3_) spectrum of compound **6d**, Figure S82: ^13^C NMR (101 MHz, CDCl_3_) spectrum of compound **6d**, Figure S83: HRMS (ESI-TOF) spectrum of compound **6d**, Figure S84: Chiral HPLC analysis of compound **6d**, Figure S85: ^1^H NMR (400 MHz, CDCl_3_) spectrum of compound **9a**, Figure S86: ^13^C NMR (101 MHz, CDCl_3_) spectrum of compound **9a**, Figure S87: HRMS (ESI-TOF) spectrum of compound **9a**, Figure S88: Chiral HPLC analysis of compound **9a**, Figure S89: ^1^H NMR (400 MHz, CDCl_3_) spectrum of compound **9b**, Figure S90: ^13^C NMR (101 MHz, CDCl_3_) spectrum of compound **9b**, Figure S91: HRMS (ESI-TOF) spectrum of compound **9b**, Figure S92: Chiral HPLC analysis of compound **9b**, Figure S93: ^1^H NMR (400 MHz, CDCl_3_) spectrum of compound **9c**, Figure S94: ^13^C NMR (101 MHz, CDCl_3_) spectrum of compound **9c**, Figure S95: HRMS (ESI-TOF) spectrum of compound **9c**, Figure S96: Chiral HPLC analysis of compound **9c**, Figure S97: ^1^H NMR (400 MHz, CDCl_3_) spectrum of compound **9d**, Figure S98: ^13^C NMR (101 MHz, CDCl_3_) spectrum of compound **9d**, Figure S99: HRMS (ESI-TOF) spectrum of compound **9d**, Figure S100: Chiral HPLC analysis of compound **9d**, Figure S101: ^1^H NMR (400 MHz, CDCl_3_) spectrum of compound **11a**, Figure S102: ^13^C NMR (101 MHz, CDCl_3_) spectrum of compound **11a**, Figure S103: HRMS (ESI-TOF) spectrum of compound **11a**, Figure S104: ^1^H NMR (400 MHz, CDCl_3_) spectrum of compound **11b**, Figure S105: ^13^C NMR (101 MHz, CDCl_3_) spectrum of compound **11b**, Figure S106: HRMS (ESI-TOF) spectrum of compound **11b**, Figure S107: ^1^H NMR (400 MHz, CDCl_3_) spectrum of compound **12a**, Figure S108: ^13^C NMR (101 MHz, CDCl_3_) spectrum of compound **12a**, Figure S109: HRMS (ESI-TOF) spectrum of compound **12a**, Figure S110: Chiral HPLC analysis of compound **12a**, Figure S111: ^1^H NMR (400 MHz, CDCl_3_) spectrum of compound **12b**, Figure S112: ^13^C NMR (101 MHz, CDCl_3_) spectrum of compound **12b**, Figure S113: HRMS (ESI-TOF) spectrum of compound **12b**, Figure S114: Chiral HPLC analysis of compound **12b**, Figure S115: ^1^H NMR (400 MHz, CDCl_3_) spectrum of compound **13a**, Figure S116: ^13^C NMR (101 MHz, CDCl_3_) spectrum of compound **13a**, Figure S117: HRMS (ESI-TOF) spectrum of compound **13a**, Figure S118: Chiral HPLC analysis of compound **13a**, Figure S119: Chiral HPLC analysis of compound **13′a**, Figure S120: ^1^H NMR (400 MHz, CDCl_3_) spectrum of compound **13b**, Figure S121: ^13^C NMR (101 MHz, CDCl_3_) spectrum compound **13b**, Figure S122: HRMS (ESI-TOF) spectrum of compound **13b**, Figure S123: Chiral HPLC analysis of compound **13b**, Figure S124: Chiral HPLC analysis of compound **13′b**, Figure S125: ^1^H NMR (400 MHz, CDCl_3_) spectrum of compound **16a**, Figure S126: ^13^C NMR (101 MHz, CDCl_3_) spectrum of compound **16a**, Figure S127: HRMS (ESI-TOF) spectrum of compound **16a**, Figure S128: Chiral HPLC analysis of compound **16a**, Figure S129: ^1^H NMR (400 MHz, CDCl_3_) spectrum of compound **16b**, Figure S130: ^13^C NMR (101 MHz, CDCl_3_) spectrum of compound **16b**, Figure S131: HRMS (ESI-TOF) spectrum of compound **16b**, Figure S132: Chiral HPLC analysis of compound **16b**, Figure S133: Chiral HPLC analysis of racemic mixture **4a** and **4b**, Figure S134: Chiral HPLC analysis of racemic mixture **13′a** and **13′b**, Figure S135: Chiral HPLC analysis of racemic mixture **16a** and **16b**. Figure S136: Chiral HPLC analysis of compounds **10a**, **10b** and their racemic mixture, Figure S137: Chiral HPLC analysis of compounds **11a** and **11b**, Table S1: Summary of Enantiomeric Excess Values and Chiral HPLC Conditions.

## References

[1] Bongioanni A., Bueno M.S., Mezzano B.A., Longhi M.R., Garnero C. (2022). Amino acids and their pharmaceutical applications: A mini review. Int. J. Pharm..

[2] Dai Z., Zheng W., Locasale J.W. (2022). Amino acid variability, tradeoffs and optimality in human diet. Nat. Commun..

[3] Wang Z. (2026). Amino acids as multifunctional molecules in plants: From fundamental metabolism to precision agriculture. Plants.

[4] Petkova D., Stoyanova S., Dinkov G., Bogdanov M. (2025). Beyond protein building blocks: A review of biological roles and therapeutic potential of free amino acids. Int. J. Mol. Sci..

[5] Jin Y., Jiang M., Wang H., Fu H. (2016). Installing amino acids and peptides on *N*-heterocycles under visible-light assistance. Sci. Rep..

[6] Sylvianingsih F., Supratman U., Maharani R. (2025). Amino acid- and peptide-conjugated heterocyclic compounds: A comprehensive review of synthesis strategies and biological activities. Eur. J. Med. Chem..

[7] Sharma K.K., Sharma K., Rao K., Sharma A., Rathod G.K., Aaghaz S., Sehra N., Parmar R., VanVeller B., Jain R. (2024). Unnatural amino acids: Strategies, designs, and applications in medicinal chemistry and drug discovery. J. Med. Chem..

[8] Kerru N., Gummidi L., Maddila S., Gangu K.K., Jonnalagadda S.B. (2020). A review on recent advances in nitrogen-containing molecules and their biological applications. Molecules.

[9] Kumar S., Kumar A., Misra S.K., Tiwari A., Verma S.S., Singh A. (2025). Indazole, a privileged scaffold: Synthetic strategies, biological potential, and future prospects. Lett. Drug Des. Discov..

[10] Marshall C.M., Federice J.G., Bell C.N., Cox P.B., Njardarson J.T. (2024). An update on the nitrogen heterocycle compositions and properties of U.S. FDA-approved pharmaceuticals. J. Med. Chem..

[11] Luo W., Liu Y., Qin H., Zhao Z., Wang S., He W., Tang S., Peng J. (2024). Nitrogen-containing heterocyclic drug products approved by the U.S. Food and Drug Administration in 2023: Synthesis and biological activity. Eur. J. Med. Chem..

[12] Blaskovich M.A.T. (2016). Unusual amino acids in medicinal chemistry. J. Med. Chem..

[13] Sardina F.J., Rapoport H. (1996). Enantiospecific synthesis of heterocycles from *α*-amino acids. Chem. Rev..

[14] Castro T.G., Melle-Franco M., Sousa C.E.A., Cavaco-Paulo A., Marcos J.C. (2023). Non-canonical amino acids as building blocks for peptidomimetics: Structure, function, and applications. Biomolecules.

[15] Singh P., Samanta K., Das S.K., Panda G. (2014). Amino acid chirons: A tool for asymmetric synthesis of heterocycles. Org. Biomol. Chem..

[16] Vale N., Ferreira A., Matos J., Fresco P., Gouveia M.J. (2018). Amino acids in the development of prodrugs. Molecules.

[17] Žukauskaitė A., Moretto A., Peggion C., De Zotti M., Šačkus A., Formaggio F., De Kimpe N., Mangelinckx S. (2014). Synthesis and conformational study of model peptides containing *N*-substituted 3-aminoazetidine-3-carboxylic acids. Eur. J. Org. Chem..

[18] Frolov N.A., Vereshchagin A.N., Vereshchagina Y.A., Makarov V.V., Safronova T.S., Slepukhin P.A., Benassi E., Elinson M.N. (2023). Piperidine derivatives: Recent advances in synthesis and pharmacological applications. Int. J. Mol. Sci..

[19] Al-Rooqi M.M., Mughal E.U., Raja Q.A., Obaid R.J., Sadiq A., Naeem N., Qurban J., Asghar B.H., Moussa Z., Ahmed S.A. (2022). Recent advancements on the synthesis and biological significance of pipecolic acid and its derivatives. J. Mol. Struct..

[20] Bonina F.B., Arenare L., Palagiano F., Nava F., Trombetta D., De Caprariis P. (1999). Synthesis, stability, and pharmacological evaluation of nipecotic acid prodrugs. J. Pharm. Sci..

[21] Luer M.S., Rhoney D.H. (1998). Tiagabine: A novel antiepileptic drug. Ann. Pharmacother..

[22] Leach J.P., Brodie M.J. (1998). Tiagabine. Lancet.

[23] Andersen K.E., Sørensen J.L., Lau J., Lundt B.F., Petersen H., Huusfeldt P.O., Suzdak P.D., Swedberg M.D.B. (2001). Synthesis of novel *γ*-aminobutyric acid (GABA) uptake inhibitors. 5. Preparation and structure–activity studies of tricyclic analogues of known GABA uptake inhibitors. J. Med. Chem..

[24] Seth A., Sharma A.P., Tripathi A., Choubey P.K., Srivastava P., Tripathi P.N., Shrivastava S.K. (2018). Design, synthesis, evaluation and molecular modeling studies of some novel *N*-substituted piperidine-3-carboxylic acid derivatives as potential anticonvulsants. Med. Chem. Res..

[25] Schaarschmidt M., Höfner G., Wanner K.T. (2019). Synthesis and biological evaluation of nipecotic acid and guvacine derived 1,3-disubstituted allenes as inhibitors of murine GABA transporter mGAT1. ChemMedChem.

[26] Kerr D.I., Ong J. (1992). GABA agonists and antagonists. Med. Res. Rev..

[27] Frølund B., Ebert B., Kristiansen U., Liljefors T., Krogsgaard-Larsen P. (2002). GABA_(A)_ receptor ligands and their therapeutic potentials. Curr. Top. Med. Chem..

[28] Kee W.D.N. (1998). Epidural pethidine: Pharmacology and clinical experience. Anaesth. Intensive Care.

[29] Latta K., Ginsberg B., Barkin R.L. (2002). Meperidine: A critical review. Am. J. Ther..

[30] Matulevičiūtė G., Arbačiauskienė E., Kleizienė N., Kederienė V., Ragaitė G., Dagilienė M., Bieliauskas A., Milišiūnaitė V., Sløk F.A., Šačkus A. (2021). Synthesis and characterization of novel methyl (3)5-(*N*-Boc-piperidinyl)-1*H*-pyrazole-4-carboxylates. Molecules.

[31] Bruzgulienė J., Račkauskienė G., Bieliauskas A., Milišiūnaitė V., Dagilienė M., Matulevičiūtė G., Martynaitis V., Krikštolaitytė S., Sløk F.A., Šačkus A. (2022). Regioselective synthesis of methyl 5-(*N*-Boc-cycloaminyl)-1,2-oxazole-4-carboxylates as new amino acid-like building blocks. Beilstein J. Org. Chem..

[32] Turk S., Blanot D., Gobec S., Živec M. (2011). Design and synthesis of new peptidomimetics as potential inhibitors of MurE. Acta Chim. Slov..

[33] Cho J.-i., Tanaka M., Sato S., Kinbara K., Aida T. (2010). Oligo(4-aminopiperidine-4-carboxylic acid): An unusual basic oligopeptide with an acid-induced helical conformation. J. Am. Chem. Soc..

[34] Wysong C.L., Yokum T.S., Morales G.A., Gundry R.L., McLaughlin M.L., Hammer R.P. (1996). 4-Aminopiperidine-4-carboxylic acid: A cyclic *α,α*-disubstituted amino acid for preparation of water-soluble highly helical peptides. J. Org. Chem..

[35] Bąchor U., Mączyński M. (2021). Selected *β*^2^-, *β*^3^- and *β*^2,3^-amino acid heterocyclic derivatives and their biological perspective. Molecules.

[36] Saitton S., Kihlberg J., Luthman K. (2004). A synthetic approach to 2,3,4-substituted pyridines useful as scaffolds for tripeptidomimetics. Tetrahedron.

[37] Croce P.D., Rosa C.L., Pizzatti E. (2000). Stereoselective synthesis of 3-heteroaromatic-substituted alanines. Tetrahedron Asymmetry.

[38] Ueyama N., Yanagisawa T., Kawai T., Sonegawa M., Baba H., Mochizuki S., Kosakai K., Tomiyama T. (1994). Nonpeptide angiotensin II receptor antagonists. I. Synthesis and biological activity of pyridine derivatives. Chem. Pharm. Bull..

[39] Kopka I.E., Young D.N., Lin L.S., Mumford R.A., Magriotis P.A., MacCoss M., Mills S.G., Van Riper G., McCauley E., Egger L.E. (2002). Substituted *N*-(3,5-dichlorobenzenesulfonyl)-*L*-prolyl-phenylalanine analogues as potent VLA-4 antagonists. Bioorg. Med. Chem. Lett..

[40] Hall C., Wolfe H., Wells A., Chien H.-C., Colas C., Schlessinger A., Giacomini K.M., Thomas A.A. (2019). *L*-Type amino acid transporter 1 activity of 1,2,3-triazolyl analogs of *L*-histidine and *L*-tryptophan. Bioorg. Med. Chem. Lett..

[41] Nammalwar B., Bunce R.A. (2024). Recent advances in pyrimidine-based drugs. Pharmaceuticals.

[42] Venugopala K.N., Kamat V. (2024). Pyrimidines: A new versatile molecule in the drug development field, scope, and future aspects. Pharmaceuticals.

[43] Ignatowska J., Mironiuk-Puchalska E., Grześkowiak P., Wińska P., Wielechowska M., Bretner M., Karatsai O., Rędowicz M.J., Koszytkowska-Stawińska M. (2020). New insight into nucleo *α*-amino acids—Synthesis and SAR studies on cytotoxic activity of *β*-pyrimidine alanines. Bioorg. Chem..

[44] Harrison D., Boutard N., Brzozka K., Bugaj M., Chmielewski S., Cierpich A., Doedens J.R., Fabritius C.-H.R.Y., Gabel C.A., Galezowski M. (2020). Discovery of a series of ester-substituted NLRP3 inflammasome inhibitors. Bioorg. Med. Chem. Lett..

[45] Palumbo R., Omodei D., Vicidomini C., Roviello G.N. (2022). Willardiine and its synthetic analogues: Biological aspects and implications in peptide chemistry of this nucleobase amino acid. Pharmaceuticals.

[46] Jane D.E., Hoo K., Kamboj R., Deverill M., Bleakman D., Mandelzys A. (1997). Synthesis of willardiine and 6-azawillardiine analogs: Pharmacological characterization on cloned homomeric human AMPA and kainate receptor subtypes. J. Med. Chem..

[47] Martis G.J., Mugali P.S., Gaonkar S.L. (2025). Recent progress in the synthesis, functionalization, and biological outlook of pyrimidines. ACS Omega.

[48] Mahfoudh M., Abderrahim R., Leclerc E., Campagne J.-M. (2017). Recent approaches to the synthesis of pyrimidine derivatives. Eur. J. Org. Chem..

[49] Basato M., Corain B., Marcomini A., Valle G., Zanotti G. (1984). Synthesis of pyrimidines from *β*-dicarbonyl compounds and cyanogen: A metal-catalysed one-pot process. J. Chem. Soc. Perkin Trans. 2.

[50] Vidal M., García-Arriagada M., Rezende M.C., Domínguez M. (2016). Ultrasound-promoted synthesis of 4-pyrimidinols and their tosyl derivatives. Synthesis.

[51] Mittersteiner M., Andrade V.P., Bonacorso H.G., Martins M.A.P., Zanatta N. (2020). The wonderful world of *β*-enamino diketones chemistry. Eur. J. Org. Chem..

[52] Díaz-Fernández M., Calvo-Losada S., Quirante J.-J., Sarabia F., Algarra M., Pino-González M.-S. (2023). Catalyzed methods to synthesize pyrimidine and related heterocyclic compounds. Catalysts.

[53] Martins M.A.P., Frizzo C.P., Moreira D.N., Buriol L., Machado P. (2015). Solvent-free heterocyclic synthesis. Molecules.

[54] Kajal K., Shakya R., Rashid M., Nigam V., Kurmi B.D., Gupta G.D., Patel P. (2024). Recent green chemistry approaches for pyrimidine derivatives as a potential anti-cancer agent: An overview (2013–2023). Sustain. Chem. Pharm..

[55] Xie M.-S., Zhou P., Niu H.-Y., Qu G.-R., Guo H.-M. (2016). Enantioselective intermolecular cyclopropanations for the synthesis of chiral pyrimidine carbocyclic nucleosides. Org. Lett..

[56] Singh A.K., Kumar A., Singh H., Sonawane P., Paliwal H., Thareja S., Pathak P., Grishina M., Jaremko M., Emwas A.-H. (2022). Concept of hybrid drugs and recent advancements in anticancer hybrids. Pharmaceuticals.

[57] Morais T.S. (2024). Recent advances in the development of hybrid drugs. Pharmaceutics.

[58] Das S., Choudhury S.B., Chauhan S., Balamurali M.M., Chanda K. (2025). An overview of the synthesis of biheterocyclic compounds featuring four-membered rings containing nitrogen, oxygen, or sulfur. Tetrahedron.

[59] Yildi M., Erşatır M., Giray S., Yalın S. (2020). Synthesis and antiproliferative evaluation of novel biheterocycles based on coumarin and 2-aminoselenophene-3-carbonitrile unit. Monatsh. Chem..

[60] Talukdar V., Mondal K., Halder P., Das P. (2024). Ullmann-type *N*-, *S*-, and *O*-arylation using a well-defined 7-azaindole-*N*-oxide (7-AINO)-based copper(II) catalyst: Scope and application to drug synthesis. J. Org. Chem..

[61] Gupta A.K., Talukder M., Venkataraman M., Bamimore M.A. (2022). Minoxidil: A comprehensive review. J. Dermatol. Treat..

[62] Gen S., Tanaka I., Morise M., Koyama J., Kodama Y., Matsui A., Miyazawa A., Hase T., Hibino Y., Yokoyama T. (2022). Clinical efficacy of osimertinib in EGFR-mutant non-small cell lung cancer with distant metastasis. BMC Cancer.

[63] Trabocchi A., Scarpi D., Guarna A. (2008). Structural diversity of bicyclic amino acids. Amino Acids.

[64] Otani Y., Park S., Ohwada T. (2020). Conformational preference of bicyclic *β*-amino acid dipeptides. Chirality.

[65] Chalyk B.A., Kandaurova I.Y., Hrebeniuk K.V., Manoilenko O.V., Kulik I.B., Iminov R.T., Kubyshkin V., Tverdokhlebov A.V., Ablialimov O.K., Mykhailiuk P.K. (2016). A base-promoted multigram synthesis of aminoisoxazoles: Valuable building blocks for drug discovery and peptidomimetics. RSC Adv..

[66] Gudelis E., Krikštolaitytė S., Stančiauskaitė M., Šachlevičiūtė U., Bieliauskas A., Milišiūnaitė V., Jankauskas R., Kleizienė N., Sløk F.A., Šačkus A. (2023). Synthesis of new azetidine and oxetane amino acid derivatives through aza-Michael addition of *NH*-heterocycles with methyl 2-(azetidin- or oxetan-3-ylidene)acetates. Molecules.

[67] Malinauskienė V., Kveselytė A., Dzedulionytė K., Bieliauskas A., Burinskas S., Sløk F.A., Šačkus A. (2018). *L*-Proline and related chiral heterocyclic amino acids as scaffolds for the synthesis of functionalized 2-amino-1,3-selenazole-5-carboxylates. Chem. Heterocycl. Compd..

[68] Blakskjær P., Hansen T.N., Petersen L.K., Sløk F.A., Hansen N.J.V. (2026). Efficient 96-well plate conjugation of unnatural amino acid building blocks for DNA-encoded library synthesis. Bioconjugate Chem..

[69] Petersen L.K., Christensen A.B., Andersen J., Folkesson C.G., Kristensen O., Andersen C., Alzu A., Sløk F.A., Blakskjær P., Madsen D. (2021). Screening of DNA-encoded small molecule libraries inside a living cell. J. Am. Chem. Soc..

[70] Brooks D.W., Lu L.D.-L., Masamune S. (1979). C-Acylation under virtually neutral conditions. Angew. Chem. Int. Ed. Engl..

[71] Greck C., Thomassigny C., Le Bouc G. (2012). Asymmetric syntheses of functionalized pyrrolizidin-3-ones. ARKIVOC.

[72] Gao W., Lau T., Pan S., Phillips D.P., Wang X. (2012). Compounds and Compositions as TGR5 Agonists.

[73] Xu W., Wang Y., Ma S., Koltun E.S., Kim B.G., Jeong J.W., Dhar T.G.M., Bannen L.C. (2014). RORY Modulators.

[74] Vashchenko B.V., Grygorenko O.O., Stepaniuk O.O. (2022). Heterocyclizations of *β*-alkoxy, *β*-diaminoalkyl, and related *β*-functionalized enones (enals) with NCN-binucleophiles. Ukr. Bioorganica Acta.

[75] Nishimura Y. (2011). Synthetic studies on novel 1,4-dihydro-2-methylthio-4,4,6-trisubstituted pyrimidine-5-carboxylic acid esters and their tautomers. Chem. Pharm. Bull..

[76] Bennett S.N.L. (2011). Adamantyl Iminocarbonyl-Substituted Pyrimidines as Inhibitors of 11-beta-HSD1.

[77] Goldberg F.W., Leach A.G., Scott J.S., Snelson W.L., Groombridge S.D., Donald C.S., Bennett S.N.L., Bodin C., Gutierrez P.M., Gyte A.C. (2012). Free-Wilson and structural approaches to co-optimizing human and rodent isoform potency for 11*β*-hydroxysteroid dehydrogenase type 1 (11*β*-HSD1) inhibitors. J. Med. Chem..

[78] Shkoor M., Riahi A., Fatunsin O., Hussain I., Yawer M.A., Lubbe M., Reim S., Reinke H., Fischer C., Langer P. (2009). Diversity-oriented synthesis of 1-hydroxy-2,4-benzodioates by regioselective [3+3] cyclocondensations of 1,3-bis(silyloxy)-1,3-butadienes with 3-alkoxy- and 3-silyloxy-2-alkoxycarbonyl-2-en-1-ones. Org. Biomol. Chem..

[79] Bauer W., Ossiander T., Weber B. (2010). A promising new Schiff base-like ligand for the synthesis of octahedral iron(II) spin crossover complexes. Z. Naturforsch. B.

[80] Bingham S.J., Tyman J.H.P. (2001). Improved synthesis of alkyl diacetylacetates. Org. Prep. Proced. Int..

[81] Lemoine R.C., Petersen A.C., Setti L., Wanner J., Jekle A., Heilek G., deRosier A., Ji C., Berry P., Rotstein D. (2010). Evaluation of secondary amide replacements in a series of CCR5 antagonists as a means to increase intrinsic membrane permeability. Part 1: Optimization of gem-disubstituted azacycles. Bioorg. Med. Chem. Lett..

[82] Xu D., Zhu Z., Wang Z. (2013). A convenient synthesis of 4,6-dimethyl-2-(methylsulfonyl)pyrimidine. J. Chem. Res..

[83] Awadallah F.M., Piazza G.A., Gary B.D., Keeton A.B., Canzoneri J.C. (2013). Synthesis of some dihydropyrimidine-based compounds bearing pyrazoline moiety and evaluation of their antiproliferative activity. Eur. J. Med. Chem..

[84] Mao Q., Xu G., Wang Y., Wen X., Wang J., Zhang X., Liu X., Lu X., Xu Y., Zhang H. (2019). Design, synthesis and biological evaluation of 2-(4-alkoxy-3-cyano)phenyl-6-oxo-1,6-dihydropyrimidine-5-carboxylic acid derivatives as novel xanthine oxidase inhibitors. Eur. J. Med. Chem..

[85] Zhao J., Mao Q., Lin F., Zhang B., Sun M., Zhang T., Wang S. (2022). Intramolecular hydrogen bond interruption and scaffold hopping of TMC-5 led to 2-(4-alkoxy-3-cyanophenyl)pyrimidine-4/5-carboxylic acids and 6-(4-alkoxy-3-cyanophenyl)-1,2-dihydro-3*H*-pyrazolo[3,4-d]pyrimidin-3-ones as potent pyrimidine-based xanthine oxidase inhibitors. Eur. J. Med. Chem..

[86] Lee Y.S., Kim H.Y., Kim Y., Seo J.H., Roh E.J., Han H., Shin K.J. (2012). Small molecules that protect against *β*-amyloid-induced cytotoxicity by inhibiting aggregation of *β*-amyloid. Bioorg. Med. Chem..

[87] Młochowski J., Wójtowicz-Młochowska H. (2015). Developments in synthetic application of selenium(IV) oxide and organoselenium compounds as oxygen donors and oxygen-transfer agents. Molecules.

[88] Machado P., Rosa F.A., Rossatto M., Sant’Anna G.S., Sauzem P.D., Silva R.M.S., Rubin M.A., Ferreira J., Bonacorso H.G., Zanatta N. (2008). Synthesis and structure of novel 4,5-dihydro-1*H*-pyrazoles: Salicylic acid based analgesic agents. ARKIVOC.

