# Supplementary material for: Synthesis and Characterization of New Functionalized Pyrimidine (Hetero)Cyclic Molecular Hybrids as Chiral Heterocyclic Amino Acid Derivatives
Source: Molecules. 2026 Aug 2;31(15):2689. doi: 10.3390/molecules31152689 (PMC13467625; doi:10.3390/molecules31152689)
Supplement: Supplementary file 1 [file molecules-31-02689-s001.zip › molecules-4447312-supplementary.pdf]

Supplementary Materials

Synthesis and characterization of new functionalized pyrimidine (hetero)cyclic molecular hybrids as chiral heterocyclic amino acid derivatives

Paulina Voznikaitė [1,2], Greta Račkauskienė [1]*, Miglė Dagilienė [1], Vilija Kederienė [2], Frank A. Sløk [3] and Algirdas Šačkus [1,2]*

[1] Institute of Synthetic Chemistry, Kaunas University of Technology, K. Baršausko g. 59, LT-51423 Kaunas, Lithuania; paulina.voznikaite@ktu.lt (P.V.); migle.burinskaite@ktu.lt (M.D.);

[2] Department of Organic Chemistry, Kaunas University of Technology, Radvilėnų pl. 19, LT-50254 Kaunas, Lithuania; vilija.kederiene@ktu.lt

[3] Vipergen ApS, Gammel Kongevej 23A, V DK-1610 Copenhagen, Denmark; fas@vipergen.com

* Correspondence: greta.ragaite@ktu.lt (G.R.); algirdas.sackus@ktu.lt (A.Š.); Tel.: +370-37-451-401 (A.Š.)

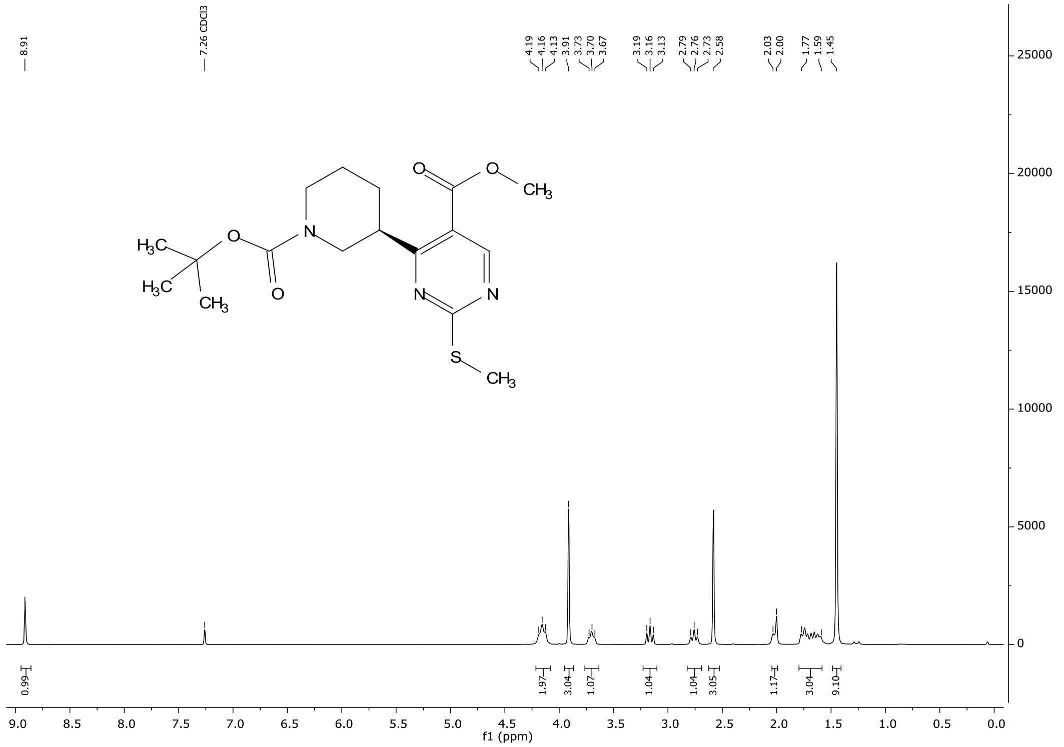

Figure S1. ^{1}H NMR (400 MHz, $CDCl_3$) spectrum of methyl 4-[(3*R*)-1-(*tert*-butoxycarbonyl)piperidin-3-yl]-2-(methylsulfanyl)pyrimidine-5-carboxylate (**4a**)

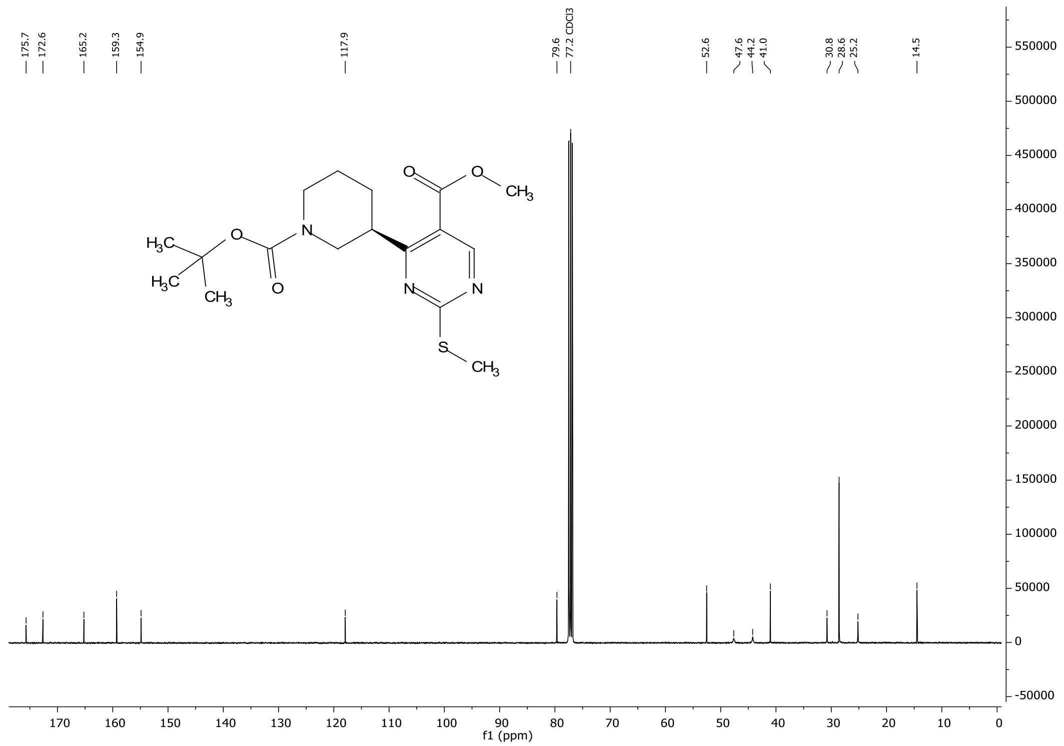

Figure S2. ^{13}C NMR (101 MHz, $CDCl_3$) spectrum of methyl 4-[(3*R*)-1-(*tert*-butoxycarbonyl)piperidin-3-yl]-2-(methylsulfanyl)pyrimidine-5-carboxylate (**4a**)

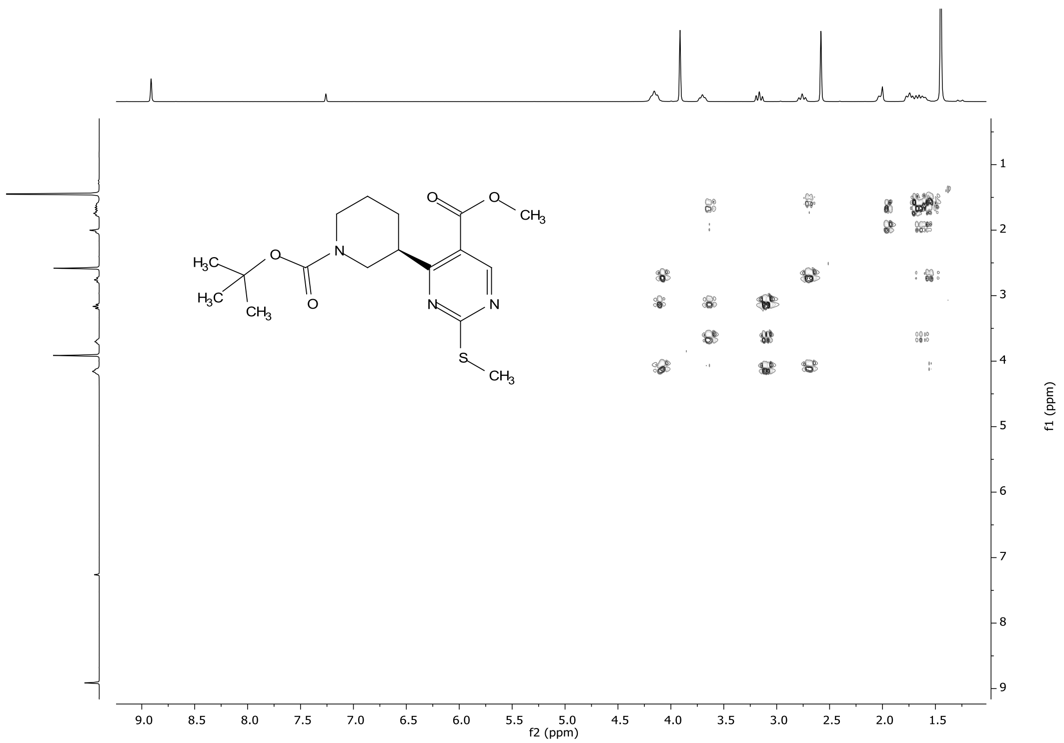

Figure S3. $^1H–^1H$ COSY NMR (400 MHz, $CDCl_3$) spectrum of methyl 4-[(3*R*)-1-(*tert*-butoxycarbonyl)piperidin-3-yl]-2-(methylsulfanyl)pyrimidine-5-carboxylate (**4a**)

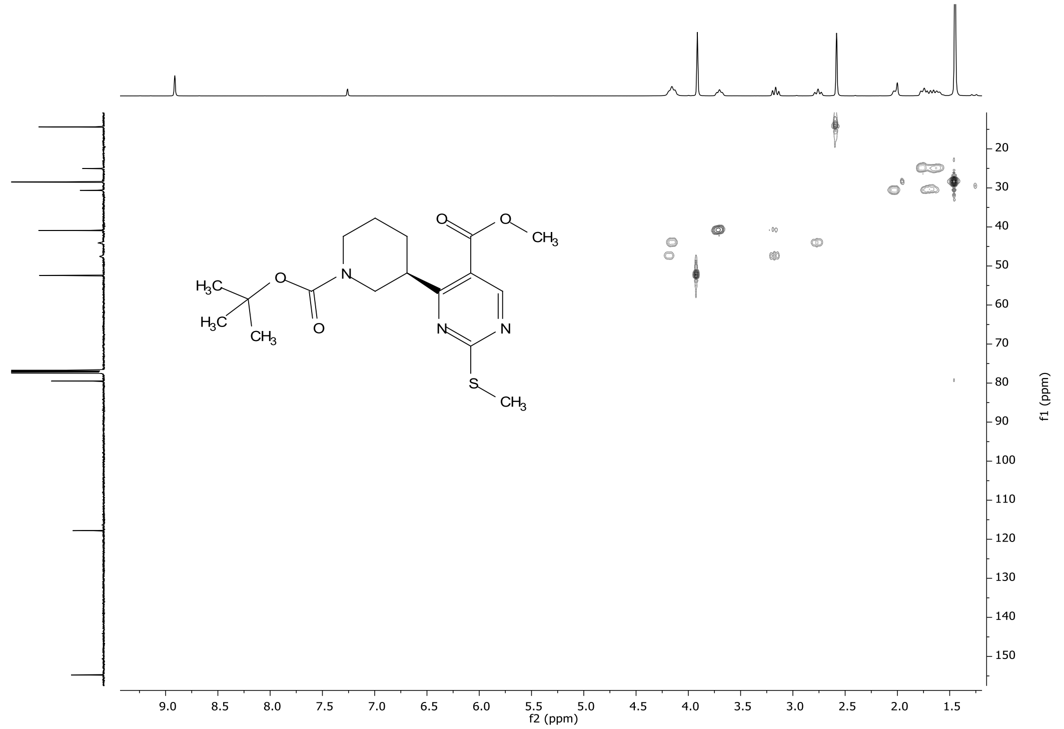

Figure S4. $^1H–^{13}C$ HSQC NMR (400/101 MHz, $CDCl_3$) spectrum of methyl 4-[(3*R*)-1-(*tert*-butoxycarbonyl)piperidin-3-yl]-2-(methylsulfanyl)pyrimidine-5-carboxylate (**4a**)

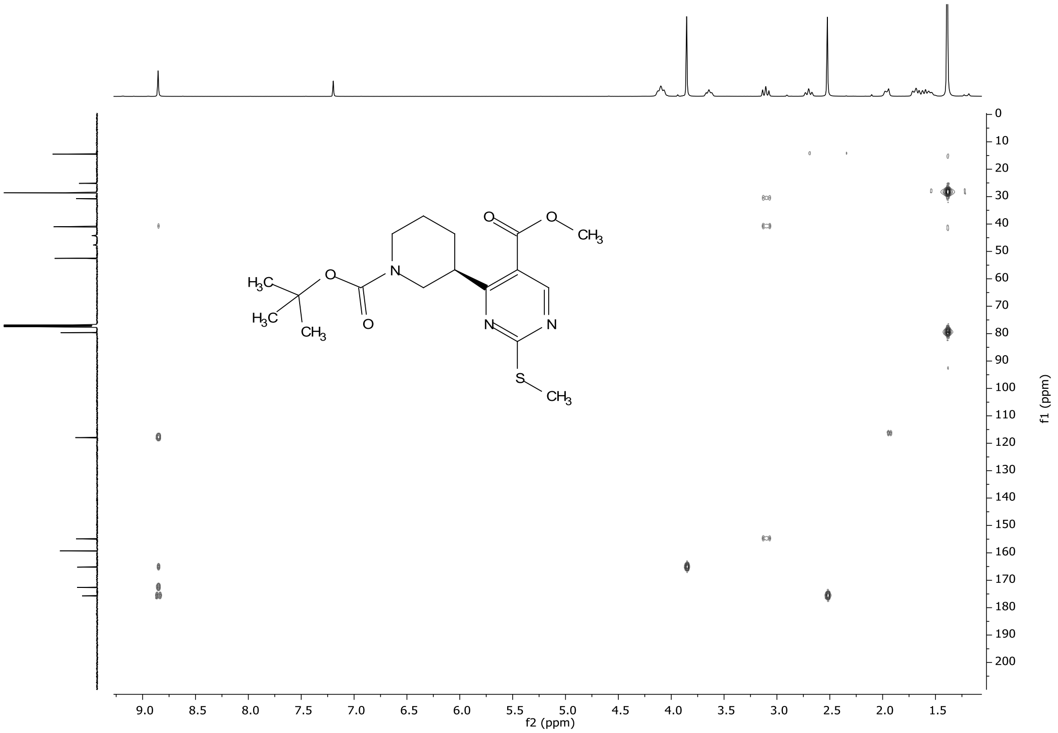

Figure S5. ^{1}H–^{13}C HMBC NMR (400/101 MHz, $CDCl_3$) spectrum of methyl 4-[(3*R*)-1-(*tert*-butoxycarbonyl)piperidin-3-yl]-2-(methylsulfanyl)pyrimidine-5-carboxylate (**4a**)

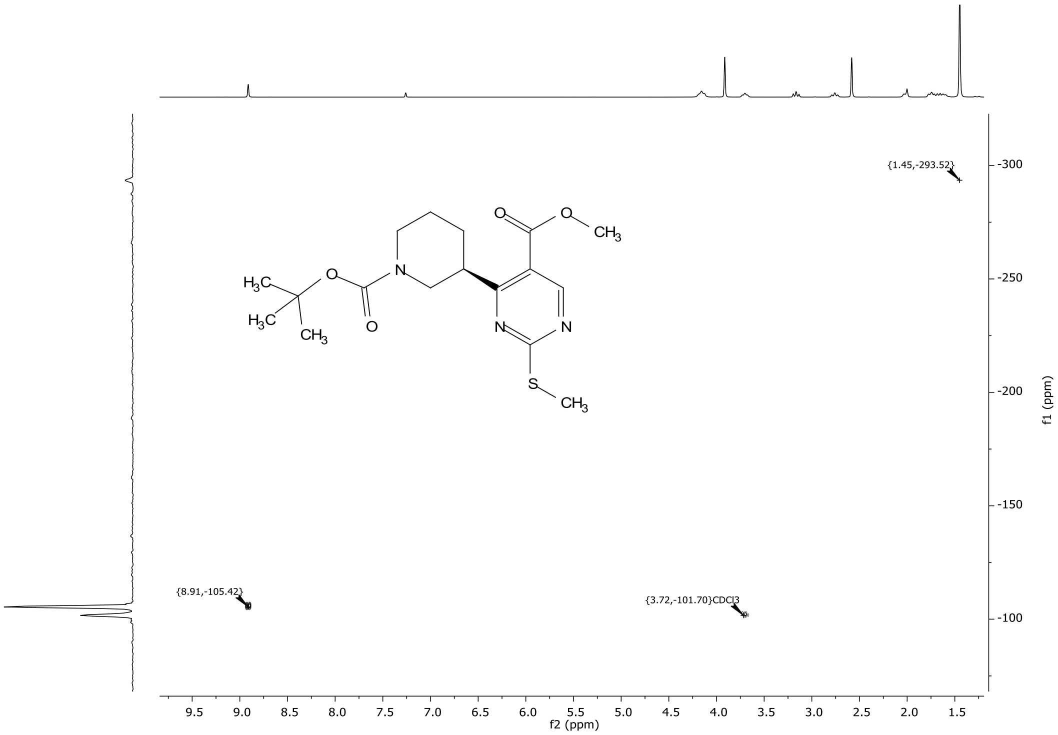

Figure S6. ^{1}H–^{15}N HMBC NMR (400/71 MHz, $CDCl_3$) spectrum of methyl 4-[(3*R*)-1-(*tert*-butoxycarbonyl)piperidin-3-yl]-2-(methylsulfanyl)pyrimidine-5-carboxylate (**4a**)

Compound Spectrum SmartFormula Report

Analysis Info

Analysis Name	D:\Data\PVP-809.d	Acquisition Date	3/18/2026 2:50:10 PM
Method	DirectInfusion_TuneLow_pos.m	Operator	hplc
Sample Name	PVP-809	Instrument	micrOTOF-Q III 8228888.20448
Comment	SB		

Acquisition Parameter

Source Type	ESI	Ion Polarity	Positive	Set Nebulizer	0.4 Bar
Focus	Not active	Set Capillary	4500 V	Set Dry Heater	180 °C
Scan Begin	50 m/z	Set End Plate Offset	-500 V	Set Dry Gas	4.0 l/min
Scan End	1000 m/z	Set Collision Cell RF	140.0 Vpp	Set Divert Valve	Waste

#	RT [min]	Area	Int. Type	I	S/N	Chromatogram	Max. m/z	FWHM [min]
n.a.	0.3	n.a.	Single spectrum	n.a.	n.a.	n.a.	304.2617	n.a.
n.a.	4.2	n.a.	Single spectrum	n.a.	n.a.	n.a.	368.1640	n.a.

+MS, 4.2min #249

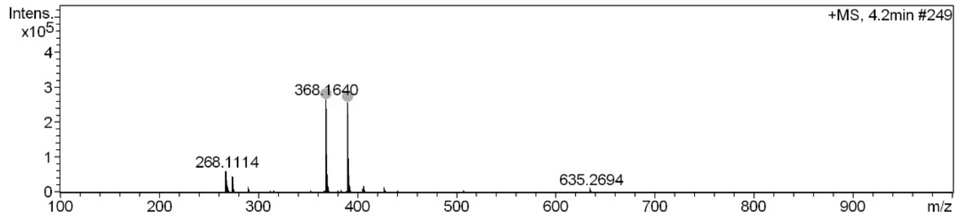

Meas. m/z	#	Ion Formula	m/z	err [ppm]	mSigma	# Sigma	Score	rdb	e⁻ Conf	N-Rule
368.1640	1	C17H26N3O4S	368.1639	-0.4	3.8	1	100.00	6.5	even	ok
390.1459	1	C17H25N3NaO4S	390.1458	-0.2	4.6	1	100.00	6.5	even	ok

Figure S7. HRMS (ESI-TOF) spectrum of methyl 4-[(3*R*)-1-(*tert*-butoxycarbonyl)piperidin-3-yl]-2-(methylsulfanyl)pyrimidine-5-carboxylate (**4a**)

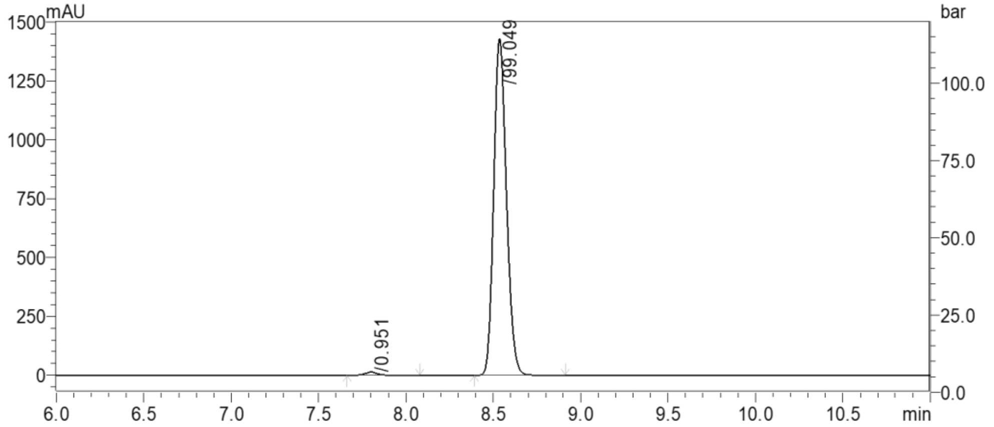

Figure S8. Chiral HPLC analysis of methyl 4-[(3*R*)-1-(*tert*-butoxycarbonyl)piperidin-3-yl]-2-(methylsulfanyl)pyrimidine-5-carboxylate (**4a**). Conditions: CHIRAL ART Cellulose-SJ (100 × 4.6 mm I.D.); S-10 μL; mobile phase: ACN/H_2O + 0.1% HCOOH (70:30→30:70 in 10 min); T = 36 ˚C; flow rate 1.0 mL/min.

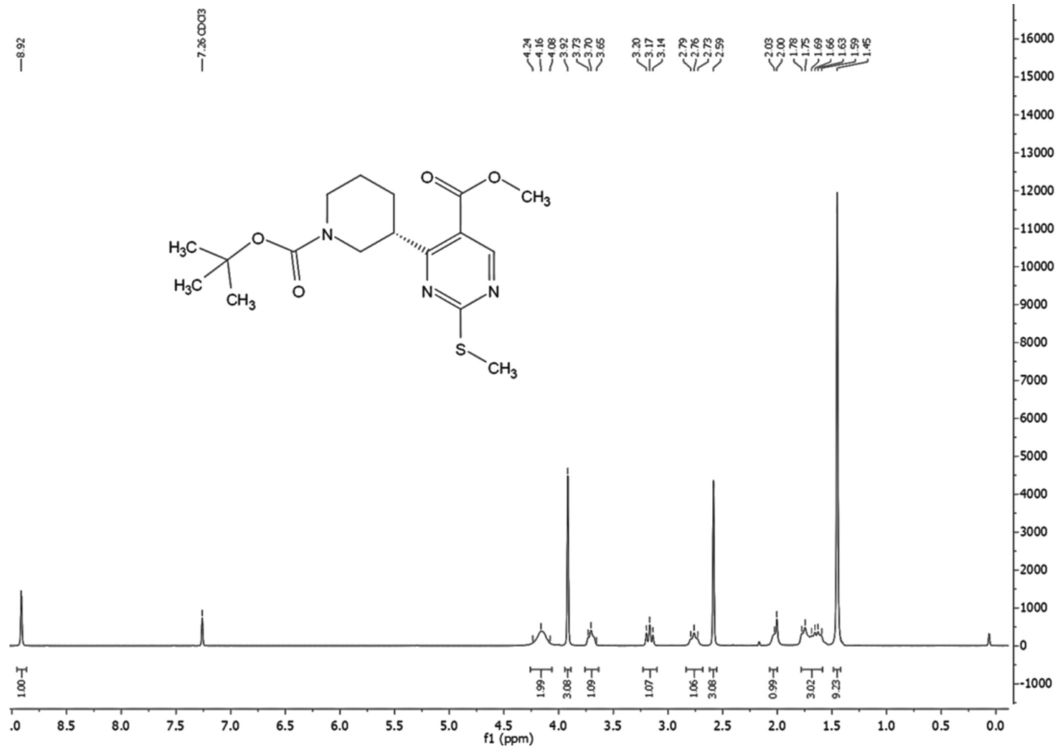

Figure S9. ^{1}H NMR (400 MHz, $CDCl_3$) spectrum of methyl 4-[(3*S*)-1-(*tert*-butoxycarbonyl)piperidin-3-yl]-2-(methylsulfanyl)pyrimidine-5-carboxylate (**4b**)

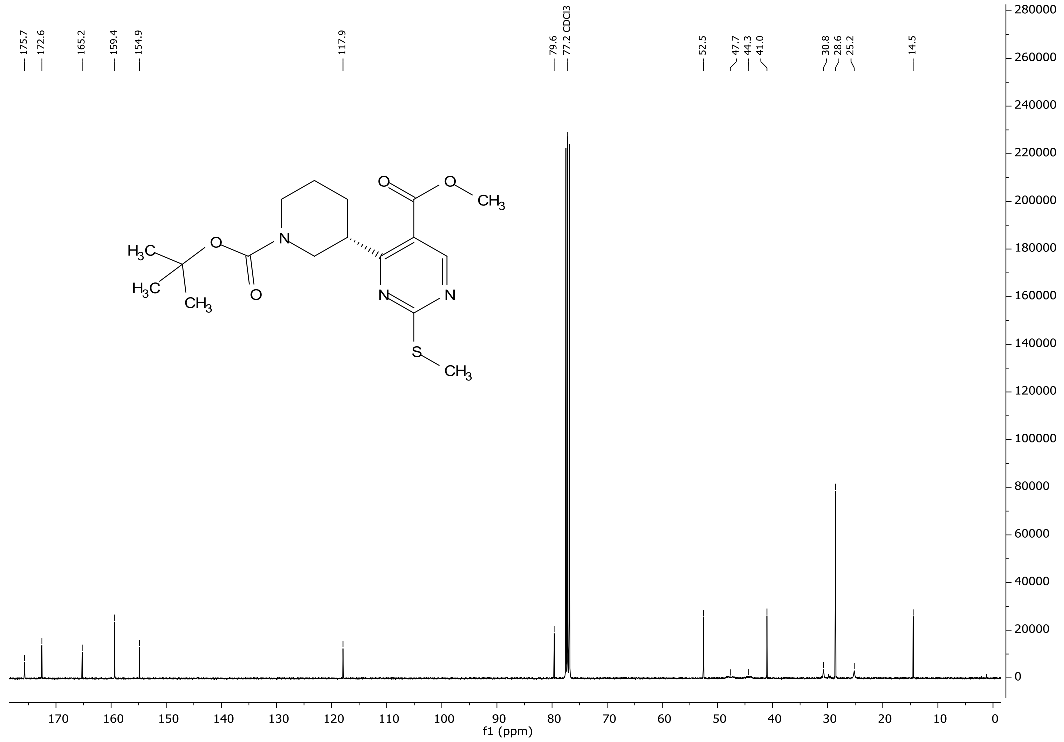

Figure S10. ^{13}C NMR (101 MHz, $CDCl_3$) spectrum of methyl 4-[(3*S*)-1-(*tert*-butoxycarbonyl)piperidin-3-yl]-2-(methylsulfanyl)pyrimidine-5-carboxylate (**4b**)

Compound Spectrum SmartFormula Report

Analysis Info

Analysis Name	D:\Data\PVP-812.d	Acquisition Date	3/26/2026 11:46:01 AM
Method	DirectInfusion_TuneLow_pos.m	Operator	hplc
Sample Name	PVP-812	Instrument	micrOTOF-Q III 8228888.20448
Comment	SB		

Acquisition Parameter

Source Type	ESI	Ion Polarity	Positive	Set Nebulizer	0.4 Bar
Focus	Not active	Set Capillary	4500 V	Set Dry Heater	180 °C
Scan Begin	50 m/z	Set End Plate Offset	-500 V	Set Dry Gas	4.0 l/min
Scan End	1000 m/z	Set Collision Cell RF	140.0 Vpp	Set Divert Valve	Waste

#	RT [min]	Area	Int. Type	I	S/N	Chromatogram	Max. m/z	FWHM [min]
n.a.	0.3	n.a.	Single spectrum	n.a.	n.a.	n.a.	304.2610	n.a.
n.a.	5.3	n.a.	Single spectrum	n.a.	n.a.	n.a.	390.1458	n.a.

+MS, 5.3min #318

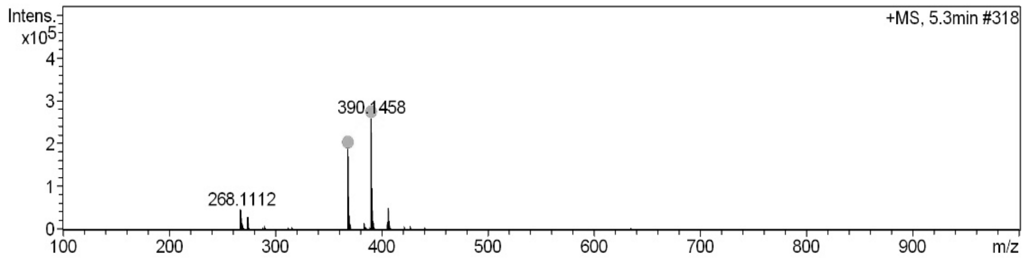

Meas. m/z	#	Ion Formula	m/z	err [ppm]	mSigma	# Sigma	Score	rdb	e⁻ Conf	N-Rule
368.1640	1	C17H26N3O4S	368.1639	0.4	4.9	1	100.00	6.5	even	ok
390.1458	1	C17H25N3NaO4S	390.1458	-0.1	2.2	1	100.00	6.5	even	ok

Figure S11. HRMS (ESI-TOF) spectrum of methyl 4-[(3*S*)-1-(*tert*-butoxycarbonyl)piperidin-3-yl]-2-(methylsulfanyl)pyrimidine-5-carboxylate (**4b**)

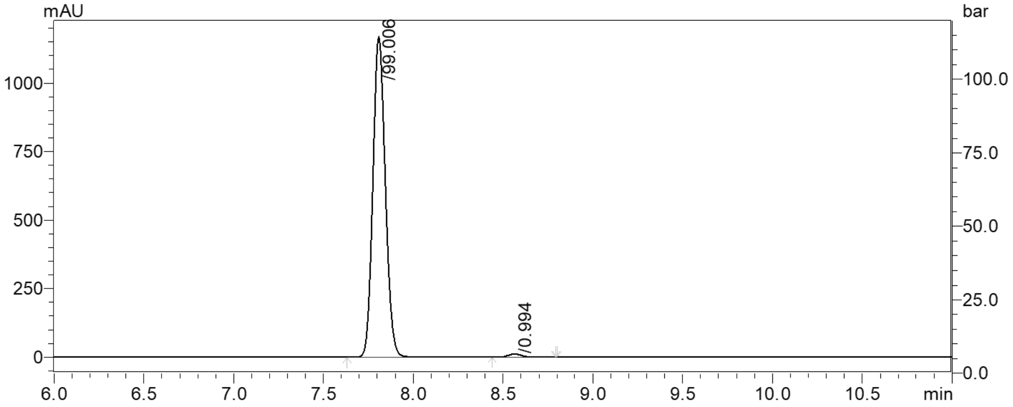

Figure S12. Chiral HPLC analysis of methyl 4-[(3*S*)-1-(*tert*-butoxycarbonyl)piperidin-3-yl]-2-(methylsulfanyl)pyrimidine-5-carboxylate (**4b**). Conditions: CHIRAL ART Cellulose-SJ (100 × 4.6 mm I.D.); S-10 μL; mobile phase: ACN/H_2O + 0.1% HCOOH (70:30→30:70 in 10 min); T = 36 ˚C; flow rate 1.0 mL/min.

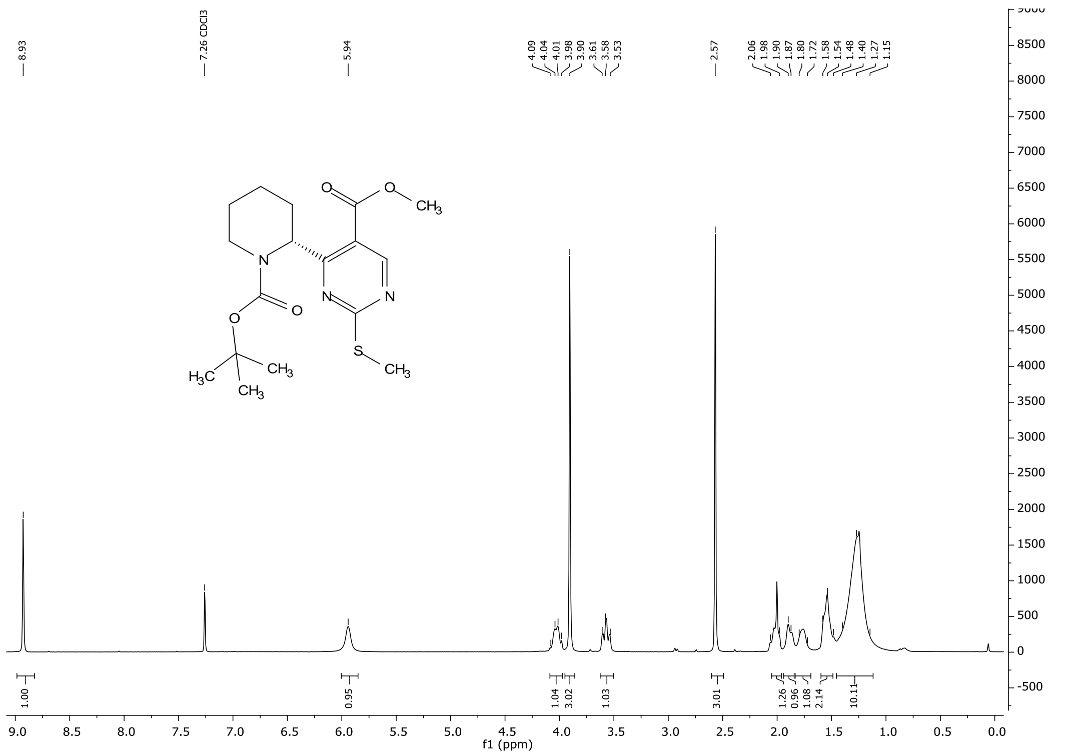

Figure S13. ^{1}H NMR (400 MHz, $CDCl_3$) spectrum of methyl 4-[(2*R*)-1-(*tert*-butoxycarbonyl)piperidin-2-yl]-2-(methylsulfanyl)pyrimidine-5-carboxylate (**4c**)

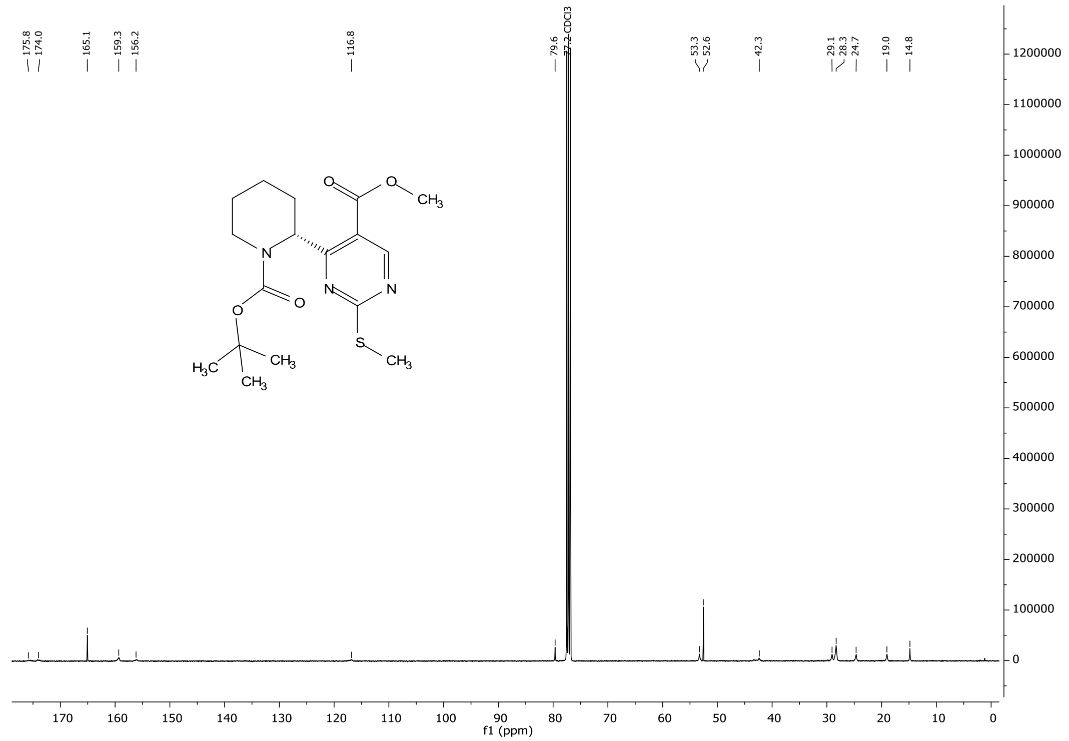

Figure S14. ^{13}C NMR (101 MHz, $CDCl_3$) spectrum of methyl 4-[(2*R*)-1-(*tert*-butoxycarbonyl)piperidin-2-yl]-2-(methylsulfanyl)pyrimidine-5-carboxylate (**4c**)

Compound Spectrum SmartFormula Report

Analysis Info

Analysis Name	D:\Data\GR-P-3094.d	Acquisition Date	3/17/2026 12:21:46 PM
Method	DirectInfusion_TuneLow_pos.m	Operator	hplc
Sample Name	GR-P-3094	Instrument	micrOTOF-Q III 8228888.20448
Comment	SB		

Acquisition Parameter

Source Type	ESI	Ion Polarity	Positive	Set Nebulizer	0.4 Bar
Focus	Not active	Set Capillary	4500 V	Set Dry Heater	180 °C
Scan Begin	50 m/z	Set End Plate Offset	-500 V	Set Dry Gas	4.0 l/min
Scan End	1000 m/z	Set Collision Cell RF	140.0 Vpp	Set Divert Valve	Waste

#	RT [min]	Area	Int. Type	I	S/N	Chromatogram	Max. m/z	FWHM [min]
n.a.	0.1	n.a.	Single spectrum	n.a.	n.a.	n.a.	304.2618	n.a.
n.a.	5.2	n.a.	Single spectrum	n.a.	n.a.	n.a.	390.1459	n.a.

+MS, 5.2min #309

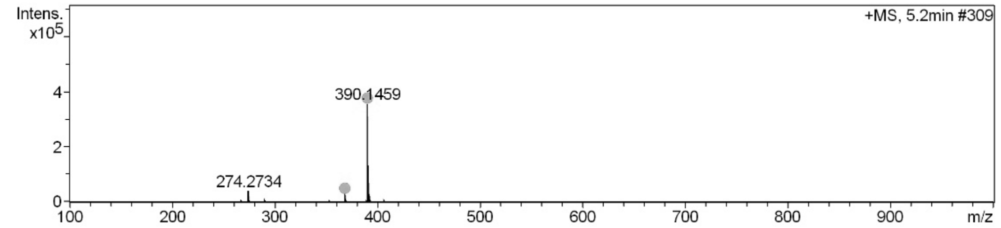

Meas. m/z	#	Ion Formula	m/z	err [ppm]	mSigma	# Sigma	Score	rdb	e⁻ Conf	N-Rule
368.1637	1	C17H26N3O4S	368.1639	-0.4	9.9	1	100.00	6.5	even	ok
390.1459	1	C17H25N3NaO4S	390.1458	-0.3	5.4	1	100.00	6.5	even	ok

Figure S15. HRMS (ESI-TOF) spectrum of methyl 4-[(2*R*)-1-(*tert*-butoxycarbonyl)piperidin-2-yl]-2-(methylsulfanyl)pyrimidine-5-carboxylate (**4c**)

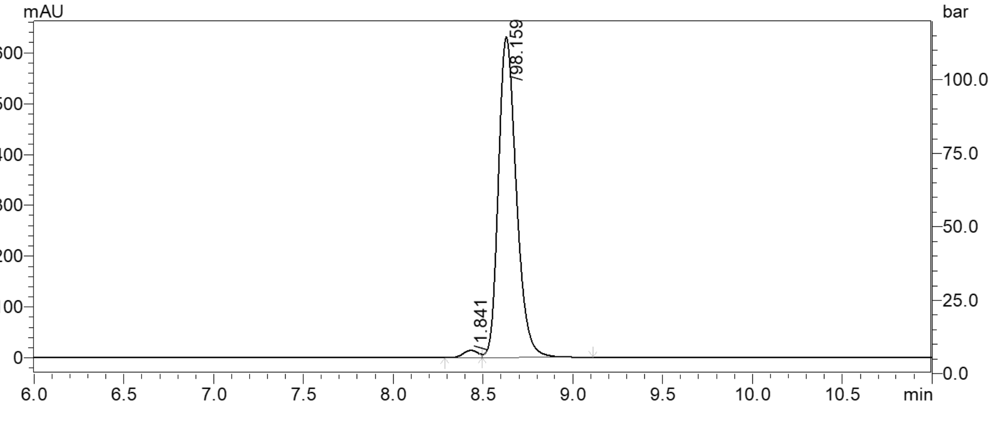

Figure S16. Chiral HPLC analysis of methyl 4-[(2*R*)-1-(*tert*-butoxycarbonyl)piperidin-2-yl]-2-(methylsulfanyl)pyrimidine-5-carboxylate (**4c**). Conditions: CHIRAL ART Amylose-SA (100 × 4.6 mm I.D.); S-10 μL; mobile phase: ACN/H_2O + 0.1% HCOOH (70:30→30:70 in 10 min); T = 36 °C; flow rate 1.0 mL/min.

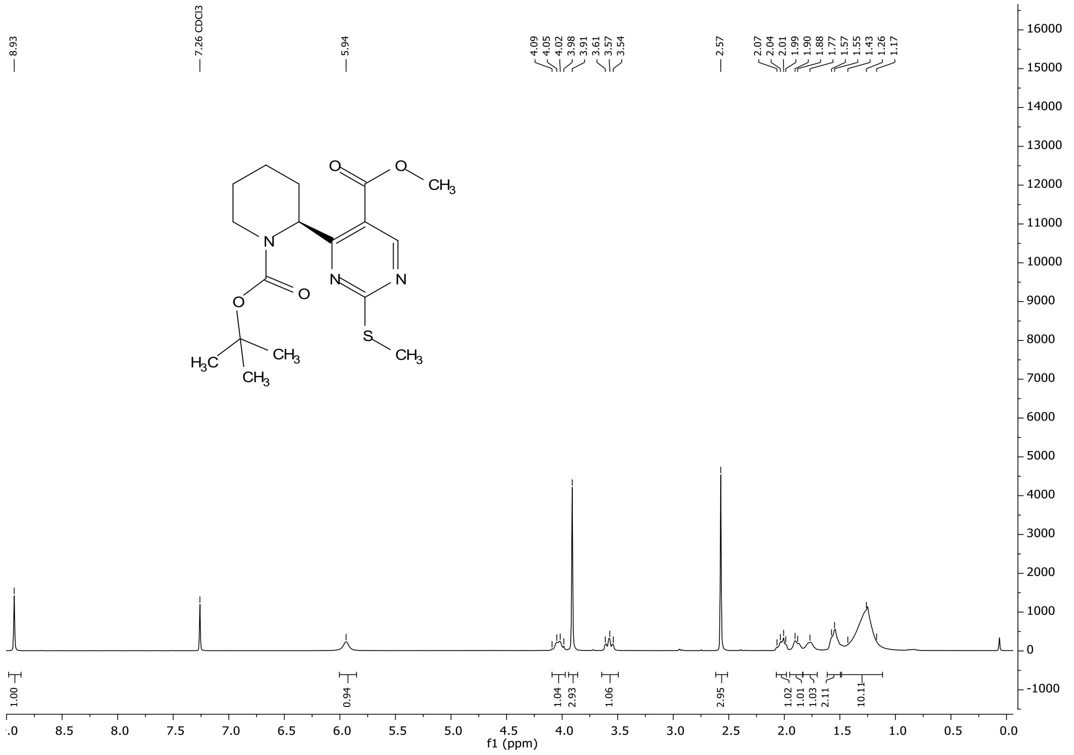

Figure S17. ^{1}H NMR (400 MHz, $CDCl_3$) spectrum of methyl 4-[(2*S*)-1-(*tert*-butoxycarbonyl)piperidin-2-yl]-2-(methylsulfanyl)pyrimidine-5-carboxylate (**4d**)

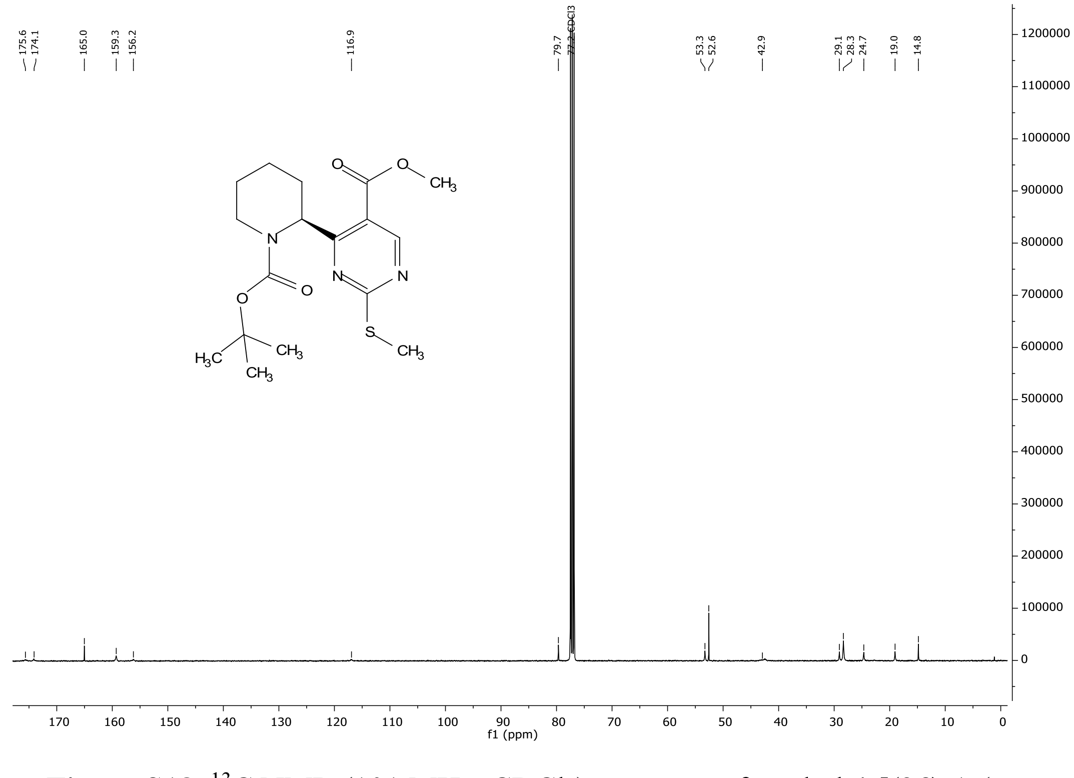

Figure S18. ^{13}C NMR (101 MHz, $CDCl_3$) spectrum of methyl 4-[(2*S*)-1-(*tert*-butoxycarbonyl)piperidin-2-yl]-2-(methylsulfanyl)pyrimidine-5-carboxylate (**4d**)

Compound Spectrum SmartFormula Report

Analysis Info

Analysis Name	D:\Data\GR-P-3093.d	Acquisition Date	3/10/2026 11:36:38 AM
Method	DirectInfusion_TuneLow_pos.m	Operator	hplc
Sample Name	GR-P-3093	Instrument	micrOTOF-Q III 8228888.20448
Comment	SB		

Acquisition Parameter

Source Type	ESI	Ion Polarity	Positive	Set Nebulizer	0.4 Bar
Focus	Not active	Set Capillary	4500 V	Set Dry Heater	180 °C
Scan Begin	50 m/z	Set End Plate Offset	-500 V	Set Dry Gas	4.0 l/min
Scan End	1000 m/z	Set Collision Cell RF	140.0 Vpp	Set Divert Valve	Waste

#	RT [min]	Area	Int. Type	I	S/N	Chromatogram	Max. m/z	FWHM [min]
n.a.	2.0	n.a.	Single spectrum	n.a.	n.a.	n.a.	304.2619	n.a.
n.a.	4.8	n.a.	Single spectrum	n.a.	n.a.	n.a.	368.1639	n.a.

+MS, 4.8min #285

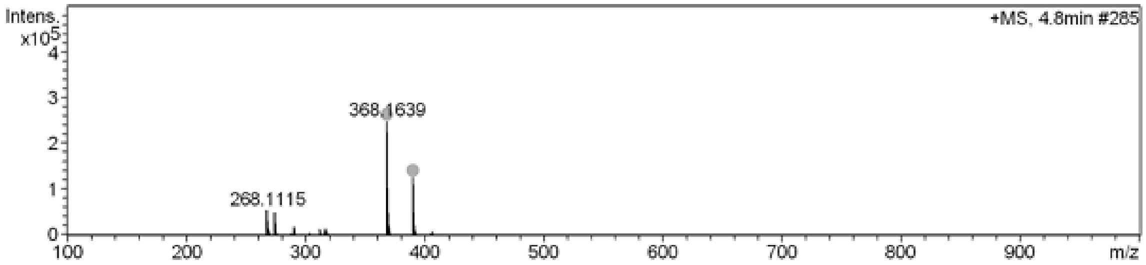

Meas. m/z	#	Ion Formula	m/z	err [ppm]	mSigma	# Sigma	Score	rdb	e⁻ Conf	N-Rule
368.1639	1	C17H26N3O4S	368.1639	0.1	1.7	1	100.00	6.5	even	ok
390.1461	1	C17H25N3NaO4S	390.1458	-0.7	1.8	1	100.00	6.5	even	ok

Figure S19. HRMS (ESI-TOF) spectrum of methyl 4-[(2*S*)-1-(*tert*-butoxycarbonyl)piperidin-2-yl]-2-(methylsulfanyl)pyrimidine-5-carboxylate (**4d**)

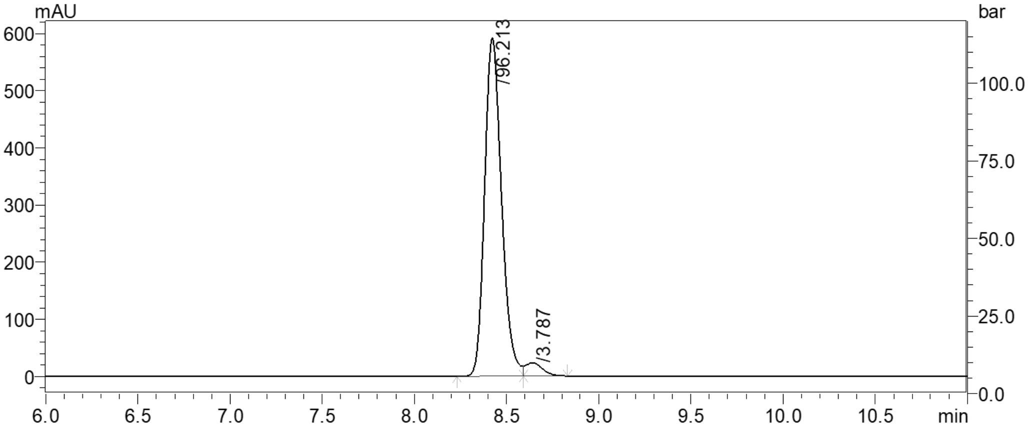

Figure S20. Chiral HPLC analysis of methyl 4-[(2*S*)-1-(*tert*-butoxycarbonyl)piperidin-2-yl]-2-(methylsulfanyl)pyrimidine-5-carboxylate (**4d**). Conditions: CHIRAL ART Amylose-SA (100 × 4.6 mm I.D.); S-10 µL; mobile phase: ACN/H_2O + 0.1% HCOOH (70:30→30:70 in 10 min); T = 36 °C; flow rate 1.0 mL/min.

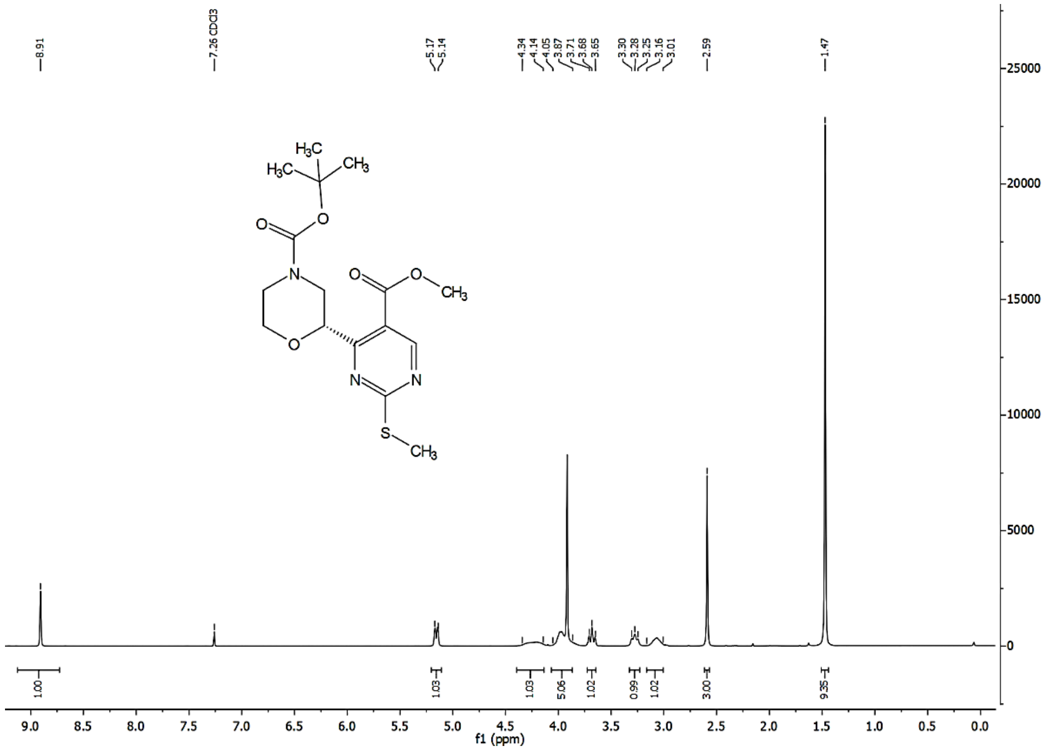

Figure S21. ^{1}H NMR (400 MHz, $CDCl_3$) spectrum of *tert*-butyl (2*R*)-2-[5-(methoxycarbonyl)-2-(methylsulfanyl)pyrimidin-4-yl]morpholine-4-carboxylate (**4e**)

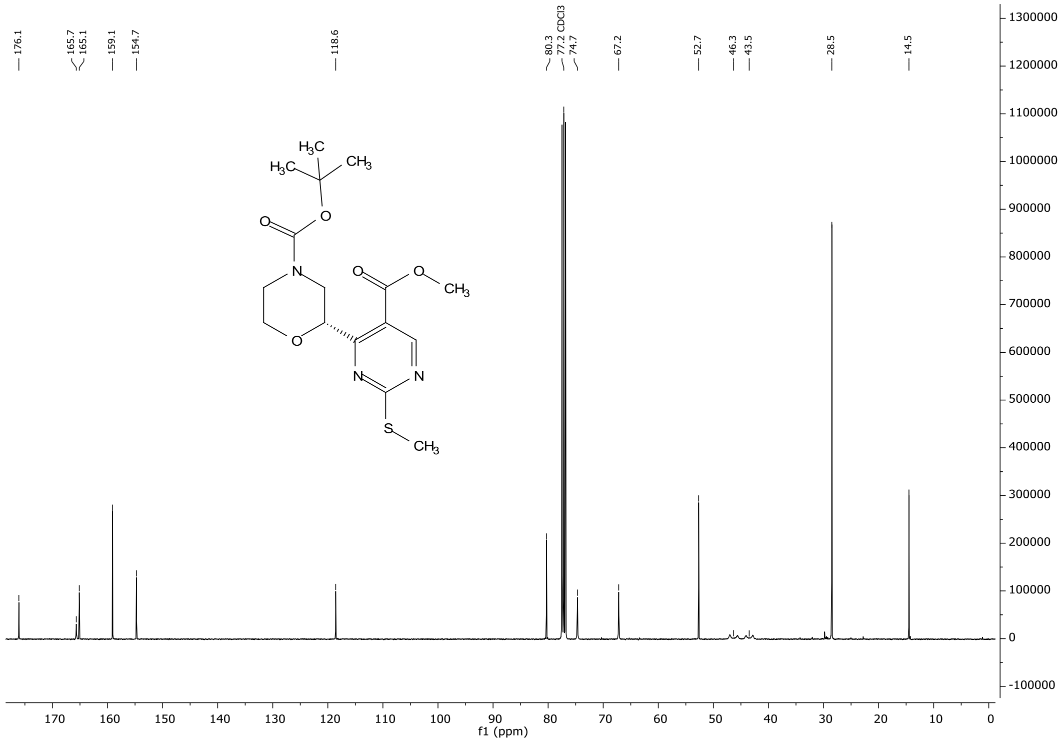

Figure S22. ^{13}C NMR (101 MHz, $CDCl_3$) spectrum of *tert*-butyl (2*R*)-2-[5-(methoxycarbonyl)-2-(methylsulfanyl)pyrimidin-4-yl]morpholine-4-carboxylate (**4e**)

Compound Spectrum SmartFormula Report

Analysis Info

Analysis Name	D:\Data\PVP-790.d	Acquisition Date	3/10/2026 12:13:00 PM
Method	DirectInfusion_TuneLow_pos.m	Operator	hplc
Sample Name	PVP-790	Instrument	micrOTOF-Q III 8228888.20448
Comment	SB		

Acquisition Parameter

Source Type	ESI	Ion Polarity	Positive	Set Nebulizer	0.4 Bar
Focus	Not active	Set Capillary	4500 V	Set Dry Heater	180 °C
Scan Begin	50 m/z	Set End Plate Offset	-500 V	Set Dry Gas	4.0 l/min
Scan End	1000 m/z	Set Collision Cell RF	140.0 Vpp	Set Divert Valve	Waste

#	RT [min]	Area	Int. Type	I	S/N	Chromatogram	Max. m/z	FWHM [min]
n.a.	0.1	n.a.	Single spectrum	n.a.	n.a.	n.a.	304.2615	n.a.
n.a.	3.9	n.a.	Single spectrum	n.a.	n.a.	n.a.	370.1431	n.a.

+MS, 3.9min #233

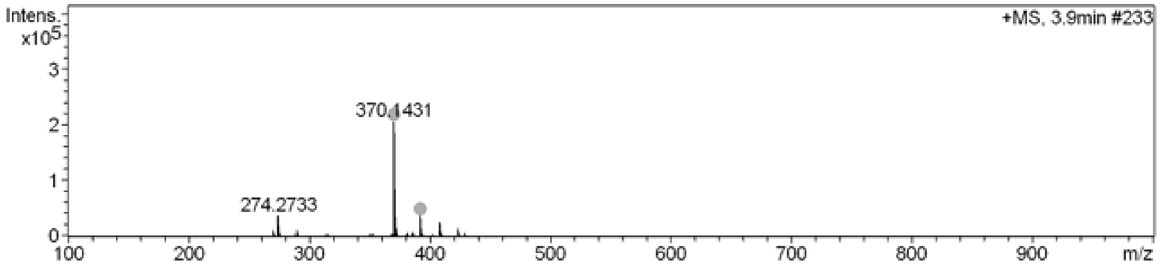

Meas. m/z	#	Ion Formula	m/z	err [ppm]	mSigma	# Sigma	Score	rdb	e⁻ Conf	N-Rule
370.1431	1	C16H24N3O5S	370.1431	0.0	2.4	1	100.00	6.5	even	ok
392.1252	1	C16H23N3NaO5S	392.1251	-0.4	1.7	1	100.00	6.5	even	ok

Figure S23. HRMS (ESI-TOF) spectrum of *tert*-butyl (2*R*)-2-[5-(methoxycarbonyl)-2-(methylsulfanyl)pyrimidin-4-yl]morpholine-4-carboxylate (**4e**)

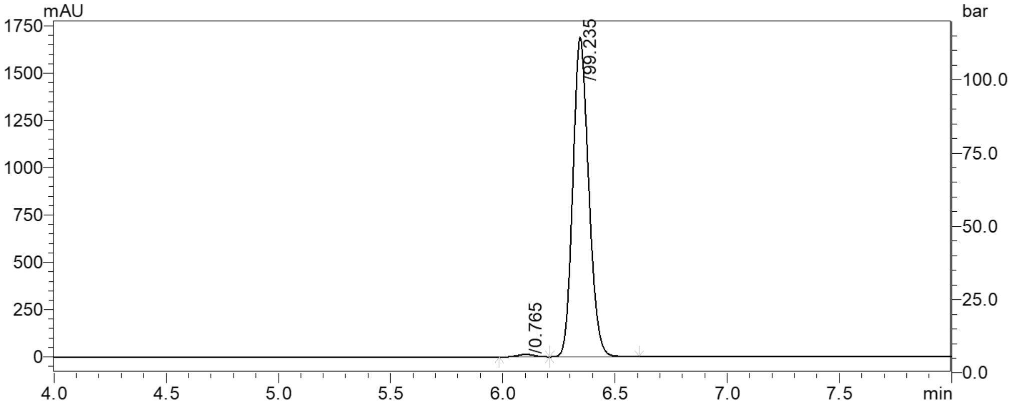

Figure S24. Chiral HPLC analysis of *tert*-butyl (2*R*)-2-[5-(methoxycarbonyl)-2-(methylsulfanyl)pyrimidin-4-yl]morpholine-4-carboxylate (**4e**). Conditions: CHIRAL ART Cellulose-SJ (100 × 4.6 mm I.D.); S-10 µL; mobile phase: ACN/H_2O + 0.1% HCOOH (70:30→30:70 in 10 min); T = 36 ˚C; flow rate 1.0 mL/min.

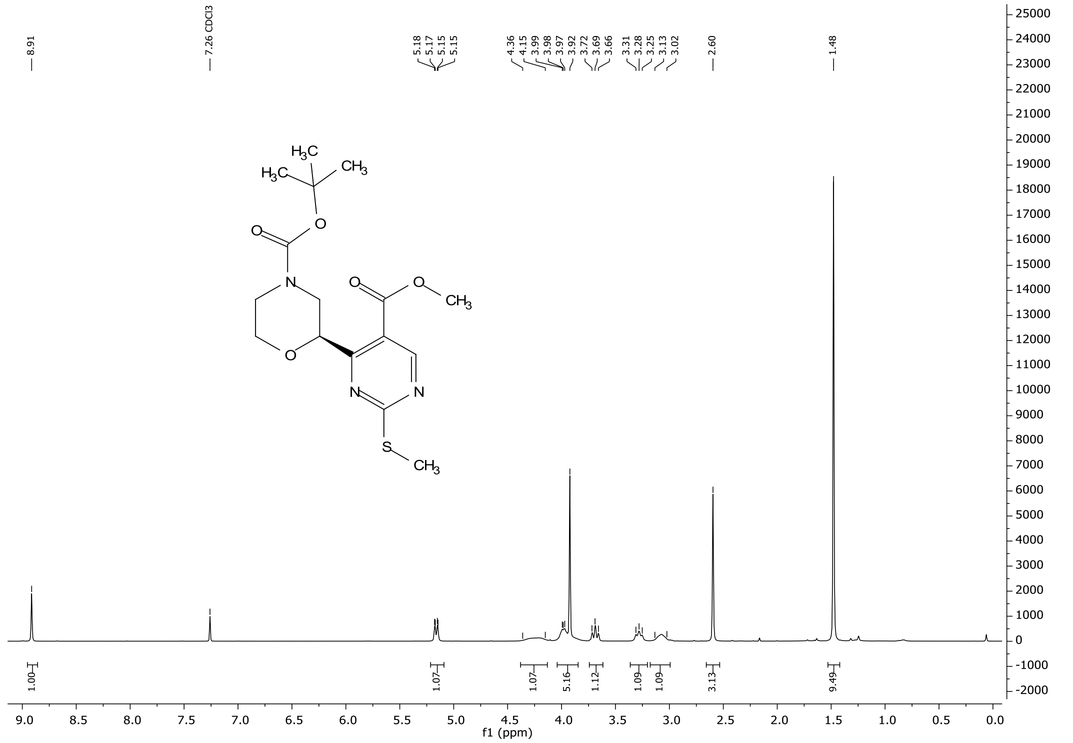

Figure S25. ^{1}H NMR (400 MHz, $CDCl_3$) spectrum of *tert*-butyl (2*S*)-2-[5-(methoxycarbonyl)-2-(methylsulfanyl)pyrimidin-4-yl]morpholine-4-carboxylate (**4f**)

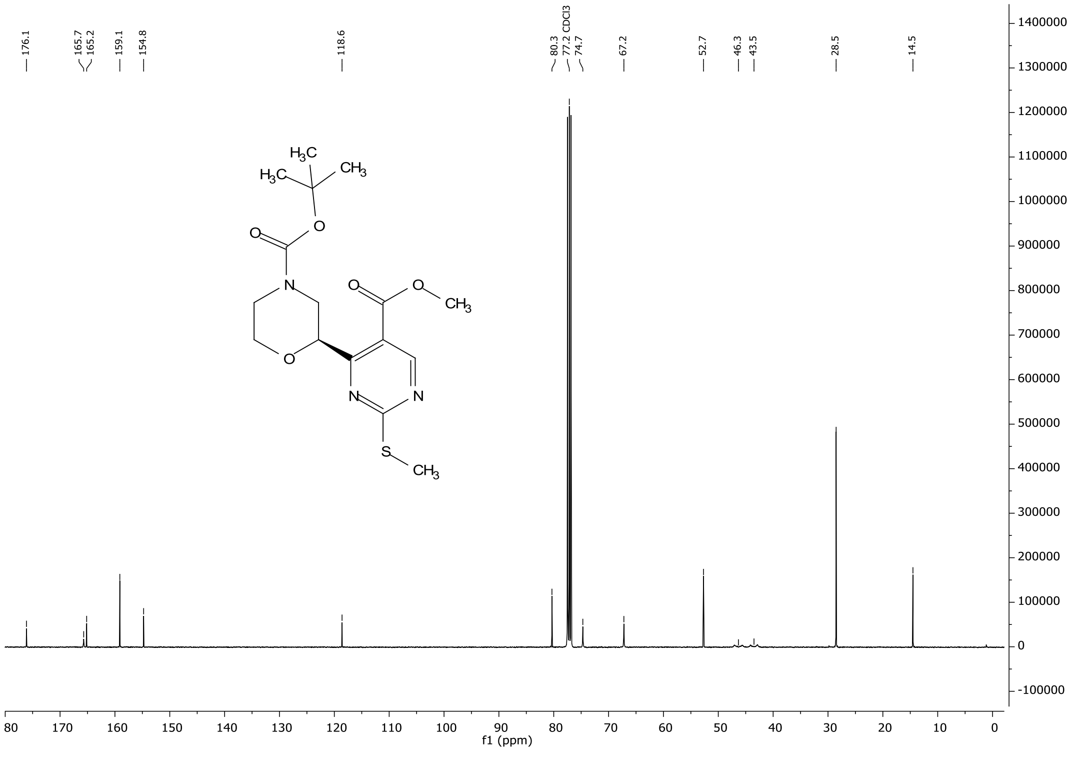

Figure S26. ^{13}C NMR (101 MHz, $CDCl_3$) spectrum of *tert*-butyl (2*S*)-2-[5-(methoxycarbonyl)-2-(methylsulfanyl)pyrimidin-4-yl]morpholine-4-carboxylate (**4f**)

Compound Spectrum SmartFormula Report

Analysis Info

Analysis Name	D:\Data\PVP-791.d	Acquisition Date	3/17/2026 11:43:10 AM
Method	DirectInfusion_TuneLow_pos.m	Operator	hplc
Sample Name	PVP-791	Instrument	micrOTOF-Q III 8228888.20448
Comment	SB		

Acquisition Parameter

Source Type	ESI	Ion Polarity	Positive	Set Nebulizer	0.4 Bar
Focus	Not active	Set Capillary	4500 V	Set Dry Heater	180 °C
Scan Begin	50 m/z	Set End Plate Offset	-500 V	Set Dry Gas	4.0 l/min
Scan End	1000 m/z	Set Collision Cell RF	140.0 Vpp	Set Divert Valve	Waste

#	RT [min]	Area	Int. Type	I	S/N	Chromatogram	Max. m/z	FWHM [min]
n.a.	1.3	n.a.	Single spectrum	n.a.	n.a.	n.a.	304.2620	n.a.
n.a.	3.6	n.a.	Single spectrum	n.a.	n.a.	n.a.	392.1250	n.a.

+MS, 3.6min #214

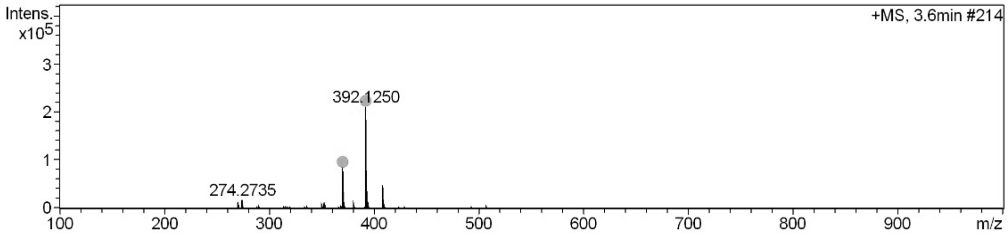

Meas. m/z	#	Ion Formula	m/z	err [ppm]	mSigma	# Sigma	Score	rdb	e⁻ Conf	N-Rule
370.1432	1	C16H24N3O5S	370.1431	-0.2	9.3	1	100.00	6.5	even	ok
392.1250	1	C16H23N3NaO5S	392.1251	0.2	1.3	1	100.00	6.5	even	ok

Figure S27. HRMS (ESI-TOF) spectrum of *tert*-butyl (2*S*)-2-[5-(methoxycarbonyl)-2-(methylsulfanyl)pyrimidin-4-yl]morpholine-4-carboxylate (**4f**)

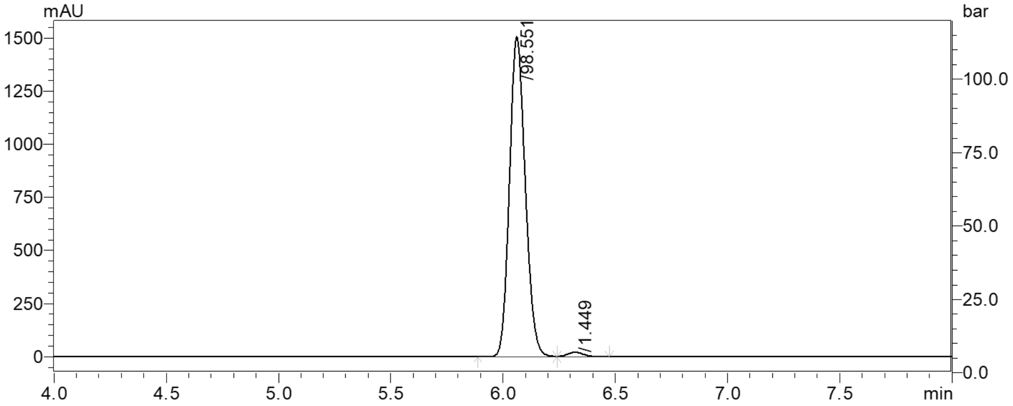

Figure S28. Chiral HPLC analysis of *tert*-butyl (2*S*)-2-[5-(methoxycarbonyl)-2-(methylsulfanyl)pyrimidin-4-yl]morpholine-4-carboxylate (**4f**). Conditions: CHIRAL ART Cellulose-SJ (100 × 4.6 mm I.D.); S-10 µL; mobile phase: ACN/H_2O + 0.1% HCOOH (70:30→30:70 in 10 min); T = 36 ˚C; flow rate 1.0 mL/min.

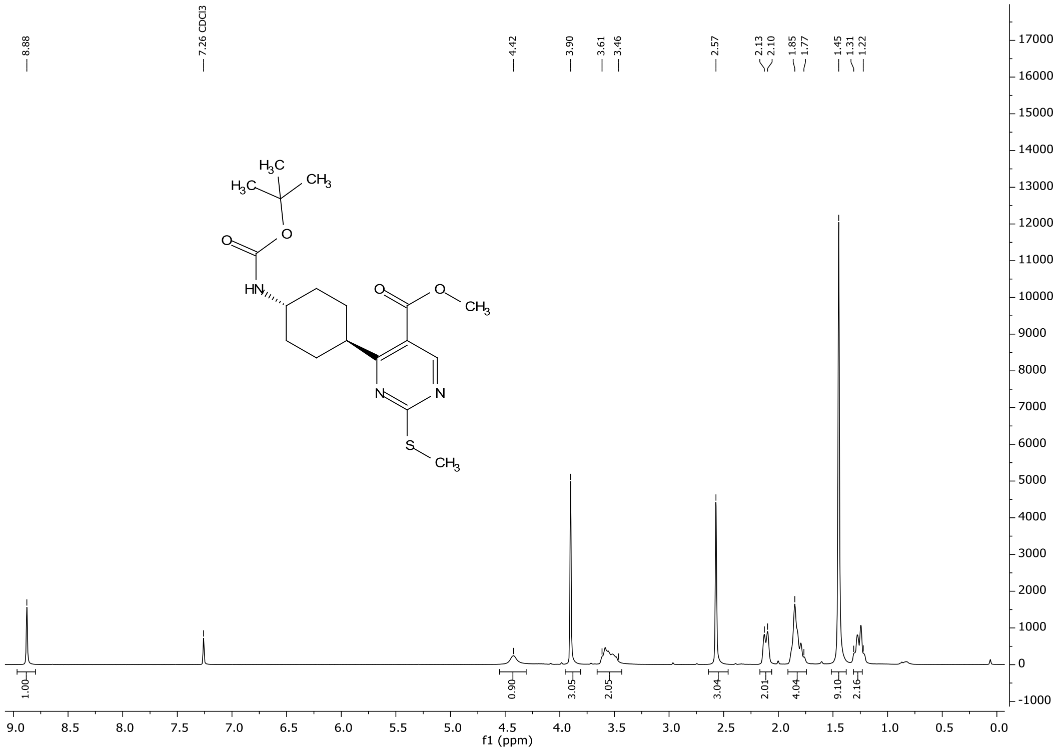

Figure S29. ^{1}H NMR (400 MHz, $CDCl_3$) spectrum of methyl 4-{*trans*-4-[(*tert*-butoxycarbonyl)amino]cyclohexyl}-2-(methylsulfanyl)pyrimidine-5-carboxylate (**4g**)

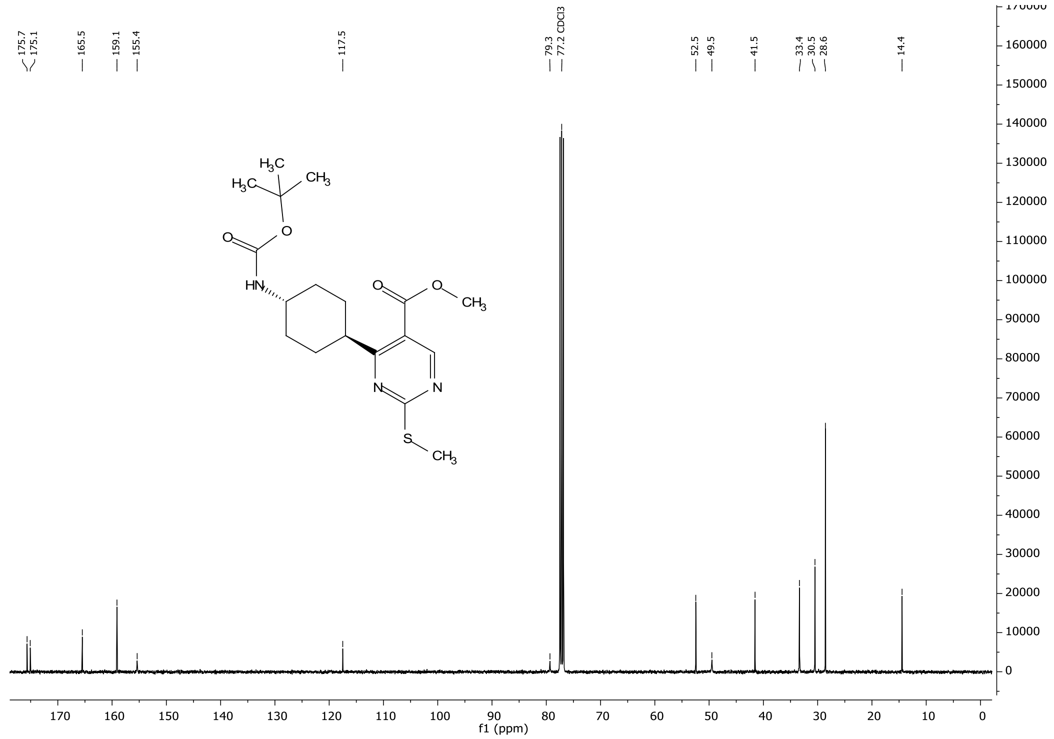

Figure S30. ^{13}C NMR (101 MHz, $CDCl_3$) spectrum of methyl 4-{*trans*-4-[(*tert*-butoxycarbonyl)amino]cyclohexyl}-2-(methylsulfanyl)pyrimidine-5-carboxylate (**4g**)

Compound Spectrum SmartFormula Report

Analysis Info

Analysis Name	D:\Data\PVP-787.d	Acquisition Date	3/19/2026 10:21:43 AM
Method	DirectInfusion_TuneLow_pos.m	Operator	hplc
Sample Name	PVP-787	Instrument	micrOTOF-Q III 8228888.20448
Comment	SB		

Acquisition Parameter

Source Type	ESI	Ion Polarity	Positive	Set Nebulizer	0.4 Bar		
Focus	Not active	Set Capillary	4500 V	Set Dry Heater	180 °C		
Scan Begin	50 m/z	Set End Plate Offset	-500 V	Set Dry Gas	4.0 l/min		
Scan End	1000 m/z	Set Collision Cell RF	140.0 Vpp	Set Divert Valve	Waste		

#	RT [min]	Area	Int. Type	I	S/N	Chromatogram	Max. m/z	FWHM [min]
n.a.	0.1	n.a.	Single spectrum	n.a.	n.a.	n.a.	304.2622	n.a.
n.a.	4.3	n.a.	Single spectrum	n.a.	n.a.	n.a.	404.1614	n.a.

+MS, 4.3min #257

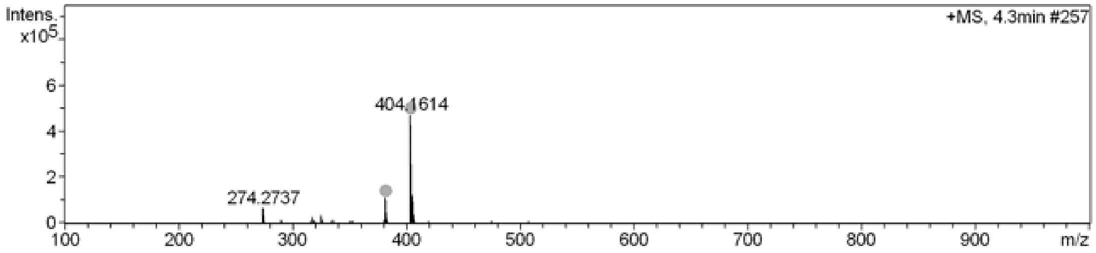

Meas. m/z	#	Ion Formula	m/z	err [ppm]	mSigma	# Sigma	Score	rdb	e⁻ Conf	N-Rule
382.1795	1	C18H28N3O4S	382.1795	0.0	5.3	1	100.00	6.5	even	ok
404.1614	1	C18H27N3NaO4S	404.1614	-0.1	8.3	1	100.00	6.5	even	ok

Figure S31. HRMS (ESI-TOF) spectrum of methyl 4-{*trans*-4-[(*tert*-butoxycarbonyl)amino]cyclohexyl}-2-(methylsulfanyl)pyrimidine-5-carboxylate (**4g**)

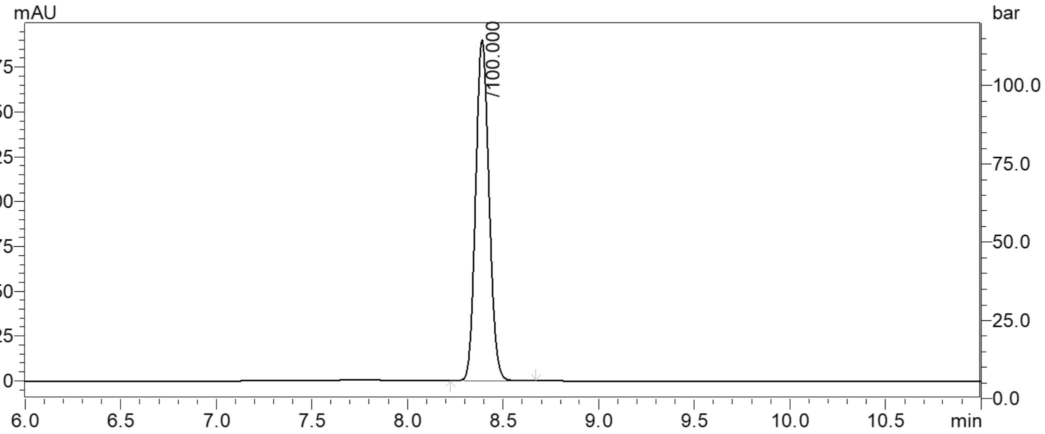

Figure S32. Chiral HPLC analysis of methyl 4-{*trans*-4-[(*tert*-butoxycarbonyl)amino]cyclohexyl}-2-(methylsulfanyl)pyrimidine-5-carboxylate (**4g**). Conditions: CHIRAL ART Cellulose-SJ (100 × 4.6 mm I.D.); S-10 µL; mobile phase: ACN/H_2O + 0.1% HCOOH (70:30→30:70 in 10 min); T = 36 ˚C; flow rate 1.0 mL/min.

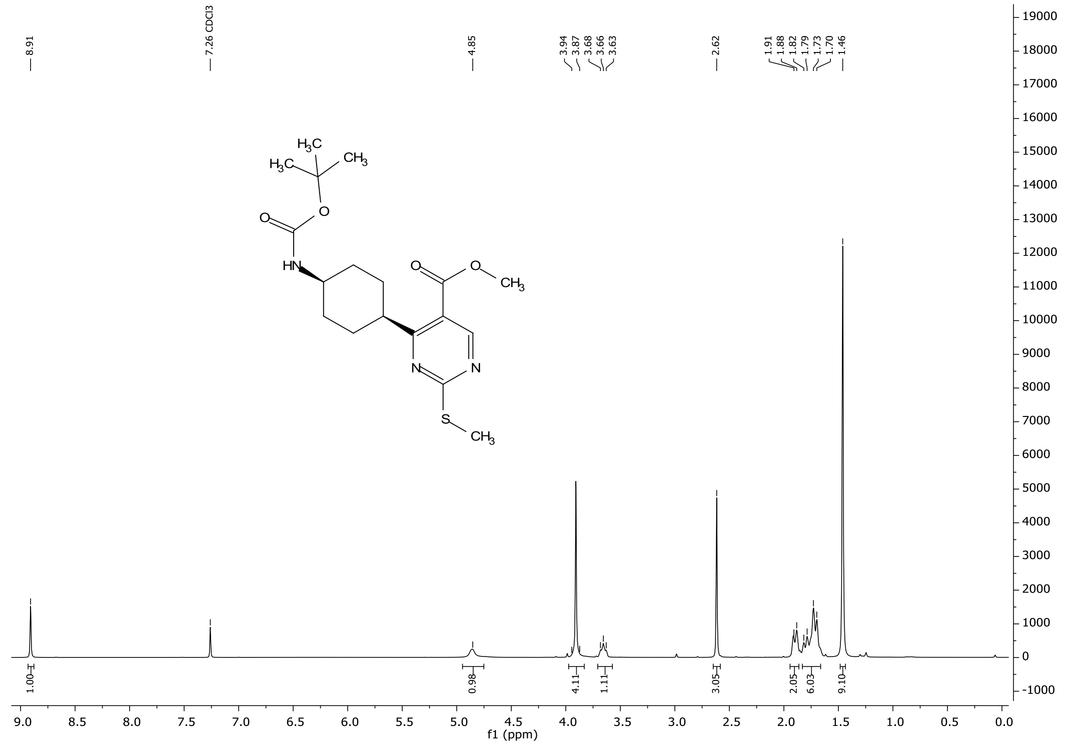

Figure S33. ^{1}H NMR (400 MHz, $CDCl_3$) spectrum of methyl 4-{*cis*-4-[(*tert*-butoxycarbonyl)amino]cyclohexyl}-2-(methylsulfanyl)pyrimidine-5-carboxylate (**4h**)

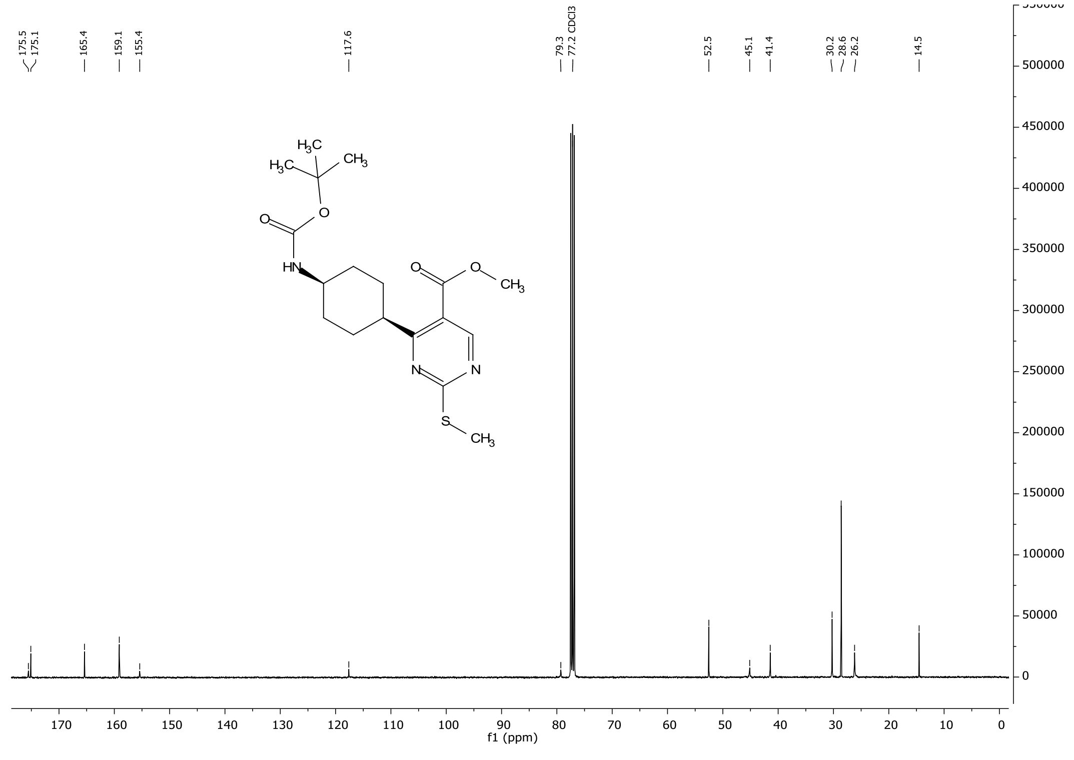

Figure S34. ^{13}C NMR (101 MHz, $CDCl_3$) spectrum of methyl 4-{*cis*-4-[(*tert*-butoxycarbonyl)amino]cyclohexyl}-2-(methylsulfanyl)pyrimidine-5-carboxylate (**4h**)

Compound Spectrum SmartFormula Report

Analysis Info

Analysis Name	D:\Data\PVP-786.d	Acquisition Date	3/30/2026 10:29:07 AM
Method	DirectInfusion_TuneLow_pos.m	Operator	hplc
Sample Name	PVP-786	Instrument	micrOTOF-Q III 8228888.20448
Comment	SB		

Acquisition Parameter

Source Type	ESI	Ion Polarity	Positive	Set Nebulizer	0.4 Bar
Focus	Not active	Set Capillary	4500 V	Set Dry Heater	180 °C
Scan Begin	50 m/z	Set End Plate Offset	-500 V	Set Dry Gas	4.0 l/min
Scan End	1000 m/z	Set Collision Cell RF	140.0 Vpp	Set Divert Valve	Waste

#	RT [min]	Area	Int. Type	I	S/N	Chromatogram	Max. m/z	FWHM [min]
n.a.	0.2	n.a.	Single spectrum	n.a.	n.a.	n.a.	304.2617	n.a.
n.a.	4.3	n.a.	Single spectrum	n.a.	n.a.	n.a.	404.1614	n.a.

+MS, 4.3min #255

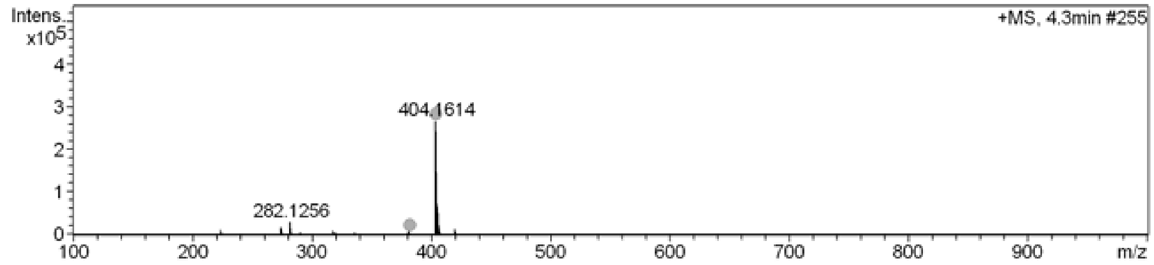

Meas. m/z	#	Ion Formula	m/z	err [ppm]	mSigma	# Sigma	Score	rdb	e⁻ Conf	N-Rule
382.1781	1	C18H28N3O4S	382.1795	-3.7	12.5	1	100.00	6.5	even	ok
404.1614	1	C18H27N3NaO4S	404.1614	0.1	1.9	1	100.00	6.5	even	ok

Figure S35. HRMS (ESI-TOF) spectrum of methyl 4-{*cis*-4-[(*tert*-butoxycarbonyl)amino]cyclohexyl}-2-(methylsulfanyl)pyrimidine-5-carboxylate (**4h**)

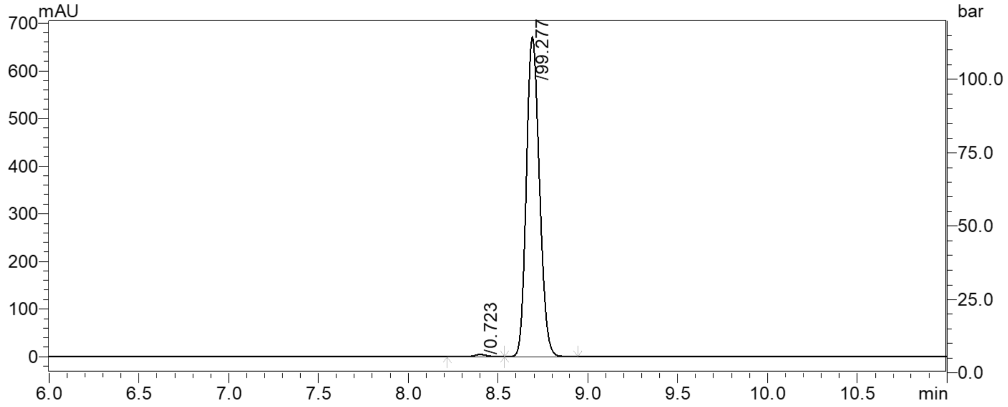

Figure S36. Chiral HPLC analysis of methyl 4-{*cis*-4-[(*tert*-butoxycarbonyl)amino]cyclohexyl}-2-(methylsulfanyl)pyrimidine-5-carboxylate (**4h**). Conditions: CHIRAL ART Cellulose-SJ (100 × 4.6 mm I.D.); S-10 µL; mobile phase: ACN/H_2O + 0.1% HCOOH (70:30→30:70 in 10 min); T = 36 ˚C; flow rate 1.0 mL/min.

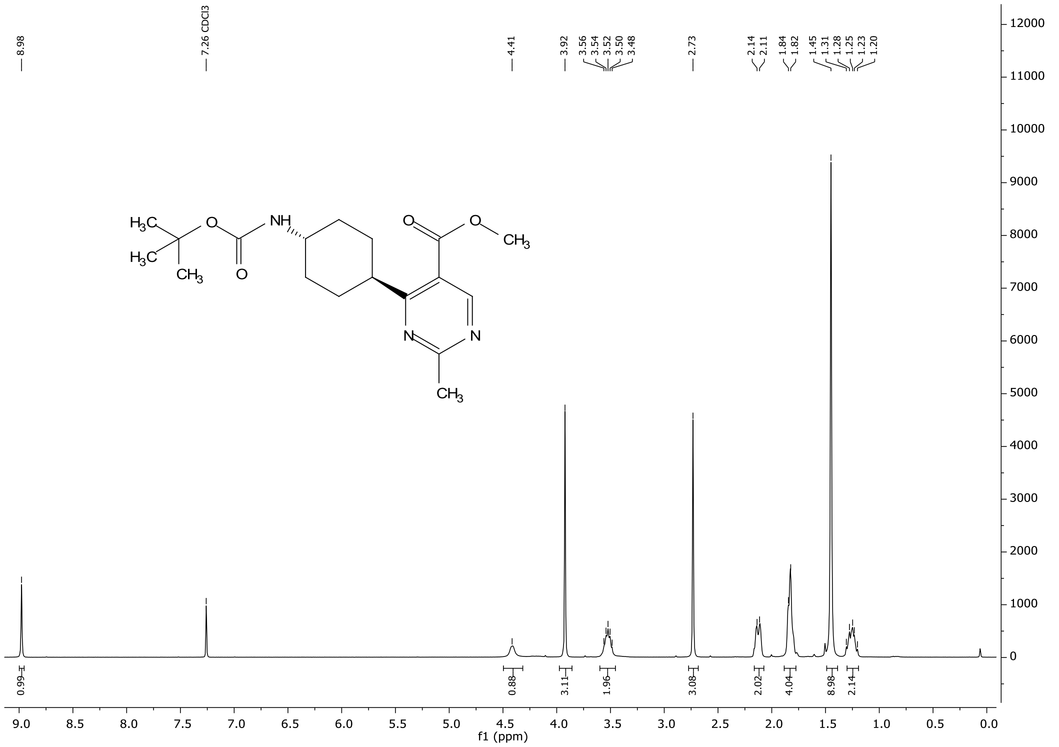

Figure S37. ^{1}H NMR (400 MHz, $CDCl_3$) spectrum of methyl 4-{*trans*-4-[(*tert*-butoxycarbonyl)amino]cyclohexyl}-2-methylpyrimidine-5-carboxylate (**4i**)

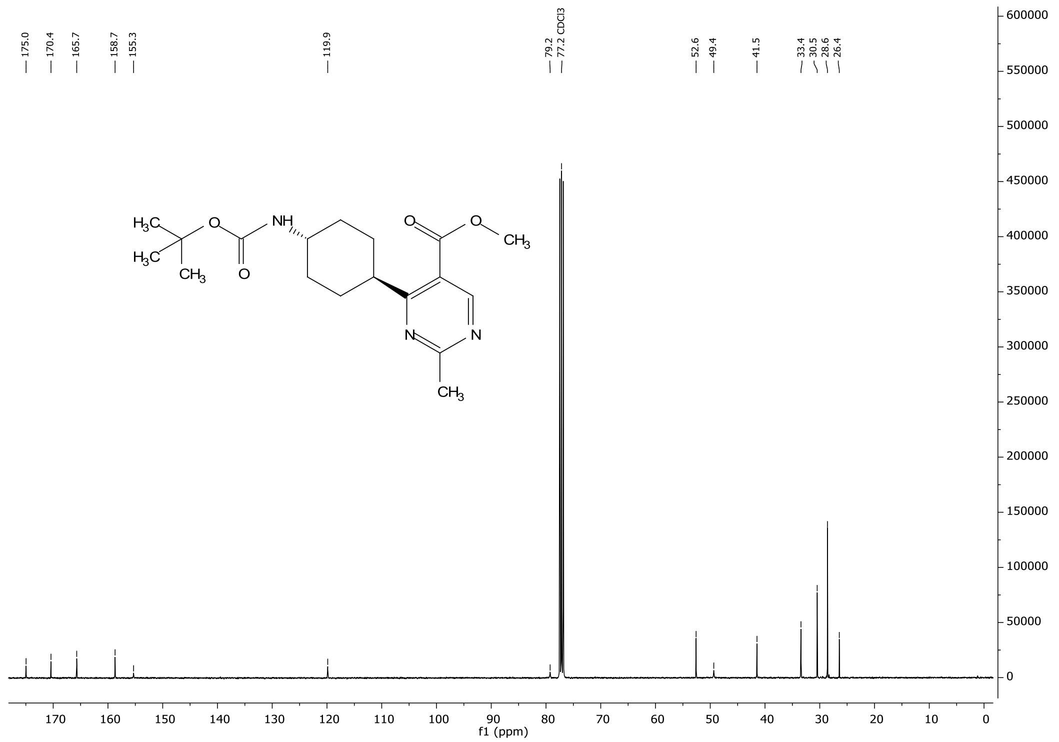

Figure S38. ^{13}C NMR (101 MHz, $CDCl_3$) spectrum of methyl 4-{*trans*-4-[(*tert*-butoxycarbonyl)amino]cyclohexyl}-2-methylpyrimidine-5-carboxylate (**4i**)

Compound Spectrum SmartFormula Report

Analysis Info

Analysis Name	D:\Data\PVP-675.d	Acquisition Date	3/19/2026 10:08:16 AM
Method	DirectInfusion_TuneLow_pos.m	Operator	hplc
Sample Name	PVP-675	Instrument	micrOTOF-Q III 8228888.20448
Comment	SB		

Acquisition Parameter

Source Type	ESI	Ion Polarity	Positive	Set Nebulizer	0.4 Bar
Focus	Not active	Set Capillary	4500 V	Set Dry Heater	180 °C
Scan Begin	50 m/z	Set End Plate Offset	-500 V	Set Dry Gas	4.0 l/min
Scan End	1000 m/z	Set Collision Cell RF	140.0 Vpp	Set Divert Valve	Waste

#	RT [min]	Area	Int. Type	I	S/N	Chromatogram	Max. m/z	FWHM [min]
n.a.	0.1	n.a.	Single spectrum	n.a.	n.a.	n.a.	304.2619	n.a.
n.a.	4.1	n.a.	Single spectrum	n.a.	n.a.	n.a.	372.1891	n.a.
n.a.	0.5	n.a.	Single spectrum	n.a.	n.a.	n.a.	304.2617	n.a.
n.a.	4.3	n.a.	Single spectrum	n.a.	n.a.	n.a.	372.1892	n.a.

+MS, 4.3min #258

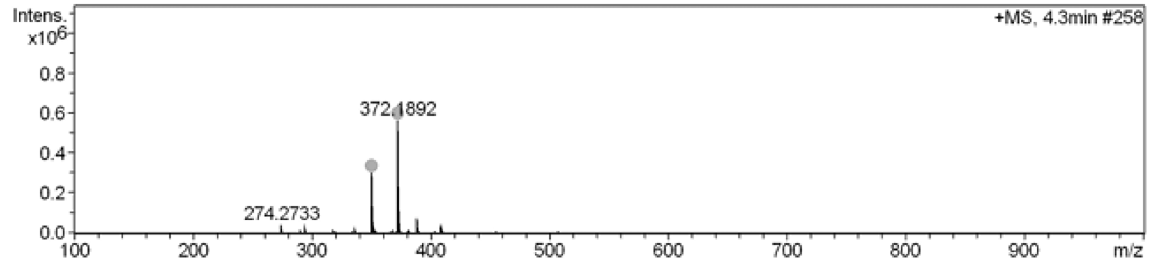

Meas. m/z	#	Ion Formula	m/z	err [ppm]	mSigma	# Sigma	Score	rdb	e⁻ Conf	N-Rule
350.2079	1	C18H28N3O4	350.2074	-1.3	30.4	1	100.00	6.5	even	ok
372.1892	1	C18H27N3NaO4	372.1894	0.5	8.8	1	100.00	6.5	even	ok

Figure S39. HRMS (ESI-TOF) spectrum of methyl 4-{*trans*-4-[(*tert*-butoxycarbonyl)amino]cyclohexyl}-2-methylpyrimidine-5-carboxylate (**4i**)

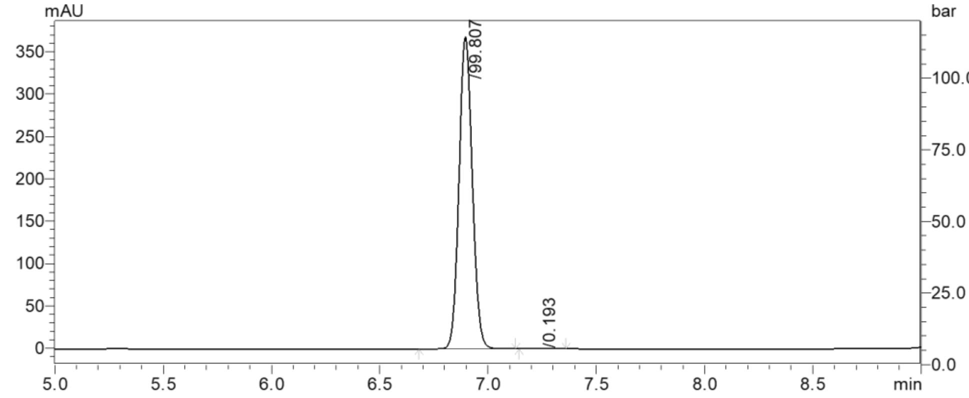

Figure S40. Chiral HPLC analysis of methyl 4-{*trans*-4-[(*tert*-butoxycarbonyl)amino]cyclohexyl}-2-methylpyrimidine-5-carboxylate (**4i**). Conditions: CHIRAL ART Cellulose-SJ (100 × 4.6 mm I.D.); S-10 µL; mobile phase: ACN/H_2O + 0.1% HCOOH (80:20→30:70 in 10 min); T = 36 ˚C; flow rate 1.0 mL/min.

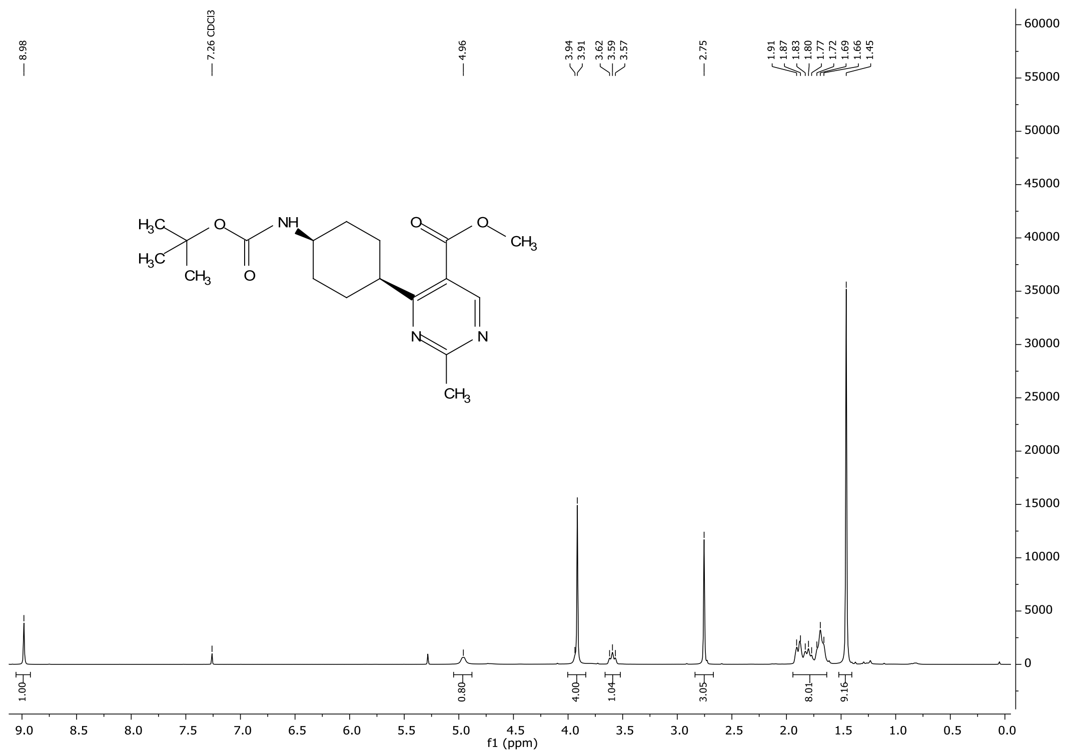

Figure S41. ^{1}H NMR (400 MHz, $CDCl_3$) spectrum of methyl 4-{*cis*-4-[(*tert*-butoxycarbonyl)amino]cyclohexyl}-2-methylpyrimidine-5-carboxylate (**4j**)

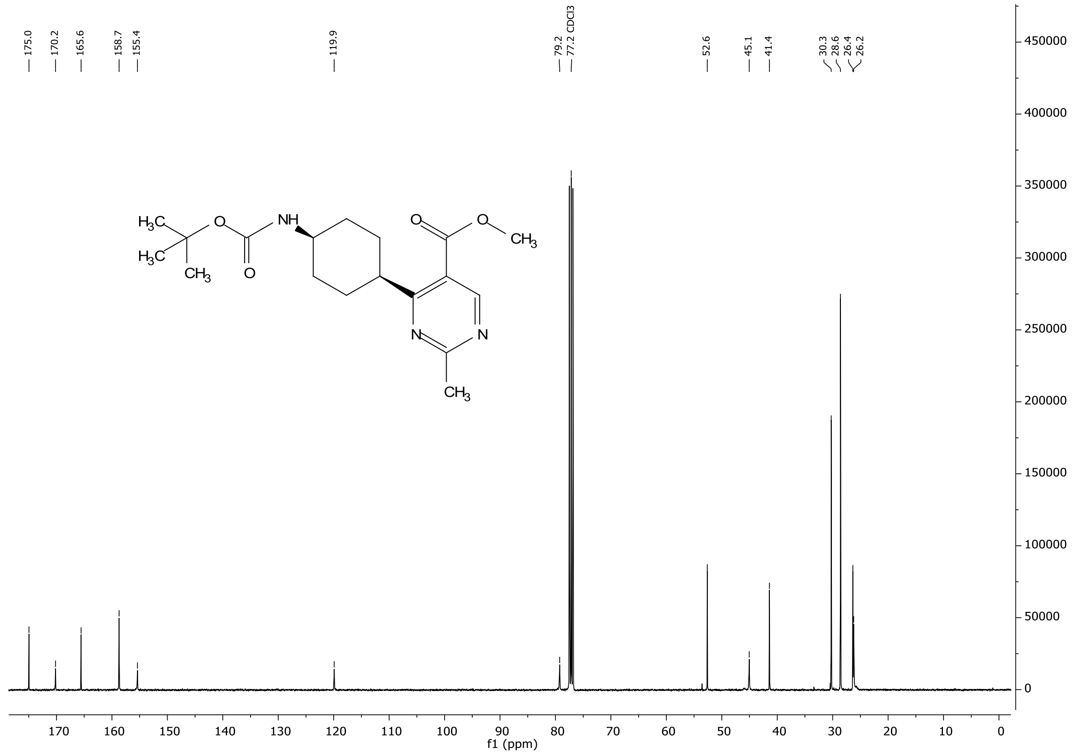

Figure S42. ^{13}C NMR (101 MHz, $CDCl_3$) spectrum of methyl 4-{*cis*-4-[(*tert*-butoxycarbonyl)amino]cyclohexyl}-2-methylpyrimidine-5-carboxylate (**4j**)

Compound Spectrum SmartFormula Report

Analysis Info

Analysis Name	D:\Data\PVP-671.d	Acquisition Date	3/23/2026 9:56:13 AM
Method	DirectInfusion_TuneLow_pos.m	Operator	hplc
Sample Name	PVP-671	Instrument	micrOTOF-Q III 8228888.20448
Comment	SB		

Acquisition Parameter

Source Type	ESI	Ion Polarity	Positive	Set Nebulizer	0.4 Bar
Focus	Not active	Set Capillary	4500 V	Set Dry Heater	180 °C
Scan Begin	50 m/z	Set End Plate Offset	-500 V	Set Dry Gas	4.0 l/min
Scan End	1000 m/z	Set Collision Cell RF	140.0 Vpp	Set Divert Valve	Waste

#	RT [min]	Area	Int. Type	I	S/N	Chromatogram	Max. m/z	FWHM [min]
n.a.	0.3	n.a.	Single spectrum	n.a.	n.a.	n.a.	304.2617	n.a.
n.a.	5.6	n.a.	Single spectrum	n.a.	n.a.	n.a.	372.1894	n.a.

+MS, 5.6min #334

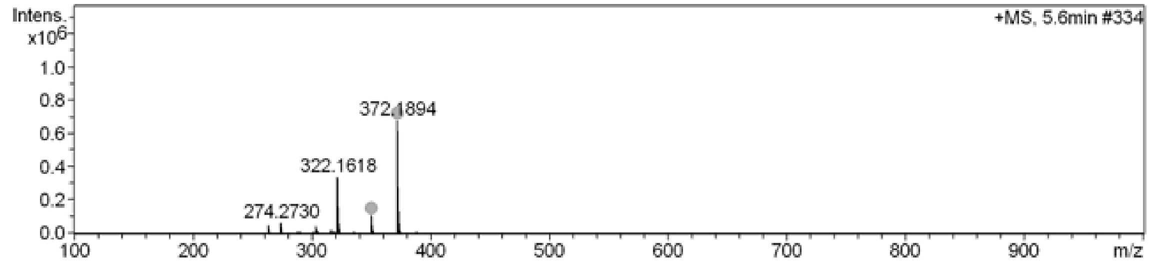

Meas. m/z	#	Ion Formula	m/z	err [ppm]	mSigma	# Sigma	Score	rdb	e⁻ Conf	N-Rule
350.2069	1	C18H28N3O4	350.2074	-1.6	9.8	1	100.00	6.5	even	ok
372.1894	1	C18H27N3NaO4	372.1894	-0.1	6.2	1	100.00	6.5	even	ok

Figure S43. HRMS (ESI-TOF) spectrum of methyl 4-{*cis*-4-[(*tert*-butoxycarbonyl)amino]cyclohexyl}-2-methylpyrimidine-5-carboxylate (**4j**)

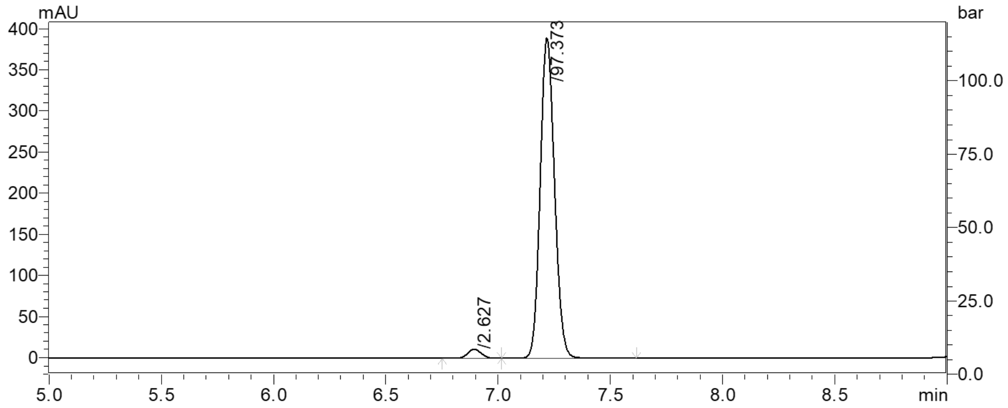

Figure S44. Chiral HPLC analysis of methyl 4-{*cis*-4-[(*tert*-butoxycarbonyl)amino]cyclohexyl}-2-methylpyrimidine-5-carboxylate (**4j**). Conditions: CHIRAL ART Cellulose-SJ (100 × 4.6 mm I.D.); S-10 μL; mobile phase: ACN/H_2O + 0.1% HCOOH (80:20→30:70 in 10 min); T = 36 °C; flow rate 1.0 mL/min.

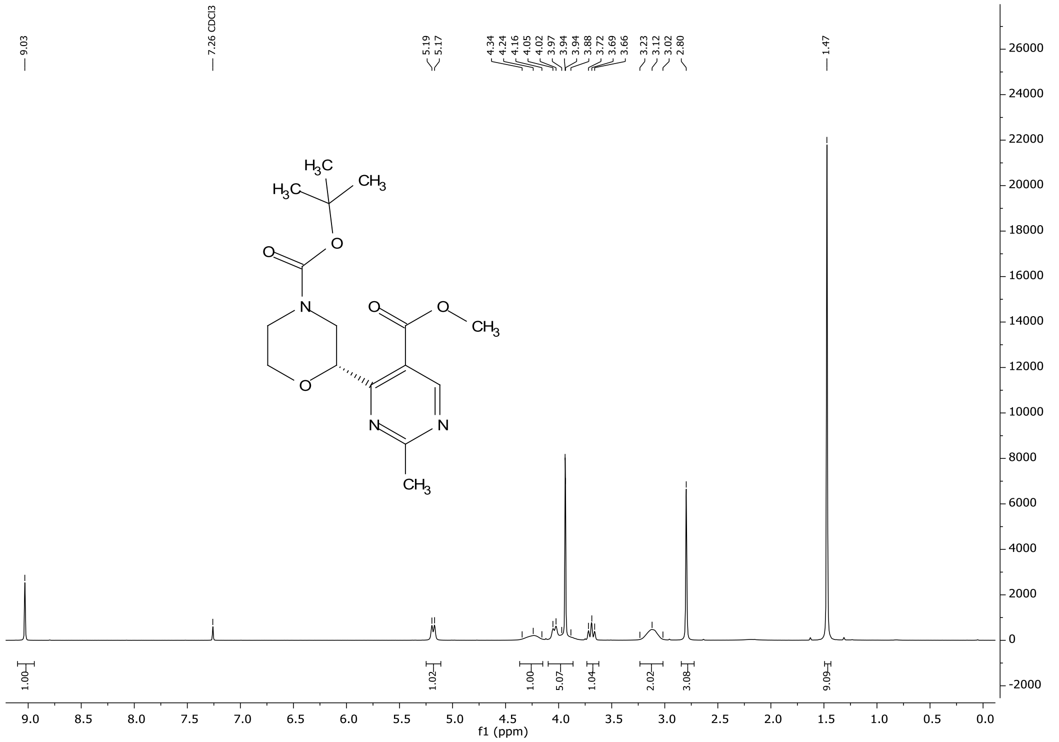

Figure S45. ^{1}H NMR (400 MHz, $CDCl_3$) spectrum of *tert*-butyl (2*R*)-2-[5-(methoxycarbonyl)-2-methylpyrimidin-4-yl]morpholine-4-carboxylate (**4k**)

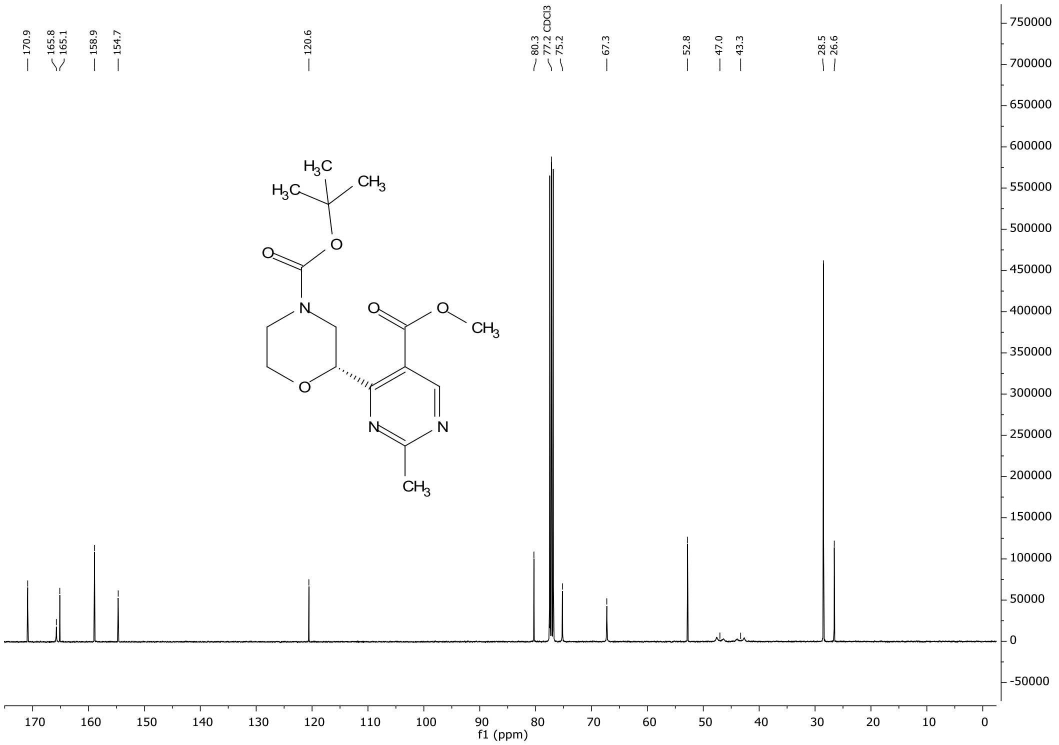

Figure S46. ^{13}C NMR (101 MHz, $CDCl_3$) spectrum of *tert*-butyl (2*R*)-2-[5-(methoxycarbonyl)-2-methylpyrimidin-4-yl]morpholine-4-carboxylate (**4k**)

Compound Spectrum SmartFormula Report

Analysis Info

Analysis Name	D:\Data\PVP-759.d	Acquisition Date	3/26/2026 11:34:28 AM
Method	DirectInfusion_TuneLow_pos.m	Operator	hplc
Sample Name	PVP-759	Instrument	micrOTOF-Q III 8228888.20448
Comment	SB		

Acquisition Parameter

Source Type	ESI	Ion Polarity	Positive	Set Nebulizer	0.4 Bar
Focus	Not active	Set Capillary	4500 V	Set Dry Heater	180 °C
Scan Begin	50 m/z	Set End Plate Offset	-500 V	Set Dry Gas	4.0 l/min
Scan End	1000 m/z	Set Collision Cell RF	140.0 Vpp	Set Divert Valve	Waste

#	RT [min]	Area	Int. Type	I	S/N	Chromatogram	Max. m/z	FWHM [min]
n.a.	1.9	n.a.	Single spectrum	n.a.	n.a.	n.a.	250.1414	n.a.
n.a.	3.1	n.a.	Single spectrum	n.a.	n.a.	n.a.	338.1710	n.a.

+MS, 3.1min #186

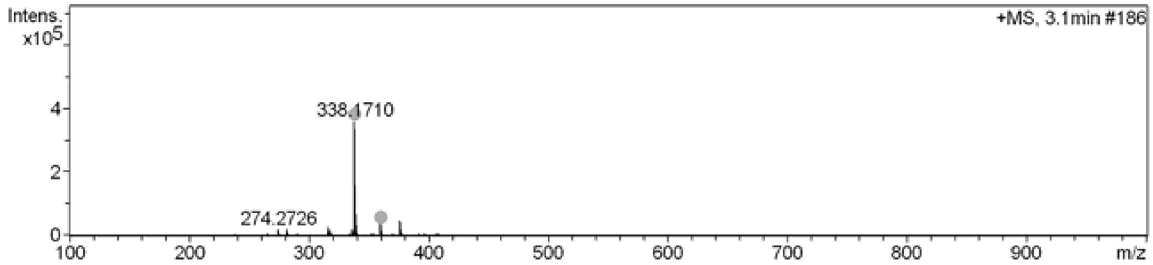

Meas. m/z	#	Ion Formula	m/z	err [ppm]	mSigma	# Sigma	Score	rdb	e⁻ Conf	N-Rule
338.1710	1	C16H24N3O5	338.1710	-0.1	4.9	1	100.00	6.5	even	ok
360.1527	1	C16H23N3NaO5	360.1530	-0.7	3.2	1	100.00	6.5	even	ok

Figure S47. HRMS (ESI-TOF) spectrum of *tert*-butyl (2*R*)-2-[5-(methoxycarbonyl)-2-methylpyrimidin-4-yl]morpholine-4-carboxylate (**4k**)

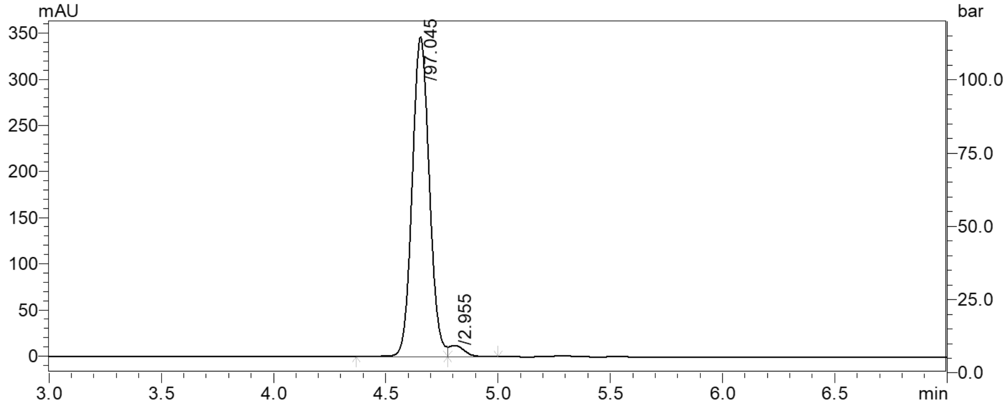

Figure S48. Chiral HPLC analysis of *tert*-butyl (2*R*)-2-[5-(methoxycarbonyl)-2-methylpyrimidin-4-yl]morpholine-4-carboxylate (**4k**). Conditions: CHIRAL ART Cellulose-SJ (100 × 4.6 mm I.D.); S-10 μL; mobile phase: ACN/H_2O + 0.1% HCOOH (80:20→30:70 in 10 min); T = 36 °C; flow rate 1.0 mL/min.

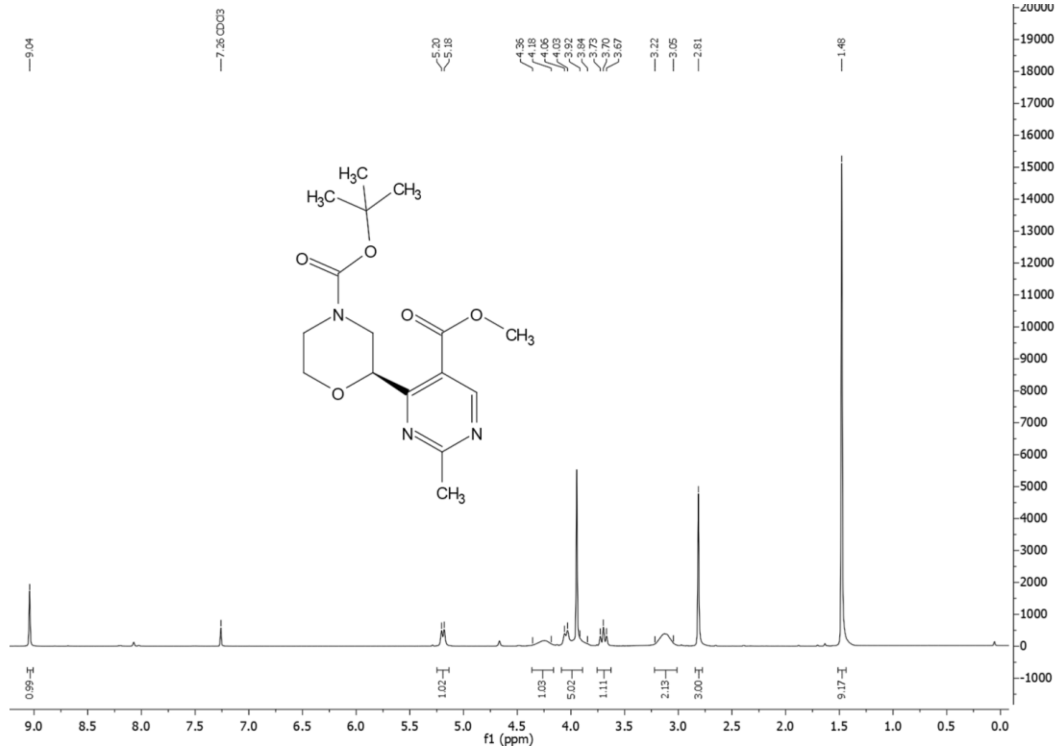

Figure S49. ^{1}H NMR (400 MHz, $CDCl_3$) spectrum of *tert*-butyl (2*S*)-2-[5-(methoxycarbonyl)-2-methylpyrimidin-4-yl]morpholine-4-carboxylate (**4l**)

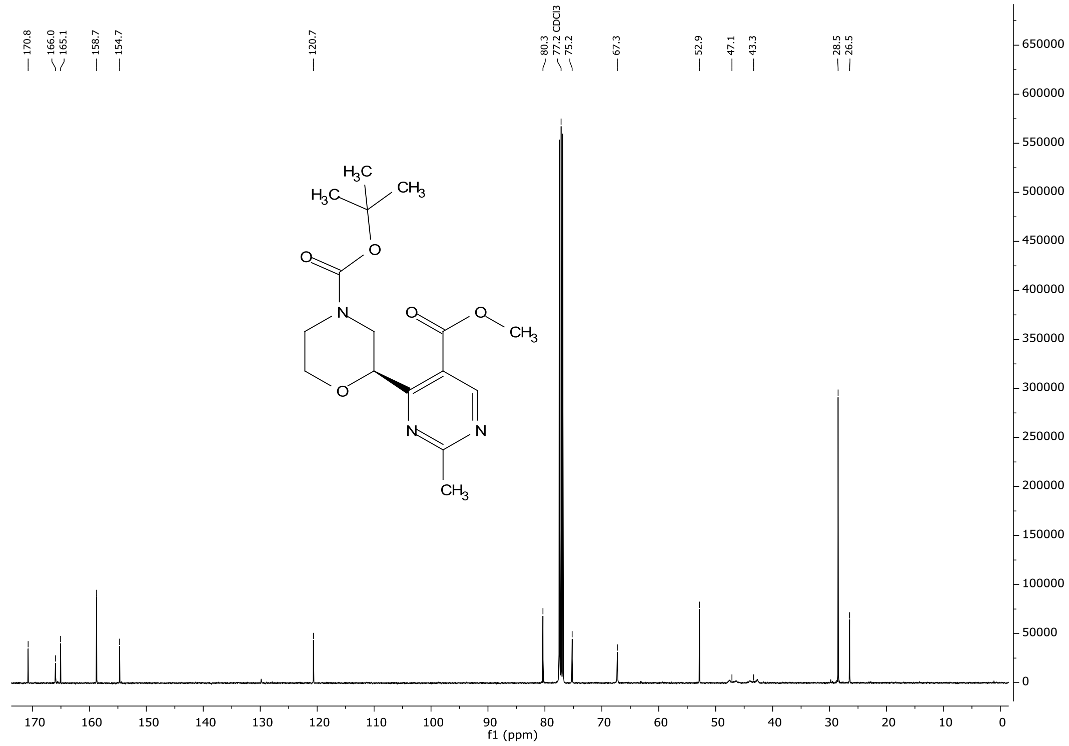

Figure S50. ^{13}C NMR (101 MHz, $CDCl_3$) spectrum of *tert*-butyl (2*S*)-2-[5-(methoxycarbonyl)-2-methylpyrimidin-4-yl]morpholine-4-carboxylate (**4l**)

Compound Spectrum SmartFormula Report

Analysis Info

Analysis Name	D:\Data\PVP-756.d	Acquisition Date	3/18/2026 2:21:17 PM
Method	DirectInfusion_TuneLow_pos.m	Operator	hplc
Sample Name	PVP-756	Instrument	micrOTOF-Q III 8228888.20448
Comment	SB		

Acquisition Parameter

Source Type	ESI	Ion Polarity	Positive	Set Nebulizer	0.4 Bar
Focus	Not active	Set Capillary	4500 V	Set Dry Heater	180 °C
Scan Begin	50 m/z	Set End Plate Offset	-500 V	Set Dry Gas	4.0 l/min
Scan End	1000 m/z	Set Collision Cell RF	140.0 Vpp	Set Divert Valve	Waste

#	RT [min]	Area	Int. Type	I	S/N	Chromatogram	Max. m/z	FWHM [min]
n.a.	0.4	n.a.	Single spectrum	n.a.	n.a.	n.a.	304.2620	n.a.
n.a.	3.9	n.a.	Single spectrum	n.a.	n.a.	n.a.	338.1710	n.a.

+MS, 3.9min #234

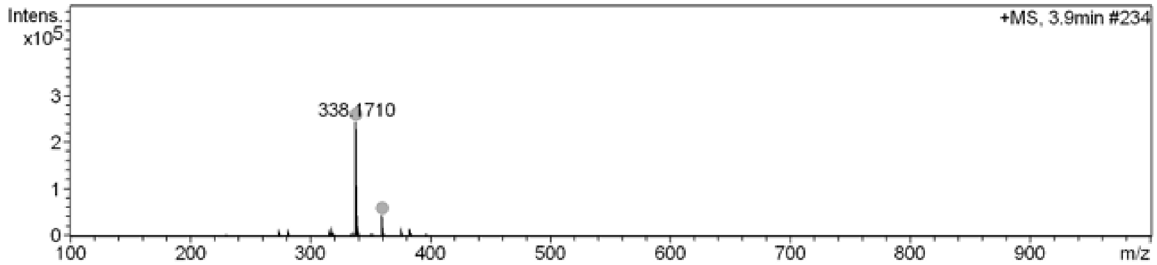

Meas. m/z	#	Ion Formula	m/z	err [ppm]	mSigma	# Sigma	Score	rdb	e⁻ Conf	N-Rule
338.1710	1	C16H24N3O5	338.1710	0.2	16.5	1	100.00	6.5	even	ok
360.1531	1	C16H23N3NaO5	360.1530	0.2	7.1	1	100.00	6.5	even	ok

Figure S51. HRMS (ESI-TOF) spectrum of *tert*-butyl (2*S*)-2-[5-(methoxycarbonyl)-2-methylpyrimidin-4-yl]morpholine-4-carboxylate (**4l**)

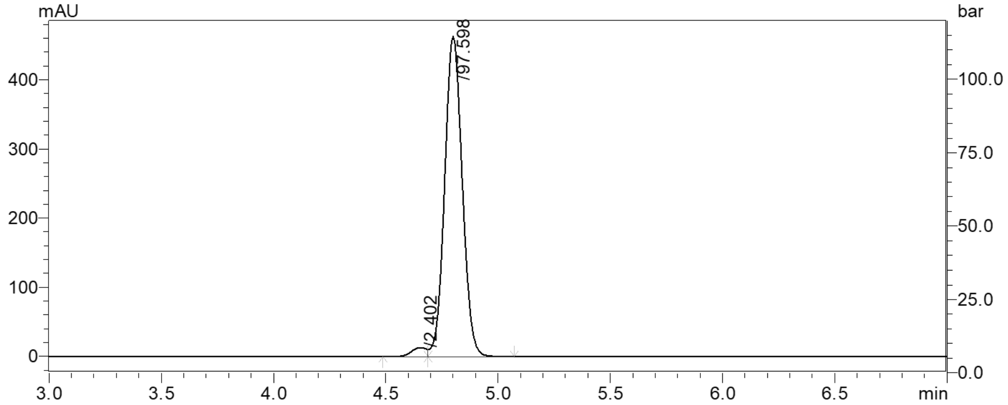

Figure S52. Chiral HPLC analysis of *tert*-butyl (2*S*)-2-[5-(methoxycarbonyl)-2-methylpyrimidin-4-yl]morpholine-4-carboxylate (**4l**). Conditions: CHIRAL ART Cellulose-SJ (100 × 4.6 mm I.D.); S-10 µL; mobile phase: ACN/H_2O + 0.1% HCOOH (80:20→30:70 in 10 min); T = 36 ˚C; flow rate 1.0 mL/min.

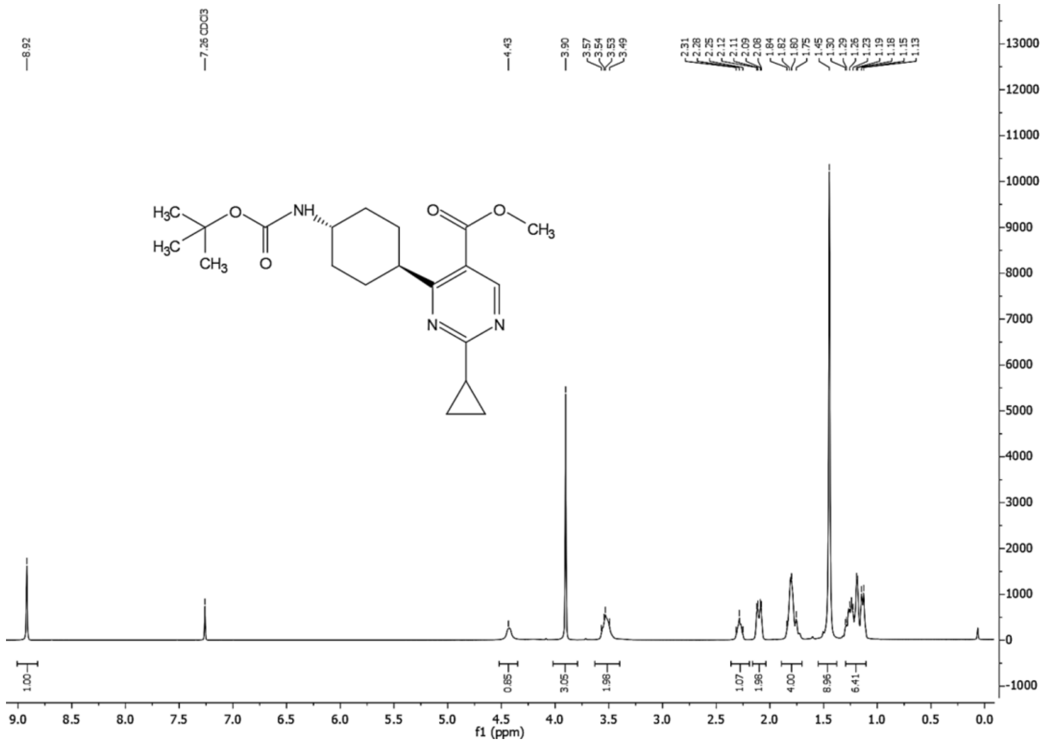

Figure S53. ^{1}H NMR (400 MHz, $CDCl_3$) spectrum of methyl 4-{*trans*-4-[(*tert*-butoxycarbonyl)amino]cyclohexyl}-2-cyclopropylpyrimidine-5-carboxylate (**4m**)

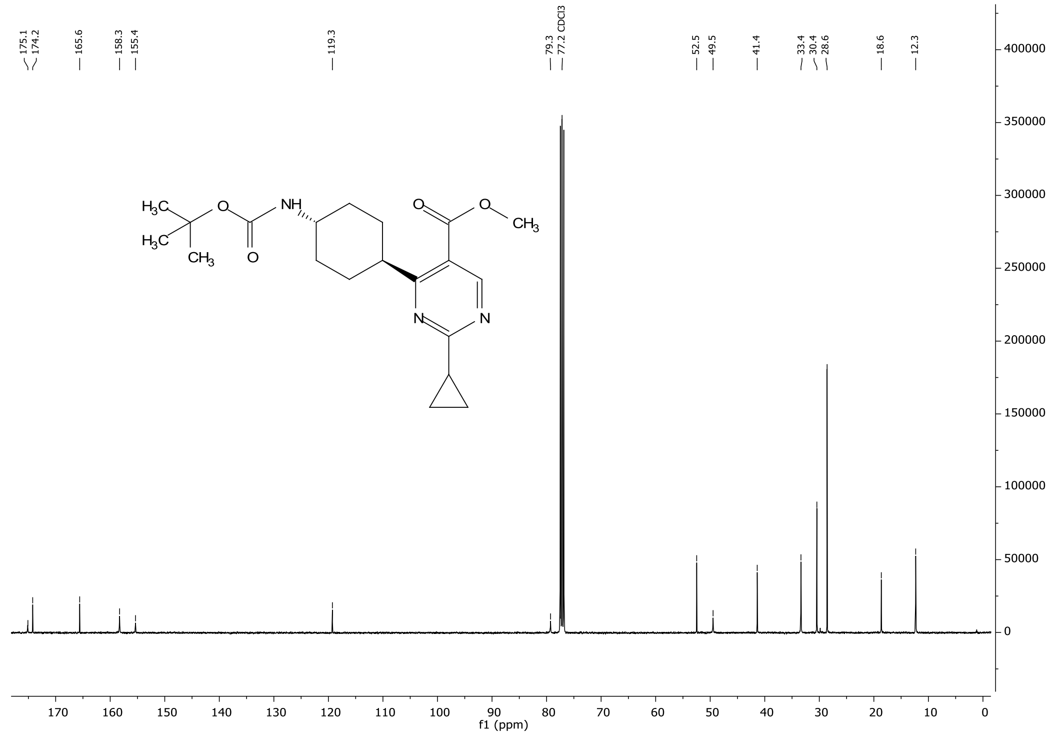

Figure S54. ^{13}C NMR (101 MHz, $CDCl_3$) spectrum of methyl 4-{*trans*-4-[(*tert*-butoxycarbonyl)amino]cyclohexyl}-2-cyclopropylpyrimidine-5-carboxylate (**4m**)

Compound Spectrum SmartFormula Report

Analysis Info

Analysis Name	D:\Data\PVP-768.d	Acquisition Date	3/17/2026 11:27:20 AM
Method	DirectInfusion_TuneLow_pos.m	Operator	hplc
Sample Name	PVP-768	Instrument	micrOTOF-Q III 8228888.20448
Comment	SB		

Acquisition Parameter

Source Type	ESI	Ion Polarity	Positive	Set Nebulizer	0.4 Bar
Focus	Not active	Set Capillary	4500 V	Set Dry Heater	180 °C
Scan Begin	50 m/z	Set End Plate Offset	-500 V	Set Dry Gas	4.0 l/min
Scan End	1000 m/z	Set Collision Cell RF	140.0 Vpp	Set Divert Valve	Waste

#	RT [min]	Area	Int. Type	I	S/N	Chromatogram	Max. m/z	FWHM [min]
n.a.	0.1	n.a.	Single spectrum	n.a.	n.a.	n.a.	304.2618	n.a.
n.a.	4.1	n.a.	Single spectrum	n.a.	n.a.	n.a.	398.2051	n.a.
n.a.	5.2	n.a.	Single spectrum	n.a.	n.a.	n.a.	398.2049	n.a.

+MS, 4.1min #244

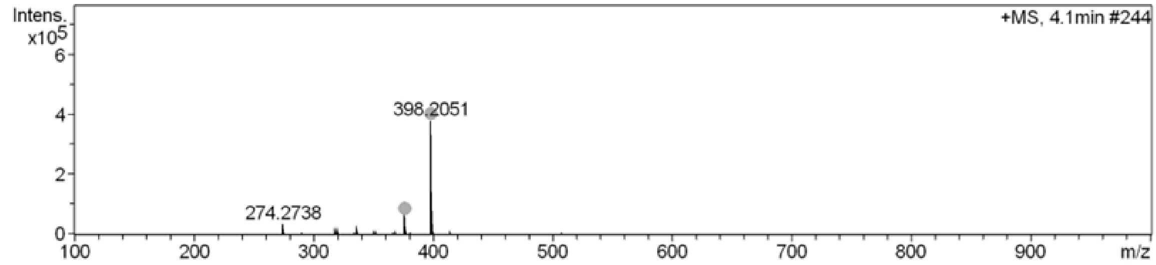

Meas. m/z	#	Ion Formula	m/z	err [ppm]	mSigma	# Sigma	Score	rdb	e⁻ Conf	N-Rule
376.2236	1	C20H30N3O4	376.2231	1.3	4.5	1	100.00	7.5	even	ok
398.2051	1	C20H29N3NaO4	398.2050	-0.3	4.5	1	100.00	7.5	even	ok

Figure S55. HRMS (ESI-TOF) spectrum of methyl 4-{*trans*-4-[(*tert*-butoxycarbonyl)amino]cyclohexyl}-2-cyclopropylpyrimidine-5-carboxylate (**4m**)

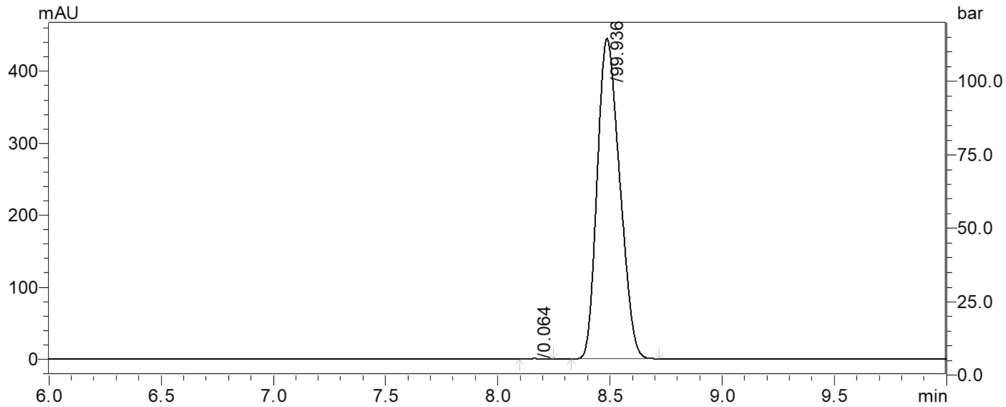

Figure S56. Chiral HPLC analysis of methyl 4-{*trans*-4-[(*tert*-butoxycarbonyl)amino]cyclohexyl}-2-cyclopropylpyrimidine-5-carboxylate (**4m**). Conditions: CHIRAL ART Cellulose-SB (100 × 4.6 mm I.D.); S-10 µL; mobile phase: ACN/H_2O + 0.1% HCOOH (70:30→20:80 in 10 min); T = 36 °C; flow rate 1.0 mL/min.

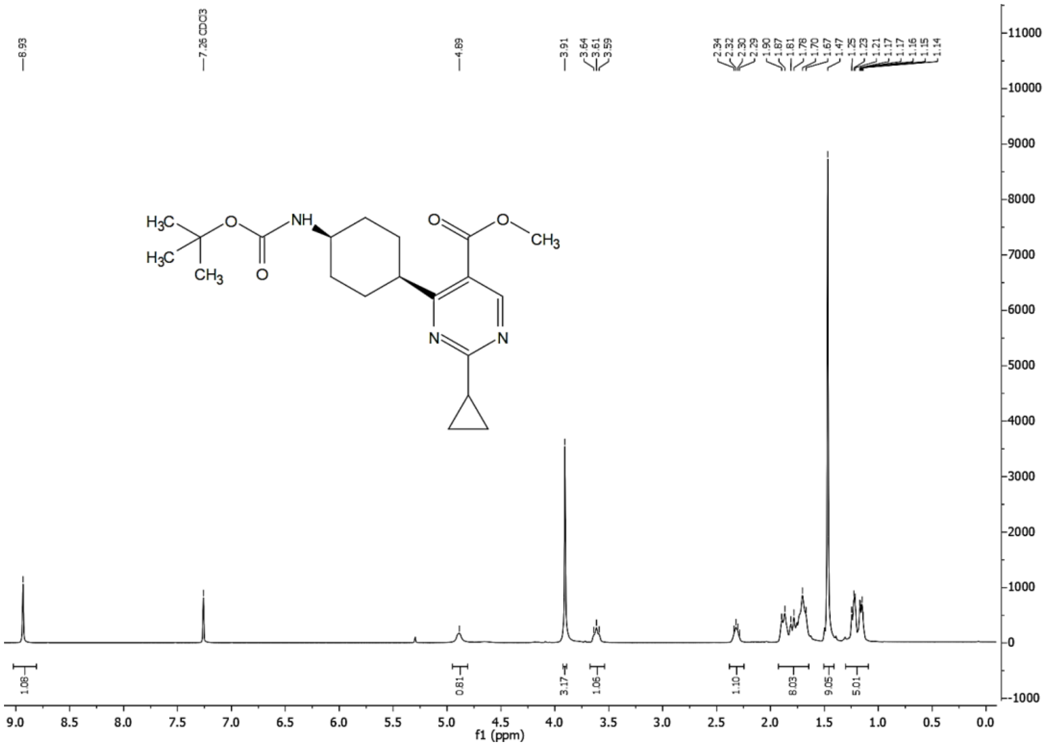

Figure S57. ^{1}H NMR (400 MHz, $CDCl_3$) spectrum of methyl 4-{*cis*-4-[(*tert*-butoxycarbonyl)amino]cyclohexyl}-2-cyclopropylpyrimidine-5-carboxylate (**4n**)

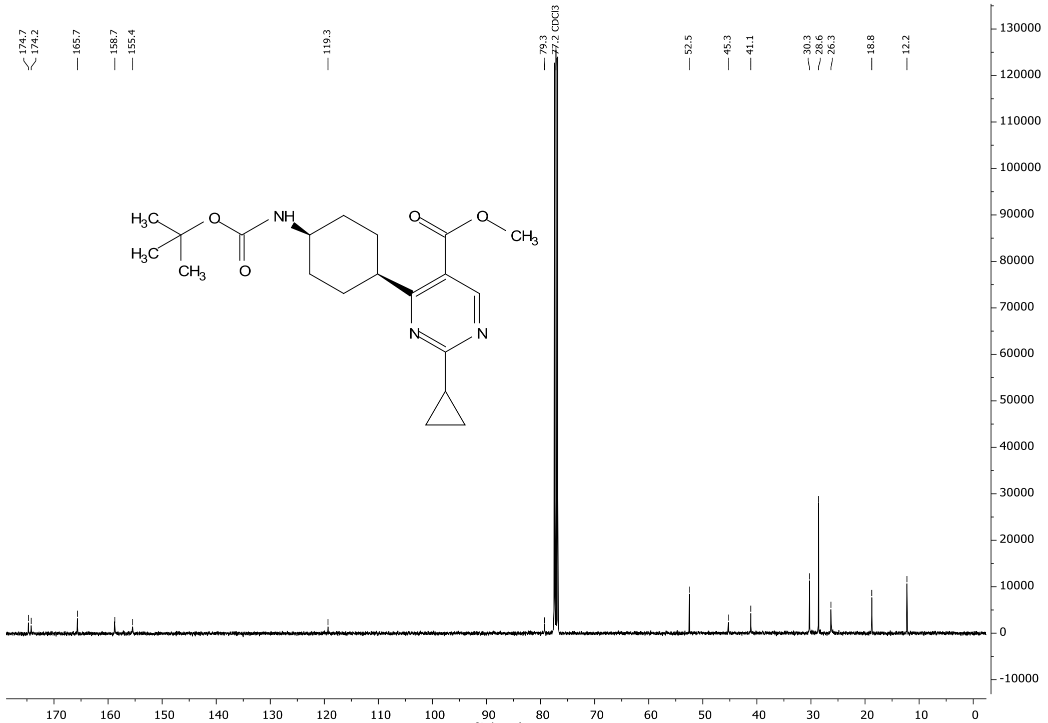

Figure S58. ^{13}C NMR (101 MHz, $CDCl_3$) spectrum of methyl 4-{*cis*-4-[(*tert*-butoxycarbonyl)amino]cyclohexyl}-2-cyclopropylpyrimidine-5-carboxylate (**4n**)

Compound Spectrum SmartFormula Report

Analysis Info

Analysis Name	D:\Data\PVP-766.d	Acquisition Date	3/10/2026 10:58:13 AM
Method	DirectInfusion_TuneLow_pos.m	Operator	hplc
Sample Name	PVP-766	Instrument	micrOTOF-Q III 8228888.20448
Comment	SB		

Acquisition Parameter

Source Type	ESI	Ion Polarity	Positive	Set Nebulizer	0.4 Bar
Focus	Not active	Set Capillary	4500 V	Set Dry Heater	180 °C
Scan Begin	50 m/z	Set End Plate Offset	-500 V	Set Dry Gas	4.0 l/min
Scan End	1000 m/z	Set Collision Cell RF	140.0 Vpp	Set Divert Valve	Waste

#	RT [min]	Area	Int. Type	I	S/N	Chromatogram	Max. m/z	FWHM [min]
n.a.	1.0	n.a.	Single spectrum	n.a.	n.a.	n.a.	304.2614	n.a.
n.a.	2.2	n.a.	Single spectrum	n.a.	n.a.	n.a.	376.2231	n.a.

+MS, 2.2min #134

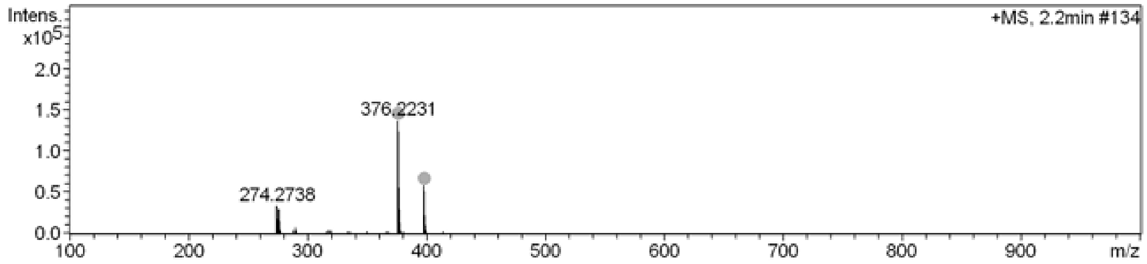

Meas. m/z	#	Ion Formula	m/z	err [ppm]	mSigma	# Sigma	Score	rdb	e⁻ Conf	N-Rule
376.2231	1	C20H30N3O4	376.2231	0.2	3.8	1	100.00	7.5	even	ok
398.2052	1	C20H29N3NaO4	398.2050	-0.4	4.1	1	100.00	7.5	even	ok

Figure S59. HRMS (ESI-TOF) spectrum of methyl 4-{*cis*-4-[(*tert*-butoxycarbonyl)amino]cyclohexyl}-2-cyclopropylpyrimidine-5-carboxylate (**4n**)

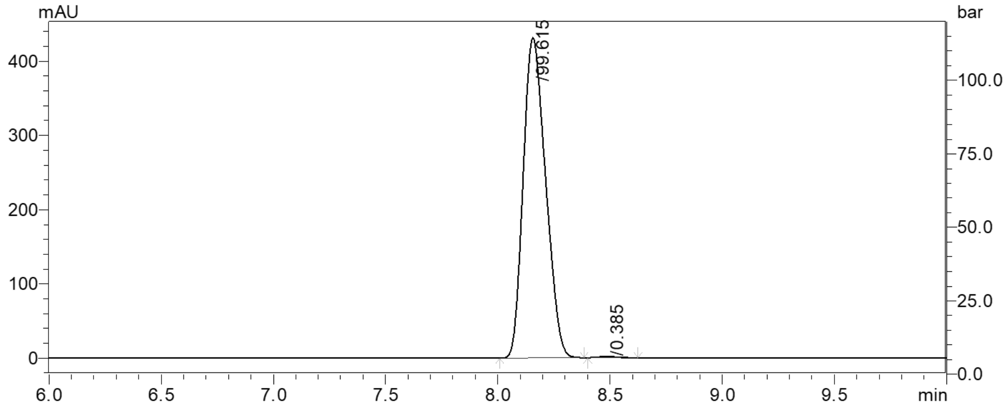

Figure S60. Chiral HPLC analysis of methyl 4-{*cis*-4-[(*tert*-butoxycarbonyl)amino]cyclohexyl}-2-cyclopropylpyrimidine-5-carboxylate (**4n**). Conditions: CHIRAL ART Cellulose-SB (100 × 4.6 mm I.D.); S-10 µL; mobile phase: ACN/H_2O + 0.1% HCOOH (70:30→20:80 in 10 min); T = 36 ˚C; flow rate 1.0 mL/min.

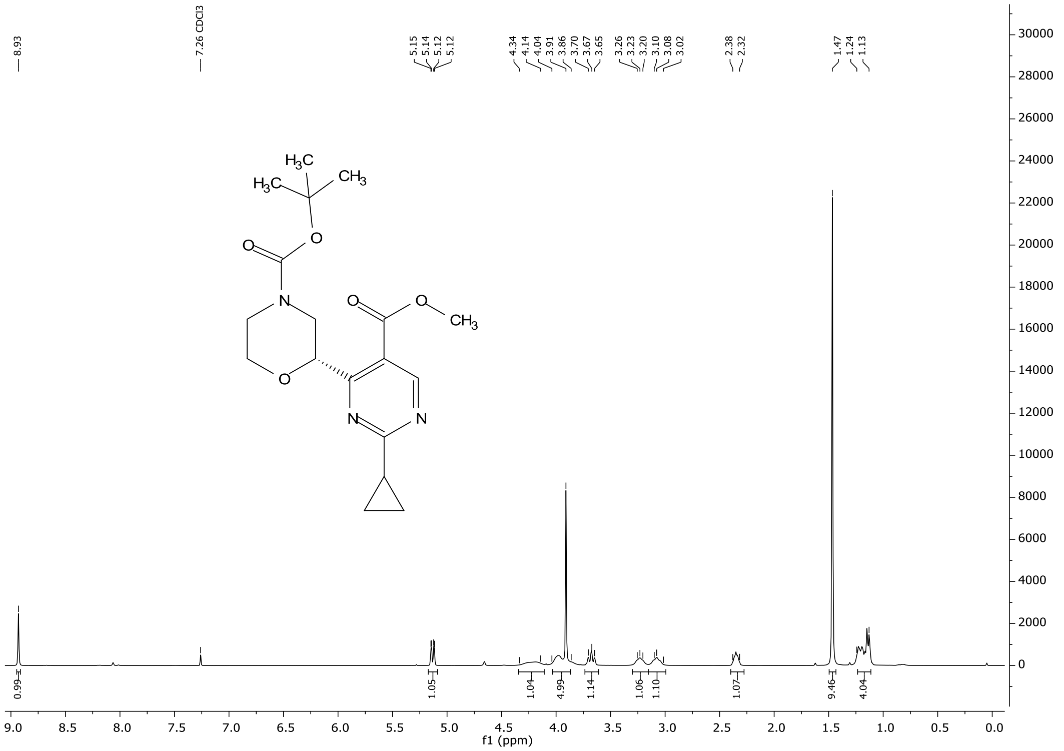

Figure S61. ^{1}H NMR (400 MHz, $CDCl_3$) spectrum of *tert*-butyl (2*R*)-2-[2-cyclopropyl-5-(methoxycarbonyl)pyrimidin-4-yl]morpholine-4-carboxylate (**4o**)

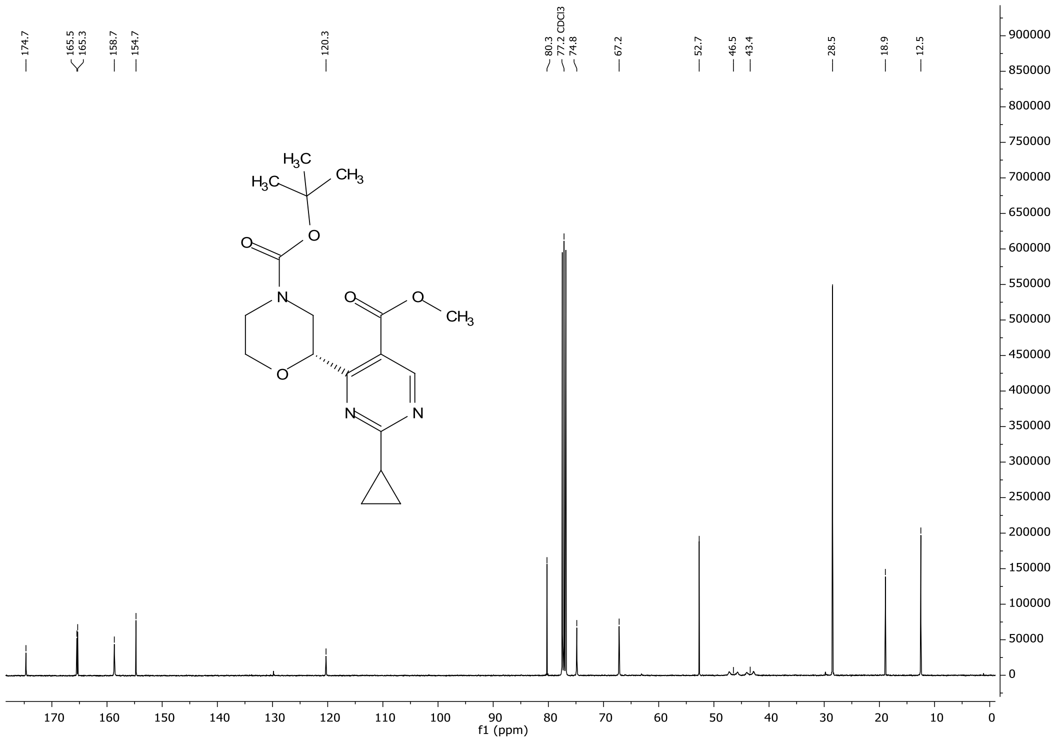

Figure S62. ^{13}C NMR (101 MHz, $CDCl_3$) spectrum of *tert*-butyl (2*R*)-2-[2-cyclopropyl-5-(methoxycarbonyl)pyrimidin-4-yl]morpholine-4-carboxylate (**4o**)

Compound Spectrum SmartFormula Report

Analysis Info

Analysis Name	D:\Data\PVP-761.d	Acquisition Date	3/17/2026 11:11:48 AM
Method	DirectInfusion_TuneLow_pos.m	Operator	hplc
Sample Name	PVP-761	Instrument	micrOTOF-Q III 8228888.20448
Comment	SB		

Acquisition Parameter

Source Type	ESI	Ion Polarity	Positive	Set Nebulizer	0.4 Bar
Focus	Not active	Set Capillary	4500 V	Set Dry Heater	180 °C
Scan Begin	50 m/z	Set End Plate Offset	-500 V	Set Dry Gas	4.0 l/min
Scan End	1000 m/z	Set Collision Cell RF	140.0 Vpp	Set Divert Valve	Waste

#	RT [min]	Area	Int. Type	I	S/N	Chromatogram	Max. m/z	FWHM [min]
n.a.	0.3	n.a.	Single spectrum	n.a.	n.a.	n.a.	304.2620	n.a.
n.a.	4.4	n.a.	Single spectrum	n.a.	n.a.	n.a.	364.1866	n.a.

+MS, 4.4min #263

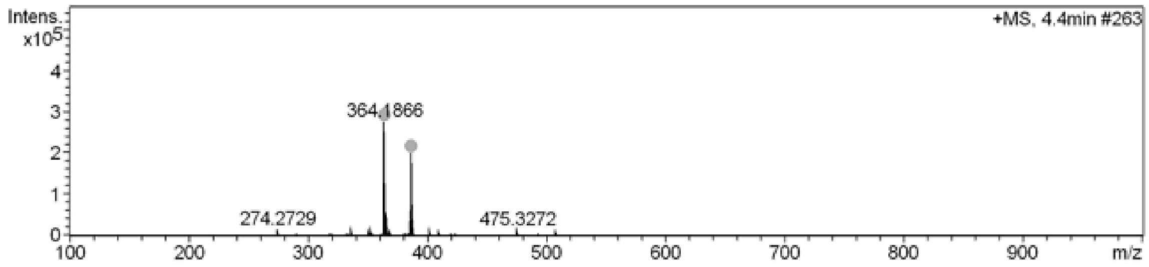

Meas. m/z	#	Ion Formula	m/z	err [ppm]	mSigma	# Sigma	Score	rdb	e⁻ Conf	N-Rule
364.1866	1	C18H26N3O5	364.1867	-0.2	8.4	1	100.00	7.5	even	ok
386.1686	1	C18H25N3NaO5	386.1686	0.2	3.9	1	100.00	7.5	even	ok

Figure S63. HRMS (ESI-TOF) spectrum of *tert*-butyl (2*R*)-2-[2-cyclopropyl-5-(methoxycarbonyl)pyrimidin-4-yl]morpholine-4-carboxylate (**4o**)

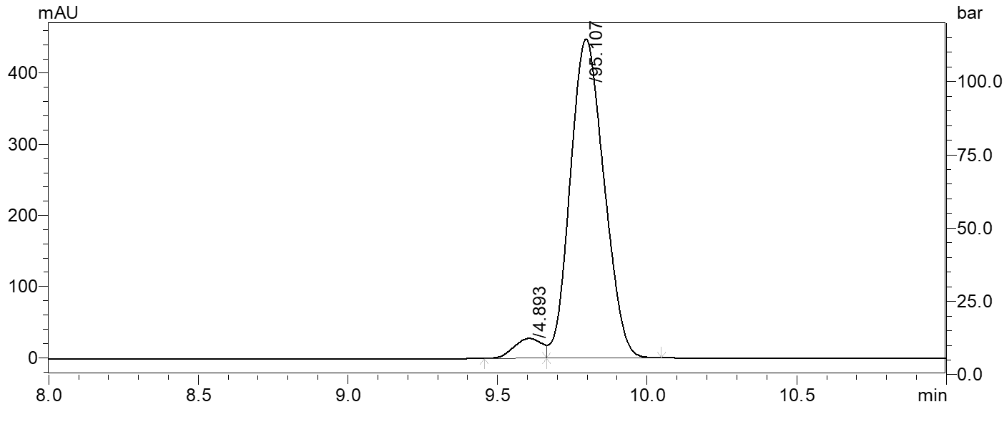

Figure S64. Chiral HPLC analysis of *tert*-butyl (2*R*)-2-[2-cyclopropyl-5-(methoxycarbonyl)pyrimidin-4-yl]morpholine-4-carboxylate (**4o**). Conditions: CHIRAL ART Cellulose-SB (100 × 4.6 mm I.D.); S-10 µL; mobile phase: ACN/H_2O + 0.1% HCOOH (80:20→40:60 in 10 min); T = 36 ˚C; flow rate 1.0 mL/min.

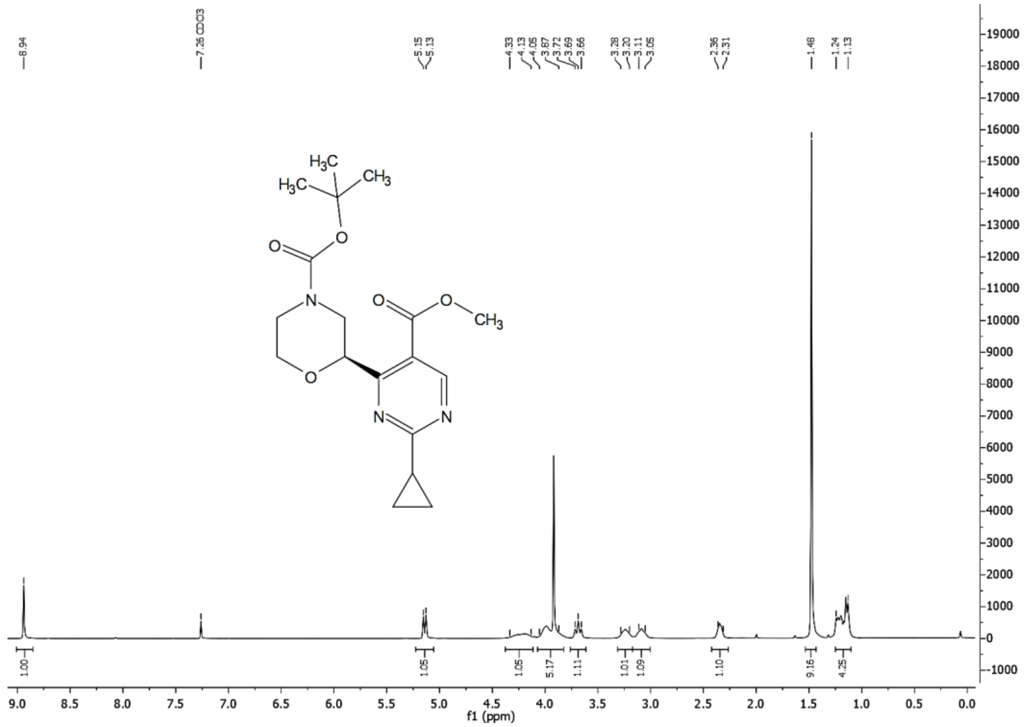

Figure S65. ^{1}H NMR (400 MHz, $CDCl_3$) spectrum of *tert*-butyl (2*S*)-2-[2-cyclopropyl-5-(methoxycarbonyl)pyrimidin-4-yl]morpholine-4-carboxylate (**4p**)

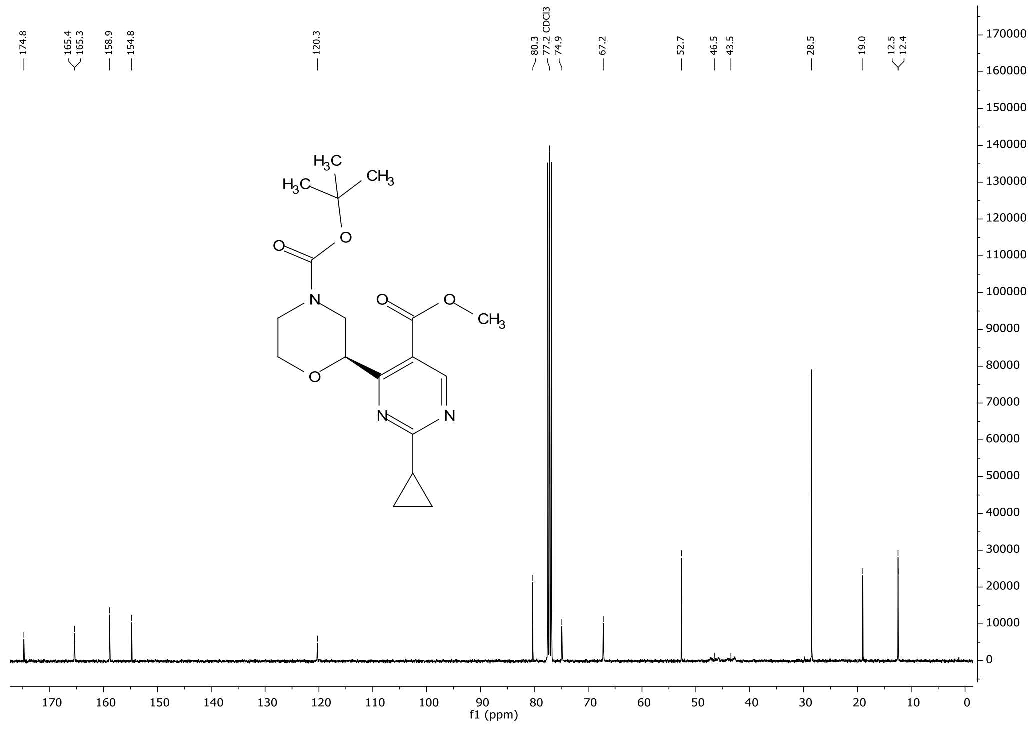

Figure S66. ^{13}C NMR (101 MHz, $CDCl_3$) spectrum of *tert*-butyl (2*S*)-2-[2-cyclopropyl-5-(methoxycarbonyl)pyrimidin-4-yl]morpholine-4-carboxylate (**4p**)

Compound Spectrum SmartFormula Report

Analysis Info

		Acquisition Date	3/10/2026 11:12:22 AM
Analysis Name	D:\Data\PVP-758.d		
Method	DirectInfusion_TuneLow_pos.m	Operator	hplc
Sample Name	PVP-758	Instrument	micrOTOF-Q III 8228888.20448
Comment	SB		

Acquisition Parameter

Source Type	ESI	Ion Polarity	Positive	Set Nebulizer	0.4 Bar
Focus	Not active	Set Capillary	4500 V	Set Dry Heater	180 °C
Scan Begin	50 m/z	Set End Plate Offset	-500 V	Set Dry Gas	4.0 l/min
Scan End	1000 m/z	Set Collision Cell RF	140.0 Vpp	Set Divert Valve	Waste

#	RT [min]	Area	Int. Type	I	S/N	Chromatogram	Max. m/z	FWHM [min]
n.a.	0.1	n.a.	Single spectrum	n.a.	n.a.	n.a.	304.2621	n.a.
n.a.	6.8	n.a.	Single spectrum	n.a.	n.a.	n.a.	386.1687	n.a.
n.a.	5.1	n.a.	Single spectrum	n.a.	n.a.	n.a.	386.1686	n.a.

+MS, 5.1min #307

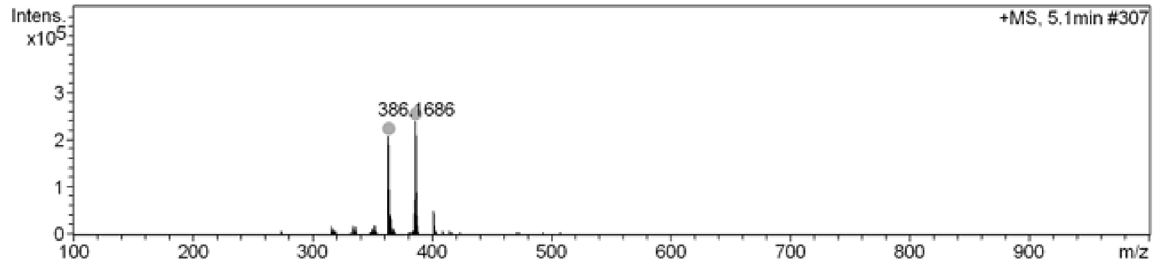

Meas. m/z	#	Ion Formula	m/z	err [ppm]	mSigma	# Sigma	Score	rdb	e⁻ Conf	N-Rule
364.1871	1	C18H26N3O5	364.1867	-1.2	17.3	1	100.00	7.5	even	ok
386.1686	1	C18H25N3NaO5	386.1686	0.2	3.2	1	100.00	7.5	even	ok

Figure S67. HRMS (ESI-TOF) spectrum of *tert*-butyl (2*S*)-2-[2-cyclopropyl-5-(methoxycarbonyl)pyrimidin-4-yl]morpholine-4-carboxylate (**4p**)

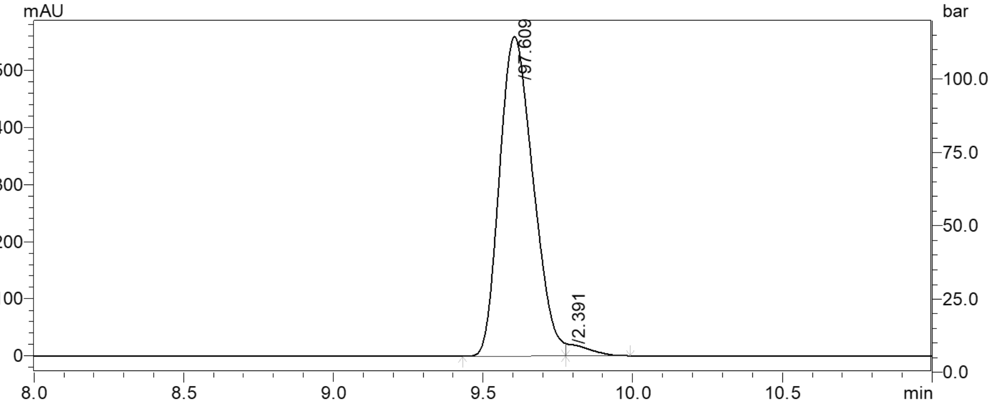

Figure S68. Chiral HPLC analysis of *tert*-butyl (2*S*)-2-[2-cyclopropyl-5-(methoxycarbonyl)pyrimidin-4-yl]morpholine-4-carboxylate (**4p**). Conditions: CHIRAL ART Cellulose-SB (100 × 4.6 mm I.D.); S-10 µL; mobile phase: ACN/H_2O + 0.1% HCOOH (80:20→40:60 in 10 min); T = 36 ˚C; flow rate 1.0 mL/min.

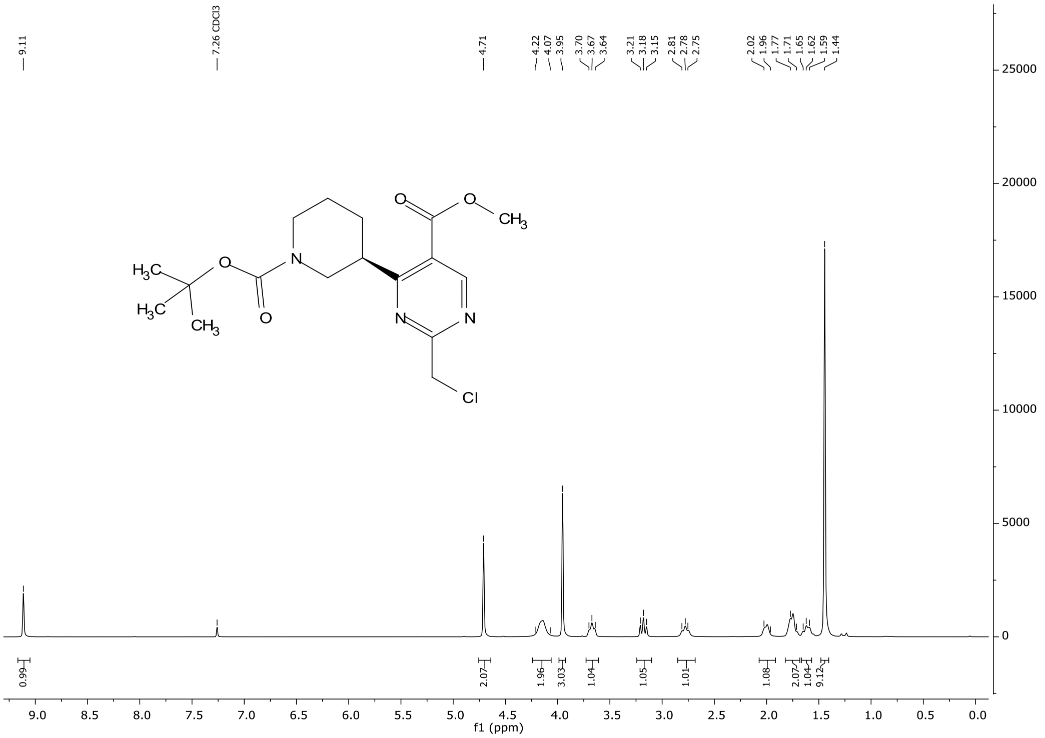

Figure S69. ^{1}H NMR (400 MHz, $CDCl_3$) spectrum of methyl 4-[(3*R*)-1-(*tert*-butoxycarbonyl)piperidin-3-yl]-2-(chloromethyl)pyrimidine-5-carboxylate (**6a**)

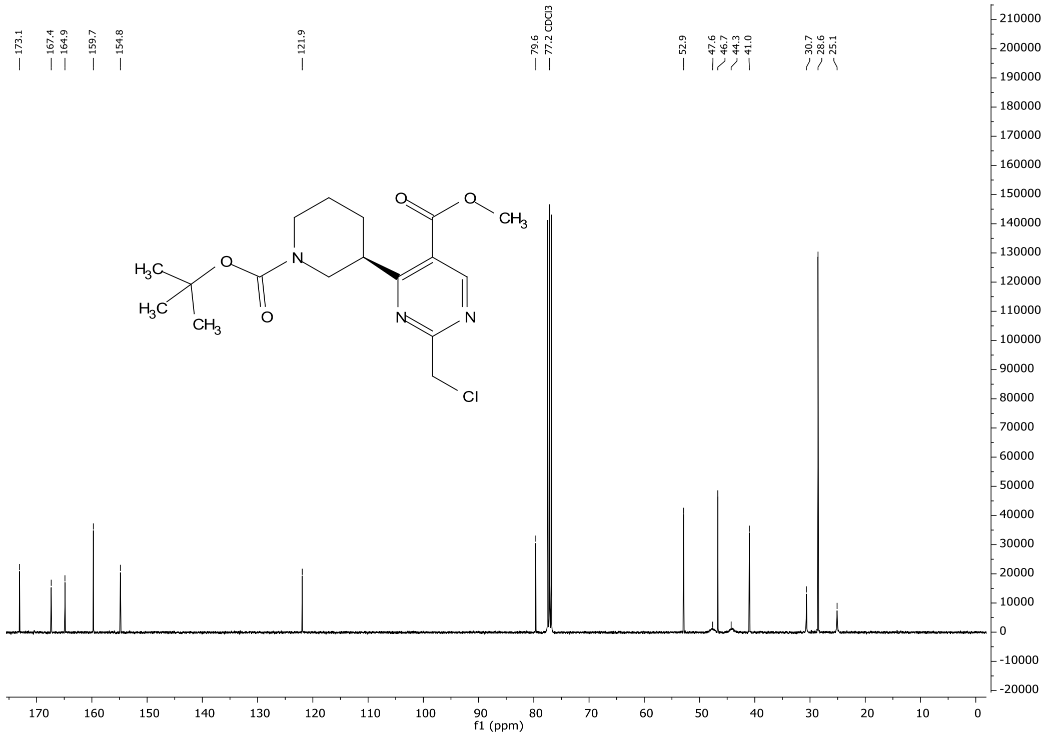

Figure S70. ^{13}C NMR (101 MHz, $CDCl_3$) spectrum of methyl 4-[(3*R*)-1-(*tert*-butoxycarbonyl)piperidin-3-yl]-2-(chloromethyl)pyrimidine-5-carboxylate (**6a**)

Compound Spectrum SmartFormula Report

Analysis Info

Analysis Name	D:\Data\PVP-810.d	Acquisition Date	3/26/2026 12:13:54 PM
Method	DirectInfusion_TuneLow_pos.m	Operator	hplc
Sample Name	PVP-810	Instrument	micrOTOF-Q III 8228888.20448
Comment	SB		

Acquisition Parameter

Source Type	ESI	Ion Polarity	Positive	Set Nebulizer	0.4 Bar
Focus	Not active	Set Capillary	4500 V	Set Dry Heater	180 °C
Scan Begin	50 m/z	Set End Plate Offset	-500 V	Set Dry Gas	4.0 l/min
Scan End	1000 m/z	Set Collision Cell RF	140.0 Vpp	Set Divert Valve	Waste

#	RT [min]	Area	Int. Type	I	S/N	Chromatogram	Max. m/z	FWHM [min]
n.a.	0.5	n.a.	Single spectrum	n.a.	n.a.	n.a.	226.9517	n.a.
n.a.	4.1	n.a.	Single spectrum	n.a.	n.a.	n.a.	370.1528	n.a.

+MS, 4.1min #244

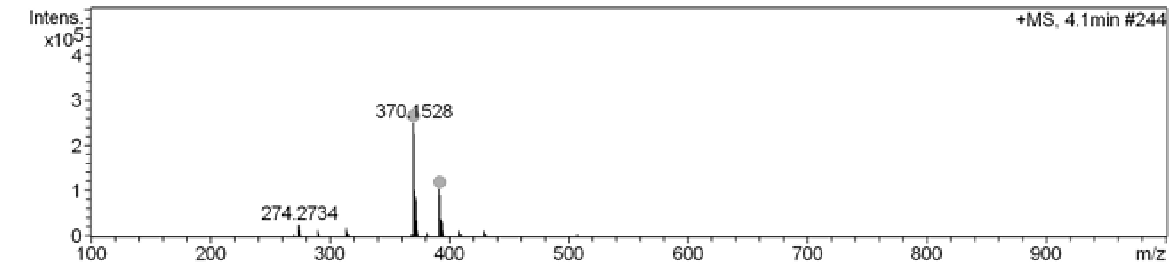

Meas. m/z	#	Ion Formula	m/z	err [ppm]	mSigma	# Sigma	Score	rdb	e⁻ Conf	N-Rule
370.1528	1	C17H25ClN3O4	370.1528	-0.1	3.6	1	100.00	6.5	even	ok
392.1345	1	C17H24ClN3NaO4	392.1348	0.8	8.7	1	100.00	6.5	even	ok

Figure S71. HRMS (ESI-TOF) spectrum of methyl 4-[(3*R*)-1-(*tert*-butoxycarbonyl)piperidin-3-yl]-2-(chloromethyl)pyrimidine-5-carboxylate (**6a**)

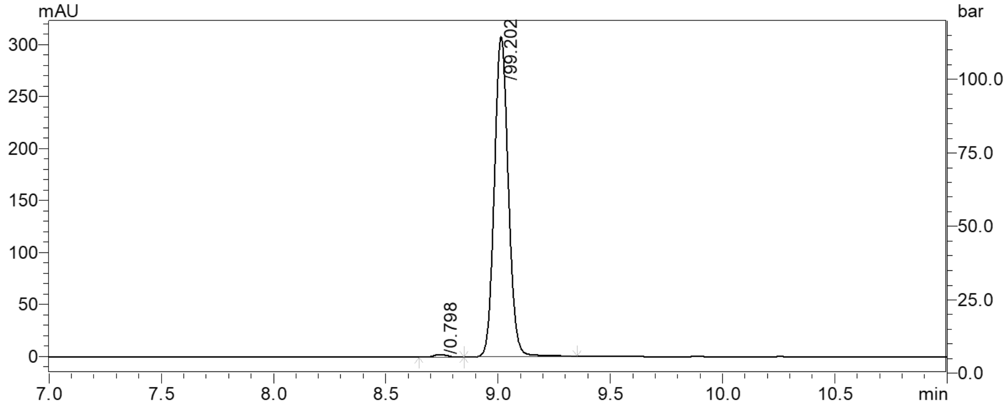

Figure S72. Chiral HPLC analysis of methyl 4-[(3*R*)-1-(*tert*-butoxycarbonyl)piperidin-3-yl]-2-(chloromethyl)pyrimidine-5-carboxylate (**6a**). Conditions: CHIRAL ART Cellulose-SJ (100 × 4.6 mm I.D.); S-10 µL; mobile phase: ACN/H_2O + 0.1% HCOOH (80:20→30:70 in 10 min); T = 36 °C; flow rate 1.0 mL/min.

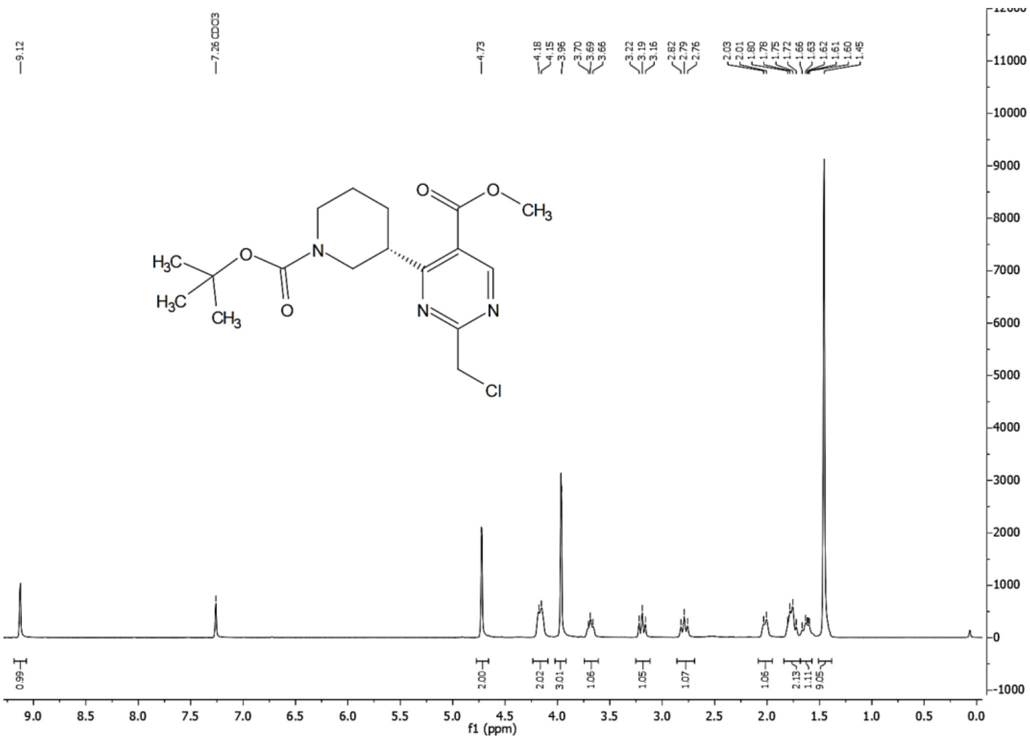

Figure S73. ^{1}H NMR (400 MHz, $CDCl_3$) spectrum of methyl 4-[(3*S*)-1-(*tert*-butoxycarbonyl)piperidin-3-yl]-2-(chloromethyl)pyrimidine-5-carboxylate (**6b**)

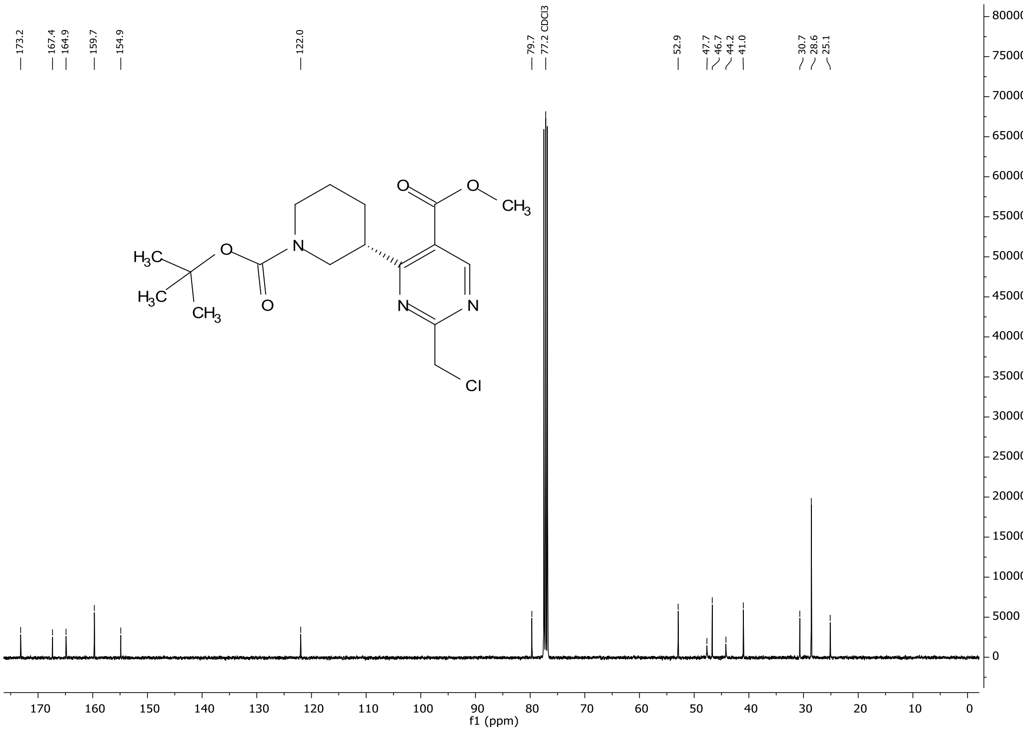

Figure S74. ^{13}C NMR (101 MHz, $CDCl_3$) spectrum of methyl 4-[(3*S*)-1-(*tert*-butoxycarbonyl)piperidin-3-yl]-2-(chloromethyl)pyrimidine-5-carboxylate (**6b**)

Compound Spectrum SmartFormula Report

Analysis Info

Acquisition Date 3/19/2026 10:52:14 AM

Analysis Name D:\Data\PVP-813.d
Method DirectInfusion_TuneLow_pos.m
Sample Name PVP-813
Comment SB

Operator hplc
Instrument micrOTOF-Q III 8228888.20448

Acquisition Parameter

Source Type	ESI	Ion Polarity	Positive	Set Nebulizer	0.4 Bar
Focus	Not active	Set Capillary	4500 V	Set Dry Heater	180 °C
Scan Begin	50 m/z	Set End Plate Offset	-500 V	Set Dry Gas	4.0 l/min
Scan End	1000 m/z	Set Collision Cell RF	140.0 Vpp	Set Divert Valve	Waste

#	RT [min]	Area	Int. Type	I	S/N	Chromatogram	Max. m/z	FWHM [min]
n.a.	0.1	n.a.	Single spectrum	n.a.	n.a.	n.a.	304.2618	n.a.
n.a.	4.3	n.a.	Single spectrum	n.a.	n.a.	n.a.	392.1349	n.a.

+MS, 4.3min #259

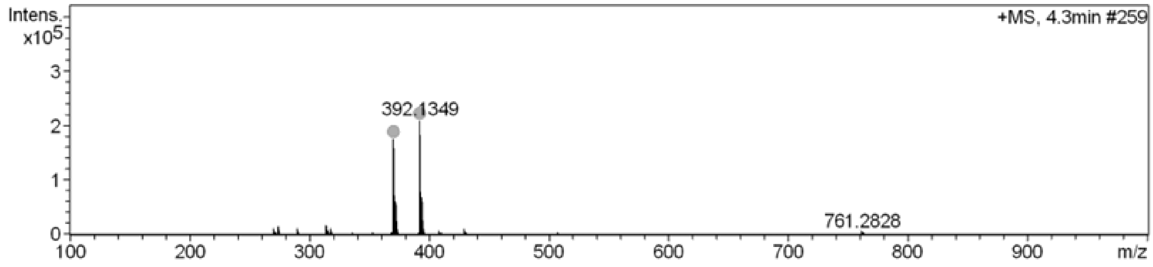

Meas. m/z	#	Ion Formula	m/z	err [ppm]	mSigma	# Sigma	Score	rdb	e⁻ Conf	N-Rule
370.1528	1	C17H25ClN3O4	370.1528	-0.1	2.6	1	100.00	6.5	even	ok
392.1349	1	C17H24ClN3NaO4	392.1348	0.4	5.4	1	100.00	6.5	even	ok

Figure S75. HRMS (ESI-TOF) spectrum of methyl 4-[(3*S*)-1-(*tert*-butoxycarbonyl)piperidin-3-yl]-2-(chloromethyl)pyrimidine-5-carboxylate (**6b**)

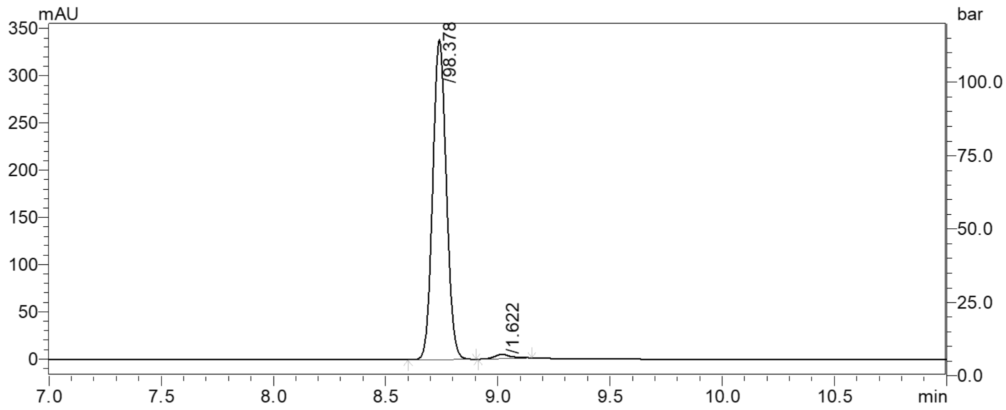

Figure S76. Chiral HPLC analysis of methyl 4-[(3*S*)-1-(*tert*-butoxycarbonyl)piperidin-3-yl]-2-(chloromethyl)pyrimidine-5-carboxylate (**6b**). Conditions: CHIRAL ART Cellulose-SJ (100 × 4.6 mm I.D.); S-10 µL; mobile phase: ACN/H_2O + 0.1% HCOOH (80:20→30:70 in 10 min); T = 36 °C; flow rate 1.0 mL/min.

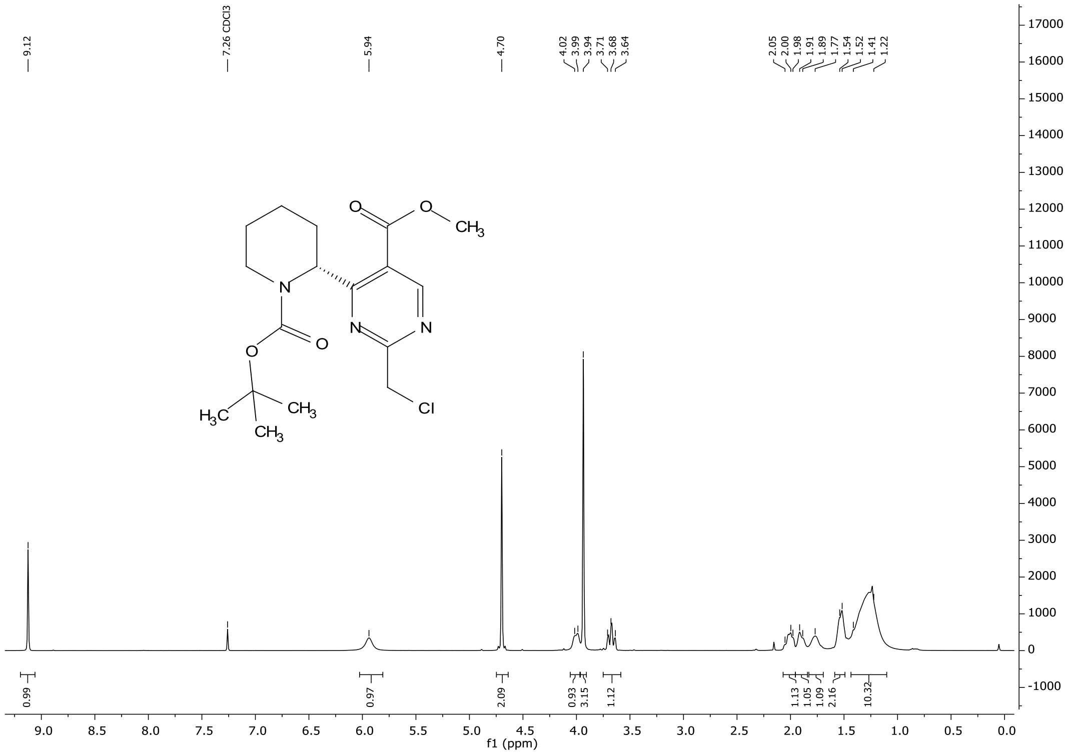

Figure S77. ^{1}H NMR (400 MHz, $CDCl_3$) spectrum of methyl 4-[(2*R*)-1-(*tert*-butoxycarbonyl)piperidin-2-yl]-2-(chloromethyl)pyrimidine-5-carboxylate (**6c**)

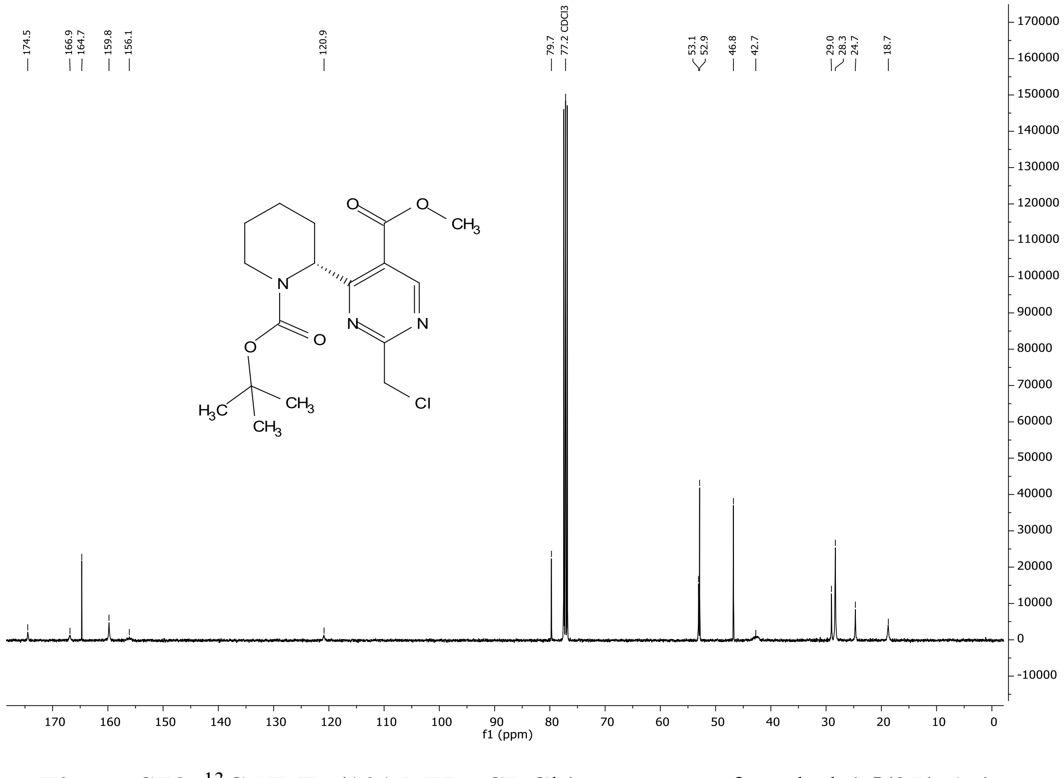

Figure S78. ^{13}C NMR (101 MHz, $CDCl_3$) spectrum of methyl 4-[(2*R*)-1-(*tert*-butoxycarbonyl)piperidin-2-yl]-2-(chloromethyl)pyrimidine-5-carboxylate (**6c**)

Compound Spectrum SmartFormula Report

Analysis Info

Analysis Name	D:\Data\GR-P-3090.d	Acquisition Date	3/23/2026 10:46:59 AM
Method	DirectInfusion_TuneLow_pos.m	Operator	hplc
Sample Name	GR-P-3090	Instrument	micrOTOF-Q III 8228888.20448
Comment	SB		

Acquisition Parameter

Source Type	ESI	Ion Polarity	Positive	Set Nebulizer	0.4 Bar
Focus	Not active	Set Capillary	4500 V	Set Dry Heater	180 °C
Scan Begin	50 m/z	Set End Plate Offset	-500 V	Set Dry Gas	4.0 l/min
Scan End	1000 m/z	Set Collision Cell RF	140.0 Vpp	Set Divert Valve	Waste

#	RT [min]	Area	Int. Type	I	S/N	Chromatogram	Max. m/z	FWHM [min]
n.a.	1.0	n.a.	Single spectrum	n.a.	n.a.	n.a.	304.2617	n.a.
n.a.	4.6	n.a.	Single spectrum	n.a.	n.a.	n.a.	370.1528	n.a.

+MS, 4.6min #274

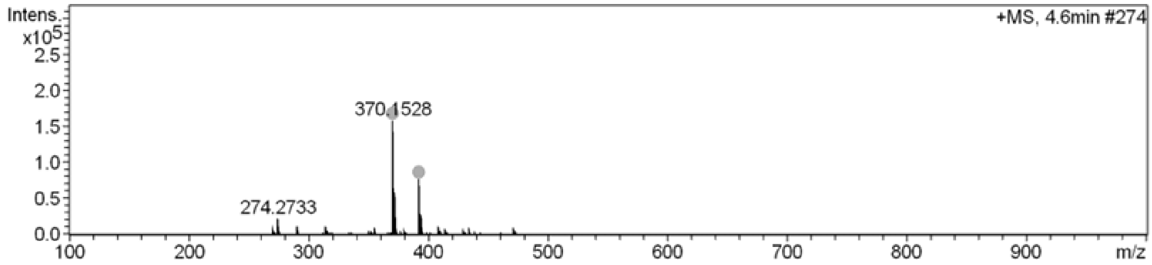

Meas. m/z	#	Ion Formula	m/z	err [ppm]	mSigma	# Sigma	Score	rdb	e⁻ Conf	N-Rule
370.1528	1	C17H25ClN3O4	370.1528	0.1	11.0	1	100.00	6.5	even	ok
392.1358	1	C17H24ClN3NaO4	392.1348	-2.6	7.8	1	100.00	6.5	even	ok

Figure S79. HRMS (ESI-TOF) spectrum of methyl 4-[(2*R*)-1-(*tert*-butoxycarbonyl)piperidin-2-yl]-2-(chloromethyl)pyrimidine-5-carboxylate (**6c**)

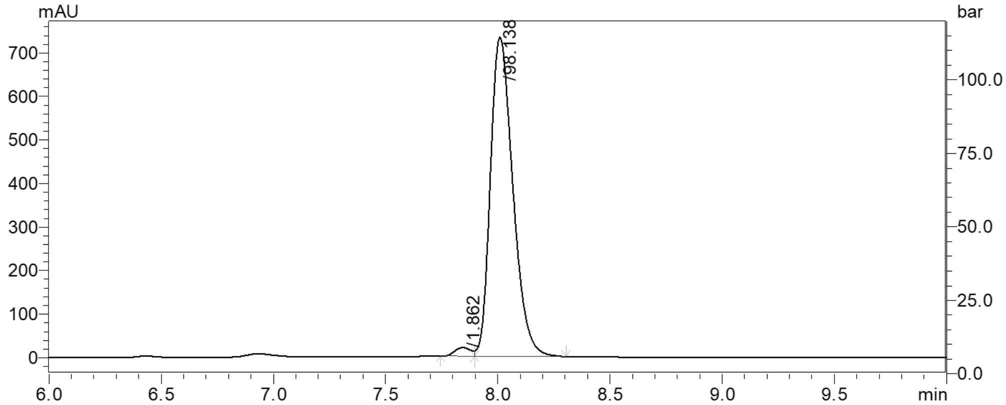

Figure S80. Chiral HPLC analysis of methyl 4-[(2*R*)-1-(*tert*-butoxycarbonyl)piperidin-2-yl]-2-(chloromethyl)pyrimidine-5-carboxylate (**6c**). Conditions: CHIRAL ART Amylose-SA (100 × 4.6 mm I.D.); S-10 μL; mobile phase: ACN/H_2O + 0.1% HCOOH (70:30→30:70 in 10 min); T = 36 ˚C; flow rate 1.0 mL/min.

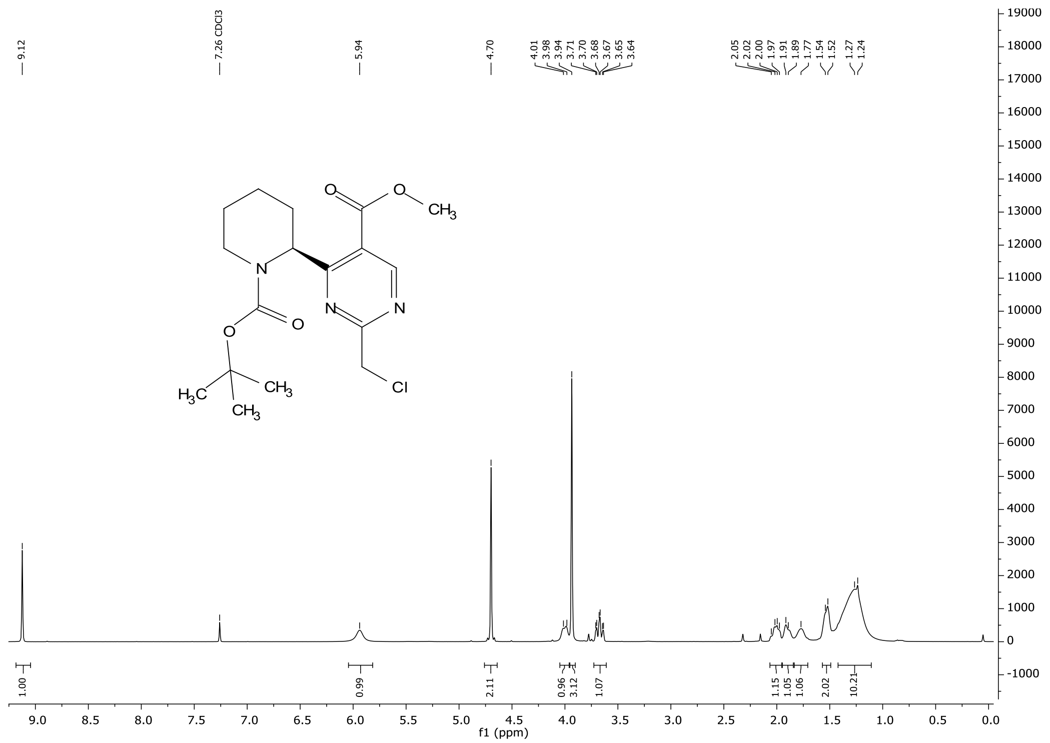

Figure S81. ^{1}H NMR (400 MHz, $CDCl_3$) spectrum of methyl 4-[(2*S*)-1-(*tert*-butoxycarbonyl)piperidin-2-yl]-2-(chloromethyl)pyrimidine-5-carboxylate (**6d**)

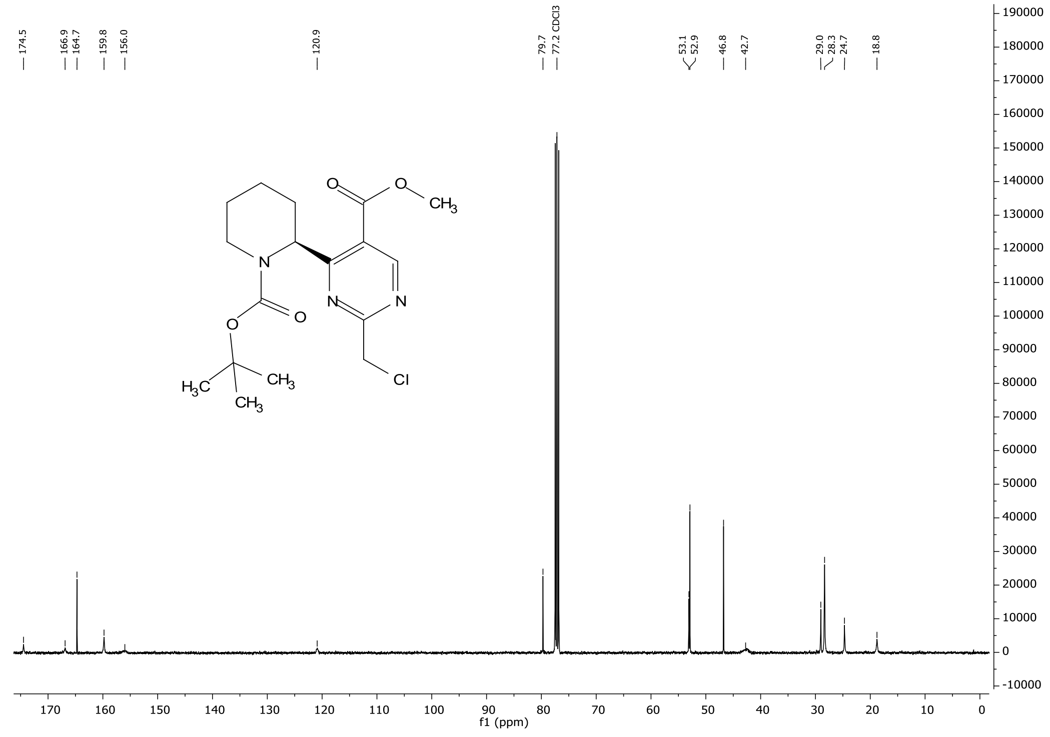

Figure S82. ^{13}C NMR (101 MHz, $CDCl_3$) spectrum of methyl 4-[(2*S*)-1-(*tert*-butoxycarbonyl)piperidin-2-yl]-2-(chloromethyl)pyrimidine-5-carboxylate (**6d**)

Compound Spectrum SmartFormula Report

Analysis Info			
Analysis Name	D:\Data\GR-P-3085.d	Acquisition Date	3/18/2026 3:02:37 PM
Method	DirectInfusion_TuneLow_pos.m	Operator	hplc
Sample Name	GR-P-3085	Instrument	micrOTOF-Q III 8228888.20448
Comment	SB		

Acquisition Parameter

Source Type	ESI	Ion Polarity	Positive	Set Nebulizer	0.4 Bar
Focus	Not active	Set Capillary	4500 V	Set Dry Heater	180 °C
Scan Begin	50 m/z	Set End Plate Offset	-500 V	Set Dry Gas	4.0 l/min
Scan End	1000 m/z	Set Collision Cell RF	140.0 Vpp	Set Divert Valve	Waste

#	RT [min]	Area	Int. Type	I	S/N	Chromatogram	Max. m/z	FWHM [min]
n.a.	2.7	n.a.	Single spectrum	n.a.	n.a.	n.a.	304.2617	n.a.
n.a.	6.6	n.a.	Single spectrum	n.a.	n.a.	n.a.	392.1355	n.a.

+MS, 6.6min #397

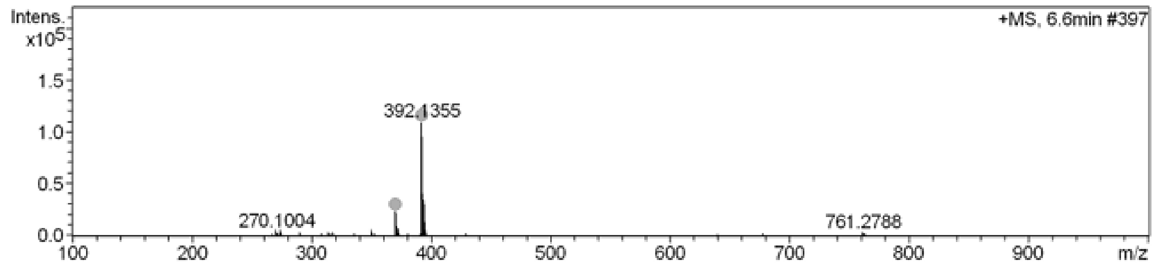

Meas. m/z	#	Ion Formula	m/z	err [ppm]	mSigma	# Sigma	Score	rdb	e⁻ Conf	N-Rule
370.1529	1	C17H25ClN3O4	370.1528	-0.4	14.1	1	100.00	6.5	even	ok
392.1355	1	C17H24ClN3NaO4	392.1348	2.0	12.9	1	100.00	6.5	even	ok

Figure S83. HRMS (ESI-TOF) spectrum of methyl 4-[(2*S*)-1-(*tert*-butoxycarbonyl)piperidin-2-yl]-2-(chloromethyl)pyrimidine-5-carboxylate (**6d**)

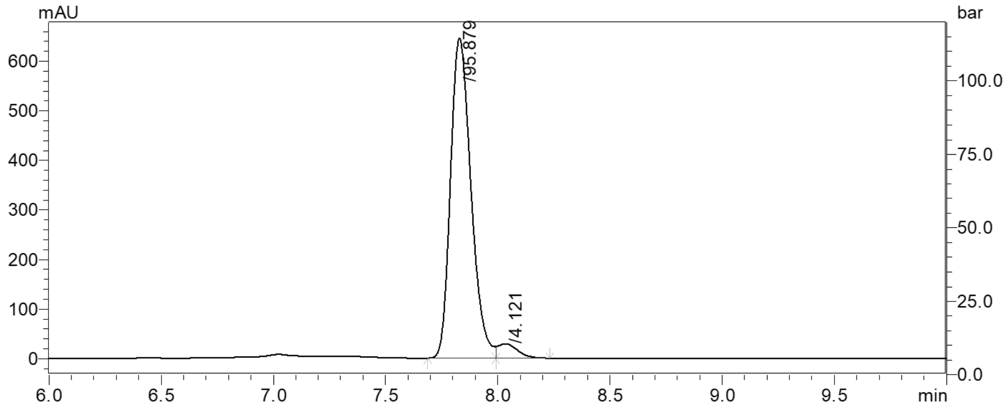

Figure S84. Chiral HPLC analysis of methyl 4-[(2*S*)-1-(*tert*-butoxycarbonyl)piperidin-2-yl]-2-(chloromethyl)pyrimidine-5-carboxylate (**6d**). Conditions: CHIRAL ART Amylose-SA (100 × 4.6 mm I.D.); S-10 µL; mobile phase: ACN/H_2O + 0.1% HCOOH (70:30→30:70 in 10 min); T = 36 ˚C; flow rate 1.0 mL/min.

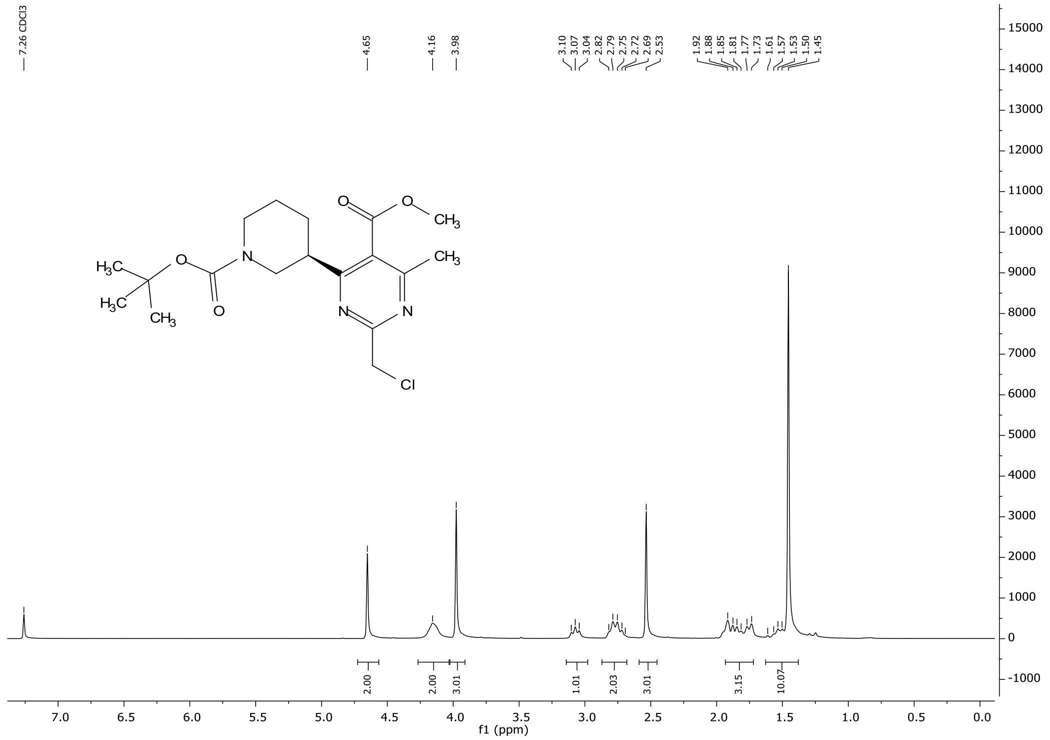

Figure S85. ^{1}H NMR (400 MHz, $CDCl_3$) spectrum of methyl 4-[(3*R*)-1-(*tert*-butoxycarbonyl)piperidin-3-yl]-2-(chloromethyl)-6-methylpyrimidine-5-carboxylate (**9a**)

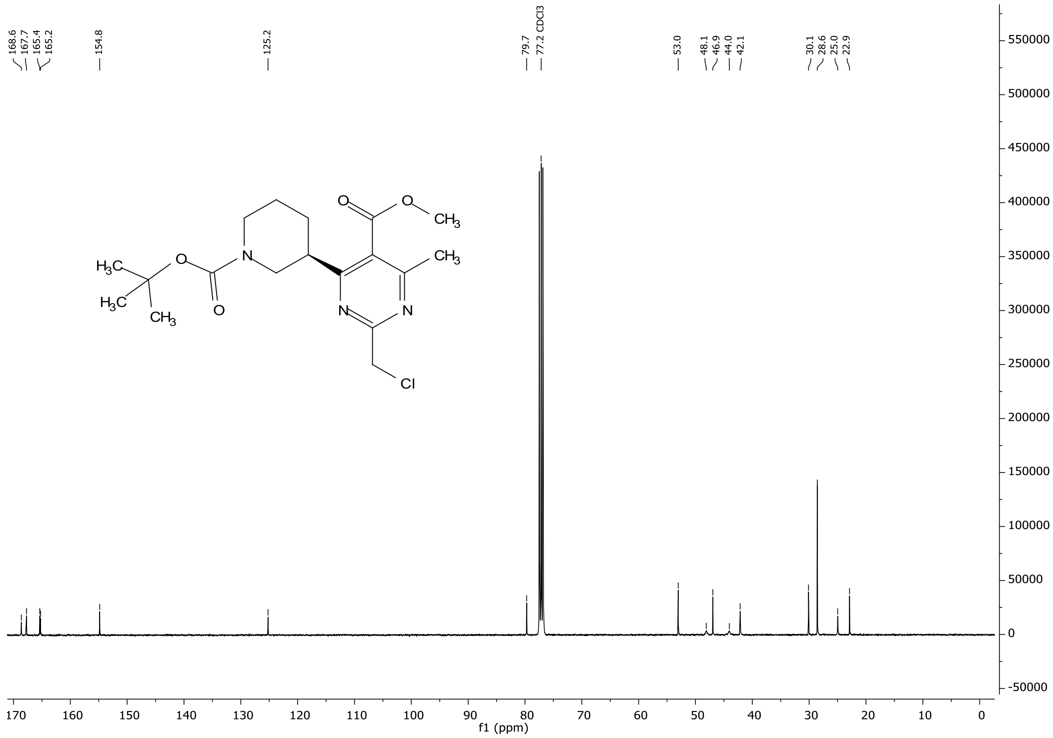

Figure S86. ^{13}C NMR (101 MHz, $CDCl_3$) spectrum of methyl 4-[(3*R*)-1-(*tert*-butoxycarbonyl)piperidin-3-yl]-2-(chloromethyl)-6-methylpyrimidine-5-carboxylate (**9a**)

Compound Spectrum SmartFormula Report

Analysis Info

Analysis Name	D:\Data\PVP-874.d	Acquisition Date	4/21/2026 9:42:08 AM
Method	DirectInfusion_TuneLow_pos.m	Operator	hplc
Sample Name	PVP-874	Instrument	micrOTOF-Q III 8228888.20448
Comment	SB		

Acquisition Parameter

Source Type	ESI	Ion Polarity	Positive	Set Nebulizer	0.4 Bar
Focus	Not active	Set Capillary	4500 V	Set Dry Heater	180 °C
Scan Begin	50 m/z	Set End Plate Offset	-500 V	Set Dry Gas	4.0 l/min
Scan End	1000 m/z	Set Collision Cell RF	140.0 Vpp	Set Divert Valve	Waste

#	RT [min]	Area	Int. Type	I	S/N	Chromatogram	Max. m/z	FWHM [min]
n.a.	1.7	n.a.	Single spectrum	n.a.	n.a.	n.a.	304.2612	n.a.
n.a.	12.1	n.a.	Single spectrum	n.a.	n.a.	n.a.	406.1506	n.a.

+MS, 12.1min #726

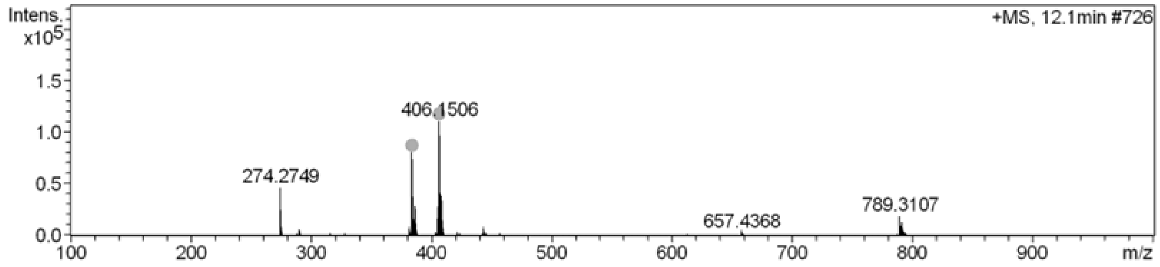

Meas. m/z	#	Ion Formula	m/z	err [ppm]	mSigma	# Sigma	Score	rdb	e⁻ Conf	N-Rule
384.1686	1	C18H27ClN3O4	384.1685	-0.2	4.9	1	100.00	6.5	even	ok
406.1506	1	C18H26ClN3NaO4	406.1504	0.6	3.4	1	100.00	6.5	even	ok

Figure S87. HRMS (ESI-TOF) spectrum of methyl 4-[(3*R*)-1-(*tert*-butoxycarbonyl)piperidin-3-yl]-2-(chloromethyl)-6-methylpyrimidine-5-carboxylate (**9a**)

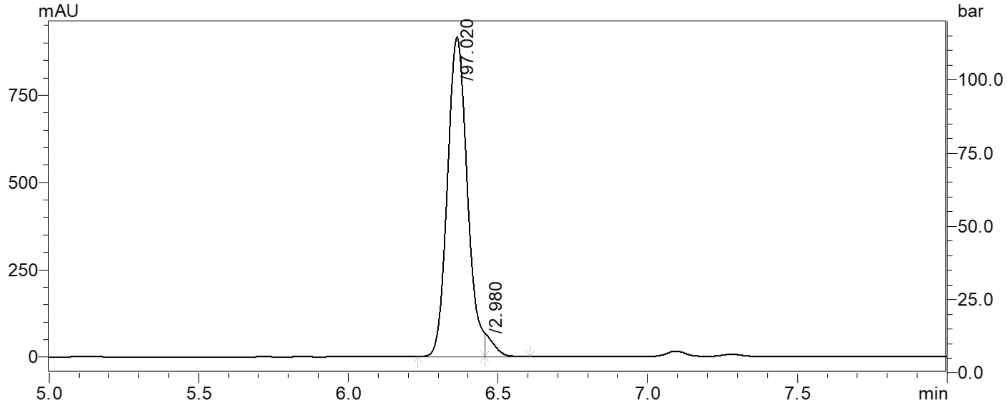

Figure S88. Chiral HPLC analysis of methyl 4-[(3*R*)-1-(*tert*-butoxycarbonyl)piperidin-3-yl]-2-(chloromethyl)-6-methylpyrimidine-5-carboxylate (**9a**). Conditions: CHIRAL ART Cellulose-SJ (100 × 4.6 mm I.D.); S-10 µL; mobile phase: ACN/H_2O + 0.1% HCOOH (70:30→30:70 in 10 min); T = 36 ˚C; flow rate 1.0 mL/min.

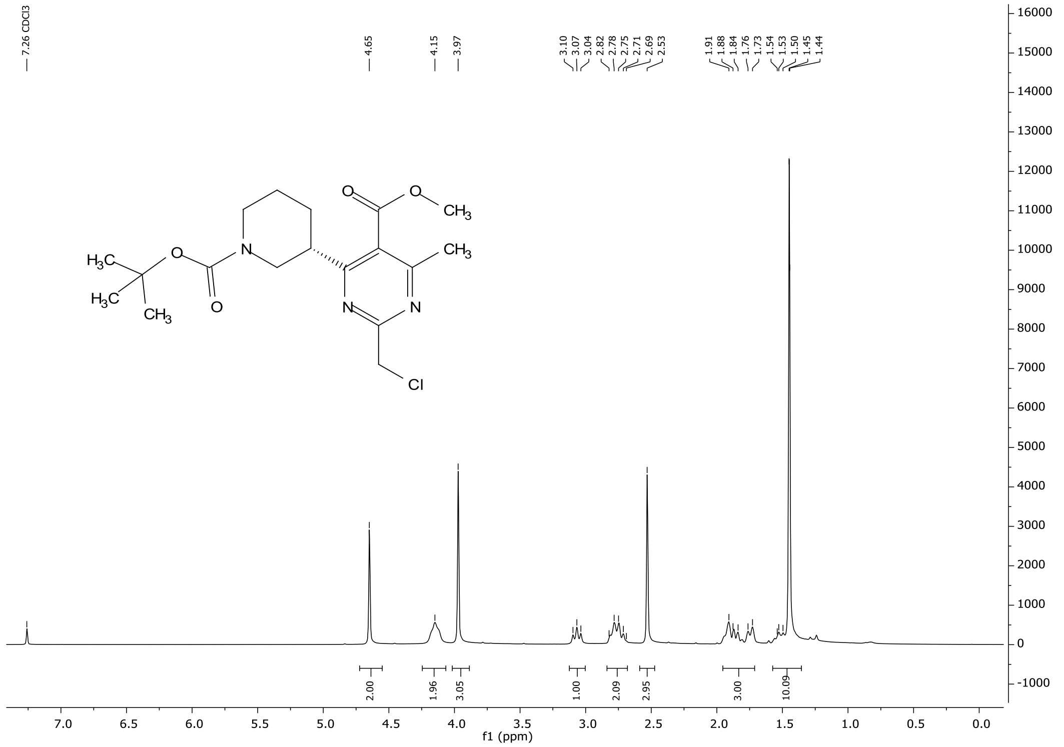

Figure S89. ^{1}H NMR (400 MHz, $CDCl_3$) spectrum of methyl 4-[(3*S*)-1-(*tert*-butoxycarbonyl)piperidin-3-yl]-2-(chloromethyl)-6-methylpyrimidine-5-carboxylate (**9b**)

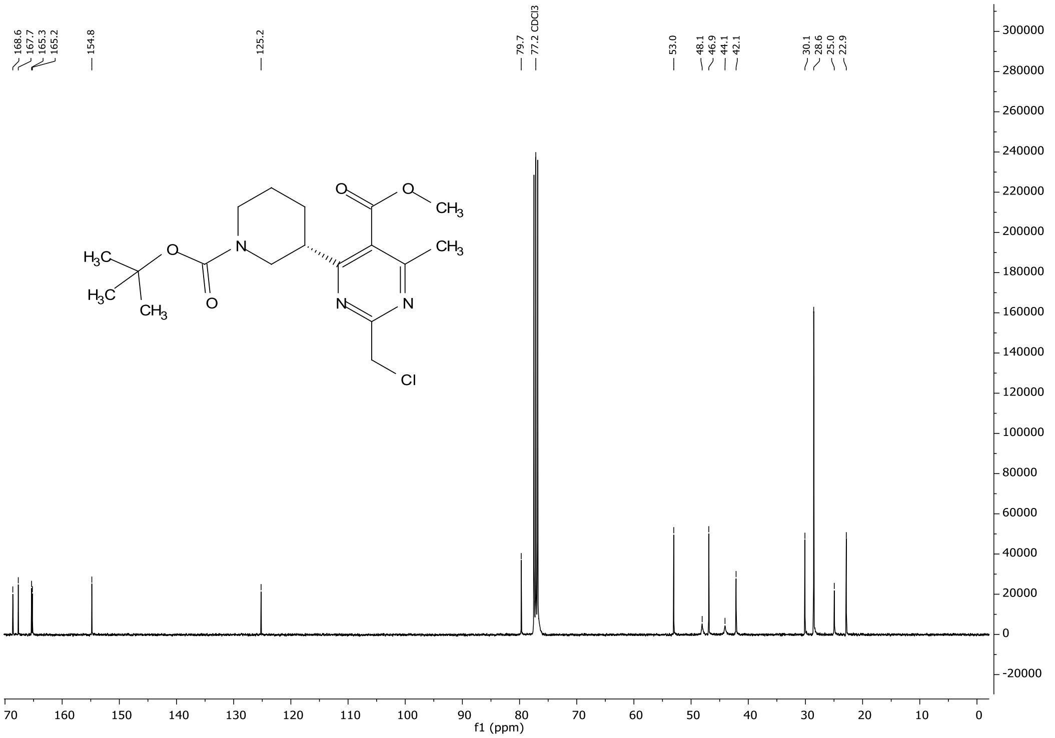

Figure S90. ^{13}C NMR (101 MHz, $CDCl_3$) spectrum of methyl 4-[(3*S*)-1-(*tert*-butoxycarbonyl)piperidin-3-yl]-2-(chloromethyl)-6-methylpyrimidine-5-carboxylate (**9b**)

Compound Spectrum SmartFormula Report

Analysis Info

Analysis Name	D:\Data\PVP-877.d	Acquisition Date	4/21/2026 10:02:31 AM
Method	DirectInfusion_TuneLow_pos.m	Operator	hplc
Sample Name	PVP-877	Instrument	micrOTOF-Q III 8228888.20448
Comment	SB		

Acquisition Parameter

Source Type	ESI	Ion Polarity	Positive	Set Nebulizer	0.4 Bar
Focus	Not active	Set Capillary	4500 V	Set Dry Heater	180 °C
Scan Begin	50 m/z	Set End Plate Offset	-500 V	Set Dry Gas	4.0 l/min
Scan End	1000 m/z	Set Collision Cell RF	140.0 Vpp	Set Divert Valve	Waste

#	RT [min]	Area	Int. Type	I	S/N	Chromatogram	Max. m/z	FWHM [min]
n.a.	0.8	n.a.	Single spectrum	n.a.	n.a.	n.a.	304.2619	n.a.
n.a.	6.9	n.a.	Single spectrum	n.a.	n.a.	n.a.	384.1685	n.a.

+MS, 6.9min #416

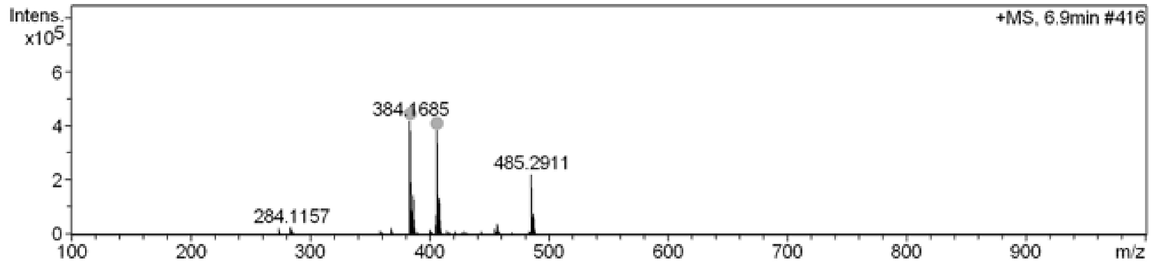

Meas. m/z	#	Ion Formula	m/z	err [ppm]	mSigma	# Sigma	Score	rdb	e⁻ Conf	N-Rule
384.1685	1	C18H27ClN3O4	384.1685	0.1	5.9	1	100.00	6.5	even	ok
406.1508	1	C18H26ClN3NaO4	406.1504	1.0	3.7	1	100.00	6.5	even	ok

Figure S91. HRMS (ESI-TOF) spectrum of methyl 4-[(3*S*)-1-(*tert*-butoxycarbonyl)piperidin-3-yl]-2-(chloromethyl)-6-methylpyrimidine-5-carboxylate (**9b**)

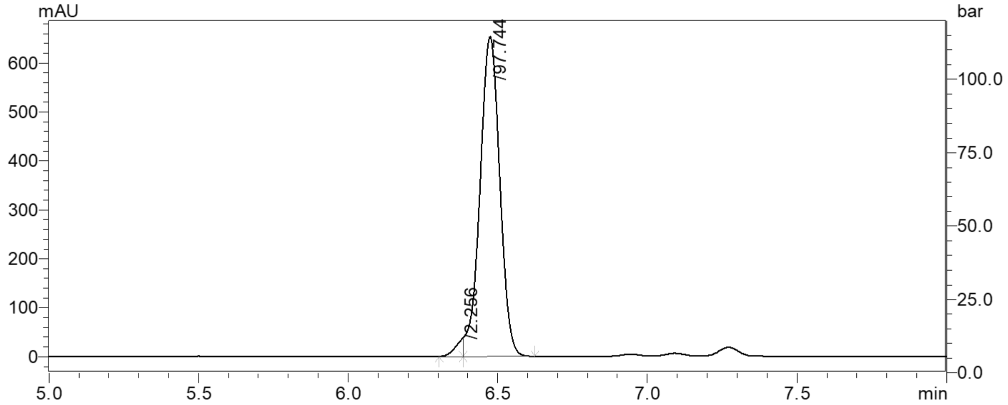

Figure S92. Chiral HPLC analysis of methyl 4-[(3*S*)-1-(*tert*-butoxycarbonyl)piperidin-3-yl]-2-(chloromethyl)-6-methylpyrimidine-5-carboxylate (**9b**). Conditions: CHIRAL ART Cellulose-SJ (100 × 4.6 mm I.D.); S-10 µL; mobile phase: ACN/H_2O + 0.1% HCOOH (70:30→30:70 in 10 min); T = 36 ˚C; flow rate 1.0 mL/min.

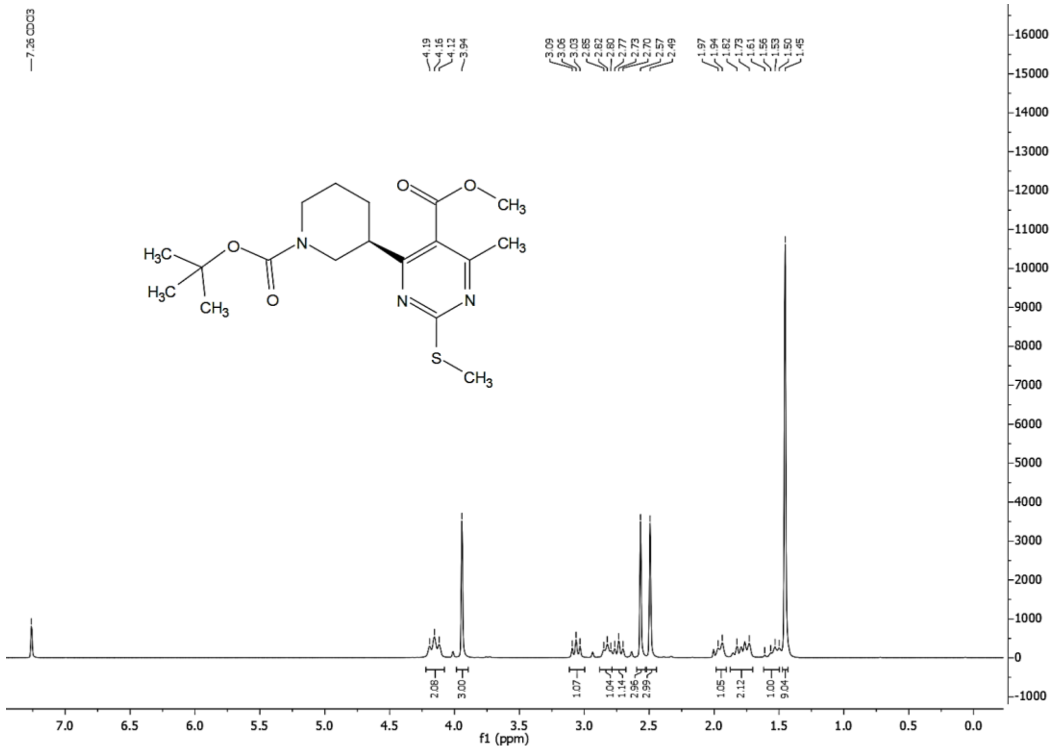

Figure S93. ^{1}H NMR (400 MHz, $CDCl_3$) spectrum of methyl 4-[(3*R*)-1-(*tert*-butoxycarbonyl)piperidin-3-yl]-6-methyl-2-(methylsulfanyl)pyrimidine-5-carboxylate (**9c**)

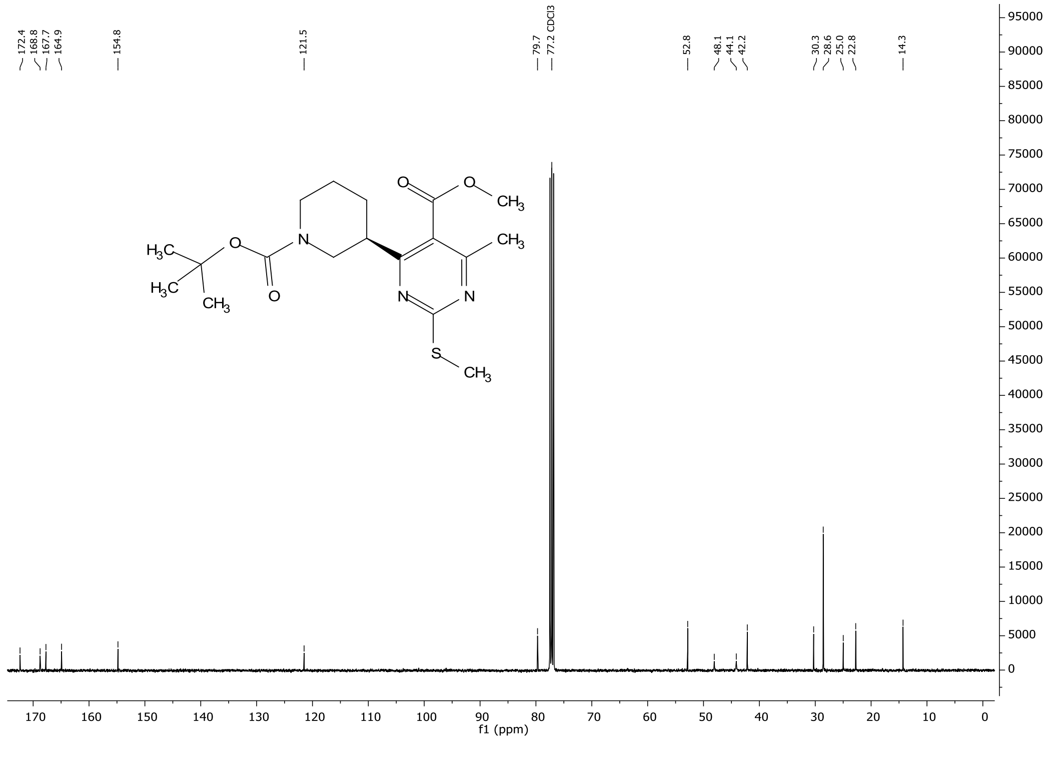

Figure S94. ^{13}C NMR (101 MHz, $CDCl_3$) spectrum of methyl 4-[(3*R*)-1-(*tert*-butoxycarbonyl)piperidin-3-yl]-6-methyl-2-(methylsulfanyl)pyrimidine-5-carboxylate (**9c**)

Compound Spectrum SmartFormula Report

Analysis Info

Analysis Name	D:\Data\GR-P-3060.d	Acquisition Date	3/10/2026 11:55:25 AM
Method	DirectInfusion_TuneLow_pos.m	Operator	hplc
Sample Name	GR-P-3060	Instrument	micrOTOF-Q III 8228888.20448
Comment	SB		

Acquisition Parameter

Source Type	ESI	Ion Polarity	Positive	Set Nebulizer	0.4 Bar
Focus	Not active	Set Capillary	4500 V	Set Dry Heater	180 °C
Scan Begin	50 m/z	Set End Plate Offset	-500 V	Set Dry Gas	4.0 l/min
Scan End	1000 m/z	Set Collision Cell RF	140.0 Vpp	Set Divert Valve	Waste

#	RT [min]	Area	Int. Type	I	S/N	Chromatogram	Max. m/z	FWHM [min]
n.a.	0.7	n.a.	Single spectrum	n.a.	n.a.	n.a.	304.2611	n.a.
n.a.	6.3	n.a.	Single spectrum	n.a.	n.a.	n.a.	382.1795	n.a.

+MS, 6.3min #377

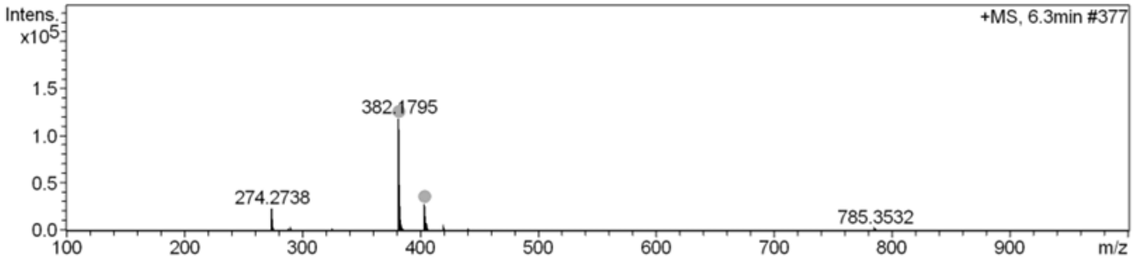

Meas. m/z	#	Ion Formula	m/z	err [ppm]	mSigma	# Sigma	Score	rdb	e⁻ Conf	N-Rule
382.1795	1	C18H28N3O4S	382.1795	0.1	11.8	1	100.00	6.5	even	ok
404.1627	1	C18H27N3NaO4S	404.1614	3.1	13.7	1	100.00	6.5	even	ok

Figure S95. HRMS (ESI-TOF) spectrum of methyl 4-[(3*R*)-1-(*tert*-butoxycarbonyl)piperidin-3-yl]-6-methyl-2-(methylsulfanyl)pyrimidine-5-carboxylate (**9c**)

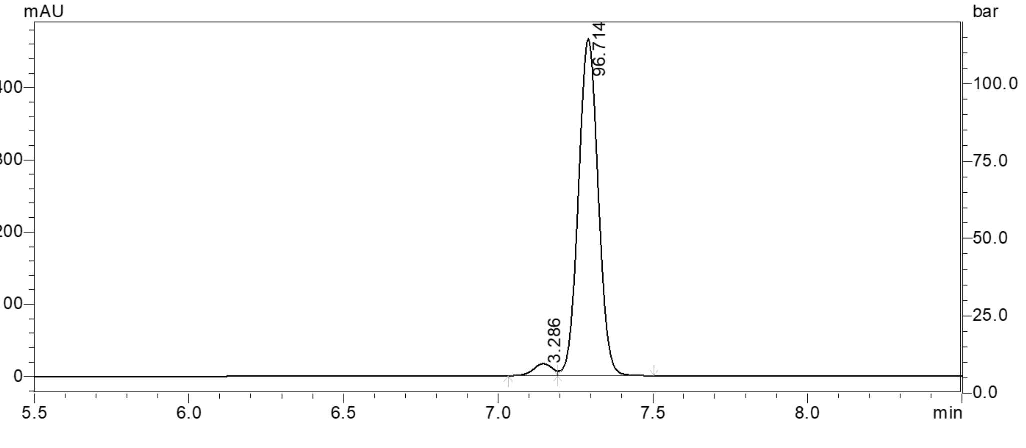

Figure S96. Chiral HPLC analysis of methyl 4-[(3*R*)-1-(*tert*-butoxycarbonyl)piperidin-3-yl]-6-methyl-2-(methylsulfanyl)pyrimidine-5-carboxylate (**9c**). Conditions: CHIRAL ART Cellulose-SJ (100 × 4.6 mm I.D.); S-10 µL; mobile phase: ACN/H_2O + 0.1% HCOOH (70:30→30:70 in 10 min); T = 36 °C; flow rate 1.0 mL/min.

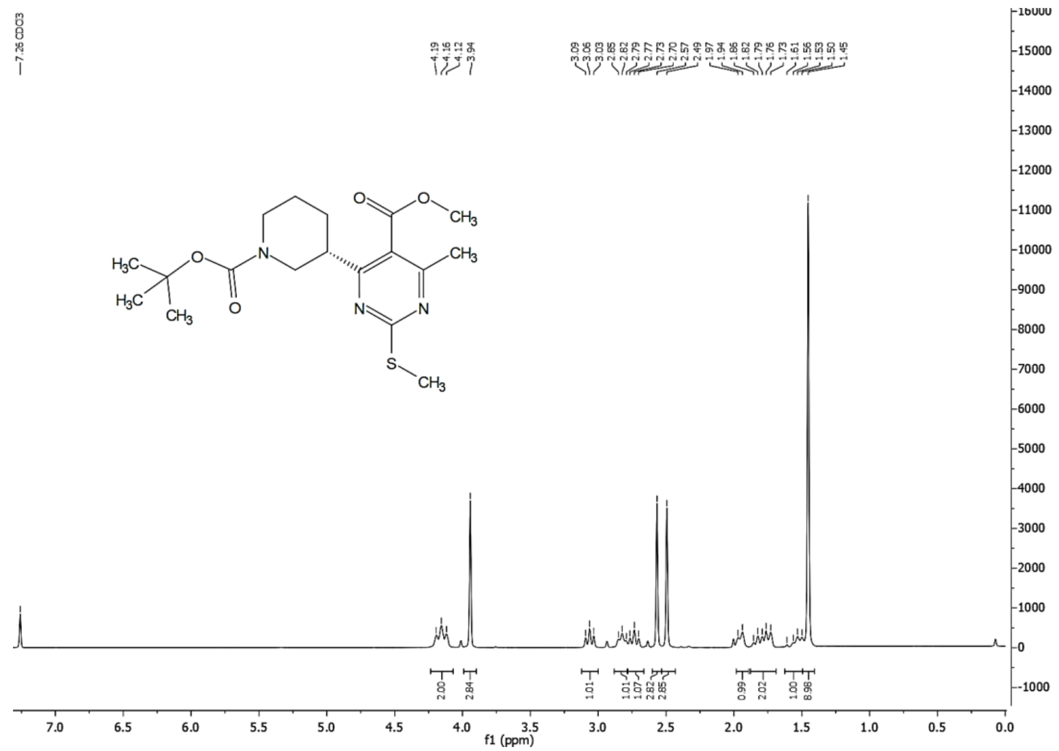

Figure S97. ^{1}H NMR (400 MHz, $CDCl_3$) spectrum of methyl 4-[(3*S*)-1-(*tert*-butoxycarbonyl)piperidin-3-yl]-6-methyl-2-(methylsulfanyl)pyrimidine-5-carboxylate (**9d**)

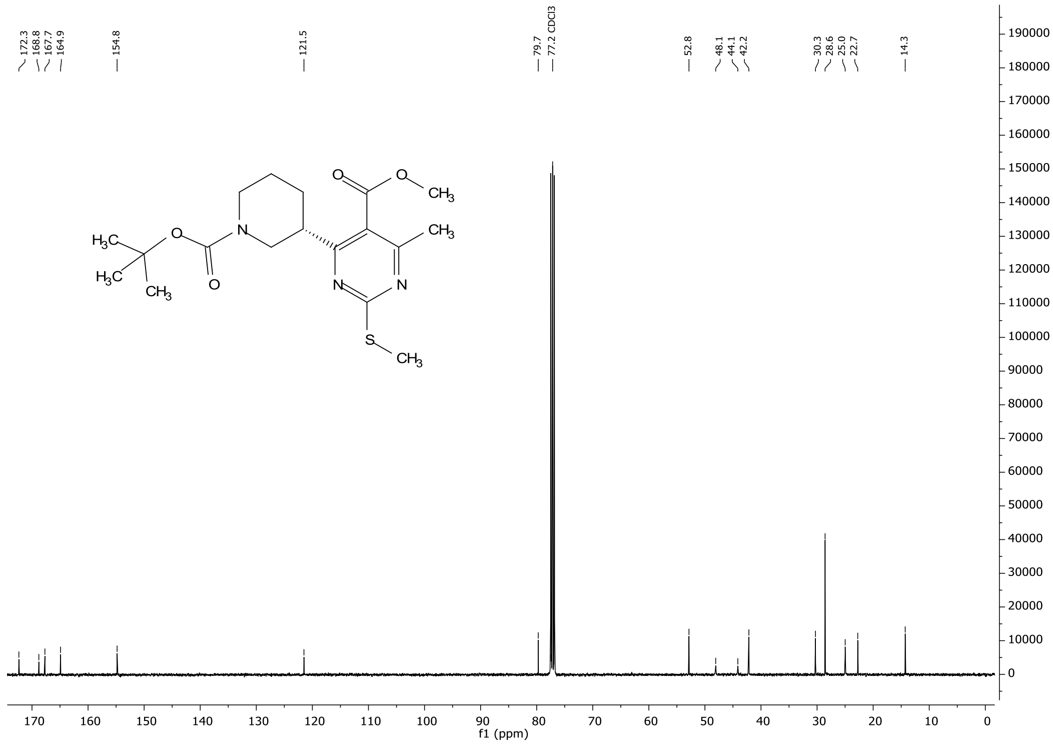

Figure S98. ^{13}C NMR (101 MHz, $CDCl_3$) spectrum of methyl 4-[(3*S*)-1-(*tert*-butoxycarbonyl)piperidin-3-yl]-6-methyl-2-(methylsulfanyl)pyrimidine-5-carboxylate (**9d**)

Compound Spectrum SmartFormula Report

Analysis Info

Analysis Name	D:\Data\GR-P-3063.d	Acquisition Date	3/17/2026 12:09:44 PM
Method	DirectInfusion_TuneLow_pos.m	Operator	hplc
Sample Name	GR-P-3063	Instrument	micrOTOF-Q III 8228888.20448
Comment	SB		

Acquisition Parameter

Source Type	ESI	Ion Polarity	Positive	Set Nebulizer	0.4 Bar
Focus	Not active	Set Capillary	4500 V	Set Dry Heater	180 °C
Scan Begin	50 m/z	Set End Plate Offset	-500 V	Set Dry Gas	4.0 l/min
Scan End	1000 m/z	Set Collision Cell RF	140.0 Vpp	Set Divert Valve	Waste

#	RT [min]	Area	Int. Type	I	S/N	Chromatogram	Max. m/z	FWHM [min]
n.a.	0.1	n.a.	Single spectrum	n.a.	n.a.	n.a.	304.2610	n.a.
n.a.	3.4	n.a.	Single spectrum	n.a.	n.a.	n.a.	382.1796	n.a.

+MS, 3.4min #204

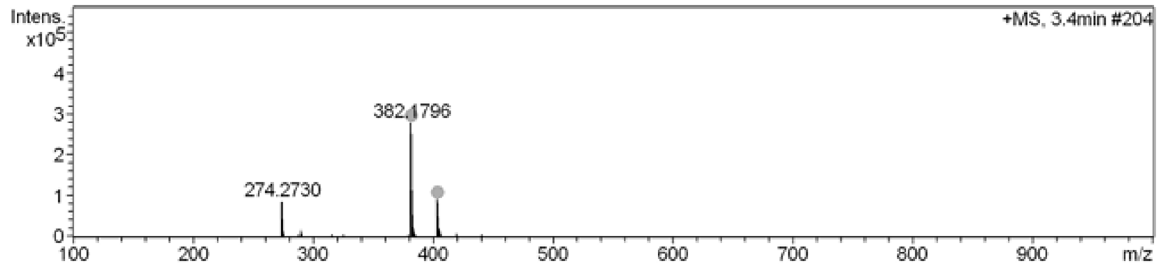

Meas. m/z	#	Ion Formula	m/z	err [ppm]	mSigma	# Sigma	Score	rdb	e⁻ Conf	N-Rule
382.1796	1	C18H28N3O4S	382.1795	0.3	2.9	1	100.00	6.5	even	ok
404.1613	1	C18H27N3NaO4S	404.1614	0.3	10.2	1	100.00	6.5	even	ok

Figure S99. HRMS (ESI-TOF) spectrum of methyl 4-[(3*S*)-1-(*tert*-butoxycarbonyl)piperidin-3-yl]-6-methyl-2-(methylsulfanyl)pyrimidine-5-carboxylate (**9d**)

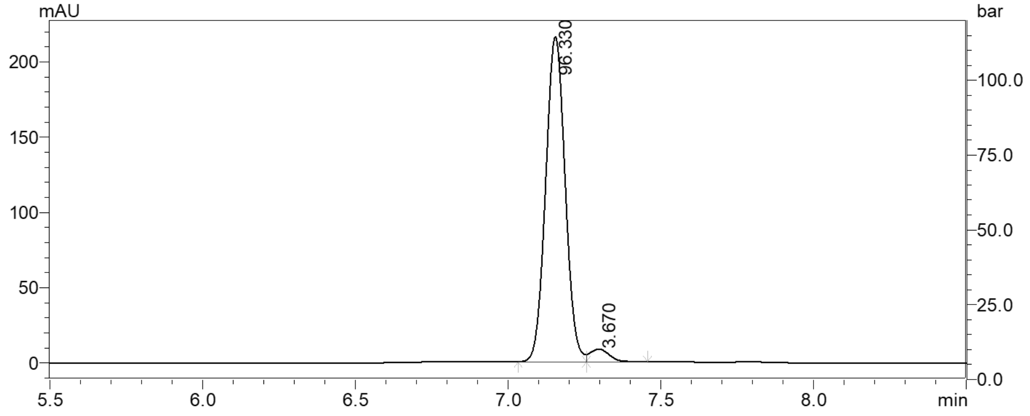

Figure S100. Chiral HPLC analysis of methyl 4-[(3*S*)-1-(*tert*-butoxycarbonyl)piperidin-3-yl]-6-methyl-2-(methylsulfanyl)pyrimidine-5-carboxylate (**9d**). Conditions: CHIRAL ART Cellulose-SJ (100 × 4.6 mm I.D.); S-10 µL; mobile phase: ACN/H_2O + 0.1% HCOOH (70:30→30:70 in 10 min); T = 36 ˚C; flow rate 1.0 mL/min.

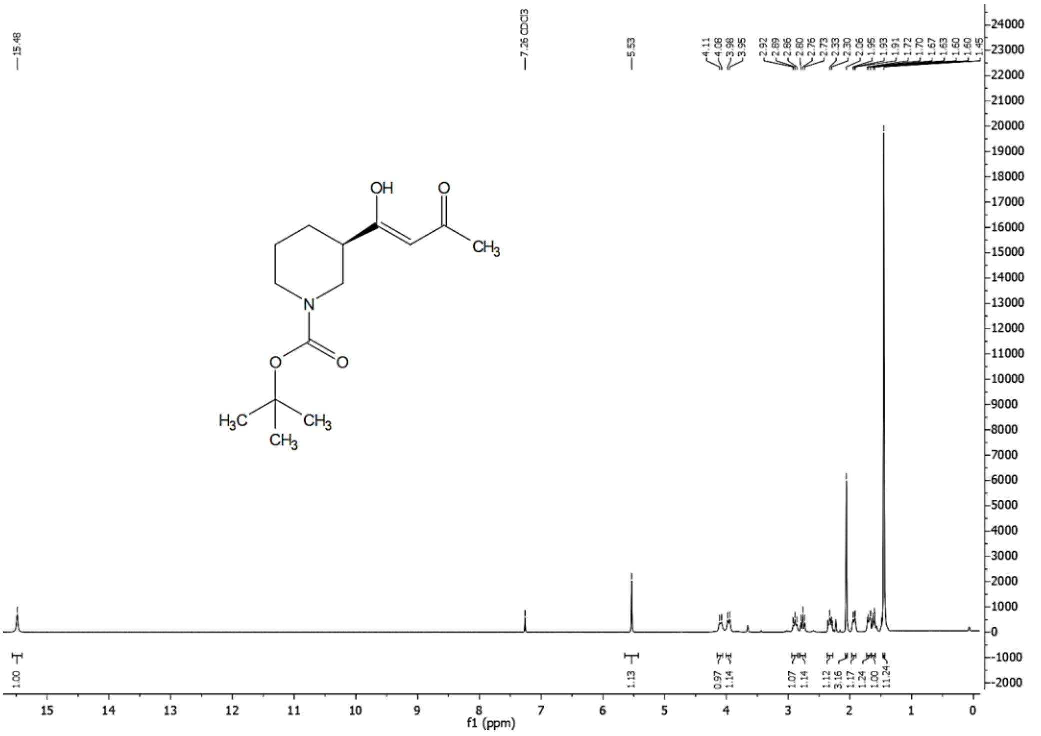

Figure S101. ^{1}H NMR (400 MHz, $CDCl_3$) spectrum of *tert*-butyl (3*R*)-3-[(1*Z*)-1-hydroxy-3-oxobut-1-en-1-yl]piperidine-1-carboxylate (**11a**)

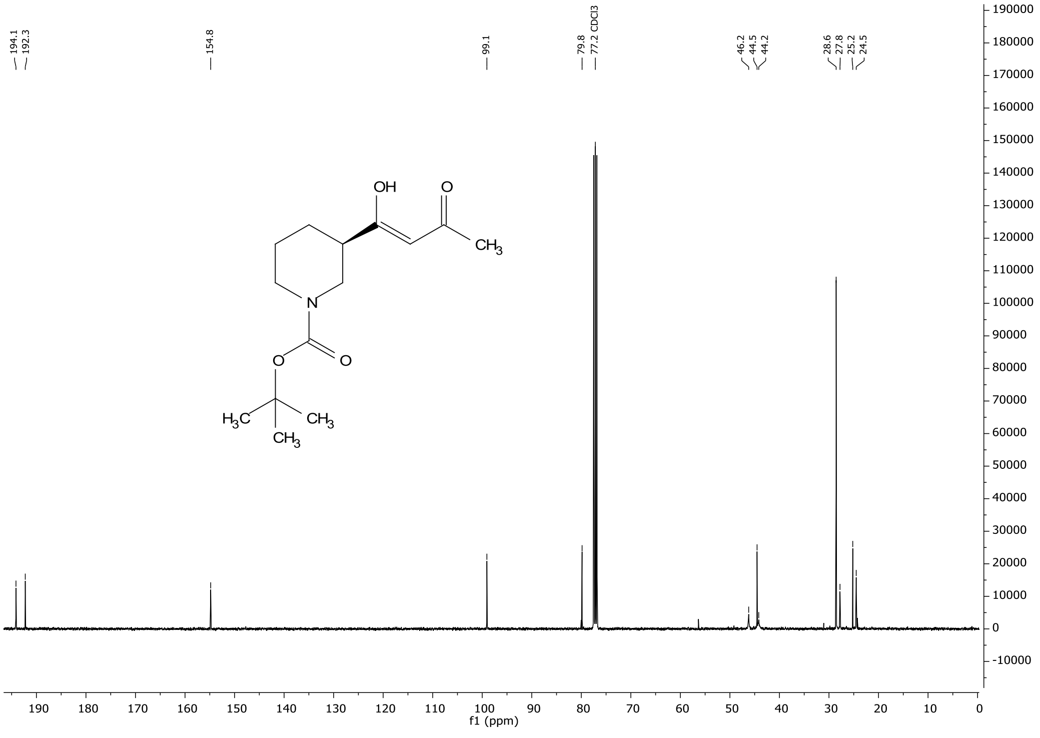

Figure S102. ^{13}C NMR (101 MHz, $CDCl_3$) spectrum of *tert*-butyl (3*R*)-3-[(1*Z*)-1-hydroxy-3-oxobut-1-en-1-yl]piperidine-1-carboxylate (**11a**)

Compound Spectrum SmartFormula Report

Analysis Info

Analysis Name	D:\Data\PVP-808.d	Acquisition Date	3/23/2026 10:08:47 AM
Method	DirectInfusion_TuneLow_pos.m	Operator	hplc
Sample Name	PVP-808	Instrument	micrOTOF-Q III 8228888.20448
Comment	SB		

Acquisition Parameter

Source Type	ESI	Ion Polarity	Positive	Set Nebulizer	0.4 Bar
Focus	Not active	Set Capillary	4500 V	Set Dry Heater	180 °C
Scan Begin	50 m/z	Set End Plate Offset	-500 V	Set Dry Gas	4.0 l/min
Scan End	1000 m/z	Set Collision Cell RF	140.0 Vpp	Set Divert Valve	Waste

#	RT [min]	Area	Int. Type	I	S/N	Chromatogram	Max. m/z	FWHM [min]
n.a.	1.2	n.a.	Single spectrum	n.a.	n.a.	n.a.	304.2616	n.a.
n.a.	3.5	n.a.	Single spectrum	n.a.	n.a.	n.a.	292.1519	n.a.

+MS, 3.5min #210

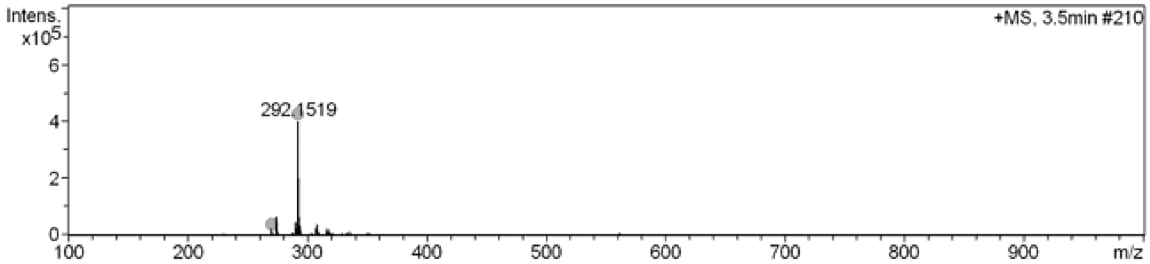

Meas. m/z	#	Ion Formula	m/z	err [ppm]	mSigma	# Sigma	Score	rdb	e⁻ Conf	N-Rule
270.1693	1	C14H24NO4	270.1700	2.5	41.0	1	100.00	3.5	even	ok
292.1519	1	C14H23NNaO4	292.1519	0.3	34.3	1	100.00	3.5	even	ok

Figure S103. HRMS (ESI-TOF) spectrum of *tert*-butyl (3*R*)-3-[(1*Z*)-1-hydroxy-3-oxobut-1-en-1-yl]piperidine-1-carboxylate (**11a**)

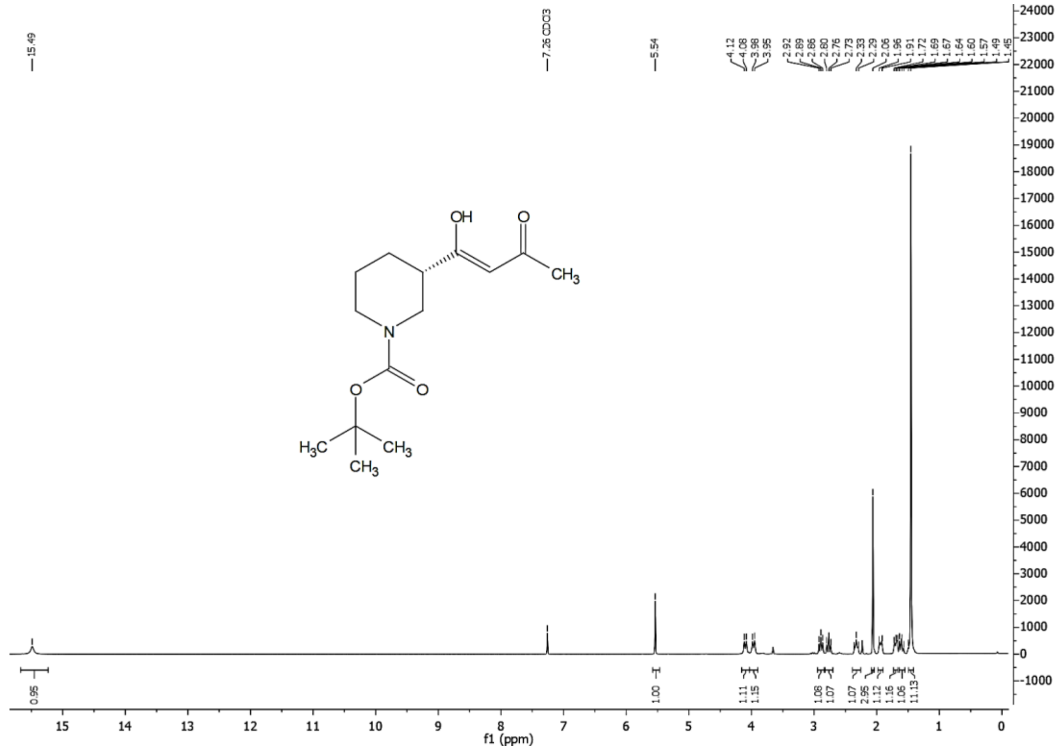

Figure S104. ^{1}H NMR (400 MHz, $CDCl_3$) spectrum of *tert*-butyl (3*S*)-3-[(1*Z*)-1-hydroxy-3-oxobut-1-en-1-yl]piperidine-1-carboxylate (**11b**)

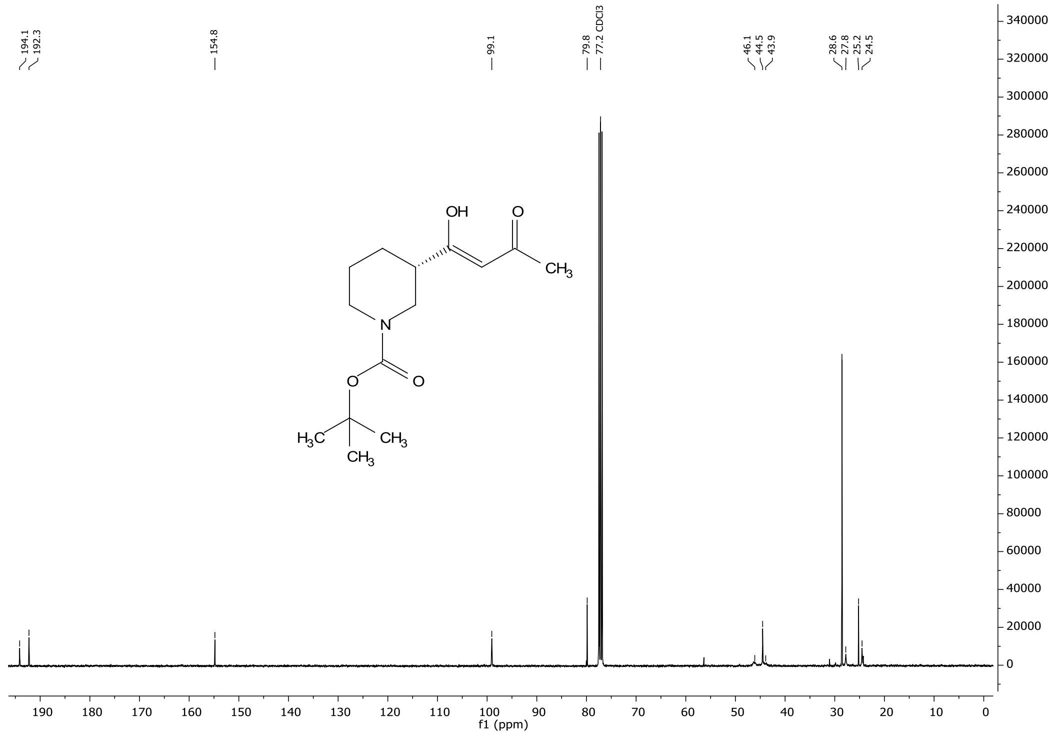

Figure S105. ^{13}C NMR (101 MHz, $CDCl_3$) spectrum of *tert*-butyl (3*S*)-3-[(1*Z*)-1-hydroxy-3-oxobut-1-en-1-yl]piperidine-1-carboxylate (**11b**)

Compound Spectrum SmartFormula Report

Analysis Info

Analysis Name	D:\Data\PVP-807.d	Acquisition Date	3/19/2026 10:34:20 AM
Method	DirectInfusion_TuneLow_pos.m	Operator	hplc
Sample Name	PVP-807	Instrument	micrOTOF-Q III 8228888.20448
Comment	SB		

Acquisition Parameter

Source Type	ESI	Ion Polarity	Positive	Set Nebulizer	0.4 Bar
Focus	Not active	Set Capillary	4500 V	Set Dry Heater	180 °C
Scan Begin	50 m/z	Set End Plate Offset	-500 V	Set Dry Gas	4.0 l/min
Scan End	1000 m/z	Set Collision Cell RF	140.0 Vpp	Set Divert Valve	Waste

#	RT [min]	Area	Int. Type	I	S/N	Chromatogram	Max. m/z	FWHM [min]
n.a.	0.1	n.a.	Single spectrum	n.a.	n.a.	n.a.	304.2617	n.a.
n.a.	6.2	n.a.	Single spectrum	n.a.	n.a.	n.a.	292.1519	n.a.

+MS, 6.2min #371

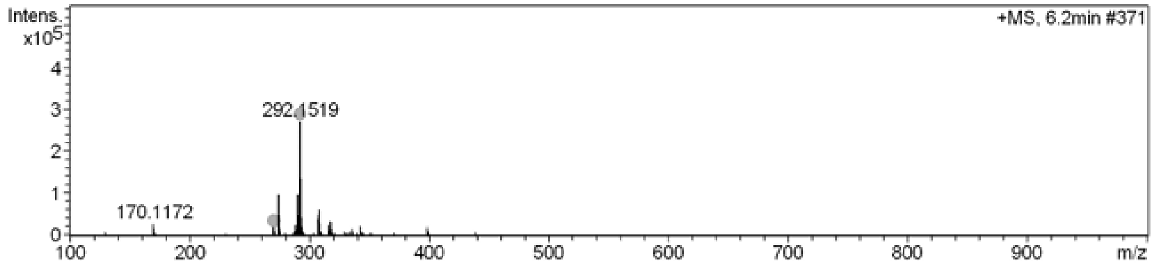

Meas. m/z	#	Ion Formula	m/z	err [ppm]	mSigma	# Sigma	Score	rdb	e⁻ Conf	N-Rule
270.1688	1	C14H24NO4	270.1700	-4.3	17.8	1	100.00	3.5	even	ok
292.1519	1	C14H23NNaO4	292.1519	-0.2	12.0	1	100.00	3.5	even	ok

Figure S106. HRMS (ESI-TOF) spectrum of *tert*-butyl (3*S*)-3-[(1*Z*)-1-hydroxy-3-oxobut-1-en-1-yl]piperidine-1-carboxylate (**11b**)

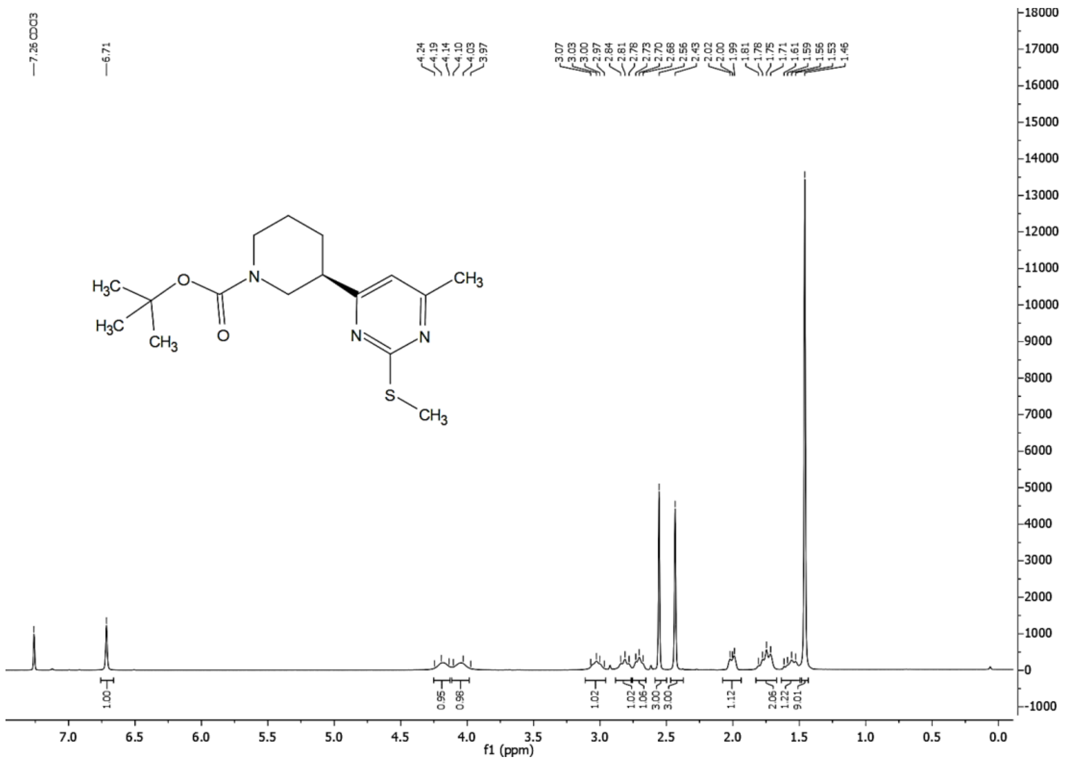

Figure S107. ^{1}H NMR (400 MHz, $CDCl_3$) spectrum of *tert*-butyl (3*R*)-3-[6-methyl-2-(methylsulfanyl)pyrimidin-4-yl]piperidine-1-carboxylate (**12a**)

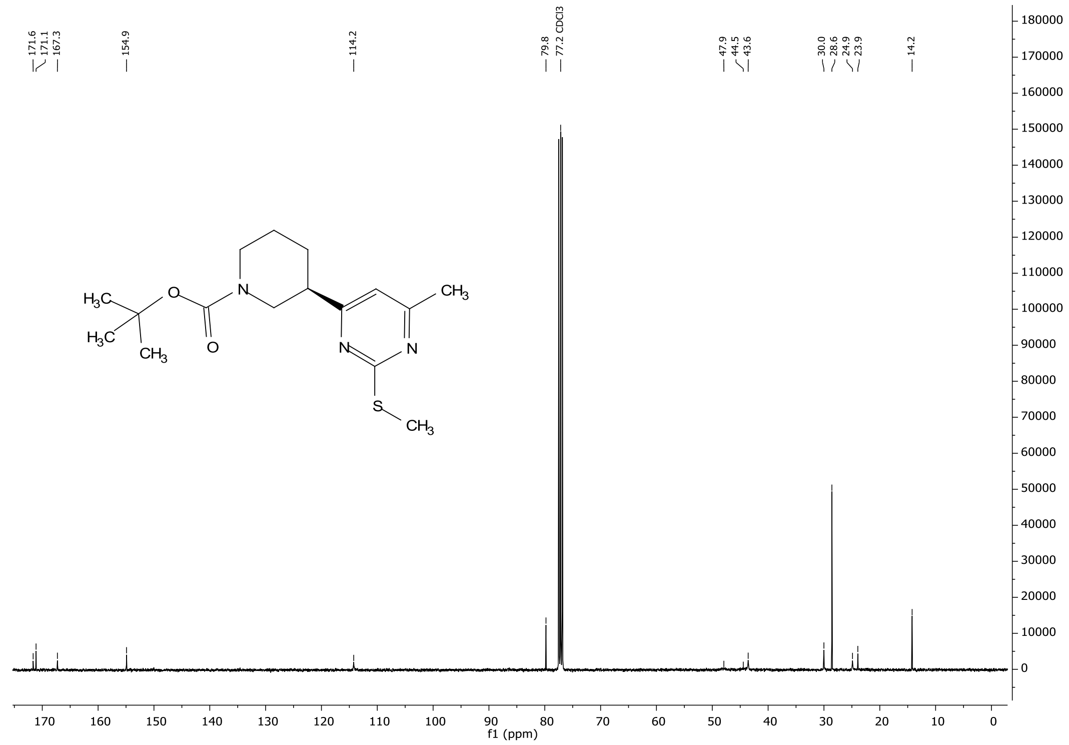

Figure S108. ^{13}C NMR (101 MHz, $CDCl_3$) spectrum of *tert*-butyl (3*R*)-3-[6-methyl-2-(methylsulfanyl)pyrimidin-4-yl]piperidine-1-carboxylate (**12a**)

Compound Spectrum SmartFormula Report

Analysis Info

Analysis Name	D:\Data\PVP-819.d	Acquisition Date	3/18/2026 2:34:46 PM
Method	DirectInfusion_TuneLow_pos.m	Operator	hplc
Sample Name	PVP-819	Instrument	micrOTOF-Q III 8228888.20448
Comment	SB		

Acquisition Parameter

Source Type	ESI	Ion Polarity	Positive	Set Nebulizer	0.4 Bar
Focus	Not active	Set Capillary	4500 V	Set Dry Heater	180 °C
Scan Begin	50 m/z	Set End Plate Offset	-500 V	Set Dry Gas	4.0 l/min
Scan End	1000 m/z	Set Collision Cell RF	140.0 Vpp	Set Divert Valve	Waste

#	RT [min]	Area	Int. Type	I	S/N	Chromatogram	Max. m/z	FWHM [min]
n.a.	0.1	n.a.	Single spectrum	n.a.	n.a.	n.a.	304.2619	n.a.
n.a.	3.6	n.a.	Single spectrum	n.a.	n.a.	n.a.	324.1740	n.a.

+MS, 3.6min #217

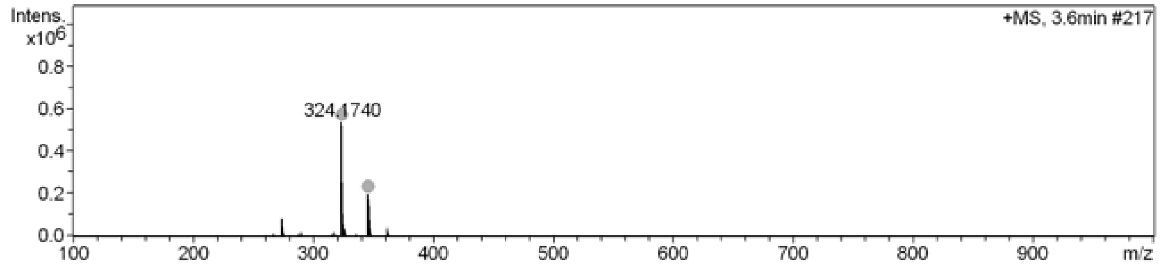

Meas. m/z	#	Ion Formula	m/z	err [ppm]	mSigma	# Sigma	Score	rdb	e⁻ Conf	N-Rule
324.1740	1	C16H26N3O2S	324.1740	-0.1	5.5	1	100.00	5.5	even	ok
346.1552	1	C16H25N3NaO2S	346.1560	-2.2	2.3	1	100.00	5.5	even	ok

Figure S109. HRMS (ESI-TOF) spectrum of *tert*-butyl (3*R*)-3-[6-methyl-2-(methylsulfanyl)pyrimidin-4-yl]piperidine-1-carboxylate (**12a**)

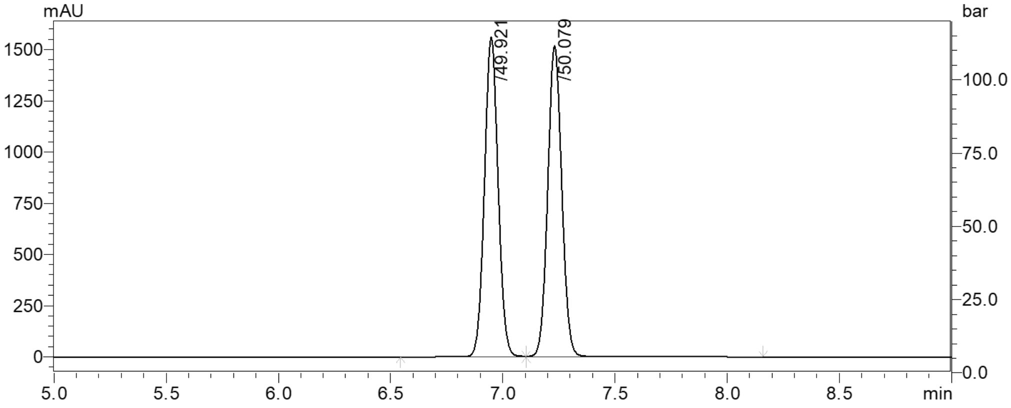

Figure S110. Chiral HPLC analysis of *tert*-butyl (3*R*)-3-[6-methyl-2-(methylsulfanyl)pyrimidin-4-yl]piperidine-1-carboxylate (**12a**). Conditions: CHIRAL ART Cellulose-SJ (100 × 4.6 mm I.D.); S-10 μL; mobile phase: ACN/H_2O + 0.1% HCOOH (70:30→30:70 in 10 min); T = 36 °C; flow rate 1.0 mL/min.

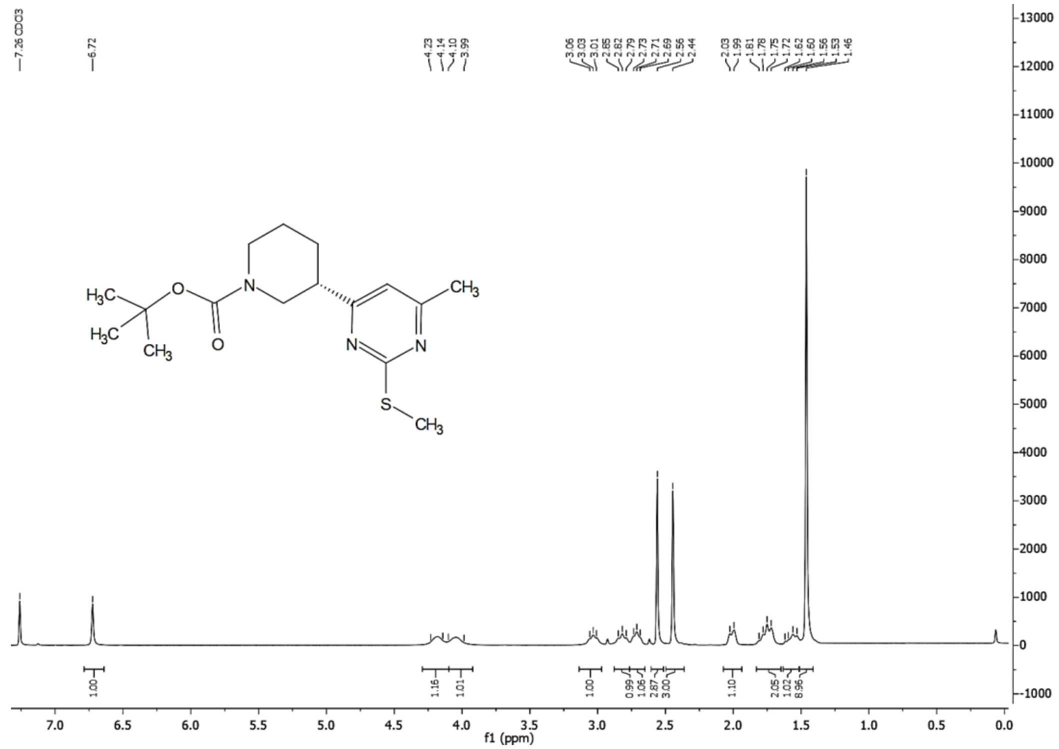

Figure S111. ^{1}H NMR (400 MHz, $CDCl_3$) spectrum of *tert*-butyl (3*S*)-3-[6-methyl-2-(methylsulfanyl)pyrimidin-4-yl]piperidine-1-carboxylate (**12b**)

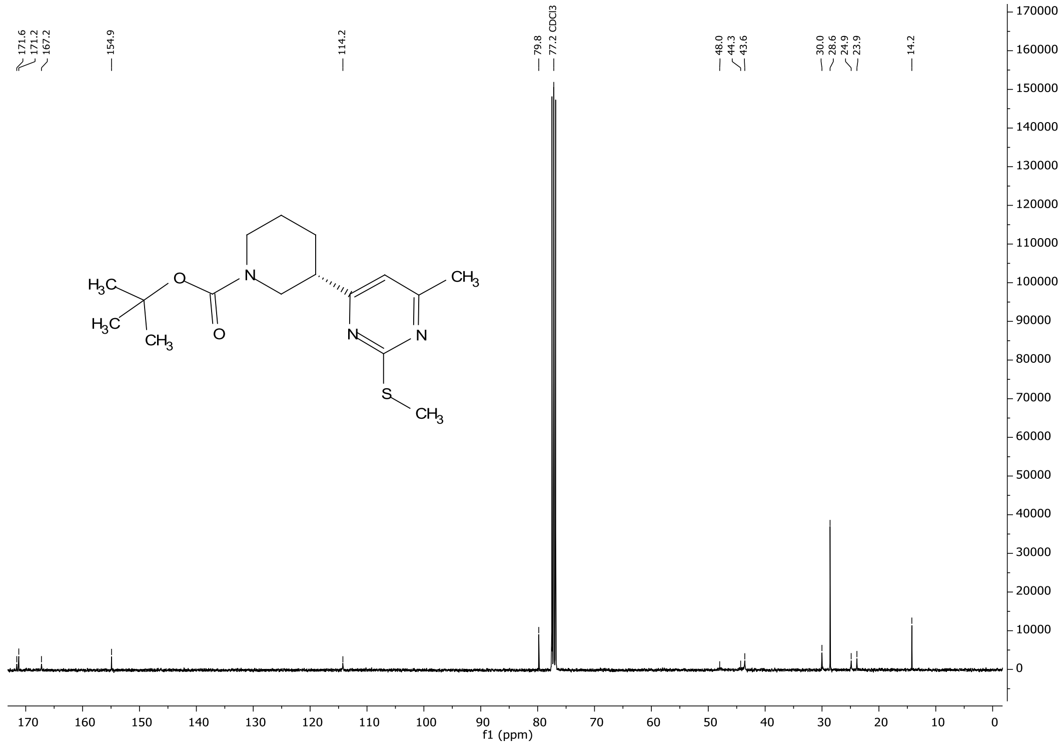

Figure S112. ^{13}C NMR (101 MHz, $CDCl_3$) spectrum of *tert*-butyl (3*S*)-3-[6-methyl-2-(methylsulfanyl)pyrimidin-4-yl]piperidine-1-carboxylate (**12b**)

Compound Spectrum SmartFormula Report

Analysis Info

Analysis Name	D:\Data\PVP-818.d	Acquisition Date	3/30/2026 10:12:22 AM
Method	DirectInfusion_TuneLow_pos.m	Operator	hplc
Sample Name	PVP-818	Instrument	micrOTOF-Q III 8228888.20448
Comment	SB		

Acquisition Parameter

Source Type	ESI	Ion Polarity	Positive	Set Nebulizer	0.4 Bar
Focus	Not active	Set Capillary	4500 V	Set Dry Heater	180 °C
Scan Begin	50 m/z	Set End Plate Offset	-500 V	Set Dry Gas	4.0 l/min
Scan End	1000 m/z	Set Collision Cell RF	140.0 Vpp	Set Divert Valve	Waste

#	RT [min]	Area	Int. Type	I	S/N	Chromatogram	Max. m/z	FWHM [min]
n.a.	0.2	n.a.	Single spectrum	n.a.	n.a.	n.a.	304.2617	n.a.
n.a.	7.0	n.a.	Single spectrum	n.a.	n.a.	n.a.	346.1560	n.a.

+MS, 7.0min #420

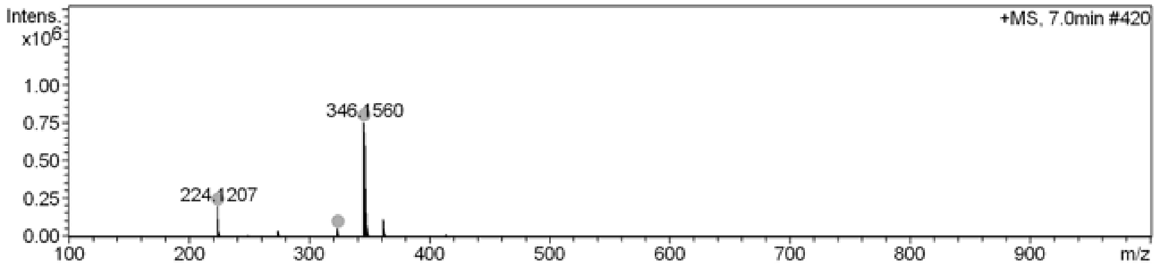

Meas. m/z	#	Ion Formula	m/z	err [ppm]	mSigma	# Sigma	Score	rdb	e⁻ Conf	N-Rule
224.1207	1	C11H18N3S	224.1216	-4.1	7.5	1	100.00	4.5	even	ok
324.1728	1	C16H26N3O2S	324.1740	3.9	5.1	1	100.00	5.5	even	ok
346.1560	1	C16H25N3NaO2S	346.1560	-0.1	13.0	1	100.00	5.5	even	ok

Figure S113. HRMS (ESI-TOF) spectrum of *tert*-butyl (3*S*)-3-[6-methyl-2-(methylsulfanyl)pyrimidin-4-yl]piperidine-1-carboxylate (**12b**)

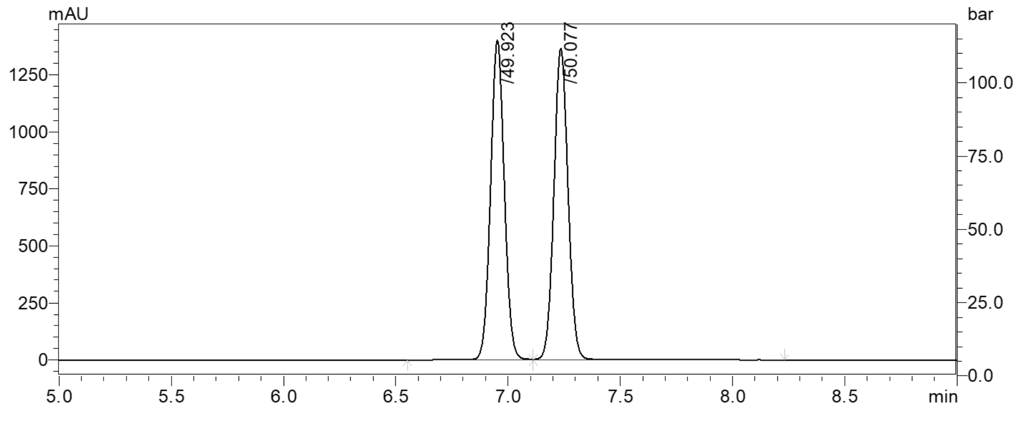

Figure S114. Chiral HPLC analysis of *tert*-butyl (3*S*)-3-[6-methyl-2-(methylsulfanyl)pyrimidin-4-yl]piperidine-1-carboxylate (**12b**). Conditions: CHIRAL ART Cellulose-SJ (100 × 4.6 mm I.D.); S-10 μL; mobile phase: ACN/H_2O + 0.1% HCOOH (70:30→30:70 in 10 min); T = 36 °C; flow rate 1.0 mL/min.

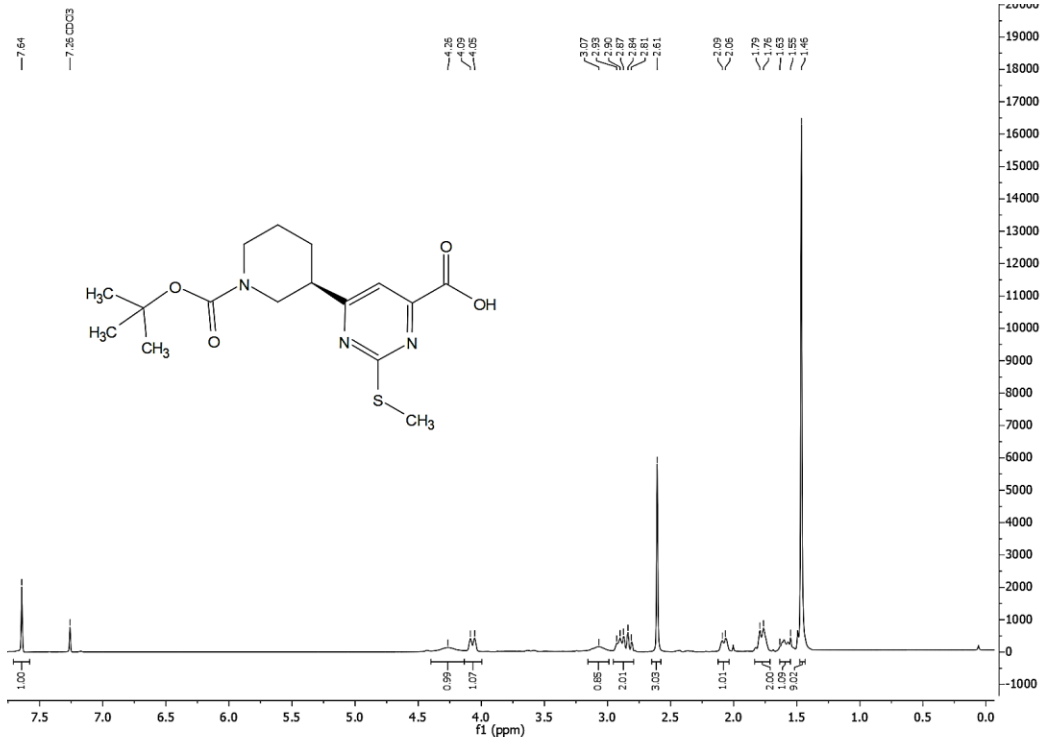

Figure S115. ^{1}H NMR (400 MHz, $CDCl_3$) spectrum of 6-[(3*R*)-1-(*tert*-butoxycarbonyl)piperidin-3-yl]-2-(methylsulfanyl)pyrimidine-4-carboxylic acid (**13a**)

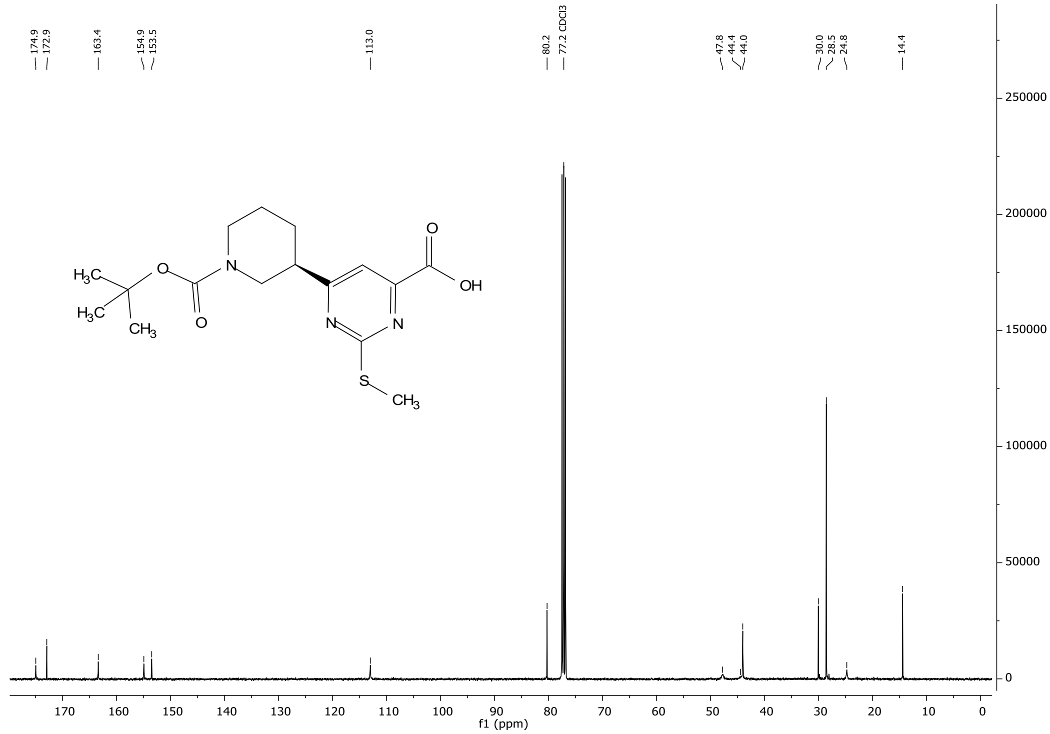

Figure S116. ^{13}C NMR (101 MHz, $CDCl_3$) spectrum of 6-[(3*R*)-1-(*tert*-butoxycarbonyl)piperidin-3-yl]-2-(methylsulfanyl)pyrimidine-4-carboxylic acid (**13a**)

Compound Spectrum SmartFormula Report

Analysis Info

Analysis Name	D:\Data\PVP-836.d	Acquisition Date	3/17/2026 11:56:25 AM
Method	DirectInfusion_TuneLow_pos.m	Operator	hplc
Sample Name	PVP-836	Instrument	micrOTOF-Q III 8228888.20448
Comment	SB		

Acquisition Parameter

Source Type	ESI	Ion Polarity	Positive	Set Nebulizer	0.4 Bar
Focus	Not active	Set Capillary	4500 V	Set Dry Heater	180 °C
Scan Begin	50 m/z	Set End Plate Offset	-500 V	Set Dry Gas	4.0 l/min
Scan End	1000 m/z	Set Collision Cell RF	140.0 Vpp	Set Divert Valve	Waste

#	RT [min]	Area	Int. Type	I	S/N	Chromatogram	Max. m/z	FWHM [min]
n.a.	0.1	n.a.	Single spectrum	n.a.	n.a.	n.a.	304.2618	n.a.
n.a.	4.5	n.a.	Single spectrum	n.a.	n.a.	n.a.	354.1482	n.a.

+MS, 4.5min #268

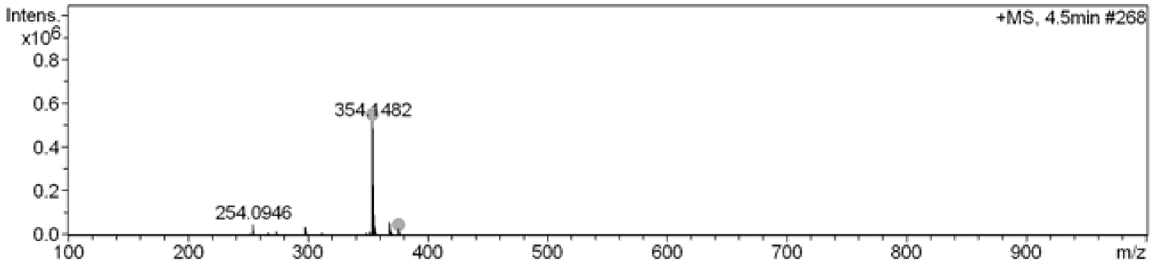

Meas. m/z	#	Ion Formula	m/z	err [ppm]	mSigma	# Sigma	Score	rdb	e⁻ Conf	N-Rule
354.1482	1	C16H24N3O4S	354.1482	0.0	0.9	1	100.00	6.5	even	ok
376.1297	1	C16H23N3NaO4S	376.1301	1.1	9.5	1	100.00	6.5	even	ok

Figure S117. HRMS (ESI-TOF) spectrum of 6-[(3*R*)-1-(*tert*-butoxycarbonyl)piperidin-3-yl]-2-(methylsulfanyl)pyrimidine-4-carboxylic acid (**13a**)

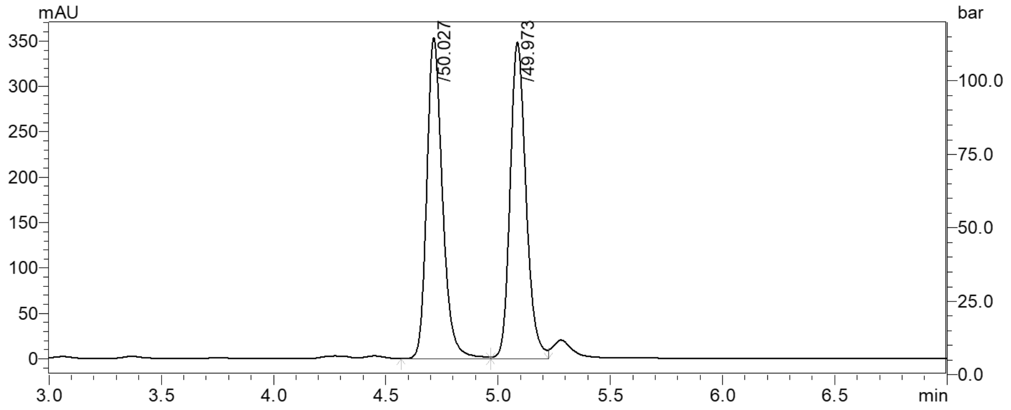

Figure S118. Chiral HPLC analysis of 6-[(3*R*)-1-(*tert*-butoxycarbonyl)piperidin-3-yl]-2-(methylsulfanyl)pyrimidine-4-carboxylic acid (**13a**). Conditions: CHIRAL ART Cellulose-SJ (100 × 4.6 mm I.D.); S-10 µL; mobile phase: ACN/H_2O + 0.1% HCOOH (70:30→30:70 in 10 min); T = 36 ˚C; flow rate 1.0 mL/min.

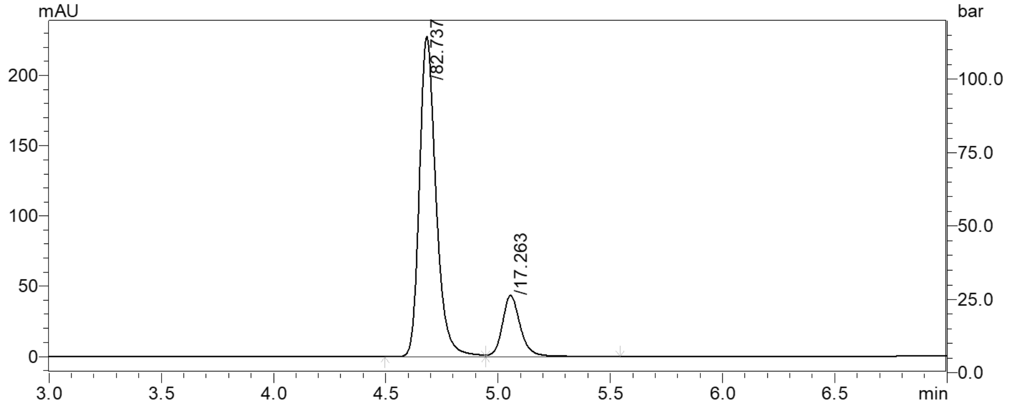

Figure S119. Chiral HPLC analysis of 6-[(3*R*)-1-(*tert*-butoxycarbonyl)piperidin-3-yl]-2-(methylsulfanyl)pyrimidine-4-carboxylic acid (**13’a**). Conditions: CHIRAL ART Cellulose-SJ (100 × 4.6 mm I.D.); S-10 µL; mobile phase: ACN/H_2O + 0.1% HCOOH (70:30→30:70 in 10 min); T = 36 ˚C; flow rate 1.0 mL/min.

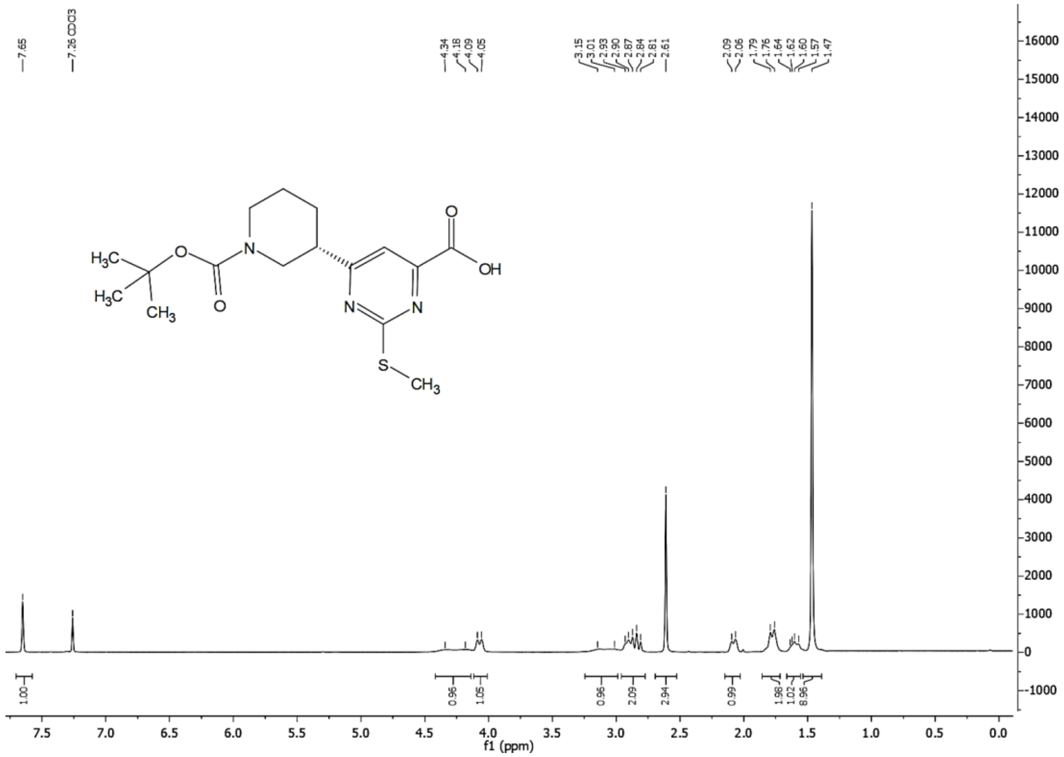

Figure S120. ^{1}H NMR (400 MHz, $CDCl_3$) spectrum of 6-[(3*S*)-1-(*tert*-butoxycarbonyl)piperidin-3-yl]-2-(methylsulfanyl)pyrimidine-4-carboxylic acid (**13b**)

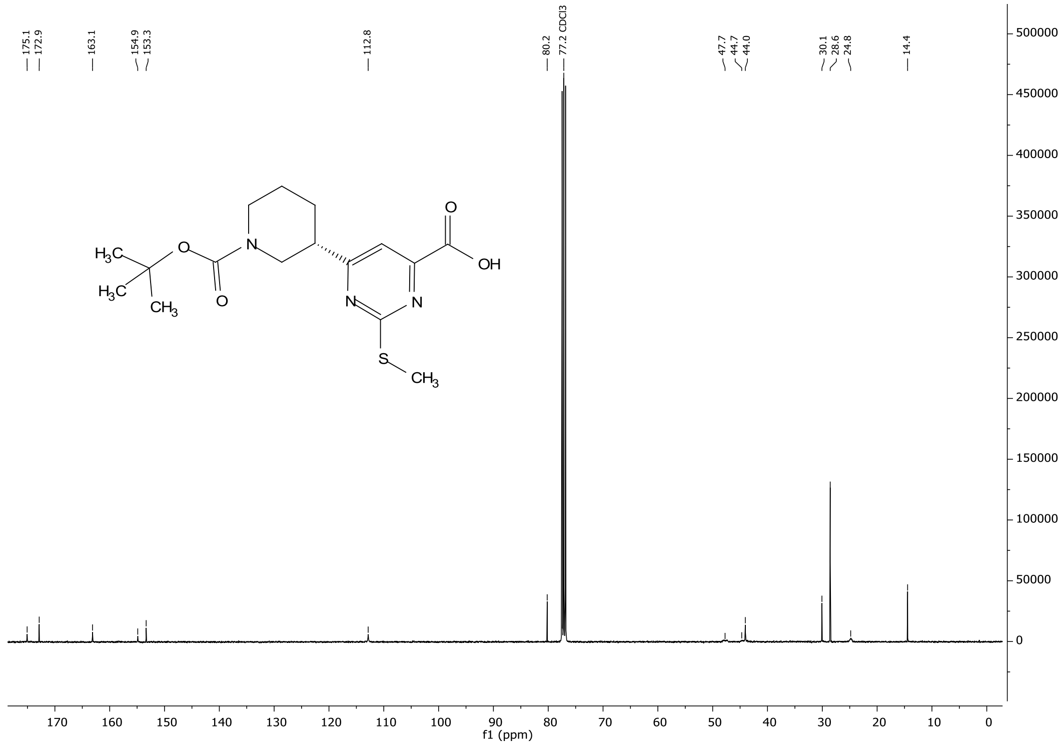

Figure S121. ^{13}C NMR (101 MHz, $CDCl_3$) spectrum of 6-[(3*S*)-1-(*tert*-butoxycarbonyl)piperidin-3-yl]-2-(methylsulfanyl)pyrimidine-4-carboxylic acid (**13b**)

Compound Spectrum SmartFormula Report

Analysis Info

Analysis Name	D:\Data\PVP-862.d	Acquisition Date	3/30/2026 10:45:00 AM
Method	DirectInfusion_TuneLow_pos.m	Operator	hplc
Sample Name	PVP-862	Instrument	micrOTOF-Q III 8228888.20448
Comment	SB		

Acquisition Parameter

Source Type	ESI	Ion Polarity	Positive	Set Nebulizer	0.4 Bar
Focus	Not active	Set Capillary	4500 V	Set Dry Heater	180 °C
Scan Begin	50 m/z	Set End Plate Offset	-500 V	Set Dry Gas	4.0 l/min
Scan End	1000 m/z	Set Collision Cell RF	140.0 Vpp	Set Divert Valve	Waste

#	RT [min]	Area	Int. Type	I	S/N	Chromatogram	Max. m/z	FWHM [min]
n.a.	3.2	n.a.	Single spectrum	n.a.	n.a.	n.a.	304.2615	n.a.
n.a.	8.9	n.a.	Single spectrum	n.a.	n.a.	n.a.	354.1482	n.a.

+MS, 8.9min #536

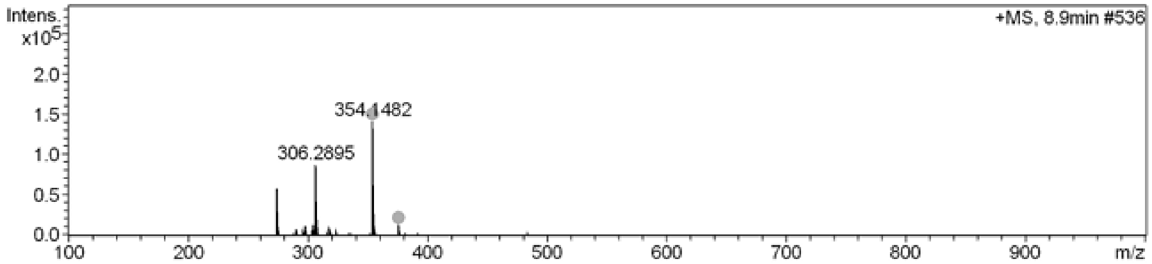

Meas. m/z	#	Ion Formula	m/z	err [ppm]	mSigma	# Sigma	Score	rdb	e⁻ Conf	N-Rule
354.1482	1	C16H24N3O4S	354.1482	-0.1	2.3	1	100.00	6.5	even	ok
376.1304	1	C16H23N3NaO4S	376.1301	-0.7	8.6	1	100.00	6.5	even	ok

Figure S122. HRMS (ESI-TOF) spectrum of 6-[(3*S*)-1-(*tert*-butoxycarbonyl)piperidin-3-yl]-2-(methylsulfanyl)pyrimidine-4-carboxylic acid (**13b**)

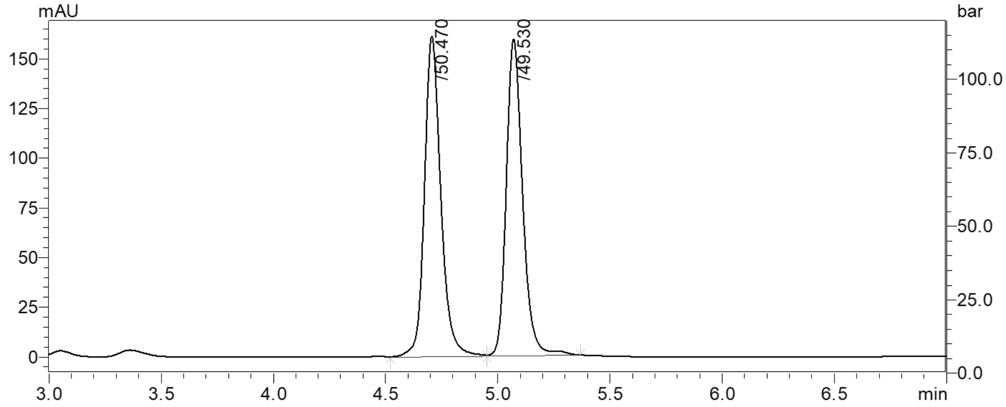

Figure S123. Chiral HPLC analysis of 6-[(3*S*)-1-(*tert*-butoxycarbonyl)piperidin-3-yl]-2-(methylsulfanyl)pyrimidine-4-carboxylic acid (**13b**). Conditions: CHIRAL ART Cellulose-SJ (100 × 4.6 mm I.D.); S-10 μL; mobile phase: ACN/H_2O + 0.1% HCOOH (70:30→30:70 in 10 min); T = 36 ˚C; flow rate 1.0 mL/min.

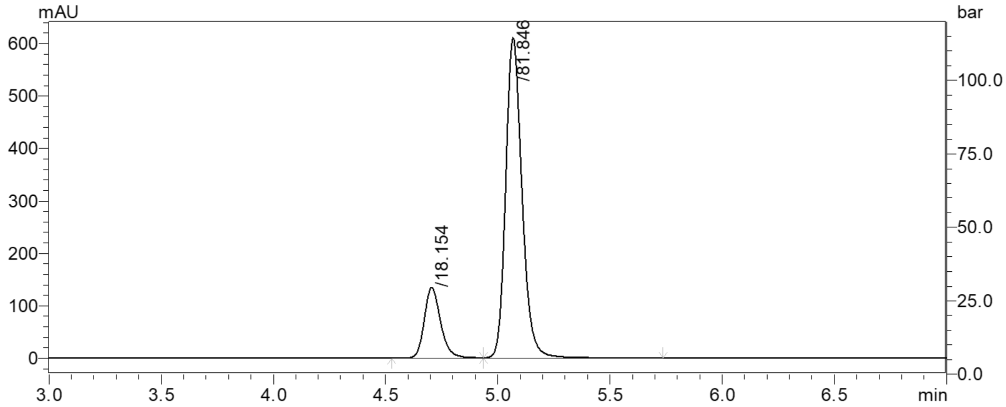

Figure S124. Chiral HPLC analysis of 6-[(3*S*)-1-(*tert*-butoxycarbonyl)piperidin-3-yl]-2-(methylsulfanyl)pyrimidine-4-carboxylic acid (**13’b**). Conditions: CHIRAL ART Cellulose-SJ (100 × 4.6 mm I.D.); S-10 μL; mobile phase: ACN/H_2O + 0.1% HCOOH (70:30→30:70 in 10 min); T = 36 ˚C; flow rate 1.0 mL/min.

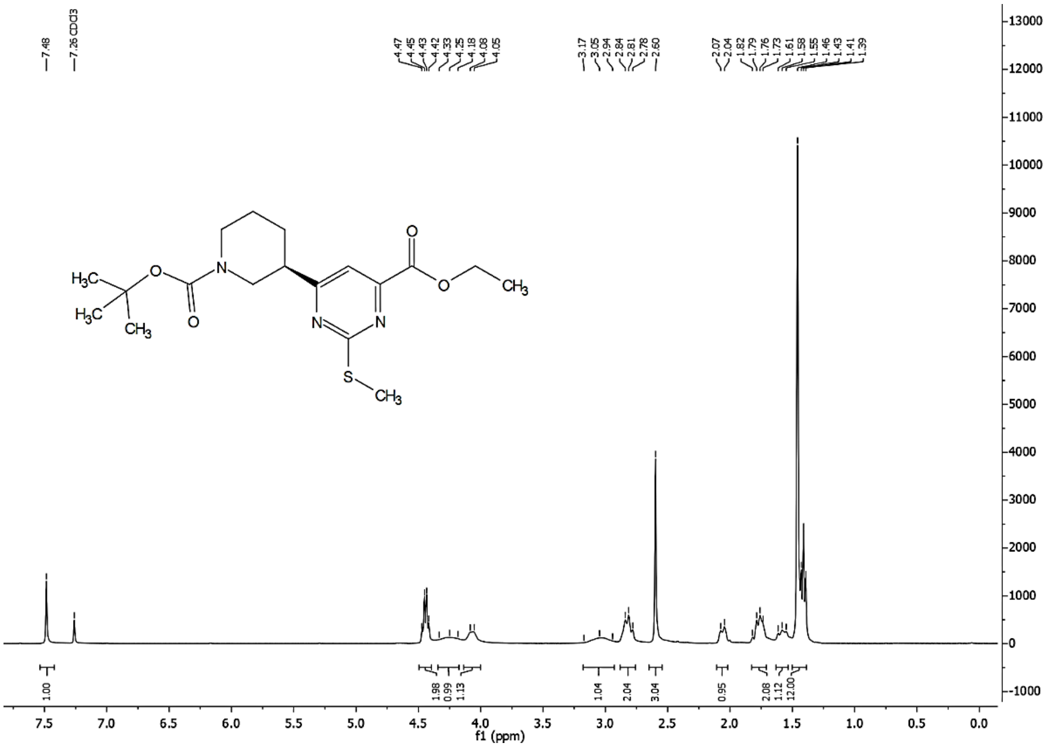

Figure S125. ^{1}H NMR (400 MHz, $CDCl_3$) spectrum of ethyl 6-[(3*R*)-1-(*tert*-butoxycarbonyl)piperidin-3-yl]-2-(methylsulfanyl)pyrimidine-4-carboxylate (**16a**)

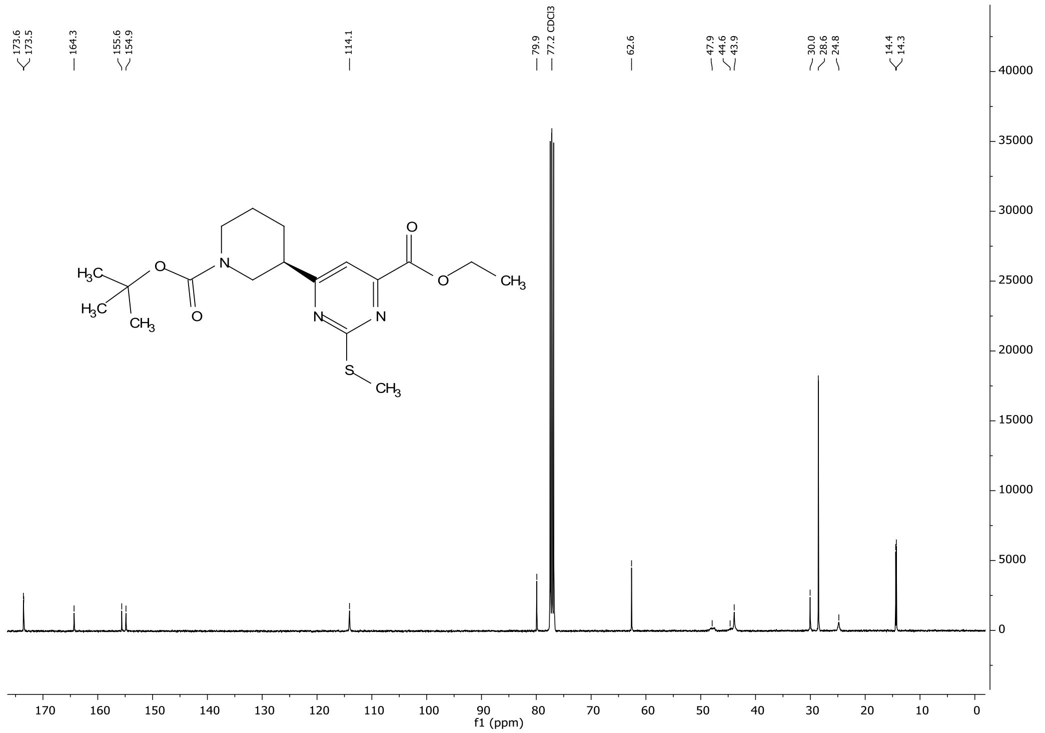

Figure S126. ^{13}C NMR (101 MHz, $CDCl_3$) spectrum of ethyl 6-[(3*R*)-1-(*tert*-butoxycarbonyl)piperidin-3-yl]-2-(methylsulfanyl)pyrimidine-4-carboxylate (**16a**)

Compound Spectrum SmartFormula Report

Analysis Info			
		Acquisition Date	3/26/2026 12:02:03 PM
Analysis Name	D:\Data\PVP-867.d		
Method	DirectInfusion_TuneLow_pos.m	Operator	hplc
Sample Name	PVP-867	Instrument	micrOTOF-Q III 8228888.20448
Comment	SB		

Acquisition Parameter

Source Type	ESI	Ion Polarity	Positive	Set Nebulizer	0.4 Bar
Focus	Not active	Set Capillary	4500 V	Set Dry Heater	180 °C
Scan Begin	50 m/z	Set End Plate Offset	-500 V	Set Dry Gas	4.0 l/min
Scan End	1000 m/z	Set Collision Cell RF	140.0 Vpp	Set Divert Valve	Waste

#	RT [min]	Area	Int. Type	I	S/N	Chromatogram	Max. m/z	FWHM [min]
n.a.	0.2	n.a.	Single spectrum	n.a.	n.a.	n.a.	304.2612	n.a.
n.a.	4.0	n.a.	Single spectrum	n.a.	n.a.	n.a.	382.1795	n.a.

+MS, 4.0min #239

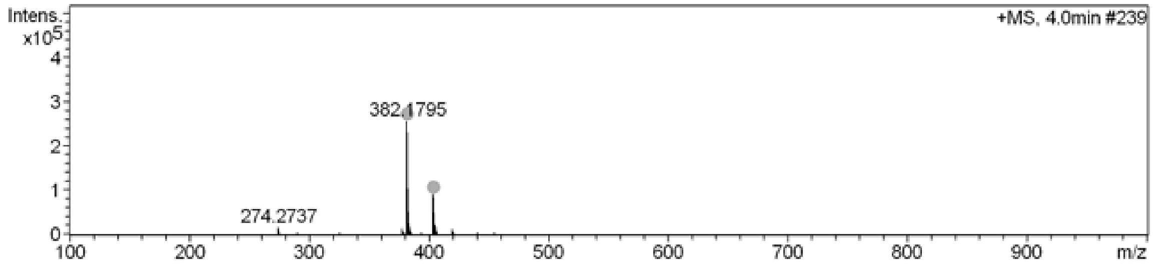

Meas. m/z	#	Ion Formula	m/z	err [ppm]	mSigma	# Sigma	Score	rdb	e⁻ Conf	N-Rule
382.1795	1	C18H28N3O4S	382.1795	-0.1	2.1	1	100.00	6.5	even	ok
404.1614	1	C18H27N3NaO4S	404.1614	-0.2	12.0	1	100.00	6.5	even	ok

Figure S127. HRMS (ESI-TOF) spectrum of ethyl 6-[(3*R*)-1-(*tert*-butoxycarbonyl)piperidin-3-yl]-2-(methylsulfanyl)pyrimidine-4-carboxylate (**16a**)

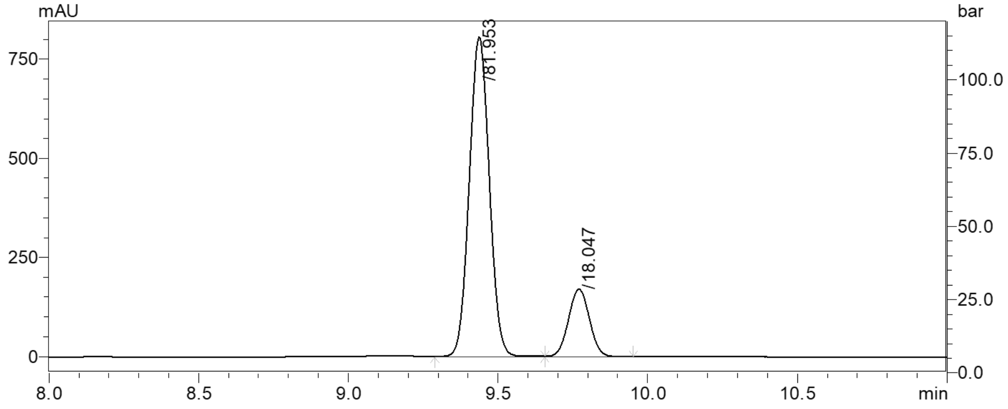

Figure S128. Chiral HPLC analysis of ethyl 6-[(3*R*)-1-(*tert*-butoxycarbonyl)piperidin-3-yl]-2-(methylsulfanyl)pyrimidine-4-carboxylate (**16a**). Conditions: CHIRAL ART Cellulose-SJ (100 × 4.6 mm I.D.); S-10 µL; mobile phase: ACN/H_2O + 0.1% HCOOH (80:20→30:70 in 10 min); T = 36 °C; flow rate 1.0 mL/min.

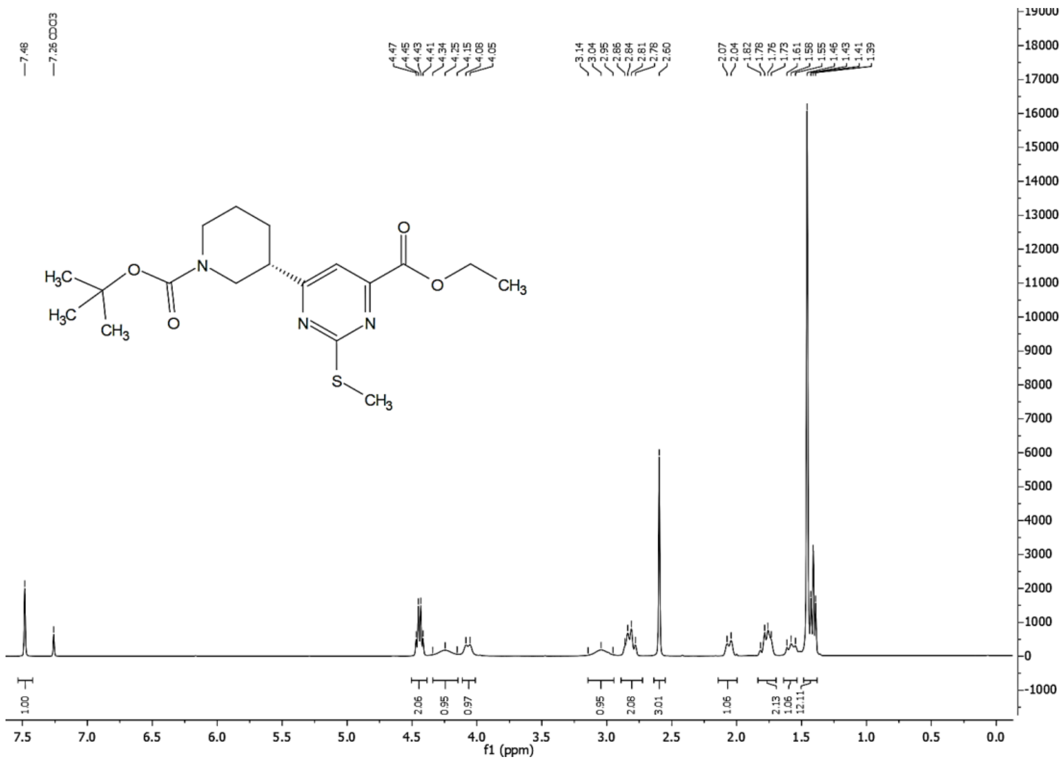

Figure S129. ^{1}H NMR (400 MHz, $CDCl_3$) spectrum of ethyl 6-[(3*S*)-1-(*tert*-butoxycarbonyl)piperidin-3-yl]-2-(methylsulfanyl)pyrimidine-4-carboxylate (**16b**)

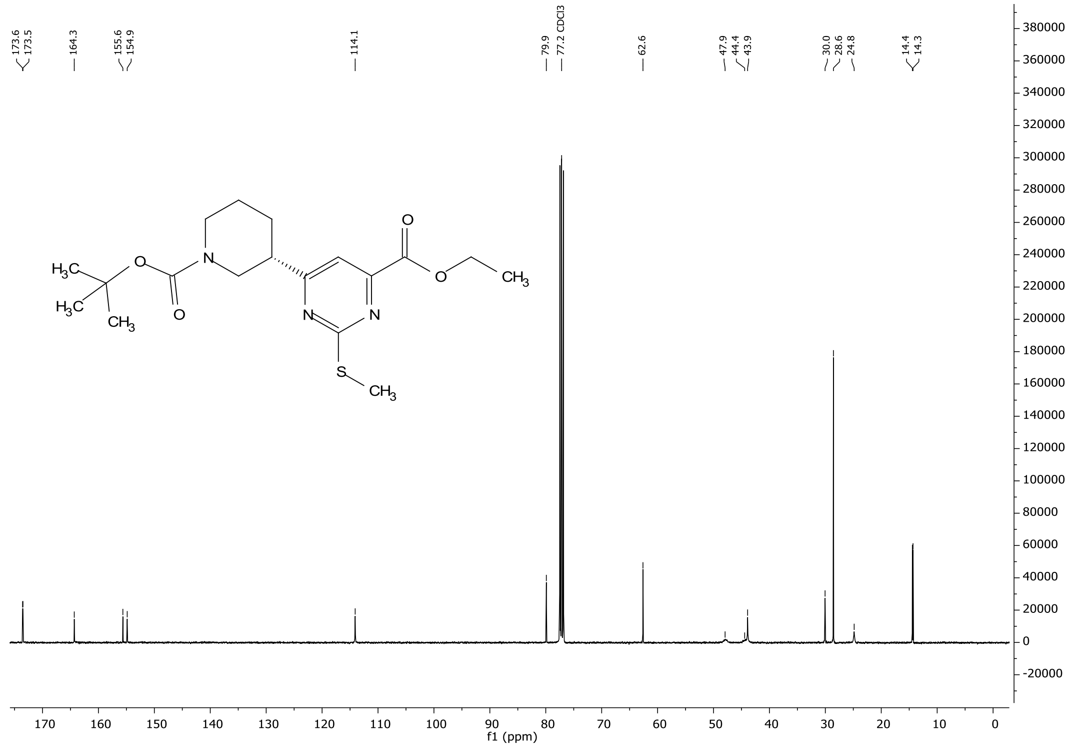

Figure S130. ^{13}C NMR (101 MHz, $CDCl_3$) spectrum of ethyl 6-[(3*S*)-1-(*tert*-butoxycarbonyl)piperidin-3-yl]-2-(methylsulfanyl)pyrimidine-4-carboxylate (**16b**)

Compound Spectrum SmartFormula Report

Analysis Info

Analysis Name	D:\Data\PVP-866.d	Acquisition Date	3/30/2026 11:30:44 AM
Method	DirectInfusion_TuneLow_pos.m	Operator	hplc
Sample Name	PVP-866	Instrument	micrOTOF-Q III 8228888.20448
Comment	SB		

Acquisition Parameter

Source Type	ESI	Ion Polarity	Positive	Set Nebulizer	0.4 Bar
Focus	Not active	Set Capillary	4500 V	Set Dry Heater	180 °C
Scan Begin	50 m/z	Set End Plate Offset	-500 V	Set Dry Gas	4.0 l/min
Scan End	1000 m/z	Set Collision Cell RF	140.0 Vpp	Set Divert Valve	Waste

#	RT [min]	Area	Int. Type	I	S/N	Chromatogram	Max. m/z	FWHM [min]
n.a.	1.3	n.a.	Single spectrum	n.a.	n.a.	n.a.	304.2616	n.a.
n.a.	4.5	n.a.	Single spectrum	n.a.	n.a.	n.a.	404.1614	n.a.

+MS, 4.5min #267

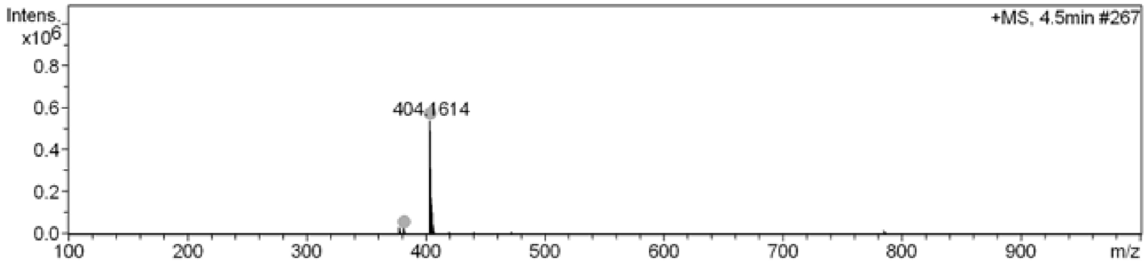

Meas. m/z	#	Ion Formula	m/z	err [ppm]	mSigma	# Sigma	Score	rdb	e⁻ Conf	N-Rule
382.1793	1	C18H28N3O4S	382.1795	-0.5	35.7	1	100.00	6.5	even	ok
404.1614	1	C18H27N3NaO4S	404.1614	-0.2	1.7	1	100.00	6.5	even	ok

Figure S131. HRMS (ESI-TOF) spectrum of ethyl 6-[(3*S*)-1-(*tert*-butoxycarbonyl)piperidin-3-yl]-2-(methylsulfanyl)pyrimidine-4-carboxylate (**16b**)

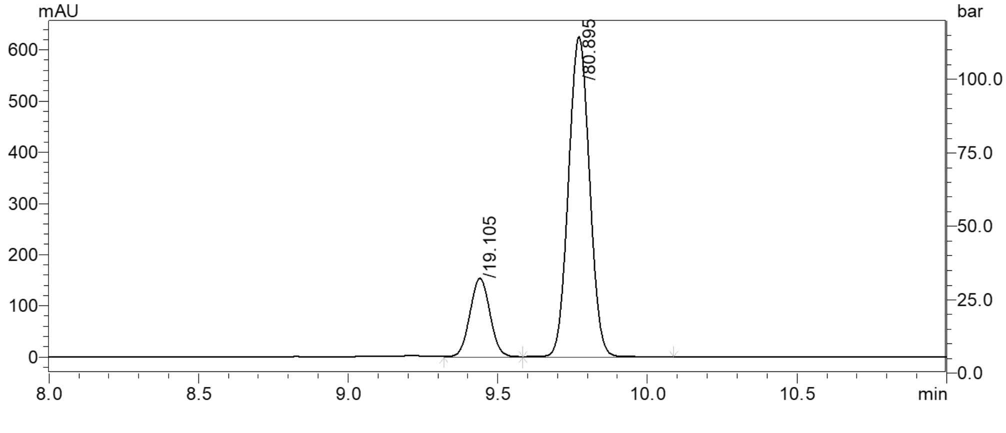

Figure S132. Chiral HPLC analysis of ethyl 6-[(3*S*)-1-(*tert*-butoxycarbonyl)piperidin-3-yl]-2-(methylsulfanyl)pyrimidine-4-carboxylate (**16b**). Conditions: CHIRAL ART Cellulose-SJ (100 × 4.6 mm I.D.); S-10 μL; mobile phase: ACN/H_2O + 0.1% HCOOH (80:20→30:70 in 10 min); T = 36 °C; flow rate 1.0 mL/min.

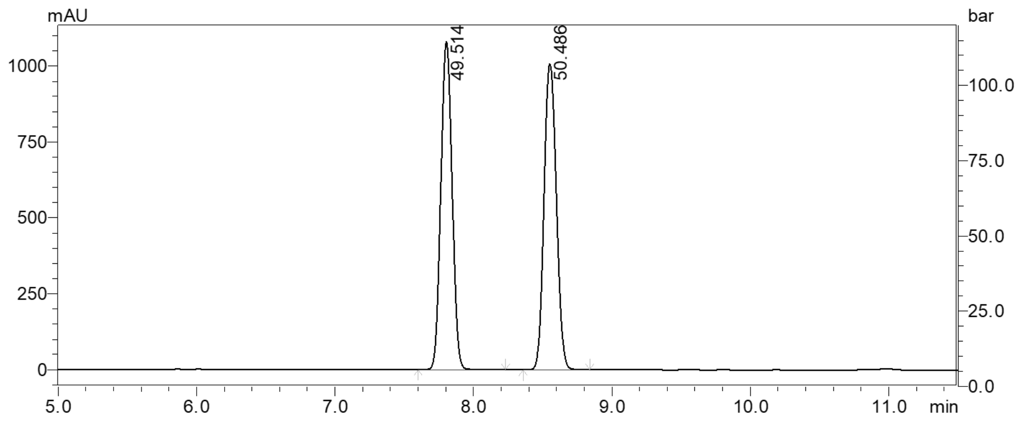

Figure S133. Chiral HPLC analysis of methyl 4-[(3*R*)-1-(*tert*-butoxycarbonyl)piperidin-3-yl]-2-(methylsulfanyl)pyrimidine-5-carboxylate (**4a**) and methyl 4-[(3*S*)-1-(*tert*-butoxycarbonyl)piperidin-3-yl]-2-(methylsulfanyl)pyrimidine-5-carboxylate (**4b**) racemic mixture. Conditions: CHIRAL ART Cellulose-SJ (100×4.6 mm I.D.); mobile phase: ACN/(H_2O + 0.1% HCOOH) (gradient from 30:70 to 70:30 in 10 min); T = 36 °C; flow rate 1.0 mL/min, UV 254 nm.

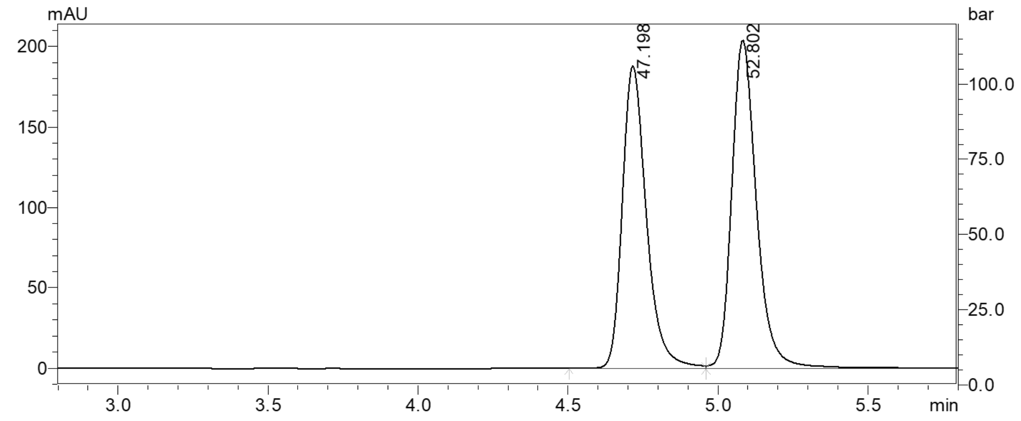

Figure S134. Chiral HPLC analysis of 6-[(3*R*)-1-(*tert*-butoxycarbonyl)piperidin-3-yl]-2-(methylsulfanyl)pyrimidine-4-carboxylic acid (**13'a**) and 6-[(3*S*)-1-(*tert*-butoxycarbonyl)piperidin-3-yl]-2-(methylsulfanyl)pyrimidine-4-carboxylic acid (**13'b**) racemic mixture. Conditions: CHIRAL ART Cellulose-SJ (100×4.6 mm I.D.); mobile phase: ACN/(H_2O + 0.1% HCOOH) (gradient from 30:70 to 70:30 in 10 min); T = 36 °C; flow rate 1.0 mL/min, UV 254 nm.

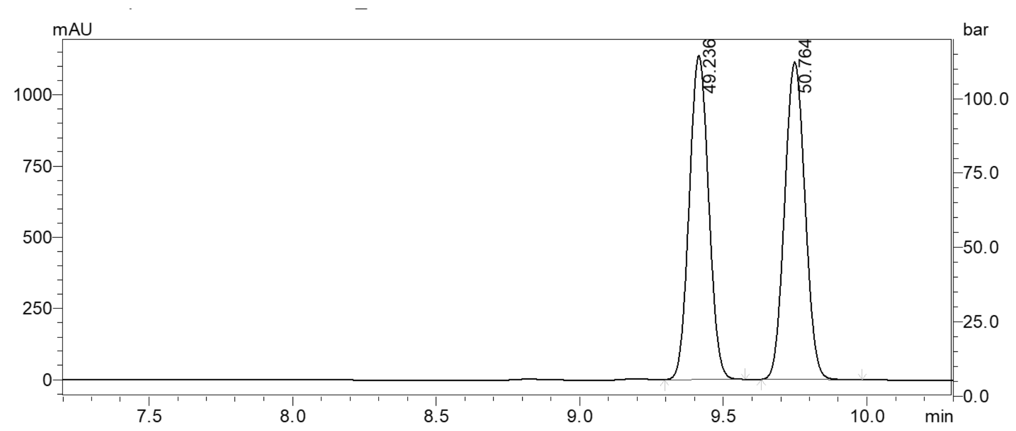

Figure S135. Chiral HPLC analysis of ethyl 6-[(3*R*)-1-(*tert*-butoxycarbonyl)piperidin-3-yl]-2-(methylsulfanyl)pyrimidine-4-carboxylate (**16a**) and ethyl 6-[(3*S*)-1-(*tert*-butoxycarbonyl)piperidin-3-yl]-2-(methylsulfanyl)pyrimidine-4-carboxylate (**16b**) racemic mixture. Conditions: CHIRAL ART Cellulose-SJ (100×4.6 mm I.D.); mobile phase: ACN/(H_2O + 0.1% HCOOH) (gradient from 30:70 to 70:30 in 10 min); T = 36 °C; flow rate 1.0 mL/min, UV 254 nm.

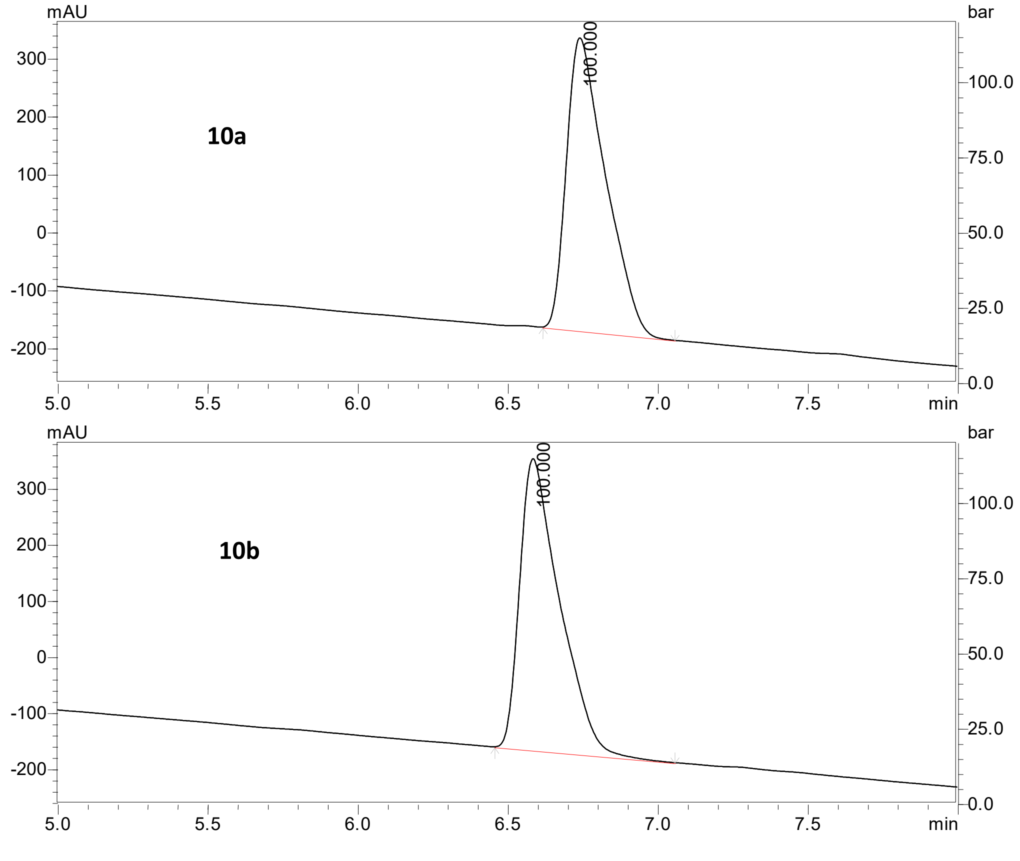

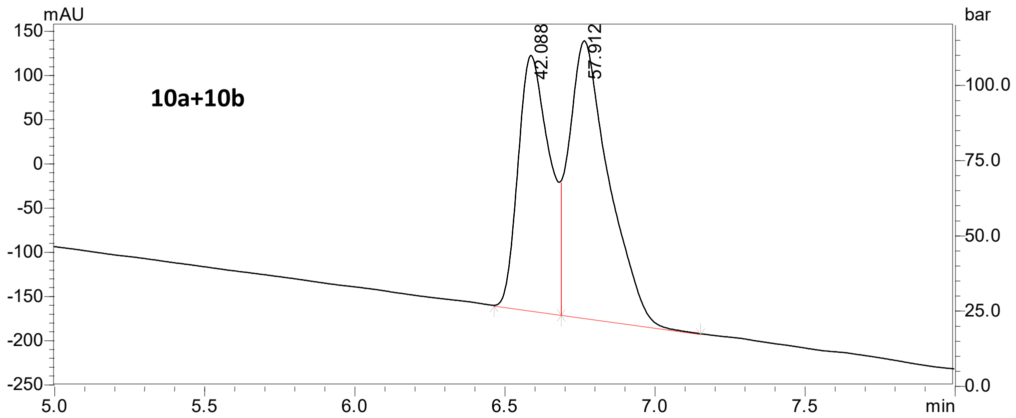

Figure 136. Chiral HPLC analysis of derivatives **10a**, **10b** and the corresponding racemic mixture. Conditions: CHIRAL ART Cellulose-SJ (100×4.6 mm I.D.); mobile phase: ACN/(H_2O + 0.1% HCOOH) (gradient from 20:80 to 60:40 in 10 min); T = 36 °C; flow rate 1.0 mL/min, UV 210 nm.

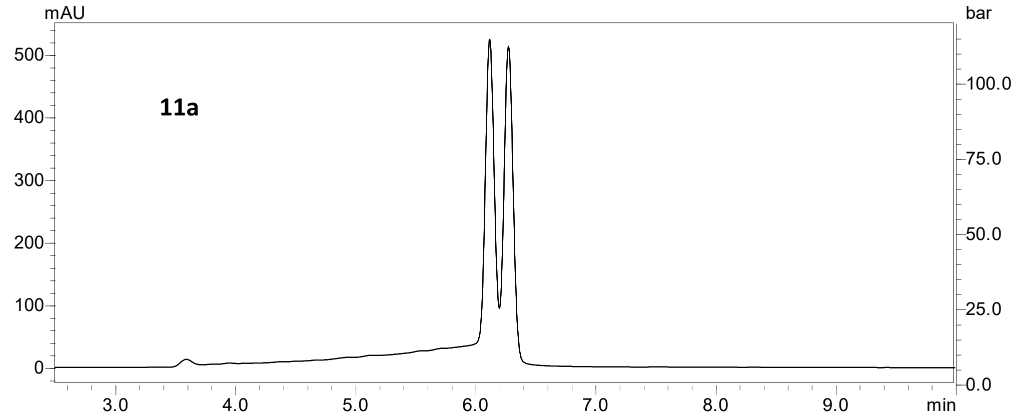

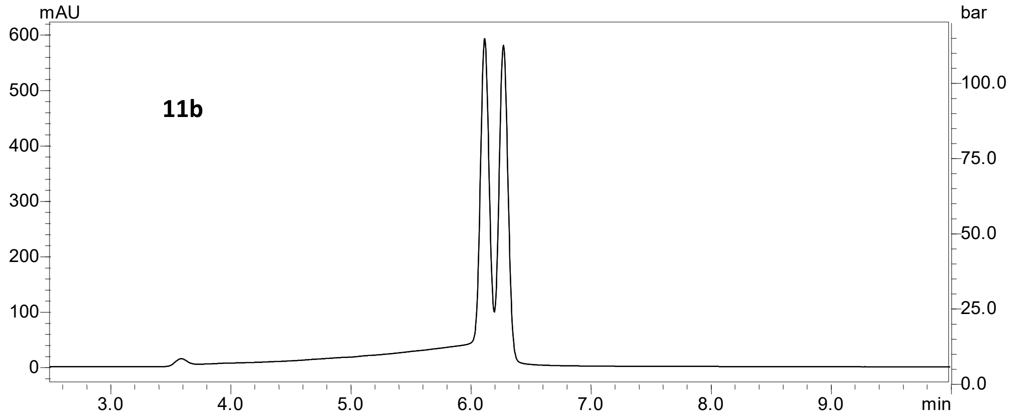

Figure 137. Chiral HPLC analysis of pyrimidine derivatives *tert*-butyl (3*R*)-3-[(1*Z*)-1-hydroxy-3-oxobut-1-en-1-yl]piperidine-1-carboxylate (**11a**) and *tert*-butyl (3*S*)-3-[(1*Z*)-1-hydroxy-3-oxo-but-1-en-1-yl]piperidine-1-carboxylate (**11b**). Conditions: CHIRAL ART Cellulose-SJ (100×4.6 mm I.D.); mobile phase: ACN/(H_2O + 0.1% HCOOH) (gradient from 30:70 to 70:30 in 10 min); T = 20 °C; flow rate 1.0 mL/min, UV 254 nm.

Table S1. Summary of Enantiomeric Excess Values and Chiral HPLC Conditions.

Compound	Absolute configuration	ee (%)	Chiral stationary phase	Mobile-phase gradient	Gradient time (min)	Supplementary Figure
4a	R	98.1	Cellulose-SJ	70:30 → 30:70	10	Figure S8
4b	S	98.0	Cellulose-SJ	70:30 → 30:70	10	Figure S12
4c	R	96.3	Amylose-SA	70:30 → 30:70	10	Figure S16
4d	S	92.4	Amylose-SA	70:30 → 30:70	10	Figure S20
4e	R	98.5	Cellulose-SJ	70:30 → 30:70	10	Figure S24
4f	S	97.1	Cellulose-SJ	70:30 → 30:70	10	Figure S28
4g	R,R	100.0	Cellulose-SJ	70:30 → 30:70	10	Figure S32
4h	S,S	98.6	Cellulose-SJ	70:30 → 30:70	10	Figure S36
4i	R	99.6	Cellulose-SJ	80:20 → 30:70	10	Figure S40
4j	S	94.7	Cellulose-SJ	80:20 → 30:70	10	Figure S44
4k	R	94.1	Cellulose-SJ	80:20 → 30:70	10	Figure S48
4l	S	95.2	Cellulose-SJ	80:20 → 30:70	10	Figure S52
4m	R,R	99.9	Cellulose-SB	70:30 → 20:80	10	Figure S56
4n	S,S	99.2	Cellulose-SB	70:30 → 20:80	10	Figure S60
4o	R	90.2	Cellulose-SB	80:20 → 40:60	10	Figure S64
4p	S	95.2	Cellulose-SB	80:20 → 40:60	10	Figure S68
6a	R	98.4	Cellulose-SJ	80:20 → 30:70	10	Figure S72
6b	S	96.8	Cellulose-SJ	80:20 → 30:70	10	Figure S76
6c	R	96.3	Amylose-SA	70:30 → 30:70	10	Figure S80
6d	S	91.8	Amylose-SA	70:30 → 30:70	10	Figure S84
9a	R	94.0	Cellulose-SJ	70:30 → 30:70	10	Figure S88
9b	S	95.5	Cellulose-SJ	70:30 → 30:70	10	Figure S92
9c	R	93.4	Cellulose-SJ	70:30 → 30:70	10	Figure S96
9d	S	92.7	Cellulose-SJ	70:30 → 30:70	10	Figure S100
12a	R	<1	Cellulose-SJ	70:30 → 30:70	10	Figure S110
12b	S	<1	Cellulose-SJ	70:30 → 30:70	10	Figure S114
13a	R	<1	Cellulose-SJ	70:30 → 30:70	10	Figure S118
13′a	R	65.5	Cellulose-SJ	70:30 → 30:70	10	Figure S119
13b	S	<1	Cellulose-SJ	70:30 → 30:70	10	Figure S123
13′b	S	63.7	Cellulose-SJ	70:30 → 30:70	10	Figure S124
16a	R	63.9	Cellulose-SJ	80:20 → 30:70	10	Figure S128
16b	S	61.8	Cellulose-SJ	80:20 → 30:70	10	Figure S132

Mobile phase: acetonitrile/(water containing 0.1% formic acid). All separations were performed on CHIRAL ART columns (100 × 4.6 mm I.D.) using an LC-2030C chromatograph (Shimadzu, Tokyo, Japan) equipped with UV detection at 254 nm. The column temperature was 36 °C, the flow rate was set to 1.0 mL min^{-1}, the injection volume was 10 µL, and samples were dissolved in methanol.
Abbreviations: ACN, acetonitrile; ee, enantiomeric excess.
